# Autologous NeoHep Derived from Chronic Hepatitis B Virus Patients’ Blood Monocytes by Upregulation of c‐MET Signaling

**DOI:** 10.5966/sctm.2015-0308

**Published:** 2016-07-28

**Authors:** Jashdeep Bhattacharjee, Barun Das, Disha Sharma, Preeti Sahay, Kshama Jain, Alaknanda Mishra, Srikanth Iyer, Puja Nagpal, Vinod Scaria, Perumal Nagarajan, Prakash Khanduri, Asok Mukhopadhyay, Pramod Upadhyay

**Affiliations:** ^1^ National Institute of Immunology, Aruna Asaf Ali Marg, New Delhi, India; ^2^ Institute of Genomics and Integrative Biology, New Delhi, India; ^3^ St. Stephen's Hospital, Tis Hazari, Delhi, India

**Keywords:** HBsAg‐NAT positive blood, Differentiation, Hepatocyte‐like cells, c‐MET signaling, Hepatectomy, Autologous cell therapy

## Abstract

In view of the escalating need for autologous cell‐based therapy for treatment of liver diseases, a novel candidate has been explored in the present study. The monocytes isolated from hepatitis B surface antigen (HBsAg) nucleic acid test (NAT)‐positive (HNP) blood were differentiated to hepatocyte‐like cells (NeoHep) in vitro by a two‐step culture procedure. The excess neutrophils present in HNP blood were removed before setting up the culture. In the first step of culture, apoptotic cells were depleted and genes involved in hypoxia were induced, which was followed by the upregulation of genes involved in the c‐MET signaling pathway in the second step. The NeoHep were void of hepatitis B virus and showed expression of albumin, connexin 32, hepatocyte nuclear factor 4‐α, and functions such as albumin secretion and cytochrome P450 enzyme‐mediated detoxification of xenobiotics. The engraftment of NeoHep derived from HBsAg‐NAT‐positive blood monocytes in partially hepatectomized NOD.CB17‐*Prkdc^scid^*/J mice liver and the subsequent secretion of human albumin and clotting factor VII activity in serum make NeoHep a promising candidate for cell‐based therapy. Stem Cells Translational Medicine *2017;6:174–186*

**Significance Statement**

This is the first report in which normal hepatocyte‐like cells have been generated from blood monocytes of hepatitis B virus‐infected patients without the introduction of any exogenous genetic material. These monocyte‐derived hepatocyte‐like cells possess cytochrome P450 enzyme cascade, suggesting their potential application in a drug‐screening system. Most important, it opens up possibilities for the autologous hepatocyte‐like cell transplantation to support liver functions during the acute conditions of viral hepatitis.

## Introduction

The liver is the largest internal organ, which serves functions such as uptake, metabolism, and elimination of nutrients, xenobiotics, and endogenous toxins. It is one of the visceral organs having the unique property of regeneration.

In some clinical conditions—namely, viral hepatitis, chronic alcoholism, and autoimmune disorders—the liver is damaged irreversibly and fails to regenerate 1. In such situations the orthotropic liver transplantation or split liver transplantation is one of the clinical remedies available for treatment 2. Limited availability of donors and complications associated with posttransplantation, such as the risk of graft rejection, are the major drawbacks of liver transplantation procedure.

Transplantation of cells such as human hepatocytes 3 isolated from cadaveric liver is a less invasive procedure than is organ transplantation. In such allogenic transplantation, risk of the manifestation of graft versus host disease 4 is higher, which can be avoided only by autologous transplantation.

Although it is not possible to isolate hepatocytes from a compromised liver for autologous transplantation, the extrahepatic cells—such as hematopoietic stem cells, mesenchymal stem cells, and healthy monocytes 5—were able to differentiate to hepatocyte‐like cells to restore liver functions in a murine liver disease model 6. In a typical clinical setting the isolation of a therapeutically significant number of stem cells from a diseased individual may not be feasible. Therefore, an alternative source for autologous extrahepatic cells capable of differentiating to hepatocyte‐like cells is highly desirable.

In the present study, monocytes were isolated from hepatitis B surface antigen (HBsAg) and nucleic acid test (NAT) 7 positive peripheral (HNP) blood collected from chronic hepatitis B individuals. The monocytes were differentiated to generate reprogrammed monocytes (RM), followed by differentiating RM to hepatocyte‐like cells (NeoHep). The NeoHep were also generated from healthy blood to demonstrate the equivalence of healthy and HNP NeoHep.

The NeoHep were investigated for the expression of hepatocyte‐specific markers, albumin secretion, hepatocyte nuclear factor 4 (HNF4α), and connexin 32 expressions and cytochrome P450 enzyme activity. The RNA sequencing (RNASeq) of the monocytes, RM, and NeoHep generated from both healthy and HNP monocytes was performed to analyze the kinetics of this differentiation process at the transcript level. The NeoHep were transplanted in partially hepatectomized NOD.CB17‐*Prkdc^scid^*/J (NOD SCID) mice, and their engraftment in the regenerated liver lobe and the secretion of human albumin and clotting factor VII activity in serum were investigated.

## Materials and Methods

The use of healthy human peripheral blood, HBsAg‐NAT‐positive peripheral blood and buffy coat in the present study was approved by the institutional human ethics committee of the National Institute of Immunology. The experiment on NOD.CB17‐*Prkdc^scid^*/J (NOD SCID) mice was approved by the institutional animal ethics committee of the National Institute of Immunology.

### Isolation of Peripheral Blood Mononuclear Cells

Anonymous samples of healthy and HBsAg‐NAT positive human peripheral (HNP) blood were collected from adults (18–55 years of age), and peripheral blood mononuclear cells (PBMCs) were isolated by density gradient centrifugation. Neutrophils, lymphocytes, natural killer cells, and other nonmonocytic cells were removed from the enumerated PBMCs by two consecutive negative magnetic‐assisted cell‐sorting selection procedures. Detailed steps are given in the supplemental online data.

### Cell Culture

The differentiation of monocytes to NeoHep was achieved by using a two‐step differentiation protocol. In the first step, monocytes were differentiated to “reprogrammed monocytes” (RM) in the presence of basal Iscove's modified Dulbecco's medium (IMDM; Thermo Fisher Scientific, Waltham, MA, https://www.thermofisher.com), supplemented with interleukin‐3, macrophage colony‐stimulating factor (Prospec, Ness‐Ziona, Israel, http://www.prospecbio.com), β‐mercaptoethanol (Sigma, St. Louis, MO, https://www.sigmaaldrich.com), and 0.5% embryonic stem cell‐grade fetal bovine serum (eFBS) (Biological Industries, Kibbutz Beit‐Haemek, Israel, http://www.bioind.com). After day 6, RM were differentiated to NeoHep in 15 days in the presence of IMDM, supplemented with epithelial growth factor (Prospec), hepatocyte growth factor (HGF), fibroblast growth factor‐4, linoleic acid (Sigma), and eFBS. Detailed steps are given in the supplemental online data.

### Abundance of Monocyte and Neutrophils in Healthy and HNP Blood‐Derived PBMCs

One million PBMCs isolated from healthy and HNP blood were incubated with phycoerythrin‐conjugated CD14 and fluorescein isothiocyanate‐conjugated CD66b (BD Biosciences, San Jose, CA, http://www.bdbiosciences.com), according to the manufacturer's instructions. The cells were washed after incubation with phosphate‐buffered saline (PBS) and analyzed with a flow cytometer. The abundance of CD14‐ (monocyte marker) and CD66b‐ (neutrophil marker) positive cells was depicted in a dot plot.

### Annexin A5 Staining

Annexin A5 staining was performed using Annexin A5 apoptosis detection kit (FITC Annexin V Apoptosis Detection Kit I; BD BioSciences). Detailed steps are given in the supplemental online data.

### Extraction of Genomic DNA and End‐Point Polymerase Chain Reaction

Genomic DNA from cells and viral DNA from plasma were extracted with the MasterPure Complete DNA and RNA Purification Kit (Epicentre, Madison, WI, http://www.epibio.com), according to the manufacturer's instructions. End‐point polymerase chain reaction (PCR) was performed with Phusion High‐Fidelity PCR Master Mix (Thermo Fisher) to amplify amplicons specific to human *Gapdh*, *Hbsag*, and *Hbxag*. The amplified amplicons were resolved in 3% agarose gel (Sigma).

### Viral Load Determination

A region of conserved hepatitis B virus (HBV) genome sequence, specific to hepatitis B surface antigen (HBsAg), was synthesized as a positive control of known concentration. Serial dilutions of synthesized standard oligonucleotides were made, and quantitative reverse transcription (qRT)‐PCR was carried out by using the SYBR Green detection system (Thermo Fisher) with HBsAg primers. The viral load of 24 anonymous HNP samples was subsequently determined through the same qRT‐PCR. The sequences of the positive control and the primers are given in the supplemental online data.

### Immunocytochemistry

Cells were washed twice in PBS and fixed in 4% formaldehyde (Himedia, Mumbai, India, http://www.himedialabs.com). Cells were permeabilized in 0.2% tritonX‐100 (Amresco, Cleveland, OH, https://www.amresco-inc.com) and blocked in 1% bovine serum albumin (Himedia), followed by incubation in primary antibodies such as antihuman albumin (Thermo Fisher Scientific), antihuman connexin 32, (Abcam, Cambridge, U.K., http://www.abcam.com), and antihuman hepatocyte nuclear factor 4 (HNF4α) (Santa Cruz Biotechnology, Dallas, TX, http://www.scbt.com), in a humidified chamber at 37°C for 1 hour. The cells were incubated with secondary antibodies conjugated with either Alexa Fluor 488 or Alexa Fluor 594 (both Thermo Fisher Scientific) in a humidified chamber at 37°C for 30 minutes. The nucleus was counterstained with 4′,6‐diamidino‐2‐phenylindole (DAPI; Himedia). Detailed steps are given in the supplemental online data.

### Functional Activity Assays

The P450 mediated detoxification was examined by Pentoxyresorufin O‐dealkylase assay, and the induction of CYP1A2 in monocytes and NeoHep was estimated using the P450‐Glo CYP1A2 assay (Promega, Madison, WI, http://www.promega.com). Microsomes from NeoHep were isolated, and a P450‐Glo CYP3A4‐pentafluoro‐benzyl ether (PFBE) induction/inhibition assay (Promega) was performed. Human albumin and human clotting factor VII activity were detected by using commercial enzyme‐linked immunosorbent assay (ELISA) kits (KOMA BIOTECH, Seoul, Korea, http://www.komabiotech.com) and a factor VII chromogenic activity assay kit (Assaypro, St. Charles, MO, http://www.assaypro.com), respectively. Assays were performed according to the manufacturer's protocol, and detailed steps are given in the supplemental online data.

### Extraction and Storage of RNA by Spin‐Column Method

The total RNA from sorted monocytes, RM, and NeoHep were isolated using Fisher BioReagents SurePrep RNA/DNA/Protein Purification Kit (Thermo Fisher, Waltham, MA, https://www.fishersci.com), according to the manufacturer's instructions. The RNA was then collected and stored in an RNA stable tube (Biomatrica, San Diego, CA, http://www.biomatrica.com).

### RNA Sequencing and Data Analysis

Monocytes sorted from healthy human peripheral blood (*n* = 5) and HNP blood (*n* = 6) were used to generate RM and NeoHep. Poly A‐tailed RNA isolated from each cell type was pooled separately for healthy and HNP samples. The pooled RNA was sequenced using the ILLUMINA HisEquation 2000 platform (Illumina, San Diego, CA, http://www.illumina.com/). The detailed description of RNA sequencing and subsequent data analysis 8 are provided in the supplemental online data.

### Validation of RNA Sequencing Data by qRT‐PCR

qRT‐PCR was performed to validate RNA sequencing data, using the same RNA samples used for RNA sequencing for the following markers: *Glul*, *Rest*, *Parg*, CD14, CD36, *Myd88*, CD93, and CD33. The qRT‐PCR was performed by using MESA GREEN qPCR kit (Eurogentech, Fremont, CA, http://www.eurogentec.com) with SYBR assay. The expression of genes in RM and NeoHep were calculated using the ∆∆C_t_ method with respect to monocytes. Experimental details and the primer sequence have been provided in the supplemental online data.

### qRT‐PCR of Differentially Expressed Genes in RM and NeoHep

The differential expression of genes in RM and NeoHep in comparison with monocytes was analyzed by using qRT‐PCR, as mentioned above. The genes analyzed were *Hif1α*, *Met*, *Chd7*, *Eed*, *Ehmt1*, *Ezh2*, *Smarca4*, *Suz12*, *Epas1*, *Parp1*, *Sall4*, *Fbxo15*, *Gab1*, *Ptprm*, *Src*, *Plcg1*, *Ranbp9*, *Pik3r1*, *Grb2*, and *Shc1*. Experimental details and the primer sequence have been provided in the supplemental online data.

### One‐Third Partial Hepatectomy and Transplantation of Cells in NOD SCID Mice

The left lateral lobe of NOD SCID mouse liver was excised to perform one‐third partial hepatectomy. Immediately after the hepatectomy, sorted monocytes, RM, and NeoHep were transplanted in NOD SCID mice by splenic route to investigate engraftment and stability of the cells in vivo. To investigate the homing locations of transplanted human cells in mouse tissue samples, we performed RT‐PCR using human glyceraldehyde‐3‐phosphate dehydrogenase (*Gapdh*) and human tumor necrosis factor (*Tnf*)‐α‐specific primer and probe sets. Detailed steps are given in the supplemental online data.

### Fluorescence In Situ Hybridization to Detect Human Nucleus in Human Cell Transplanted NOD SCID Mouse Liver Sections

Fluorescence in situ hybridization was performed using ready‐to‐use (RTU) Human Specific‐Centro Probes Biotin Chromosome 9 (Cambio, Cambridge, U.K., http://www.cambio.co.uk/) in the cryosections of hepatectomized NOD SCID mouse liver transplanted with human cells. The hybridization procedure was followed according to the manufacturer's instruction, and detailed steps are given in the supplemental online data.

### Live Cell Imaging

Cell IQ (CM Technologies, Tampere, Finland) single‐label fluorescence was used to monitor the differentiation process of monocytes to NeoHep for a period of 16 days continuously at a particular selected field with routine change of media. Detailed steps are given in the supplemental online data.

### Statistical Analysis

Graphpad Prism 5 software (Graphpad, La Jolla, CA, http://www.graphpad.com) was used to plot graphs and analyze data; details are provided in the supplemental online data.

## Results

### The Isolation and Culture of Monocytes

#### Higher Neutrophil Counts in Peripheral Blood of Hepatitis B Patients

**Figure 1:**
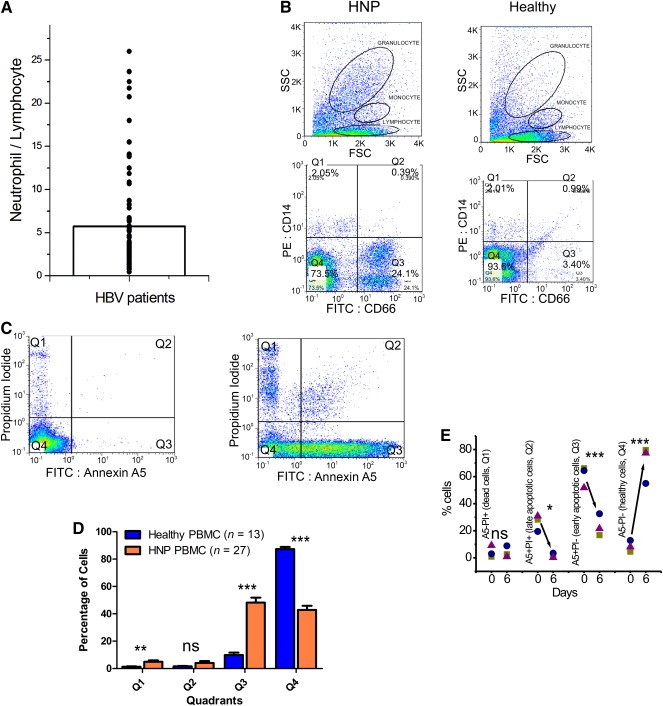
Neutrophils and apoptotic cells in healthy and hepatitis B surface antigen (HBsAg) nucleic acid test (NAT) positive (HNP) peripheral blood mononuclear cells (PBMCs). **(A):** Neutrophil to lymphocyte ratio of hepatitis B patients (*n* = 84). **(B):** Representative flow cytometric dot plots in arbitrary units on a linear scale showing forward scatter and side scatter (upper panel) of PBMCs derived from HBsAg‐NAT positive (HNP) and healthy PBMCs; regions represent the corresponding granulocyte, monocyte, and lymphocyte populations. In the lower panel, dot plots represent fluorescence intensity of respective PBMCs dual‐stained with anti‐CD66b‐fluorescein isothiocyanate in *x*‐axis and anti‐CD14‐PE in *y*‐axis, measured in arbitrary units on log scale. Quadrant gates were created using unstained and single‐color positive controls. **(C):** Representative Annexin A5 versus propidium iodide staining of healthy and HNP PBMCs. **(D):** Apoptotic cells in healthy and HNP PBMCs. Overall summary of number of samples is shown. **(E):** Kinetics of HNP monocyte culture, depicting how the percentage of apoptotic cells changes during initial 6 days of culture in three typical samples. ∗, *p* < .05; ∗∗, *p* < .01; ∗∗∗, *p* < .001. Abbreviations: FITC, fluorescein isothiocyanate; FSC, forward scatter; HBV, hepatitis B virus; HNP, hepatitis B surface antigen nucleic acid test positive; NeoHep, hepatocyte‐like cells; ns, not significant; PBMC, peripheral blood mononuclear cell; PE, phycoerythrin; PI, propidium iodide; SSC, side scatter.

In a few initial experiments, we isolated PBMCs from healthy and HNP blood by density gradient centrifugation. It was followed by a plastic adherence technique to enrich the monocyte population. We found that the culture of such monocytes isolated from HNP blood could not last for more than 2–3 days. In order to investigate the reason behind this limitation, we retrospectively analyzed 2 years of data (March 2013 to February 2015) on hepatitis B positive patients (excluding patients who had sepsis or bleeds), who visited St. Stephen's Hospital, Delhi. The neutrophil to lymphocyte ratio (NLR) for these patients is shown in Figure 1A. The mean NLR was 6.62 ± 2.77 (*n* = 84), which reaffirms the fact that typically, the HBV patients have a higher neutrophil count 9.

Because of enhanced neutrophil counts in the HNP blood, additional steps were required for the isolation of monocytes. The density gradient isolation of PBMCs is known to deplete neutrophils, as is shown in Figure 1B, but owing to a very high proportion of neutrophils in HBV patient's blood in comparison with healthy blood, the forward scatter versus side scatter plot in HNP blood was dominated mainly by neutrophils. This was further confirmed by the higher abundance of CD66b‐positive cells, which is a typical neutrophil marker.

In order to isolate monocytes from HNP blood, the excess neutrophils (CD66b+ve) were first depleted from the PBMCs. It was followed by the removal of the entire nonmonocytic cells to yield a homogeneous population of monocytes. Thereafter, the percentage of apoptotic and dead cells in the HNP PBMC‐derived culture was estimated.

#### Fate of Apoptotic Cells in HNP PBMC Culture

It was observed that most of the apoptotic cells died and were depleted during the initial 6 days in the HNP blood‐derived culture, unlike the healthy blood‐derived culture. The plots obtained after Annexin A5 staining (Fig. 1C, 1D) confirmed that the percentage of apoptotic cells was higher among HNP PBMCs than it was among healthy PMBCs.

However, it was interesting to note that the percentage of apoptotic cells decreased remarkably during the initial 6 days of HNP monocyte culture. A summary of data obtained from three typical cultures is shown in Figure 1E.

#### Yield of Differentiated Cells

**Figure 2:**
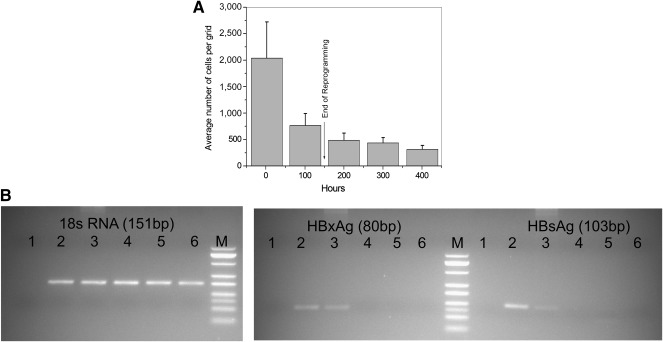
Culture of monocytes isolated from healthy and hepatitis B surface antigen nucleic acid test positive (HNP)‐derived peripheral blood mononuclear cells. **(A):** Average cell density per grid during the differentiation of monocyte (0 hour) to hepatocyte‐like cells (NeoHep) (400 hours) in a reference culture. **(B):** Absence of hepatitis B virus gene in differentiated HNP monocytes. In all three gel images the numbers refer to lanes as follows: **1**, nontemplate control; **2**, HNP serum; **3**, day 0 HNP monocytes; **4**, HNP reprogrammed monocytes (day 6); **5**, HNP NeoHep (day 21); **6**, day 0 healthy monocytes; **M**, Low‐range (25–500) base pair DNA marker. Abbreviations: bp, base pair; HBsAg, hepatitis B surface antigen; HBV, hepatitis B virus; HNP, hepatitis B surface antigen nucleic acid test positive; PBMC, peripheral blood mononuclear cell.

The percentage yield of the NeoHep varied from 5% to 15% of cells present at the beginning of differentiation, irrespective of healthy or HNP monocytes, as is shown in Figure 2A.

#### Absence of Hepatitis B Viral Gene in the Genome of Differentiated Cells

One of our major concerns was to confirm the absence of the HBV genome from the RM and NeoHep derived from HNP monocytes. Whereas the median viral load in HNP blood samples was 2.17E6 copies per milliliter (*n* = 24; minimum = 237,450; maximum = 1.96E10; interquartile range = 9.72E6) and the PCR amplicon specific to *HBsAg* and *HBxAg* was present in HNP monocyte genomic DNA, no *HBsAg* or *HBxAg* was detected in HNP RM and HNP NeoHep genomic DNA (Fig. 2B), confirming that cells having hepatitis B viral genes in their genome were eliminated during the differentiation process.

### Intracellular Hepatic Markers

**Figure 3:**
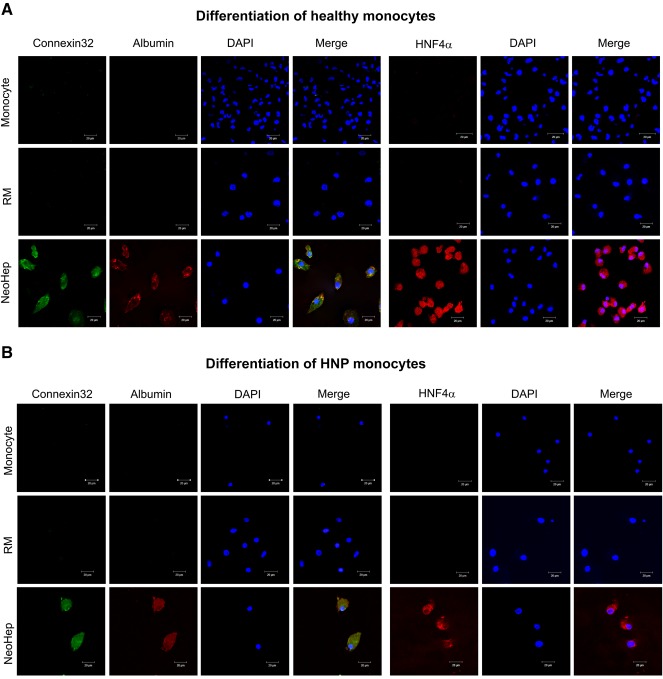
Immunocytochemistry for connexin 32, albumin, and hepatocyte nuclear factor 4 (HNF4α) in monocytes, reprogrammed monocytes, and hepatocyte‐like cells derived from healthy monocytes **(A)** and HNP monocytes **(B)**. Cells were stained with anticonnexin 32, antialbumin, and anti‐HNF4α (1:100) antibody followed by donkey anti‐sheep IgG (Alexa Fluor 488) for connexin 32 and donkey anti‐goat IgG (Alexa Fluor 594) for albumin and HNF4α (1:200). The images were visualized and captured with ZEISS LSM 510 META confocal laser scanning microscope under Plan Apochromat ×63/1.4 oil objective. Scale bar denotes 20 µm for all panels. Abbreviations: DAPI, 4′,6‐diamidino‐2‐phenylindole; HNF4α, hepatocyte nuclear factor 4; HNP, hepatitis B surface antigen nucleic acid test positive; NeoHep, hepatocyte‐like cells; RM, reprogrammed monocytes.

The expression of albumin, connexin 32, and hepatocyte nuclear factor 4α (HNF4α), which are hepatocyte specific markers, was observed only in NeoHep. The images are depicted in Figure 3A and 3B, in which panels show immunostained images obtained from healthy and HNP monocytes. The NeoHep differentiated from healthy monocytes or HNP monocytes showed similar expression of the hepatic markers.

### Functional Activity and Expression in Differentiated Cells

#### Induction of CYP1A2 Activity in NeoHep by Benzo(α)pyrene

**Figure 4:**
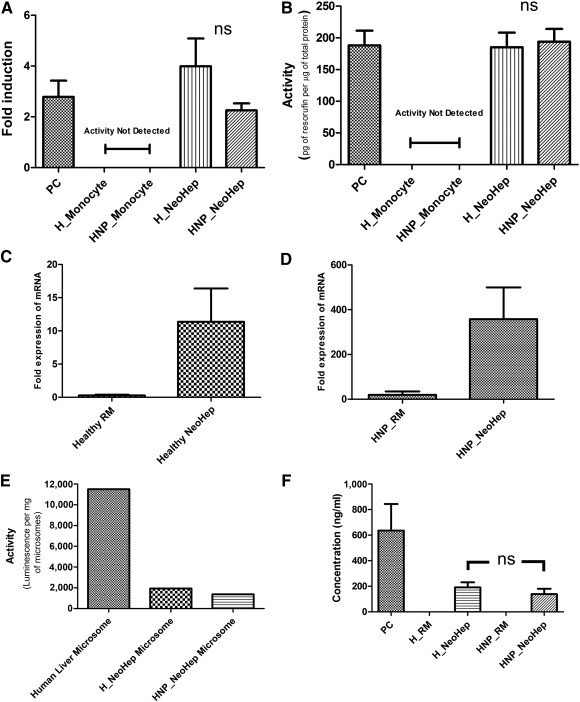
Functional activity and expression in differentiated monocytes. **(A):** Fold increase of CYP1A2 activity after induction with benzo(a)pyrene for 48 hours with that of uninduced monocytes and hepatocyte‐like cells (NeoHep) from healthy and hepatitis B surface antigen nucleic acid test positive (HNP) peripheral blood mononuclear cell (PBMCs) and positive control (PC; HUH 7) (*n* = 5). **(B):** Detoxification activity of 7‐pentoxy resorufin, a CYP2B6 substrate to resorufin by HepG2 (PC), monocytes, and NeoHep derived from healthy and HNP PBMCs (*n* = 5). The amount of resorufin produced was deduced from resorufin standard curve, and the activity was measured in terms of total protein content of cell lysate. **(C, D):** Relative quantification of CYP3A4 mRNA, normalized with *Gapdh*, in reprogrammed monocytes (RM) and NeoHep with respect to monocyte derived from healthy and HNP samples, respectively (*n* = 5). **(E):** Activity of CYP3A4 (Luciferin‐pentafluoro‐benzyl ether [PFBE]) in microsomal fraction isolated from healthy NeoHep, HNP NeoHep (5 pooled samples in each case), and human liver (50 pooled samples). The CYP3A4 activity is measured in terms of luminescence units produced, upon incubation with its substrate Luciferin‐PFBE, per microgram of total microsomal protein content. **(F):** Amount of secreted human albumin in the culture supernatant of HepG2 (PC), H_RM, HNP_RM, healthy NeoHep, and HNP NeoHep (*n* = 3). The error bars represent standard error of the mean. ∗, *p* < .05; ∗∗, *p* < .01; ∗∗∗, *p* < .001. Abbreviations: H, hepatitis; HNP, hepatitis B surface antigen nucleic acid test positive; NeoHep, hepatocyte‐like cells; ns, not significant; PC, positive control; PFBE, pentafluoro‐benzyl ether; RM, reprogrammed monocytes.

The CYP1A2 enzyme is inducible after exposure to polycyclic aromatic hydrocarbons. The inductive potential of CYP1A2 was estimated by incubating benzo(a)pyrene with monocytes and NeoHep separately. CYP1A2 induction was then measured by Luciferin‐1A2 substrate. The results are shown in Figure 4A. The induction of the CYP1A2 enzyme was not observed in monocytes, whereas a fourfold increase was observed for NeoHep's CYP1A2.

#### Detoxification of 7‐Pentoxy Resorufin by NeoHep Using P450 Enzymes

The activity of the CYP2B6 enzyme was measured in terms of the amount of resorufin produced per microgram of total cell lysate protein. The NeoHep generated from healthy monocytes and HNP monocytes was able to metabolize 7‐pentoxy resorufin to resorufin. No such metabolic activity was shown by the monocytes. Remarkably, the activity of HNP NeoHep and healthy NeoHep matched the activity of the hepatocarcinoma cell line HepG2 (Fig. 4B)

#### Relative Quantification of CYP3A4 mRNA Expression

The expression of CYP3A4 was estimated by qRT‐PCR, and the results are shown in Figure 4C and 4D. The NeoHep derived from both; the HNP and healthy monocytes had a higher expression of CYP3A4 in comparison with that of monocytes and RM.

#### Activity of CYP3A4 in Microsomes

The activity of CYP3A4 in the microsomes isolated from the NeoHep was estimated and compared with the activity of human liver microsomes. The microsomes from five samples, from healthy and HNP NeoHep, were pooled, and equal amounts of human liver microsomes were used to evaluate the CYP3A4 activity. The luminescence of the product formed after CYP3A4 substrate (PFBE) metabolism was determined. It was observed that NeoHep microsomes showed CYP3A4 activity, although it was around 10% in comparison with the human liver microsomes (Fig. 4E).

#### Secretion of Human Albumin and Clotting Factor VII Activity in the Culture Supernatant by NeoHep

The presence of secretory albumin and clotting factor VII activity in the culture supernatant of NeoHep and RM was estimated. It was observed that both the NeoHep derived from healthy and HNP monocytes secrete human albumin in their culture supernatant. Albumin was not observed in the culture supernatant of RM (Fig. 4F). Similarly, clotting factor VII activity was detected in the culture supernatant of NeoHep (0.0150 ± 0.007 IU/ml, *n* = 5), and there was no such activity in the culture supernatant of RM.

### Global Transcriptome Analysis for Monocytes, RM, NeoHep, and Human Hepatocytes

The differentially expressed genes and associated processes in monocytes, RM, and NeoHep were examined. The Solexa output readings had a Phred quality score >20, and the summary of filtered read‐outs mapped on human genome hg19 are given in the supplemental online data. The minimum and maximum mapping percentages were 87.89 and 97.55, respectively.

**Figure 5:**
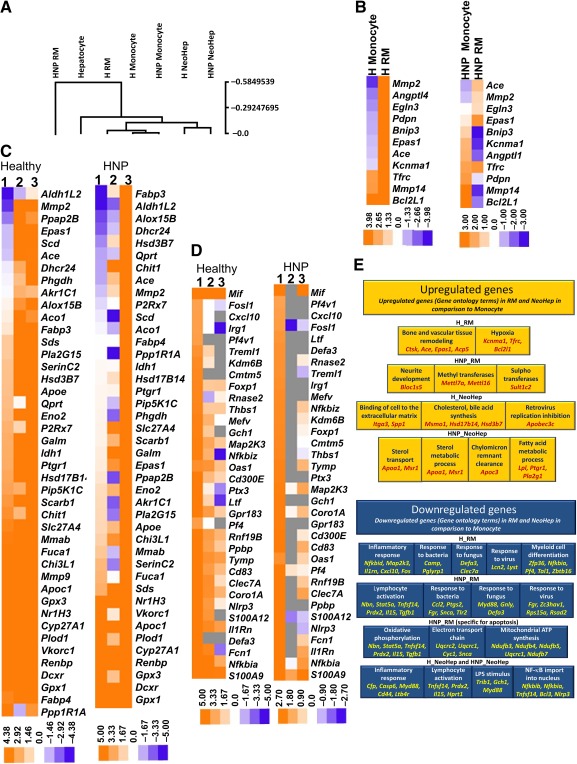
Differential expression of genes during reprogramming and differentiation of monocytes. **(A):** Dendrogram of hierarchical clustering using Spearman's rank correlation matrix. The heat maps of genes involved in hypoxia, expressed in monocyte and reprogrammed monocytes (RM), are shown **(B)**; cholesterol and bile acid metabolism expressed in monocyte, RM, and hepatocyte‐like cells (NeoHep) are also shown **(C)**. The expressions of downregulated genes related to inflammation and immune response in RM and NeoHep in comparison with monocytes are shown **(D)**. **(C, D):** Columns 1, 2, and 3 indicate monocyte, RM, and NeoHep, respectively, and gray squares indicate that no transcript was present. **(E):** Summary of differential expression of genes and corresponding biological processes. Abbreviations: ATP, adenosine triphosphate; H, hepatitis; HNP, hepatitis B surface antigen nucleic acid test positive; LPS, lipopolysaccharide; NeoHep, hepatocyte‐like cells; NF, nuclear factor; RM, reprogrammed monocytes.

The fragments per kilobase million values of the genes for each sample were converted to logarithmic base 10 value. Using these values, we performed a hierarchical clustering of the seven samples on the basis of their gene expression by using Spearman's rank correlation matrix; the dendrogram obtained is shown in Figure 5A.

In this dendrogram the H_monocytes and HNP_monocytes were in the same cluster. Similarly, H_NeoHep and HNP_NeoHep were also in the same cluster. However, the H_RM and HNP_RM were in different clusters. The node height for the NeoHep cluster (0.068) was closer to the node height of hepatocyte (0.187) in comparison with that of H_RM (0.033) and the monocyte cluster (0.0). The node height of HNP_RM (0.584) was distant from the remaining six samples.

This hierarchical clustering must have happened because of the up‐ and downregulation of unique genes responsible for maintenance of crucial biological and molecular functions in RM and NeoHep. Upregulation of these genes with respect to a few typical biological processes such as cholesterol, bile acid metabolism, and hypoxia was examined by generating corresponding heat maps.

The heat map (Fig. 5B) displays the differential expression of hypoxia‐related genes during the reprogramming of healthy and HNP monocytes. There were many common upregulated hypoxia‐related genes in H_RM and HNP_RM. Similarly, there were a large number of common upregulated metabolism‐related genes (Fig. 5C) in H_NeoHep and HNP_NeoHep, but the upregulated genes in RM were not common, possibly because the expression of these genes in the corresponding monocytes was different. A noteworthy downregulation in inflammation and immune defense‐related gene expression was observed in RM and NeoHep, as is shown in the heat maps (Fig. 5D).

A summarized overview of differential expression of genes is given in Figure 5E, which has input both from these heat maps and from the gene ontology record given in the supplemental online data.

#### Early Markers of Induced Pluripotent Stem Cells

**Figure 6:**
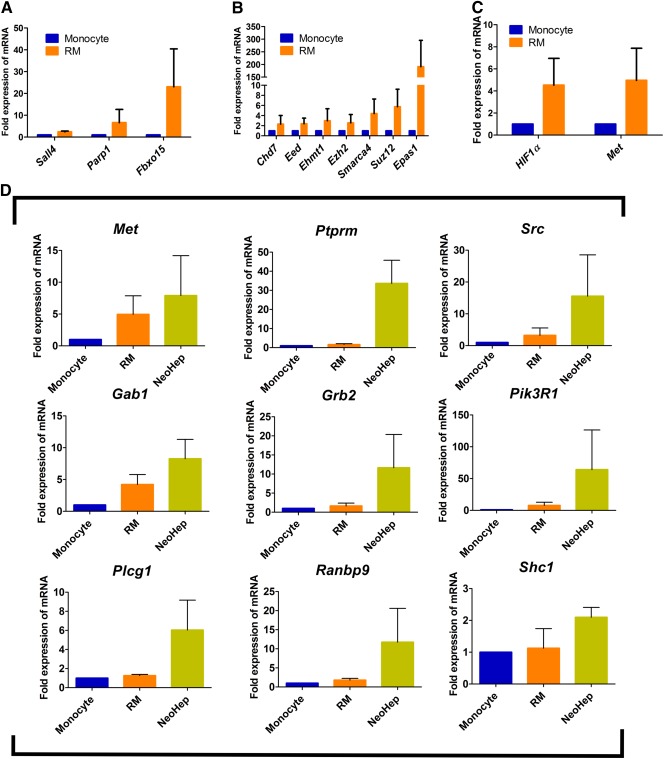
Chromatin remodeling, hypoxia, and c‐MET signaling during reprogramming and differentiation. Some of the typical markers (*Sall4, Parp1, Fbxo15*) expressed in induced pluripotent cells are also upregulated in reprogrammed monocytes (RM) in comparison with monocytes **(A)**. The upregulation of chromosome remodeling complex (*Chd7, Eed, Ehmt1, Ezh2, Smarca4, Suz12, Epas1*) **(B)** and *Hif1α* and *Met* **(C)**. The relative quantification of *Met* and genes involved in c‐MET signaling pathways (*Ptprm, Src, Gab1, Grb2, Pik3r1, Plcg1, Ranbp9, Shc1*) of monocytes, RM, and hepatocyte‐like cells are summarized **(D)**. For all the plots, *n* = 3, and error bar denotes standard error of the mean. Abbreviations: NeoHep, hepatocyte‐like cells; RM, reprogrammed monocytes.

It was also observed that RM shows expression of *Sall4*, *Parp1*, and *Fbxo15*, which are well‐known early markers of induced pluripotent stem cells (iPSCs) 10, thus correlating the RM properties to those of iPSCs (Fig. 6A).

#### Chromatin Remodeling

Epigenetic modulation due to the upregulation of chromosome remodeling complex plays a critical role in cellular reprogramming. Furthermore, *Hif1α* and *Hif2α* (*Epas1*) play a key role in reprogramming of somatic cells to induce pluripotent stem cells 11.

The regulation of chromosome remodeling complex in RM was compared with monocytes by performing qRT‐PCR with the same RNA samples with which the RNASeq was done. Figure 6B shows that *Chd7*, *Eed*, *Ehmt1*, *Ezh2*, *Smarca4*, and *Suz12*, which are the key genes of chromosome remodeling, were upregulated in RM along with *Epas1*.

#### Hypoxia and c‐MET Signaling

It is an established fact that *Hif1α* induces the expression of *Met*, which is a receptor of hepatocyte growth factor (HGF) 12. The expression of both *Hif1α* and *Met* was higher in RM than in monocytes (Fig. 6C).

The upregulation of *Met* in RM unfolded a unique scenario, because the media used for the differentiation of RM to NeoHep were supplemented with HGF. The first graph of Figure 6D shows that the expression of *Met* was further increased in NeoHep. The genes of the c‐MET signaling pathway—such as *Ptprm, Src, Plcg1, Ranbp9, Pik3r1, Grb2, Shc1*, and *Gab1—*were more highly expressed in NeoHep than in RM, possibly because of the presence of HGF in the culture medium, as is shown in Figure 6D.

### Transplantation of NeoHep in One‐Third Partially Hepatectomized NOD SCID Mice

To explore the potential of in vitro generated NeoHep for cell‐based therapy, we transplanted the NeoHep by splenic infusion in a partially hepatectomized NOD SCID mouse. It was found that not all the transplanted cells ended up in regenerated liver lobes, and DNA specific to human TNF‐α was detected in the bone morrow of the recipient mouse.

#### Detection of Human Albumin and Human Connexin 32 in the Liver Section of NOD SCID Mouse by Immunohistochemistry

**Figure 7:**
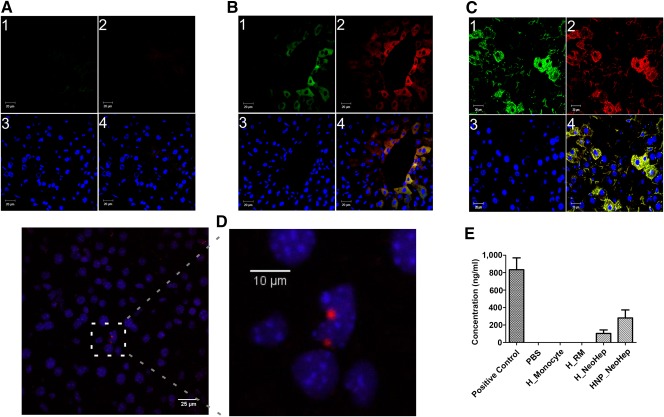
Transplanted hepatocyte‐like cells (NeoHep) in an hepatectomized NOD.CB17‐*Prkdc^scid^*/J (NOD SCID) mouse. **(A):** Liver tissue sections of a partially hepatectomized NOD SCID mouse without cell transplantation (only phosphate‐buffered saline [PBS]). **(B, C):** Partially hepatectomized NOD SCID mouse liver sections in which healthy hepatocyte‐like cells (NeoHep) and hepatitis B surface antigen nucleic acid test‐positive (HNP) NeoHep were transplanted, respectively. Images were taken using Plan Apochromat 63×/1.4 oil objective, and the numerals indicate the following: (1) human anti‐connexin 32 (Alexa Fluor 488); (2) human anti‐albumin (Alexa Fluor 594); (3) nucleus 4′,6‐diamidino‐2‐phenylindole; and (4) merged image. Scale bar denotes 20 µm. **(D):** Binding of the fluorescence in situ hybridization probe specific to human chromosome 9 centromere in the liver section of a hepatectomized NOD SCID mouse in which HNP NeoHep were transplanted. Scale bar is 25 µm and 10 µm for normal and zoomed panels, respectively. **(E):** Concentration of human albumin secreted by transplanted NeoHep in the blood stream of a recipient NOD SCID mouse when no cells (only PBS), healthy monocytes, healthy reprogrammed monocytes, healthy NeoHep, and HNP NeoHep were transplanted (*n* = 3). Error bars represent standard errors of the mean. The positive control (PC) is the serum of a NOD SCID mouse in which 1 mg of human albumin was injected through the tail vein and blood was then collected after 6 hours. Abbreviations: FISH, fluorescence in situ hybridization; H, hepatitis; HNP, hepatitis B surface antigen nucleic acid test positive; NeoHep, hepatocyte‐like cells; PBS, phosphate‐buffered saline; RM, reprogrammed monocytes.

The functional activity of transplanted NeoHep in the hepatectomized NOD SCID mouse was confirmed by the presence of human albumin in its liver section. The presence of human connexin 32 established the engraftment of transplanted NeoHep in the mouse liver cortex. The liver sections are shown in Figure 7A–7C.

#### Fluorescence In Situ Hybridization to Detect Nucleus of Engrafted Human Cells in the Liver Tissue Sections of NOD SCID Mouse

Figure 7D shows the binding of DNA probe specific to human chromosome 9 centromeric regions in the nucleus of the transplanted NeoHep, engrafted in the liver cortex of a hepatectomized NOD SCID mouse.

#### Detection of Human Albumin and Clotting Factor VII Activity in the Serum of NOD SCID Mice

Functional metabolic activity of transplanted NeoHep, derived from both healthy monocytes and HNP monocytes, was confirmed by the presence of human albumin in the serum of the recipient mouse detected by ELISA (Fig. 7E). No human albumin was detected when the monocytes or the RM were transplanted.

Ten days’ posttransplantation of NeoHep in a partially hepatectomized mouse, we detected human clotting factor VII activity in the serum of the recipient mouse (0.087 ± 0.033 IU/ml, *n* = 4; typical range for plasma is 0.5–2.0 IU/ml). Interestingly, because monocytes are known to secrete clotting factor VII 13, 14, its activity was observed when monocytes were transplanted in a partially hepatectomized mouse, which is a confirmation. No human clotting factor VII activity was detected when RM were transplanted.

## Discussion

In this article we have demonstrated for the first time that the monocytes from HBsAg‐NAT‐positive peripheral blood (HNP) collected from chronic HBV patients can be differentiated to hepatocyte‐like cells (NeoHep). This article describes a step toward possible autologous cell‐based therapy for patients having hepatic insufficiency due to hepatitis B infection.

The binding of HBsAg specifically to the CD14 molecule of the monocytes is a well‐known phenomenon 15, and the HBsAg is also known to inhibit secretion of inflammatory cytokines by monocytes 16. Also, the HBsAg proviral particles of hepatitis B virus, which are released in the blood stream of chronic hepatitis B patients, preferentially deposit in CD14+ monocytes 17. These two facts together render monocytes apoptotic 18, 19. We observed a similar result with a higher abundance of apoptotic monocytes and neutrophils in the HNP blood.

We found that before initiating the culture of PBMCs from HNP blood, it was essential to deplete excess neutrophils. In order to minimize the effect of serine proteases present in serum, which are known to induce apoptosis 20, we used a limiting serum concentration of 0.5% to culture‐sorted monocytes to generate RM.

The expression of hepatocyte‐specific markers in NeoHep was evidence of the differentiation of nonparenchymal cells to hepatic parenchymal cells. Connexin 32, which is an important gap‐junction protein specific to hepatocytes 21, was expressed by NeoHep derived from healthy and HNP blood. HNF4α expression was observed in the nucleus of NeoHep but not in the corresponding monocyte and RM. Albumin was detected in the cytoplasm of NeoHep. Most important, the NeoHep derived from both healthy and HNP blood showed similar expression of connexin 32, HNF4α, and albumin.

The functional activity of NeoHep was confirmed by examining the activity of cytochrome P450 enzyme. The metabolic potential of NeoHep to reduce 7‐pentoxy resorufin to resorufin confirmed the activity of the CYP2B6 enzyme. In hepatocytes and HepG2 22, the induction of enzyme CYP1A2 activity by polyaromatic compounds is well known. Similarly, the activity of CYP1A2 was induced when NeoHep was treated with benzo(a)pyrene. The activity of the CYP3A4 enzyme was confirmed in the microsomes isolated from the uninduced NeoHep. Thus the induction of CYP1A2 activity and the presence of CYP2B6 and CYP3A4 activity in NeoHep make them a very promising candidate for screening new drugs. The secretion of albumin is one of the crucial functional attributes for hepatocytes, which was observed in the culture supernatant of NeoHep. Thus apart from detoxification, NeoHep have physiological activity similar to that of primary human hepatocytes.

To mimic a clinical situation in which NeoHep could be used as a cell‐based therapy to treat compromised liver, we transplanted NeoHep to a partially hepatectomized NOD SCID mouse by splenic infusion. The NeoHep were engrafted in the liver of a recipient mouse, and they expressed hepatocyte‐specific markers and functions in vivo. The presence of human connexin 32 in the liver of the recipient mouse demonstrated the engrafting ability of NeoHep. The presence of cytosolic human “proalbumin”—human albumin and human clotting factor VII activity in the serum of recipient NOD SCID mouse 10 days’ posttransplantation— confirms the “stable” and successful differentiation of monocyte to NeoHep in vitro and their engraftment in the host.

The RNA sequencing data of monocytes, RM, and NeoHep revealed the genes that were differentially expressed in RM and NeoHep. The claim of differentiation of monocyte to NeoHep was further emphasized by the hierarchical clustering of gene expression profiles, which indicated that H_NeoHep and HNP_NeoHep had similar gene expression profiles. This clustering further proved that the expression profiles of NeoHep were different from those of monocyte and RM but more similar to those of hepatocytes. Additionally, the genes responsible for the immunological defense response to bacteria, fungi, T‐cell activation, and so on, were downregulated in the RM and NeoHep, and several genes associated with metabolism were upregulated in NeoHep.

The RNA sequencing data also uncovered the process of reprogramming and differentiation of monocytes to NeoHep. During the reprogramming step—in the presence of IL3, MCSF, and 2‐ME—the genes influenced by hypoxia were upregulated, leading to chromosome remodeling 23. Interestingly, the upregulated expression of hypoxia‐related genes in RM induces MET expression. Similar upregulation of c‐MET has been reported earlier 12. During the differentiation of RM to NeoHep, the c‐MET signaling cascade was further activated, due to the presence of HGF 24, thereby leading to the expression of hepatocyte‐specific genes in NeoHep 25, 26.

One of the most significant results of this study is that there was no hepatitis B virus gene in the NeoHep differentiated from monocytes of HNP blood. The NeoHep generated from HNP blood were similar to NeoHep obtained from healthy blood. Besides, the NeoHep display key phenotypic characteristics, as found in primary hepatocytes; their P450 enzyme were metabolically active, and they were integrated within the liver and remained metabolically functional. These unique features open up possibilities for autologous NeoHep transplantation in HBV‐infected patients, although many novel interventions including antiviral therapy are essential before this approach could be translated further for clinical therapeutics.

## Author Contributions

J.B. and B.D.: conception and design, collection and/or assembly of data, manuscript writing; D.S., S.I., and P. Nagpal: collection and/or assembly of data; P.S. and A. Mishra: collection and/or assembly of data, manuscript writing; K.J.: collection and/or assembly of data, manuscript writing; V.S., P. Nagaragan, P.K., and A. Mukhopadhyay: supervision; P.U.: conception and design, collection and/or assembly of data, manuscript writing, supervision.

## Disclosure of Potential Conflicts of Interest

The authors indicated no potential conflicts of interest.

## Supporting information

Supporting Information
