# Supplementary material for: Autologous NeoHep Derived from Chronic Hepatitis B Virus Patients’ Blood Monocytes by Upregulation of c‐MET Signaling
Source: Stem Cells Transl Med. 2016 Jul 28;6(1):174–86. doi: 10.5966/sctm.2015-0308 (PMC5442753; doi:10.5966/sctm.2015-0308)
Supplement: Supplementary file 1 — Supporting Information [file SCT3-6-174-s001.pdf]

Supplementary Materials

Autologous NeoHep derived from chronic HBV patient's blood monocytes by upregulation of cMET signalling

Jashdeep Bhattacharjee[1], Barun Das[1], Disha Sharma[2], Preeti Sahay[1], Kshama Jain[1], Alaknanda Mishra[1], Srikanth Iyer[1], Puja Nagpal[1], Vinod Scaria[2], Perumal Nagarajan[1], Prakash Khanduri[3], Asok Mukhopadhyay[1], Pramod Upadhyay[1]

1. National Institute of Immunology, Aruna Asaf Ali Marg, New Delhi 110067
2. Institute of Genomics and Integrative Biology, South Campus, Mathura Road, New Delhi 110025
3. St. Stephen's Hospital, Tis Hazari, Delhi 110055.

TABLE OF CONTENTS		
**Serial Number**	**Name**	**Page Number**
1	Materials and Methods	**4**
2	Validation plot of RNASeq data using qPCR	**12**
3	Quantitation of reads in each sample having phred score > 20	**13**
4	Mapping percentage of reads on human genome, hg19, for each sample using TOPHAT	**14**
5	Hepatectomy and Cell Infusion Procedure	**15**
6	Sequences of primers used in this study	**16**
7	Biological Process (BP_FAT) associated with genes having decreased expression in **H_RM** with respect to H_Monocyte	**17**
8	Biological Process (BP_FAT) associated with genes having decreased expression in **H_NeoHep** with respect to H_Monocyte	**21**
9	Biological Process (BP_FAT) associated with genes having decreased expression in **HNP_RM** with respect to HNP_Monocyte	**43**
10	Biological Process (BP_FAT) associated with genes having decreased expression in **HNP_NeoHep** with respect to HNP_Monocyte	**163**
11	Biological Process (BP_FAT) associated with genes having increased expression in **H_RM** with respect to H_Monocyte	**179**
12	Biological Process (BP_FAT) associated with genes having increased expression in **H_NeoHep** with respect to H_Monocyte	**183**
13	Biological Process (BP_FAT) associated with genes having increased expression in **HNP_NeoHep** with respect to H_Monocyte	**185**
14	Cellular Component (CC_FAT) associated with genes having decreased expression in **H_RM** with respect to H_Monocyte	**191**

15	Cellular Component (CC_FAT) associated with genes having decreased expression in **H_NeoHep** with respect to H_Monocyte	**192**
16	Cellular Component (CC_FAT) associated with genes having decreased expression in **HNP_RM** with respect to HNP_Monocyte	**208**
17	Cellular Component (CC_FAT) associated with genes having decreased expression in **HNP_NeoHep** with respect to HNP_Monocyte	**262**
18	Cellular Component (CC_FAT) associated with genes having increased expression in **H_RM** with respect to H_Monocyte	**270**
19	Cellular Component (CC_FAT) associated with genes having increased expression in **H_NeoHep** with respect to H_Monocyte	**271**
20	Cellular Component (CC_FAT) associated with genes having increased expression in **HNP_RM** with respect to HNP_Monocyte	**273**
21	Cellular Component (CC_FAT) associated with genes having increased expression in **HNP_NeoHep** with respect to HNP_Monocyte	**274**
22	Molecular Function (MF_FAT) associated with genes having decreased expression in **H_RM** with respect to H_Monocyte	**277**
23	Molecular Function (MF_FAT) associated with genes having decreased expression in **H_NeoHep** with respect to H_Monocyte	**281**
24	Molecular Function (MF_FAT) associated with genes having decreased expression in **HNP_RM** with respect to HNP_Monocyte	**290**
25	Molecular Function (MF_FAT) associated with genes having decreased expression in **HNP_NeoHep** with respect to HNP_Monocyte	**325**
26	Molecular Function (MF_FAT) associated with genes having increased expression in **H_RM** with respect to H_Monocyte	**330**
27	Molecular Function (MF_FAT) associated with genes having increased expression in **H_NeoHep** with respect to H_Monocyte	**331**
28	Molecular Function (MF_FAT) associated with genes having increased expression in **HNP_NeoHep** with respect to HNP_Monocyte	**332**

Materials and Methods

Ethics statement

The use of healthy human peripheral blood and buffy coat in the present study was approved by the institutional human ethics committee of the National Institute of Immunology. The use of HBsAg-NAT positive peripheral blood was approved by the institutional human ethics committee and the institutional biosafety committee of the National Institute of Immunology. The experiment on NOD SCID mice was approved by the institutional animal ethics committee of National Institute of Immunology.

Isolation of peripheral blood mono-nuclear cells (PBMCs)

Anonymous samples of healthy and HBsAg-NAT positive human peripheral (HNP) blood were collected from adults (18-55 years age) and PBMCs were isolated by density mediated centrifugation over Ficoll-Paque PREMIUM (GE Healthcare, Little Chalfont, United Kingdom).The cells were collected from the buffy layer followed by washing in Dulbecco's phosphate buffer saline (DPBS) (Himedia, Mumbai, MH, India) and resuspended in phenol red free IMDM (Gibco, Waltham, MA, USA). The cells were counted using hemocytometer (Paul Marienfeld GmbH & Co. KG,Lauda-Königshofen, Germany).

Isolation of monocytes from PBMCs (sorting strategy)

Granulocytes, lymphocytes, NK cells and other non-monocytic cells were removed from the enumerated PBMCs by two consecutive negative magnetic assisted cell sorting (MACS) procedure. To remove granulocytes, cells were incubated with CD66abce- biotin antibody followed by incubation with anti-biotin microbeads (Miltenyi Biotec, Bergisch Gladbach, Germany). The cells were washed, suspended in MACS buffer and fractionated into negative and positive populations using pre-wet LS magnetic column. The granulocyte depleted cell population was then used to isolate monocytes using Monocyte Isolation Kit II (MiltenyiBiotec, Bergisch Gladbach, Germany) as instructed in the product technical datasheet.

Cell culture

The monocytes were seeded on Matrigel^{TM} (Corning Inc.,Corning, NY, USA) coated plate at a density of 0.5 million cells per cm^2.The differentiation to NeoHep is achieved by using a two stage differentiation protocol. In the first stage, monocytes were differentiated to "reprogrammed monocytes" (RM) in presence of basal media Iscove's Modified Dulbecco's Media (IMDM) (Gibco,Waltham, MA,USA) supplemented with interleukin-3 (4.0ng/ml) (ProSpec-TanyTechnoGene Ltd., Rehovot, Israel), macrophage colony stimulating factor (5.0ng/ml) (ProSpec-TanyTechnoGene Ltd.,Rehovot, Israel), β-mercaptoethanol (140.0µM)(Sigma, St.Louis, MO, USA) and 0.5% human embryonic stem cell grade fetal bovine serum(eFBS)(Biological Industries Israel Beit-HaemekLtd., KibbutzBeit-Haemek, Israel). After day 6, RM was differentiated to NeoHep for a tenure of 15 days in presence of IMDM supplemented with epithelial growth factor (10.0ng/ml) (ProSpec-TanyTechnoGene Ltd.,Rehovot, Israel), hepatocyte growth factor (20.0ng/ml) (ProSpec-TanyTechnoGeneLtd.Prospec, Rehovot, Israel), fibroblast growth factor-4 (3.0ng/ml) (ProSpec-TanyTechnoGeneLtd.Prospec, Rehovot, Israel) and linoleic acid (5.0µg/ml) (Sigma, St. Louis, MO, USA). A sequential increase in the concentration of eFBS was maintained during the culture tenure starting with 2.0% from day 6 to day 8, 5.0% from day 8 to day 13 and 10.0% from day 13 to day 21.

Anti-bacterial and anti-mycotic solution (1X) (Himedia, Mumbai, MH, India) was added in the media to avoid contamination. The cell culture plates were incubated in CO_2 incubator maintained at 37^0C/ 5% CO_2/85%-90% RH.

Abundance of monocyte and granulocyte in healthy and HNP blood derived PBMCs

1 million PBMCs isolated from healthy and HNP blood was incubated with PE-conjugated CD14 (monocyte marker) and FITC-conjugated CD66b (neutrophil marker) on ice for 30 minutes. The cells were washed with PBS and were analysed in flow cytometer CyFlow (SysmexPartec GmbH, Görlitz, Germany). The data was analysed using FlowJo software (Tree Star, Ashland, OR, USA). Abundance of CD14 positive and CD66b positive cells were represented in dot plots.

Annexin A5 staining

The annexin A5 staining was performed using FITC Annexin A5 apoptosis detection kit I (BD Biosciences, Franklin Lakes, NJ, USA) according to manufacturer's instruction. The cells were resuspended in binding buffer (1X) followed by incubation with FITC-annexin A5 and propidium iodide (PI) for 15 minutes at room temperature. Individual single colour control was made according to manufacturer's instruction. Stained cells were analysed using flow cytometer CyFlow. The data was analysed using FlowJo software. Abundance of annexin A5 positive and PI positive cells were represented in dot plots

Extraction of genomic DNA and End point polymerase chain reaction

For extraction of genomic DNA from cells and viral DNA from plasma, MasterPure™ Complete DNA and RNA Purification Kit (Epicentre, Madison, WI, USA) was used according to manufacturer extraction. The DNA was used as template for the end point PCR using Phusion® High-Fidelity PCR Master Mix (NEB, Ipswich, MA, USA). The amplified amplicons were viewed in 2.5% agarose gel. Low range DNA marker (25-500 base pair) (Bio Basic Inc., Markham, ON, Canada) was used in the ladder lane. The sequences of the primers are provided in supplementary data sheet.

Viral load determination

To obtain the positive control of known concentration, a region of conserved HBV genome sequence, specific to hepatitis B surface antigen (HBSAg) was synthesized. To prepare the standard curve of HBV DNA, the synthesized oligonucleotides was serially diluted from 6.02E10 copies/µl to 6.02E02 copies/ µl and qRTPCR was carried out using SYBR green detection system with primers designed in a way that are specific to the hepatitis B surface antigen and flanking the synthesized 150 bases oligonucleotide sequences. The viral load of 24 anonymous HNP sample was subsequently determined through qRTPCR with the same primer pair from the isolated serum/plasma DNA. The respective Ct value was plotted in the standard curve and corresponding load was calculated from the graph. The values obtained in copies/ml was converted in IU/ml according to standard convention. The sequence of the positive control and the primers is given on page 16.

Immunocytochemistry

Cells were washed twice in PBS and fixed in 4% formaldehyde solution for 15 minutes at 4^oC. Cells were permeabilized in 0.2% Triton X-100 (Amresco, Solon, OH, USA) and blocked by 1% bovine serum albumin (Sigma, St.Louis, MO, USA) followed by incubation in the following primary antibodies in humidified chamber at 37^0C for 1 hour: anti- human connexin32 (Abcam, Cambridge,

England, UK), anti-human albumin (Pierce Biotechnology, RockFord, IL, USA), anti-human HNF4α (Santa Cruz Biotechnology, Dallas, TX, USA. Secondary Antibody used are: Donkey anti-Goat IgG (H+L) Alexa Fluor® 594 and Donkey anti-Sheep IgG (H+L) Alexa Fluor® 488 (Molecular Probes, Pittsburgh, PA, USA). The nucleus was counterstain with 4', 6-diamidino-2-phenylindole (DAPI) (Himedia, Mumbai, MH,India). The slides were mounted with Vectashield Mounting Media (VectorLab, Burlingame, CA, USA) and viewed using confocal laser scanning microscope, LSM 510 META (Zeiss, Oberkochen, Germany) under Plan-Apochromat 63X/1.4 oil objective lens.

Activity of CYP2B6 by PROD assay

The P450 mediated detoxification was examined by PROD assay. Briefly, cells were incubated in media containing 7-Pentoxy resorufin at 37^0C in 5% CO_2 for 24 hours. The cell culture supernatant was collected and analyzed in spectrofluorimeter (Shimadzu, Kyoto, Japan) to detect the presence of resorufin in the supernatant. To detect resorufin, the sample was excited at 571nm and fluorescence emission at 585nm was recorded. The concentration of produced resorufin was detected with the reference of resorufin standard. The total cell lysate was isolated by SDS-RIPA buffer and the concentration of protein was measured by Micro BCA protein assay kit (Pierce Biotechnology, RockFord, IL, USA).

Induction of CYP1A2 in Monocytes, NeoHep and HepG2

The Cells were plated in a 96 well culture plate and incubated for 48 hours with media containing IMDM, 1X ITS (Sigma Aldrich, St. Louis, MO, USA) and 5µM benzo(a)pyrene (Sigma Aldrich, St. Louis, MO, USA) at 37^0C/5%CO_2. The metabolic activity was assessed by the measurement of luciferase activity using the P450-Glo CYP1A2 assay kit (Promega, Madison, WI) according to the manufactures guidance.

Detection of CYP3A4 substrate metabolism potential

Microsomes from NeoHep were isolated as described previously(1). Briefly, the cells were homogenised in homogenisation buffer (Tris-HCl -10mM pH7.4; sucrose 250mM; PMSF 0.1mM, glycerol) and the lysate was centrifuged at 5000*g* for 15 minutes at 4^OC. The supernatant was centrifuged at 12000*g* for 20 minutes at 4^OC. After centrifugation the supernatant was collected and centrifuged at 100,000g for 1 hour at 4^OC. The pellet containing microsomes was resuspended in homogenisation buffer.

To detect the metabolic potential of CYP3A4 substrate for the microsome of the given cell type, P450-Glo™ CYP3A4-PFBE Induction/Inhibition Assay (Promega Corporation,Madison, WI, USA) was performed according to manufacturer's protocol. For the assay, microsomes from 5 different samples were collected and pooled. Briefly, 50µM of CYP3A4-PFBE substrate was incubated with 20µg of pooled microsome sample in a reaction mixture containing KPO_4 buffer (100mM) and NADPH regeneration system (1X) at 37^OC for 30 minutes. After incubation, equal volume of Luciferin detection reagent was added to the reaction mixture and luminescence was recorded after 10 minutes using Luminoskan Ascent Microplate Luminometer (Thermo Scientific, Pittsburgh, PA, USA). Human liver microsomes derived from 50 donours was used as positive control (Life technologies,Waltham, MA, USA).

Detection of human albumin by ELISA

Human albumin ELISA was performed to quantify the secretion of human albumin by RM and NeoHeps in the cell culture supernatant with respect to that of HepG2. The presence of human albumin in the serum of human cell transplanted hepatectomised NOD SCID mice was determined using the serum collected on 10th day post transplantation. The culture supernatant and serum was collected and stored at -20^{0}C. To perform ELISA, the supernatants were thawed and 1:10 dilution of each supernatant was made in PBS. The NOD SCID mouse serum was diluted to 1:10 in PBS. Human Albumin ELISA Core Kit (KOMA Biotech, Yeongdeungpo-gu, Seoul, South Korea) was used to quantify human albumin according to manufacturer's instruction.

Human Clotting Factor VII activity

We checked for clotting factor VII activity in both cell culture supernatant (H_RM, HNP_RM, H_NeoHep & HNP_ NeoHep) as well as NOD SCID Mice serum (control, monocyte transplanted, RM transplanted, H_NeoHep transplanted & HNP_NeoHep transplanted). To estimate the Human factor VII activity in cell culture supernatant, culture medium was augmented with 100 ng/ml vitamin K. Undiluted culture supernatants were directly used as sample whereas serum of the mice were diluted 5 time with Phosphate buffer Saline. Factor VII activity was determined by Human factor VII Chromogenic activity kit (Assaypro LLC, MO. USA) according to the manufacture's protocol. Briefly, 100 µl of culture supernatant/ diluted serum was incubated in the pre-coated wells for 2 hours at room temperature. After, 5 time wash with 1X wash buffer 80 µl assay mix containing recombinant tissue factor and human factor X was added per well. After incubation for 30 min at 37°C, 20 µl of factor Xa Substrate was added and zero minute background absorbance was recorded at 405 nm. The plate was then kept at 37°C and absorbance was recorded at 405 nm at different time interval. Human Serum was taken as a positive control. The activity of factor VII in samples was determined from the standard curve that was generated according to the supplied protocol.

Extraction and storage of RNA by spin column method

The total RNA from sorted monocytes, RM and NeoHep was isolated using Fisher BioReagents™ SurePrep™ RNA/DNA/Protein Purification Kit (Thermo Scientific, Pittsburgh, PA, USA) according to manufacturer's instructions. The RNA was then collected and stored in RNA stable tube (Biomatrica Inc., San Diego, CA, USA).

Library preparation and Sequencing of poly (A) tailed RNA

Monocytes sorted from healthy human peripheral blood (n=5) and HBsAg-NAT human peripheral blood (n=6) were differentiated to NeoHep. The differentiation of monocytes to RM and then to NeoHep was analysed on the basis of transcripts expressed at each of these three stages by sequencing of poly (A) tailed RNA. The integrity of the extracted RNA was analysed by Agilent 2100 Bioanalyzer (Agilent Technologies, Santa Clara, CA, USA). For sequencing, RNA extracted from each of these stages was pooled separately for healthy blood derived cells and HBsAg-NAT positive blood derived cells. In total there were 7 samples for RNA sequencing. They were human hepatocyte RNA (Hepatocyte), healthy blood derived- monocytes (H_Monocyte), RM (H_RM), NeoHep (H_NeoHep), HBsAg-NAT positive blood derived-monocytes (HBV_Monocytes), RM (HBV_RM), NeoHep (HBV_NeoHep). The library preparation for RNA sequencing was performed using TruSeq RNA Sample Prep Kit v2 (Illumina Inc., San Diego, CA, USA) according to the protocol outlined in "TruSeq RNA Sample Preparation Guide". Briefly, the total RNA in RNA Stable tube was recovered by adding

sterile water and hydrated for 20 minutes at RT until the pellet was completely dissolved. 50µl (~1.5-3.5µg) of total RNA was subjected to Poly A purification of RNA. Purified RNA was reverse transcribed with Superscript III Reverse transcriptase by priming with Random Hexamers. Second strand cDNA was synthesized in the presence of DNA polymerase I and RNaseH. The cDNA was cleaned up using Agencourt Ampure XP SPRI beads (Beckman Coulter, Brea, CA, USA). Illumina Adapters were ligated to the cDNA molecules after end repair and addition of A base. SPRI cleanup was performed after ligation. The library was amplified using 13 cycles of PCR for enrichment of adapter ligated fragments. The prepared library was quantified using Nanodrop and validated for quality by running an aliquot on High Sensitivity Bioanalyzer Chip (Agilent Technologies, Santa Clara, CA, USA).TruSeq Cluster Kit v3 (cBot - HS) (Illumina Inc., San Diego, CA, USA) with TruSeq v3 Flow Cell (Illumina Inc., San Diego, CA, USA) was used to sequence the transcriptome library on Hiseq 2000 platform (Illumina Inc., San Diego, CA, USA).

Bioinformatic analysis for RNASeq datasets

The reads ranging from 43 million to 168 million per dataset were prepared for alignment over full reference hg19 human genome. Fastq reads were quality checked using Fastqc and filtered using SolexaQA[(2)] with phred cut-off 20. The Human reference genome was indexed using Bowtie2(3).TopHat(4) version 2.0.6 was used for alignment. The reads were aligned over reference index individually using TopHat2 to call for alternate splice transcripts using default options. Cufflinks(4) was used to assemble transcripts and estimate the abundance and expression of transcripts for 7 samples along with reference annotation gencodev20 that was downloaded. Cuffmerge was used to merge the output (transcripts.gtf) of Cufflinks for individual samples. Cuffdiff(5), was used to evaluate the differential expression of transcripts. FPKM (fragment per kilobase of exon per million base pair) values for isoforms transcripts were log 2 normalized by adding 2 to the FPKM values to avoid negative data.

To determine the upregulated genes in RM and NeoHep with respect to monocytes, the genes expressed in RM and NeoHep having FPKM value greater than or equal to 10 were selected. The fold increase of these selected genes of RM and NeoHep was determined in comparison to the monocytes. The genes which have more than 10 fold increased expression in RM and NeoHep with respect to monocytes were selected for GOTERM annotation using DAVID. The genes which got down regulated in their expression during differentiation from monocyte to RM and then NeoHep were also selected. For this, genes expressed in monocytes having FPKM value greater than or equal to 10 were selected. Then the ratios of expression value for those genes in RM and NeoHep in comparison to monocytes were determined. The genes having ratio less than 0.1 were selected for GOTERM annotation using DAVID. The analysis for healthy blood derived cells and HBsAg-NAT positive blood derived cells were performed separately. The heat map for gene expression was constructed using Cluster 3.0(6) and Java TreeView version 1.1.6r4.

Validation of RNA Sequencing data

The FPKM values of RNA sequence data sets were validated by qRT-PCR using the same RNA samples as used for pooling during library preparation. Each sample were analysed individually for the following markers: glutamate-ammonia ligase (*Glul*), RE1-silencing transcription factor (*Rest*), poly (ADP-ribose) glycohydrolase (*Parg*), CD14, CD36, *Myd88*, CD93 and CD33. The gene expression was normalized with housekeeping gene, glyceraldehyde-3-phosphate dehydrogenase (*Gapdh*). The

expression of genes in RM and NeoHep was calculated using the $\Delta\Delta C_t$ method with respect to monocytes. The qRT-PCR was performed using thermo cycler Realplex[4] (Eppendorf AG, Hamburg, Germany).

qRT-PCR of Differentially expressed genes in RM and NeoHep

qRT-PCR was performed to detect the differential expression of genes in RM and NeoHep in comparison to monocytes. *Hif1α, Met, Cdh7, Eed, Ehmt1, Ezh2, Smarca4, Suz12, Epas1, Parp1, Sall4, Fbxo15,Ptprm, Src, Plcg1, Ranbp9, Pik3r1, Grb2, Shc1* and *Gab1* genes were analyzed. cDNA was synthesized using Verso cDNA Kit (Thermo Scientific, Pittsburgh, PA, USA). For each 20µl qPCR, the concentration of forward and reverse primers was 200nM and 2µl of cDNA was added. qRTPCR of 40 cycles was performed using 2X MESA GREEN qPCR Master Mix Plus (Eurogentac, Seraing, Liège, Belgium) with enzyme activation at 95°C for 5 minutes, denaturation step at 95°C for 15 seconds, annealing temperature at 55°-65°C for 30 seconds and extension at 60°C for 30 seconds. The annealing temperature for each primer pair is provided in supplementary data. The differential expression of genes was calculated using $\Delta\Delta C_t$ method with respect to monocytes. The qRT-PCR was performed using thermo cycler Realplex[4].

Hepatectomy and transplantation of cells in NOD SCID mice

The sorted monocytes, RM and NeoHep were transplanted in NOD SCID mice by splenic infusion to investigate the engraftment and stability of the cells *in vivo*. The entire procedure was divided in two steps, excision of left lateral lobe of the liver of NOD SCID mice followed by transplantation of cells (either monocyte or RM or NeoHep in the same mice). At 10th day post transplantation, the mice were terminally bleeded to isolate serum and the liver and spleen were excised out. Both liver and spleen were weighed. The liver sections were stained for the presence of human albumin and human connexin 32. The serum from NOD SCID mice was used to detect human albumin by ELISA as mentioned earlier.

Homing locations of transplanted cells

To investigate the homing location and quantification of transplanted human cells in mouse tissue samples, RT-PCR was performed using human GAPDH and Human TNF-alpha specific primer and probe sets (7). Animals bearing transplanted cells after hepatectomy were euthanized 10 and 20 days post transplantation. Six organs; brain, heart, lung, liver, kidney and spleen including blood and bone marrow were isolated from these animals and their DNA and RNA were isolated. c-DNA samples were prepared from respective RNA samples to be used as templates against human GAPDH primers and DNA samples from different organs were used as templates against Human TNF-alpha primers.

For standard calibration RNA and DNA was isolated from two million human PBMCs and subsequent 10 fold dilutions were used for standard calibration. The probe and primer sequences used in the experiment is given on page 16. Reaction was set using Takyon ROX Probe MasterMix BLUE dTTP (Eurogentec) as per the product datasheet.

Cryosectioning of liver lobe

The excised mouse liver was cut and the liver pieces were fixed in 4% paraformaldehyde and then transferred to 30% sucrose solution for dehydration. The dehydrated tissue samples were then briefly immersed in freezing medium to remove sucrose. The tissue was frozen in tissue freezing

medium (Tissue-Tek®, Sakura Finetak, Torrance, CA, USA) at -22^{0}C for block making. Thereafter, 5µm tissue sections were obtained at OCT using Shandon cryotome (Thermo Electron Corporation, Waltham, MA, USA). The sections were collected on poly-L-Lysine coated glass slides and preserved at -22^{0}C.

Immunohistochemistry

Slides were washed twice in PBS and fixed in 4% formaldehyde. The sections were permeabilized in 0.2% triton X-100 and blocked by 1% bovine serum albumin followed by incubation in the following primary antibodies in humidified chamber at 37^{0}C for 1 hour: connexin32 (Abcam, Cambridge, England, UK), albumin (Pierce Biotechnology, RockFord, IL, USA). Secondary antibodies used were: Donkey anti-Sheep IgG (H+L) Alexa Fluor® 488 and Donkey anti-Goat IgG (H+L) Alexa Fluor® 594. The nucleus was counterstained with 4', 6-diamidino-2-phenylindole (DAPI) and the slides were mounted with Vectashield Mounting Media (VectorLab,Burlingame, CA,USA). Sections with HNP NeoHep and healthy NeoHep were viewed under Plan-Apochromat 63X/1.4 oil objective lens using Confocal laser scanning microscope, LSM 510 META (Zeiss,Oberkochen, Germany) and LSM 700 (Zeiss,Oberkochen, Germany) respectively.

Fluorescence in situ hybridisation (FISH) to detect human nucleus in human cell transplanted NOD SCID mouse liver section

FISH was performed using RTU Human Specific-Centro Probes Biotin Chromosome 9 (Cambio Ltd, Cambridge, UK) on the cryosection of NOD SCID mouse liver transplanted with human cells. The hybridisation procedure was followed according to the manufacturer's instruction. Briefly, liver cryosection was permeabilized using 0.2% Triton X-followed by treatment with 8% sodium thiocyanate (Sigma, St.Louis, MO, USA).The slide was incubated with 0.4% pepsin solution and then pepsin was quenched by adding 0.4% glycine (Sigma, St.Louis, MO, USA) in 2X PBS. Slide was fixed with 4% formaldehyde solution and dehydrated using ethanol series. The cellular DNA was denatured by immersing slide in 70% formamide (Sigma, St.Louis, MO, USA) solution in 2X SSC at 70^{0}C for 2 minutes immediately followed by immersing in ice cold 70% ethanol solution for 2 minutes. The slide was again dehydrated in ethanol series and air dried at room temperature. The probe was diluted in hybridization buffer, denatured for 10 minutes at 80^{0}C and immediately chilled in ice after denaturation. The denatured probe was applied on the air dried section and allowed to hybridise for 16 hours approximately at 37^{0}C in humidified chamber. The slide was washed in formamide wash solution (50% Formamide solution in 2X SSC) followed by wash with detergent wash solution (0.005% tween 20 in 4X SSC).

2µg of streptavidin Alexa Fluor 594 (Molecular Probes, Pittsburgh, PA, USA) in 100µl staining buffer (PBS+0.1% Tween-20) was added in each slide and incubated at 37^{0}C for 30 minutes. The nucleus was counter stained with DAPI (50µg/ml) at room temperature for 10 minutes and the slide was mounted with Vectashield Mounting Media (Imaging was done using 63X objective of Nikon A1R$^+$confocal microscope (Nikon Corporation, Chiyoda, Tokyo, Japan).

Live cell imaging

Cell IQ-SLF (CM Technologies, Tampere, Finland) was used to monitor the differentiation process of Monocyte to NeoHep for a period of 400 hours continuously at a particular field with routine change of media. The images were recorded at an interval of 4 hours in each grid (487µm X 364 µm) under 20X objective using Cell-IQ Imagen software v2.9.5 c (CM Technologies, Biokatu, Tampere, Finland).

The images were analyzed using Cell-IQ Analyzer post aquisition (CM Technologies, Tampere, Finland).

Statistical Analysis

All data represent at least three biological replicates (independent experiment with different blood donors) unless otherwise indicated. Error bars, where present indicate the standard error mean (SEM). No statistical method was used to predetermine sample size. Statististical significance of difference between two groups were calculated by student's unpaired t-test (Prism 5; GraphPad software Inc). Two-tailed *P* value of <0.05 were considered statistically significant. P values were categorized as ns (non-significant) P>0.05; *(significant) P < 0.05; **(very significant)P < 0.01 and ***(extremely significant)P < 0.001.

Reference List

1. Rasmussen MK, Ekstrand B, Zamaratskaia G. Comparison of cytochrome P450 concentrations and metabolic activities in porcine hepatic microsomes prepared with two different methods. TOXICOL. IN VITRO 2011;25(1):343-346.

2. Cox MP, Peterson DA, Biggs PJ. SolexaQA: At-a-glance quality assessment of Illumina second-generation sequencing data. BMC. BIOINFORMATICS. 2010;11:485.

3. Langmead B, Salzberg SL. Fast gapped-read alignment with Bowtie 2. NAT. METHODS 2012;9(4):357-359.

4. Trapnell C, Roberts A, Goff L et al. Differential gene and transcript expression analysis of RNA-seq experiments with TopHat and Cufflinks. NAT. PROTOC. 2012;7(3):562-578.

5. Trapnell C, Williams BA, Pertea G et al. Transcript assembly and quantification by RNA-Seq reveals unannotated transcripts and isoform switching during cell differentiation. NAT. BIOTECHNOL. 2010;28(5):511-515.

6. de Hoon MJ, Imoto S, Nolan J et al. Open source clustering software. BIOINFORMATICS. 2004;20(9):1453-1454.

7. Nitsche A, Becker M, Junghahn I et al. Quantification of human cells in NOD/SCID mice by duplex real-time polymerase-chain reaction. HAEMATOLOGICA 2001;86(7):693-699.

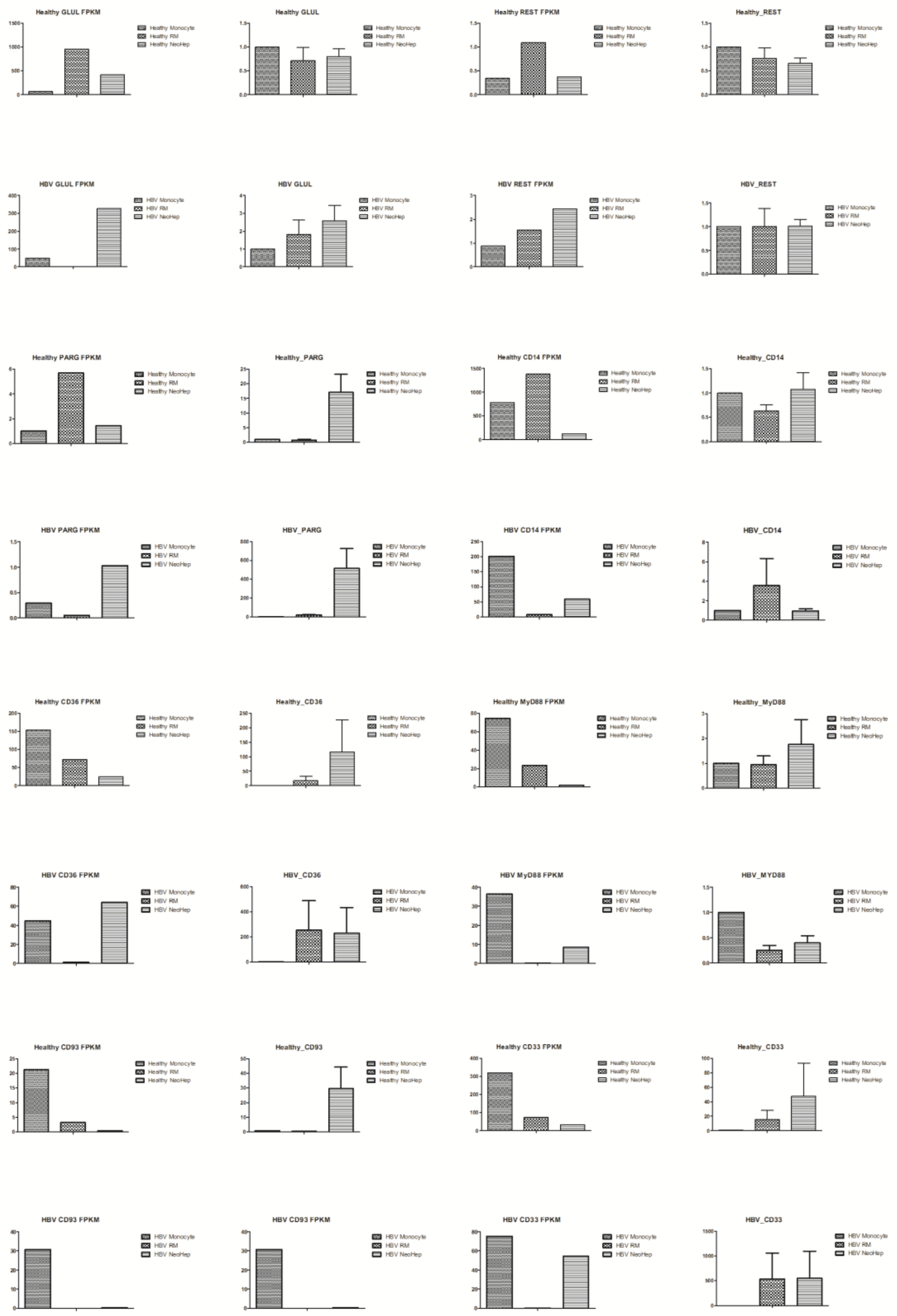

Supplementary Figure s1: Validation plot of RNASeq data using qPCR.

Fastq name	Raw Read Count	Adaptor_trim count	Reads with phred cutoff >20
Healthy Monocyte R1	57646548	56536505	56536505
Healthy Monocyte R2	57646548	56536505	56536505
Healthy RM R1	60605433	59906019	59906019
Healthy RM R2	60605433	59906019	59906019
HBV Monocyte R1	61194745	60343222	60343222
HBV Monocyte R2	61194745	60343222	60343222
Hepatocyte R1	50971726	50325047	50325047
Hepatocyte R2	50971726	50325047	50325047
Healthy Neohep R1	38856658	33979948	33979948
Healthy NeoHep R2	38856658	33979948	33979948
HBV RM R1	25047825	22388810	22388810
HBV RM R2	25047825	22388810	22388810
HBV NeoHep R1	89405621	85597162	85597162
HBV NeoHep R2	89405621	85597162	85597162

Supplementary Table sT1. Quantitation of reads in each sample having phred score > 20.

Fastq Name	tophat accepted reads	tophat discard reads	total reads	tophat mapped reads	tophat unmapped reads	mapping percentage
Healthy Monocyte R1	56321387	215118	111822233	107458534	5614476	96.10
Healthy Monocyte R2	55500846	1035659				
Healthy RM R1	59705480	200539	118497905	114520022	5282016	96.64
Healthy RM R2	58792425	1113594				
HBV Monocyte R1	60120758	222464	119231736	115186236	5500208	96.61
HBV Monocyte R2	59110978	1232244				
Hepatocyte R1	50158570	166477	99412181	96180497	4469597	96.75
Hepatocyte R2	49253611	1071436				
Healthy Neohep R1	33838811	141137	66741765	58657011	9302885	87.89
Healthy NeoHep R2	32902954	1076994				
HBV RM R1	22152116	236694	43573963	40702049	4075571	93.41
HBV RM R2	21421847	966963				
HBV NeoHep R1	85257759	339403	168983152	164835757	6358567	97.55
HBV NeoHep R2	83725393	1871769				

Supplementary Table sT2. Mapping percentage of reads on human genome, hg19, for each sample using TOPHAT.

Pictorial description of hepatectomy and cell transplantation through splenic route

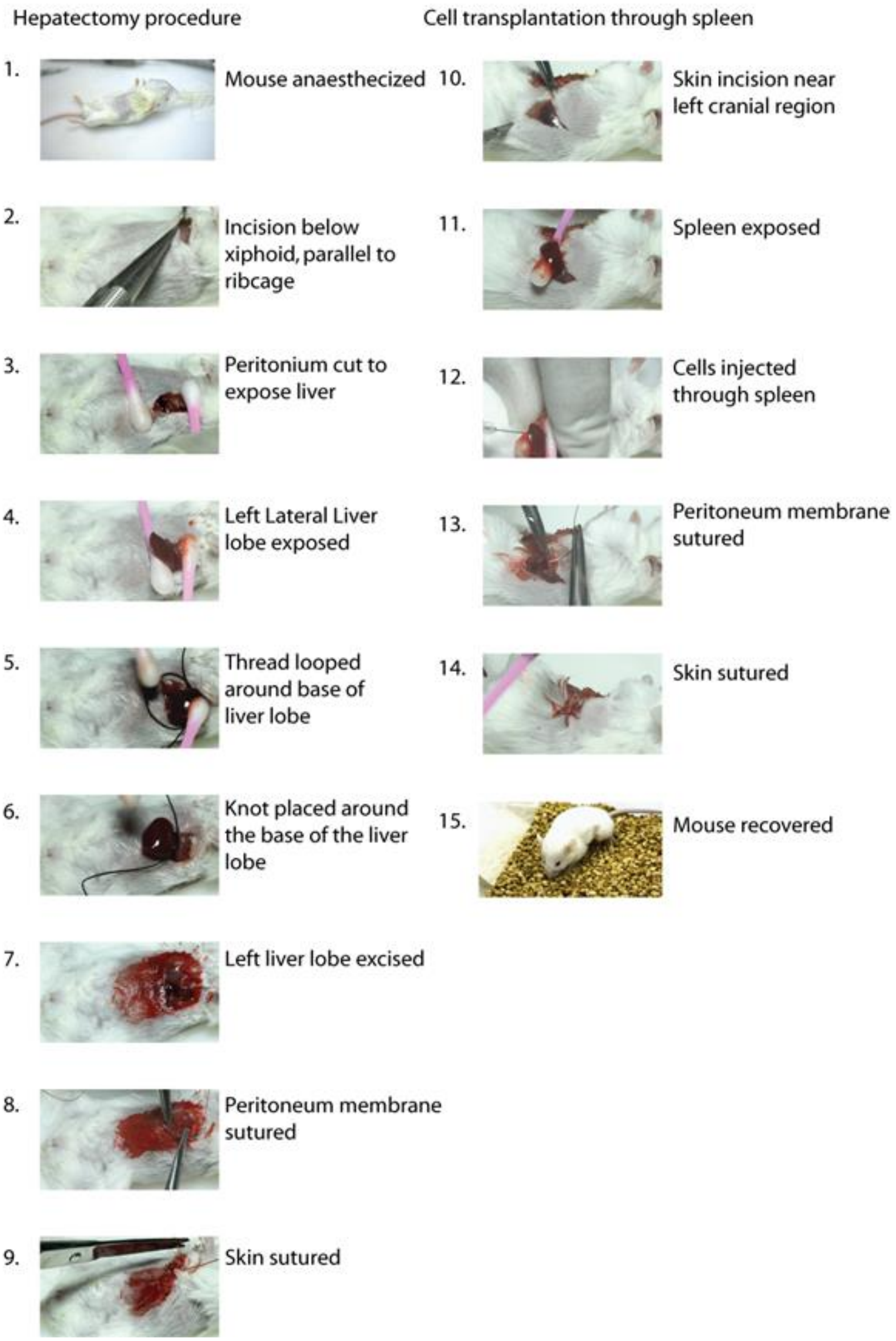

Supplementary Figure s2: Hepatectomt and Cell infusion procedure.

Primer Sequences

Gene Symbol	Gene description	Sense Primer	Anti-sense Primer	Annealing Temperature(^{o}C)
HBsAg	Hepatitis B surface antigen	CCAATCTCCAATCACTCACCAACC	ATAGCAGCAGGATGAAGAGGAAGA	60
HBxAg	Hepatitis B X protein	GCATGGAGACCACCGTGAA	TGACATCGCTGAGAGTCCAAGAG	60
Gapdh	glyceraldehyde-3-phosphate dehydrogenase	CAACAGCCTCAAGATCATCAG	GAGTCCTTCCACGATACCAA	60
Cyp3A4	cytochrome P450, subfamily IIIA, polypeptide 4	GGGTGGTGGTGATGATTCC	TGTTGTCCTTGTTCTTCTTGCT	60
CD14	Cluster of Differentiation 14	AGGTTCGGAAGACTTATC	GAAATCTTCATCGTCCAG	60
CD33	Cluster of Differentiation 33	TCTGCCTCATCTTCTTCA	ATGTAACTTGGACTTCTTCTG	60
CD36	Cluster of Differentiation 36	GCCTTCTCATCACCAATGGTCCCA	GCAGGTCAACCTATTGGTCAAGCC	60
CD93	Cluster of Differentiation 93	GAGAACCAGTACAGTCCGACA	ATGGTGGCTGGTGACTCT	60
Glul	glutamate-ammonia ligase	CCTGCTTGTATGCTGGAGT	TCCCATGCTGATTCCTTCAC	60
Myd88	myeloid differentiation factor 88	CACCTGTGTCTGGTCTATTGC	AAGTCACATTCCTTGCTCTGC	60
Parg	poly (ADP-ribose) glycohydrolase	CCGTCCTGGAGTTTCTTC	ATAAACCACATCTCGCTCAG	60
Rest	RE1-silencing transcription factor	GCGTACTCATTCAGGTGAGAAGC	ACAGTGTGGGCAATTAAGAGGTTT	60
Chd7	chromodomain helicase DNA binding protein 7	TGTAGGCGAGTATGTCGAATGC	TCTGTGATCGGCTCAATTATGGAG	60.5
Eed	embryonic ectoderm development	GCAGTGACGAGAACAGCAATC	TATCAGGGCGTTCAGTGTTTGTA	60
Ehmt1	euchromatic histone-lysine N-methyltransferase 1	TGAACATCCACGGAGACTC	TCTTTAAGGTGACATCTGAATCC	60
Ezh2	enhancer of zeste homolog 2	GGACCACAGTGTTACCAGCAT	TGGGGTCTTTATCCGCTCAG	60
Smarca 4	SWI/SNF related, matrix associated, actin dependent regulator of chromatin, subfamily a, member 4	ACTACAGCGACTCACTGA	CCTCCTCTTCGATCTCCT	60
Suz12	suppressor of zeste 12 homolog	AGAGCAACATGGGAGACTATTC	TTCTAGTGGCAAGAGGTTTGG	60
Epas1	endothelial PAS domain protein 1	GGAGTATGAAGAGCAAGCC	CATCCGTTTCCACATCAAAT	60
Fbxo15	F-box protein 15	AGAACACCTACCTCTTATTGGA	TCTTCCTTCCGCATCAAC	55
Parp1	poly (ADP-ribose) polymerase family, member 1	CTTCTGGAGGACGACAAGG	TTGGACGGCATCTGTTCC	60.5
Sall4	sal-like 4	GGAAGAACTTCTCGTCTGC	GGCTGAGTTATTGTTCGC	60.5
Hif1A	hypoxia inducible factor 1, alpha subunit (basic helix-loop-helix transcription factor)	TACCCTAACTAGCCGAGGAAGAA	AATCAGCACCAAGCAGGTCATA	60
Met	met proto-oncogene	CATCAGAGGGTCGCTTCAT	TGTATGCTCCACAATCACTTCT	60
Gab1	GRB2-associated binding protein 1	TCCCATGAACCCAAACCTGTCCAG	GGGCTGCTTCCTCCATCAAGACTG	60.4
Grb2	growth factor receptor-bound protein 2	CATAGAACAGGTGCCACAGC	CCAGCTCTCCATCCTCCT	59.1
Pik3R1	phosphoinositide-3-kinase, regulatory subunit 1 (alpha)	ACTGCCTCCTAAACCACCAAAACC	AAAGGTCCCGTCTGCTGTATCTC	60
Plcg1	phospholipase C, gamma 1	CTGTGGTTCCCATCAAACT	TGTTCTCGTCCAAGTGCT	61.8
Ptprm	protein tyrosine phosphatase, receptor type, M	ACGGAGGGTGAACATGGTGCAA	CCACAAAGACAGGCTTCCAGGATC	61.8
Ranbp9	RAN binding protein 9	TCAGAACAACCTGCGGGTG	TGGTATTGGATGCGTGGCT	60
Shc1	SHC (Src homology 2 domain containing) transforming protein 1	CAATCACTCTCACCGTCTCC	GTGGTGGTTGGCGATGAT	59.1
Src	v-src sarcoma (Schmidt-Ruppin A-2) viral oncogene homolog	GCTCCAGATTGTCAACAACAC	GATGTAGCCTGTCTGTCCTG	61.8
	Customised oligonucleotide for HBV positive control	GTGTCTTGGCCAAAATTCGCAGTCCCCAATCTCCAATCACTCACCAACCTCTTGTCCTCCAAT TTGTCCTGGCTATCGCTGGATGTGTCTGCGGCGTTTTATCATCTTCCTCTTCATCCTGCTGCTA TGCCTCATCTTCTTGTTGGTTCT		
				Sequence of the Probe-5'-3'
	Human Gapdh Taqman Probe	ACATCGCTCAGACACCAT	GCAACAATATCCACTTTACCAGAG	6-FAM-TCCGTTG ACTCCGA CCTTCAC CTTCC-TAMRA
	Human TNF-alpha TaqMan Probe	AGGAACAGCACAGGCCTTAGTG	AAGACCCCTCCCAGATAGATGG	6-FAM – CCAGGAT GTGGAGA GTGAACC GACATG-TAMRA

Supplementary Table sT3. Primer sequence used in conventional PCR and qRT-PCR.

Biological Process (BP_FAT) associated with genes having decreased expression in **H_RM** with respect to H_Monocyte

Term	PValue	Genes	Benjamini
GO:0006952~defense response	1.00E-17	NFKBID, S100A8, CLU, S100A9, PGLYRP1, HP, GCH1, CXCL10, AZU1, FOS, MEFV, HIST1H2BJ, ITIH4, LTF, PTX3, THBS1, FOSL1, NFKBIZ, RNASE3, CAMP, MAP2K3, IL1RN, MECOM, NLRP3, NLRP1, S100A12, CD1D, PROK2, CD83, CORO1A, BPI, PPBP, IRF7, LYST, DEFA4, DEFA3, MPO, CLEC7A, TREML1, HDAC9, KDM6B, CTSG, CLEC1B	1.75E-14
GO:0009617~response to bacterium	3.17E-10	RNASE3, CAMP, PGLYRP1, NFKBIA, MECOM, S100A12, GCH1, CD1D, AZU1, FOS, BPI, PPBP, LYST, DEFA4, HIST1H2BJ, DEFA3, LTF, IRG1, CTSG	2.76E-07
GO:0006955~immune response	3.50E-09	GPR183, CLU, PGLYRP1, PF4, VIPR1, PF4V1, GCH1, CXCL10, FCN1, LTF, THBS1, PTX3, IL1RN, IGJ, CD1C, CD300E, CTLA4, NLRP3, FOXP1, CD1D, HLA-F, CD83, CORO1A, OASL, BPI, RGS1, PPBP, LYST, CD79B, CLEC7A, RNF19B, TREML1, CTSG	2.03E-06
GO:0006954~inflammatory response	8.43E-09	NFKBIZ, S100A8, NFKBID, MAP2K3, CLU, S100A9, IL1RN, MECOM, NLRP3, S100A12, CXCL10, AZU1, FOS, PROK2, MEFV, IRF7, ITIH4, CLEC7A, HDAC9, THBS1, PTX3, KDM6B	3.66E-06
GO:0009611~response to wounding	9.62E-09	NFKBID, S100A8, CLU, S100A9, PF4, CXCL10, AZU1, FOS, MEFV, GP1BB, ITIH4, THBS1, NRG1, PTX3, NFKBIZ, MAP2K3, IL1RN, MECOM, NLRP3, S100A12, PROK2, EREG, F5, IRF7, CLEC7A, HDAC9, TREML1, KDM6B	3.34E-06
GO:0042742~defense response to bacterium	6.80E-08	RNASE3, CAMP, PGLYRP1, S100A12, AZU1, BPI, PPBP, LYST, HIST1H2BJ, DEFA4, DEFA3, LTF, CTSG	1.97E-05
GO:0042981~regulation of	1.29E-07	MEF2C, CLU, NFKBIA, PF4,	3.20E-05

apoptosis		ZBTB16, PMAIP1, TCF7L2, GCH1, AZU1, CDKN2D, THBS1, NRG1, MYC, FOSL1, MAP2K6, AIFM3, LGALS12, NR4A2, NR4A1, NLRP3, NLRP1, SERPINB9, PROK2, BTG2, CARD16, DUSP1, LYST, CARD17, MPO, GHRL, TNFAIP3, SCAND1, ALOX12	
GO:0043067~regulation of programmed cell death	1.61E-07	MEF2C, CLU, NFKBIA, PF4, ZBTB16, PMAIP1, TCF7L2, GCH1, AZU1, CDKN2D, THBS1, NRG1, MYC, FOSL1, MAP2K6, AIFM3, LGALS12, NR4A2, NR4A1, NLRP3, NLRP1, SERPINB9, PROK2, BTG2, CARD16, DUSP1, LYST, CARD17, MPO, GHRL, TNFAIP3, SCAND1, ALOX12	3.50E-05
GO:0010941~regulation of cell death	1.75E-07	MEF2C, CLU, NFKBIA, PF4, ZBTB16, PMAIP1, TCF7L2, GCH1, AZU1, CDKN2D, THBS1, NRG1, MYC, FOSL1, MAP2K6, AIFM3, LGALS12, NR4A2, NR4A1, NLRP3, NLRP1, SERPINB9, PROK2, BTG2, CARD16, DUSP1, LYST, CARD17, MPO, GHRL, TNFAIP3, SCAND1, ALOX12	3.39E-05
GO:0006916~anti-apoptosis	1.60E-06	MEF2C, CLU, NFKBIA, TCF7L2, AZU1, PROK2, SERPINB9, CDKN2D, MPO, GHRL, TNFAIP3, NRG1, THBS1, MYC, ALOX12	2.77E-04
GO:0042330~taxis	3.25E-06	CMTM2, RNASE2, S100A9, PF4, CXCL10, AZU1, PROK2, CORO1A, TYMP, PPBP, LYST, FOSL1, CMTM5	5.14E-04
GO:0006935~chemotaxis	3.25E-06	CMTM2, RNASE2, S100A9, PF4, CXCL10, AZU1, PROK2, CORO1A, TYMP, PPBP, LYST, FOSL1, CMTM5	5.14E-04
GO:0007610~behavior	1.35E-05	RNASE2, S100P, CMTM2, S100A9, NR4A2, PGLYRP1, PF4, FOSB, CXCL10, ATXN1, AZU1, FOS, PROK2, TYMP, CORO1A, PPBP, LYST, GHRL, PLCB1, FOSL1, CMTM5	1.95E-03
GO:0043066~negative regulation of apoptosis	1.37E-05	MEF2C, CLU, NR4A2, NFKBIA, PF4, TCF7L2, AZU1, PROK2,	1.83E-03

		SERPINB9, BTG2, CDKN2D, MPO, GHRL, TNFAIP3, NRG1, THBS1, MYC, ALOX12	
GO:0043069~negative regulation of programmed cell death	1.63E-05	MEF2C, CLU, NR4A2, NFKBIA, PF4, TCF7L2, AZU1, PROK2, SERPINB9, BTG2, CDKN2D, MPO, GHRL, TNFAIP3, NRG1, THBS1, MYC, ALOX12	2.02E-03
GO:0060548~negative regulation of cell death	1.70E-05	MEF2C, CLU, NR4A2, NFKBIA, PF4, TCF7L2, AZU1, PROK2, SERPINB9, BTG2, CDKN2D, MPO, GHRL, TNFAIP3, NRG1, THBS1, MYC, ALOX12	1.97E-03
GO:0007626~locomotory behaviour	4.12E-05	RNASE2, CMTM2, S100A9, NR4A2, PF4, CXCL10, AZU1, ATXN1, PROK2, TYMP, CORO1A, PPBP, LYST, FOSL1, CMTM5	4.46E-03
GO:0031328~positive regulation of cellular biosynthetic process	4.62E-05	MEF2C, NFKBIA, TCF7L2, AZU1, FOS, THBS1, PTX3, MYC, FOSL1, SAMD4A, FCER1A, NFE2, IKZF1, MAP2K3, NR4A2, NR4A1, CREB5, MECOM, JUNB, ATXN1, HES1, EREG, MTF1, CSRNP1, KLF2	4.71E-03
GO:0009891~positive regulation of biosynthetic process	5.80E-05	MEF2C, NFKBIA, TCF7L2, AZU1, FOS, THBS1, PTX3, MYC, FOSL1, SAMD4A, FCER1A, NFE2, IKZF1, MAP2K3, NR4A2, NR4A1, CREB5, MECOM, JUNB, ATXN1, HES1, EREG, MTF1, CSRNP1, KLF2	5.59E-03
GO:0010557~positive regulation of macromolecule biosynthetic process	6.35E-05	MEF2C, FCER1A, NFE2, IKZF1, MAP2K3, NR4A2, NFKBIA, NR4A1, CREB5, MECOM, TCF7L2, JUNB, ATXN1, HES1, AZU1, FOS, MTF1, EREG, CSRNP1, KLF2, THBS1, MYC, FOSL1, SAMD4A	5.79E-03
GO:0006357~regulation of transcription from RNA polymerase II promoter	1.17E-04	MEF2C, NFKBIA, ZNF345, TCF7L2, FOS, TAL1, GFI1B, JUND, MYC, FOSL1, NFE2, IKZF1, NR4A2, NR4A1, FOSB, MECOM, FOXP1, JUNB, ATXN1, HES1, MTF1, CSRNP1, IRF7, HDAC9, KDM6B	1.01E-02
GO:0010033~response to organic substance	2.68E-04	ADCY4, SELL, IL1RN, NR4A2, NFKBIA, GNG11, PMAIP1, JUNB, GCH1, GNG8, CD83, FOS, RETN, CORO1A, DUSP1, BTG2,	2.19E-02

		GHRL, CLEC7A, HDAC9, THBS1, IRG1, PPP1R15A, FOSL1, MYC	
GO:0001817~regulation of cytokine production	2.82E-04	FCER1A, AZU1, CD83, BPI, EREG, MAP2K3, GHRL, PF4, CLEC7A, THBS1, NLRP3	2.21E-02
GO:0009620~response to fungus	3.48E-04	DEFA4, DEFA3, CLEC7A, PTX3, S100A12	2.59E-02
GO:0043085~positive regulation of catalytic activity	4.98E-04	FCER1A, ADCY4, AIFM3, MAP2K3, NR4A2, ZEB2, PMAIP1, NLRP3, NLRP1, GCH1, AZU1, PROK2, EREG, RASGRP4, GHRL, NRG1, THBS1, MYC, MAP2K6	3.54E-02
GO:0010628~positive regulation of gene expression	6.95E-04	MEF2C, NFE2, IKZF1, MAP2K3, NR4A2, NFKBIA, NR4A1, CREB5, PF4, MECOM, TCF7L2, JUNB, HES1, ATXN1, FOS, MTF1, CSRNP1, KLF2, FOSL1, MYC	4.72E-02

Biological Process (BP_FAT) associated with genes having decreased expression in **H_NeoHep** with respect to H_Monocyte

Term	PValue	Genes	Benjamini
GO:0006955~immune response	8.55E-19	XRCC4, IL16, AQP9, SNCA, PGLYRP1, TLR2, TNFSF14, IL15, VIPR1, PNP, B2M, CXCL10, CFP, LILRA1, MYD88, LILRA2, CLEC4E, LTB4R, S1PR4, LILRA4, CLEC4A, LILRA6, IL1B, ERAP2, CLEC4D, AKIRIN2, IL1A, SPN, RAB27A, GTPBP1, CRTAM, GBP5, NCF2, BST1, NCF1, HLA-A, SERPING1, HLA-C, HLA-B, HLA-F, LILRB1, IGSF6, LILRB2, CCR7, LAT2, PPBP, LYST, GBP4, GBP3, GBP2, GBP1, HLA-DRA, IFITM2, CLU, OAS3, OAS2, PF4V1, CCL7, IL23A, DHX58, ARHGDIB, CARD9, IL1RN, IGJ, CD300E, SMAD3, FOXP1, CD55, OASL, TNFSF10, CORO1A, CLEC7A, TREML1, NBN, PRDX2, NFKB2, TMEM173, NOD2, IL1RAP, TICAM1, LTF, NFIL3, ICAM1, IK, EXOSC9, PTGER4, NLRP3, PRKCD, WAS, OSM, CD83, BPI, C1QBP, TREM1, CTSG, LCP1, LCP2, GPR183, YWHAZ, CXCL3, CXCL2, CALCOCO2, PF4, CLEC10A, GCH1, CCL20, XBP1, FCGR1A, FCN1, POU2F2, BCL3, THBS1, PTX3, SECTM1, IL6, CEBPB, OLR1, IL8, CEBPG, CTLA4, CD1C, CD1D, TNFSF8, ILF2, IRF8, C1RL, RNF19B, CD302	3.01E-15
GO:0006952~defense response	5.06E-18	S100A8, S100A9, SNCA, PGLYRP1, TLR2, IL15, CXCL10, CFP, CD48, LILRA1, MYD88, LILRA2, CD44, LTB4R, HIST1H2BJ, CSF3R, IL1B, MX2,	8.90E-15

		AKIRIN2, IL1A, SPN, RAB27A, NCF2, NCF1, GNLY, SERPING1, HLA-C, HLA-B, MECOM, SP140, TNFAIP6, LILRB2, CCR7, PPBP, TIAL1, LYST, F3, DEFA4, DEFA3, MNDA, RIPK2, HLA-DRA, FGR, NFKBID, ADORA2A, CLU, CCL7, IL23A, DHX58, B4GALT1, CARD9, MAP2K3, IL1RN, S100A12, APOL3, CD55, CORO1A, P2RX1, CLEC7A, TREML1, HDAC9, PRDX2, NFKB1, HP, CASP6, TMEM173, NOD2, IL1RAP, TICAM1, ITIH4, LTF, SERPINA1, TNIP1, FOSL1, IRAK2, NFKBIZ, CAMP, LYZ, NLRP3, WAS, NLRP1, PROK2, CD83, BPI, HIST2H2BE, CTSG, KDM6B, CLEC1B, YWHAZ, NMI, CXCL3, CXCL2, CALCOCO2, GCH1, AZU1, MEFV, CCL20, FCGR1A, BCL3, THBS1, PTX3, IL6, CEBPB, AIMP1, IL8, OLR1, HCK, CEBPG, ANXA1, COTL1, CD1D, ORM1, C1RL, MPO, LTA4H, ALOX5, CD302	
GO:0006414~translational elongation	4.05E-14	RPL18, RPL17, RPL36A, EEF1B2, RPL15, RPS15A, RPL37, RPL38, RPS25, RPS27, RPL7, RPL31, RPL6, RPL9, RPL34, RPL3, RPL5, RPL7A, RPS20, RPS21, RPS23, RPS24, RPL35A, RPL26, RPL27, RPL24, RPS6, RPS4X, HNRNPH2, RPS16, RPL22, RPL13A, RPS14, RPL21, RPS13, RPS10, UBB	4.75E-11
GO:0006412~translation	2.03E-13	MRPS36, RPL18, RPL17, RPL36A, MRPL42, RBM3, RPL15, EIF2A, EIF1AY, POLG2, MRPL32, MRPL33, TNIP1, RPL36AL, MRPL1,	1.78E-10

		RPL35A, MRPL3, EIF1B, DARS2, RPS4X, GTF2B, RSL1D1, RPS16, EIF2S1, RPS14, FARSB, RPS13, RPS10, UBB, NACA, EEF1B2, FARS2, RPS15A, RPL37, RPL38, MTIF3, RPS25, RPS27, RPL7, RPL31, RPL6, EIF3H, EIF3E, RPL34, RPL9, EIF3F, MRPL16, MRPL19, RPL3, MRPL18, RPL5, RPL7A, RPS20, RPS21, RPS23, EIF3M, RPS24, AIMP1, RPL26, RPL27, RPL24, RPS6, EIF4B, MRPL21, HNRNPH2, EIF4E, RPL13A, RPL22, EEF1E1, RPL21, CARS2	
GO:0042981~regulation of apoptosis	1.33E-11	ITGB3BP, MEF2C, XRCC4, PTGS2, SNCA, TLR2, TNFSF14, PMAIP1, BTK, CITED2, MYD88, CD44, CDKN2D, TPT1, IL1B, WWOX, IL1A, MAP2K6, SPN, RAB27A, CRTAM, SOCS3, BCL2A1, PIM3, PIM2, BCL2L13, STK3, TIAL1, F3, LYST, TNFAIP8, RIPK2, TNFAIP3, TRAF1, MCL1, ADORA2A, ADAMTSL4, CLU, STK17B, NFKBIA, PPT1, SCRIB, TAF9, PLAGL2, B4GALT1, CFLAR, HERPUD1, CARD9, KLF10, NR4A2, NR4A1, SMAD3, RPS6, PPIF, TNFSF10, HDAC3, RNF7, P2RX1, HDAC1, PDCD6, PDIA3, PRDX2, NFKB1, PRDX3, PTEN, CASP5, ARHGAP4, CASP6, NOD2, CASP4, AEN, TICAM1, NRG1, CASP1, MYC, FOSL1, CYCS, LGALS12, NLRP3, NLRP1, PROK2, SERPINB9, TNFRSF10B, BTG2, CARD16, BNIP2, BTG1, CARD17, SERPINB2, GHRL, UBB, ALOX12, BID,	9.37E-09

		ING3, YWHAZ, PF4, ZBTB16, TCF7L2, GCH1, AZU1, PLEKHG2, PPP2CA, BCL3, HSPA5, THBS1, IL6, CEBPB, CEBPG, CREB1, ANXA1, ANXA5, SOD2, TNFSF8, HSP90B1, SON, PSMG2, DUSP1, GSK3B, EEF1E1, IFT57, NLRP12, MPO	
GO:0010941~regulation of cell death	1.55E-11	ITGB3BP, MEF2C, XRCC4, PTGS2, SNCA, TLR2, TNFSF14, PMAIP1, BTK, CITED2, MYD88, CD44, CDKN2D, TPT1, IL1B, WWOX, IL1A, MAP2K6, SPN, RAB27A, CRTAM, SOCS3, BCL2A1, PIM3, PIM2, BCL2L13, STK3, TIAL1, F3, LYST, TNFAIP8, RIPK2, TNFAIP3, TRAF1, MCL1, ADORA2A, ADAMTSL4, CLU, STK17B, NFKBIA, RRAGA, PPT1, SCRIB, TAF9, PLAGL2, B4GALT1, CFLAR, HERPUD1, CARD9, KLF10, NR4A2, NR4A1, SMAD3, RPS6, PPIF, TNFSF10, HDAC3, RNF7, P2RX1, HDAC1, PDCD6, PDIA3, PRDX2, NFKB1, PRDX3, PTEN, CASP5, ARHGAP4, CASP6, NOD2, CASP4, AEN, TICAM1, NRG1, CASP1, MYC, FOSL1, CYCS, LGALS12, NLRP3, NLRP1, PROK2, SERPINB9, TNFRSF10B, BTG2, CARD16, BNIP2, BTG1, CARD17, SERPINB2, GHRL, UBB, ALOX12, BID, ING3, YWHAZ, PF4, ZBTB16, TCF7L2, GCH1, AZU1, PLEKHG2, PPP2CA, BCL3, HSPA5, THBS1, IL6, CEBPB, CEBPG, CREB1, ANXA1, ANXA5, SOD2, TNFSF8, HSP90B1, SON, PSMG2, DUSP1, GSK3B,	9.10E-09

		EEF1E1, IFT57, NLRP12, MPO	
GO:0043067~regulation of programmed cell death	2.57E-11	ITGB3BP, MEF2C, XRCC4, PTGS2, SNCA, TLR2, TNFSF14, PMAIP1, BTK, CITED2, MYD88, CD44, CDKN2D, TPT1, IL1B, WWOX, IL1A, MAP2K6, SPN, RAB27A, CRTAM, SOCS3, BCL2A1, PIM3, PIM2, BCL2L13, STK3, TIAL1, F3, LYST, TNFAIP8, RIPK2, TNFAIP3, TRAF1, MCL1, ADORA2A, ADAMTSL4, CLU, STK17B, NFKBIA, PPT1, SCRIB, TAF9, PLAGL2, B4GALT1, CFLAR, HERPUD1, CARD9, KLF10, NR4A2, NR4A1, SMAD3, RPS6, PPIF, TNFSF10, HDAC3, RNF7, P2RX1, HDAC1, PDCD6, PDIA3, PRDX2, NFKB1, PRDX3, PTEN, CASP5, ARHGAP4, CASP6, NOD2, CASP4, AEN, TICAM1, NRG1, CASP1, MYC, FOSL1, CYCS, LGALS12, NLRP3, NLRP1, PROK2, SERPINB9, TNFRSF10B, BTG2, CARD16, BNIP2, BTG1, CARD17, SERPINB2, GHRL, UBB, ALOX12, BID, ING3, YWHAZ, PF4, ZBTB16, TCF7L2, GCH1, AZU1, PLEKHG2, PPP2CA, BCL3, HSPA5, THBS1, IL6, CEBPB, CEBPG, CREB1, ANXA1, ANXA5, SOD2, TNFSF8, HSP90B1, SON, PSMG2, DUSP1, GSK3B, EEF1E1, IFT57, NLRP12, MPO	1.29E-08
GO:0006954~inflammatory response	6.24E-11	S100A8, S100A9, TLR2, PRDX2, NFKB1, IL15, CXCL10, CFP, CASP6, MYD88, CD44, LTB4R, IL1RAP, TICAM1, ITIH4, IL1B, SERPINA1, IL1A, IRAK2, NFKBIZ, LYZ,	2.75E-08

		SERPING1, MECOM, NLRP3, TNFAIP6, PROK2, CCR7, F3, RIPK2, KDM6B, YWHAZ, NMI, NFKBID, ADORA2A, CXCL3, CXCL2, CLU, CCL7, AZU1, IL23A, CCL20, MEFV, PTX3, THBS1, B4GALT1, IL6, CEBPB, AIMP1, IL8, OLR1, MAP2K3, IL1RN, ANXA1, S100A12, ORM1, APOL3, CD55, P2RX1, C1RL, LTA4H, ALOX5, CLEC7A, HDAC9, CD302	
GO:0006916~anti-apoptosis	6.94E-10	MEF2C, YWHAZ, MCL1, CLU, SNCA, NFKBIA, NFKB1, PRDX2, TCF7L2, CITED2, AZU1, MYD88, CDKN2D, TPT1, IL1B, HSPA5, THBS1, NRG1, MYC, IL1A, CFLAR, CEBPB, SOCS3, BCL2A1, ANXA1, PIM2, ANXA5, SOD2, PROK2, SERPINB9, SON, HSP90B1, HDAC3, RNF7, HDAC1, BNIP2, GSK3B, F3, TNFAIP8, SERPINB2, MPO, GHRL, RIPK2, UBB, TNFAIP3, ALOX12	2.72E-07
GO:0009617~response to bacterium	8.70E-10	PTGS2, FGR, SNCA, TLR2, PGLYRP1, NFKBIA, PRDX2, PRDX3, COMT, GCH1, B2M, TRIB1, AZU1, CFP, NOD2, MYD88, CCL20, TICAM1, HIST1H2BJ, BCL3, IL1B, LTF, SERPINA1, IRG1, AKIRIN2, SPN, IL6, CARD9, SOCS3, CAMP, HCK, GNLY, LYZ, MECOM, S100A12, CD1D, BPI, PPBP, HIST2H2BE, LYST, DEFA4, DEFA3, RIPK2, CTSG	3.06E-07
GO:0009611~response to wounding	1.87E-09	S100A8, F13A1, S100A9, TLR2, PRDX2, NFKB1, IL15, CXCL10, LUZP6, CFP, CASP6, MYD88, CD44, GSN, LTB4R, IL1RAP,	5.99E-07

		TICAM1, ITIH4, IL1B, SERPINA1, NRG1, IL1A, RAB27A, IRAK2, NFKBIZ, LYZ, SERPING1, NLRP3, MECOM, WAS, PLAUR, PROK2, TNFAIP6, CCR7, F5, EREG, F3, SERPINB2, RIPK2, VCAN, KDM6B, YWHAZ, NMI, NFKBID, ADORA2A, CXCL3, CXCL2, CLU, PF4, TPM1, CCL7, AZU1, MIA3, IL23A, MEFV, CCL20, GP1BB, THBS1, PTX3, B4GALT1, KLF6, IL6, CEBPB, OLR1, PLEK, AIMP1, IL8, MAP2K3, IL1RN, ANXA1, SMAD3, ANXA5, S100A12, SOD2, ORM1, APOL3, CD55, P2RX1, C1RL, LTA4H, CLEC7A, ALOX5, TREML1, HDAC9, CD302	
GO:0001775~cell activation	1.21E-08	XRCC4, NBN, SNCA, TLR2, TNFSF14, PRDX2, IL15, NFKB2, CD48, CD93, TICAM1, SPN, RAB27A, GAPT, EGR1, ICAM1, CRTAM, WAS, PRKCD, NCK2, LAT2, CCND3, CD80, IGBP1, RIPK2, LCP1, LCP2, GPR183, YWHAZ, ADORA2A, PF4, HPRT1, HSH2D, AZU1, IL23A, GP1BB, BCL11A, BCL3, KLF6, IL6, PLEK, IKZF1, IL8, CEBPG, SMAD3, FOXP1, CD1D, P2RX1, FYN, RPL22, IRF1, CLEC7A, TREML1, HDAC9	3.54E-06
GO:0006396~RNA processing	1.86E-08	NCBP1, RPL36A, RBM3, LSM6, CWC15, NONO, PCBP1, U2AF1, RBMS2, LSM5, QKI, MAGOHB, DUS1L, TSEN15, LUC7L3, ZFP36, MRPL1, RPL35A, SNRPA1, EXOSC8, EXOSC9, SF3B14, MAGOH, HNRNPA2B1, EXOSC5, EXOSC3, PRPF3, RSL1D1, TARBP1, AQR, RPS16,	5.03E-06

		RPS14, CPSF6, SLU7, RBM39, CPSF3, SNRPG, FUS, PPP4R2, POLR2E, TRA2B, FARS2, TRA2A, SNRPB2, WBP11, QTRT1, FCF1, NAA38, ZFC3H1, HNRNPL, HNRNPM, HNRNPK, RPL7, HNRNPF, PPP2CA, HNRNPD, DHX15, THG1L, WDR12, RPL5, HNRNPC, PABPC1, KIAA0391, NSA2, PRPF40A, RPS24, DUS3L, BCAS2, RPL26, SMAD3, SSB, RPS6, RPF1, HNRNPA0, TRNT1, ATXN1, HNRNPH3, HNRNPH2, SYF2, NOP58, RNPC3, SNRNP27, RBM14, POP5, RBM17	
GO:0045321~leukocyte activation	1.25E-07	GPR183, XRCC4, NBN, YWHAZ, SNCA, TLR2, TNFSF14, PRDX2, IL15, HPRT1, HSH2D, AZU1, CD48, IL23A, CD93, TICAM1, BCL11A, BCL3, SPN, RAB27A, GAPT, EGR1, ICAM1, KLF6, CRTAM, IL8, IKZF1, CEBPG, SMAD3, WAS, PRKCD, FOXP1, CD1D, NCK2, LAT2, CCND3, CD80, FYN, RPL22, IGBP1, IRF1, RIPK2, CLEC7A, HDAC9, LCP1, LCP2	3.14E-05
GO:0043066~negative regulation of apoptosis	2.05E-07	MEF2C, XRCC4, SNCA, PRDX2, NFKB1, PRDX3, PTEN, CITED2, MYD88, CDKN2D, TPT1, IL1B, NRG1, MYC, IL1A, SOCS3, BCL2A1, PIM3, PIM2, SERPINB9, PROK2, BTG2, BNIP2, F3, TNFAIP8, SERPINB2, GHRL, RIPK2, UBB, TNFAIP3, ALOX12, YWHAZ, MCL1, ADORA2A, CLU, NFKBIA, PPT1, PF4, TCF7L2, AZU1, BCL3, TAF9, HSPA5, THBS1, CFLAR, IL6,	4.80E-05

		CEBPB, NR4A2, ANXA1, SMAD3, ANXA5, SOD2, SON, HSP90B1, HDAC3, RNF7, HDAC1, GSK3B, MPO	
GO:0043069~negative regulation of programmed cell death	3.34E-07	MEF2C, XRCC4, SNCA, PRDX2, NFKB1, PRDX3, PTEN, CITED2, MYD88, CDKN2D, TPT1, IL1B, NRG1, MYC, IL1A, SOCS3, BCL2A1, PIM3, PIM2, SERPINB9, PROK2, BTG2, BNIP2, F3, TNFAIP8, SERPINB2, GHRL, RIPK2, UBB, TNFAIP3, ALOX12, YWHAZ, MCL1, ADORA2A, CLU, NFKBIA, PPT1, PF4, TCF7L2, AZU1, BCL3, TAF9, HSPA5, THBS1, CFLAR, IL6, CEBPB, NR4A2, ANXA1, SMAD3, ANXA5, SOD2, SON, HSP90B1, HDAC3, RNF7, HDAC1, GSK3B, MPO	7.36E-05
GO:0032680~regulation of tumor necrosis factor production	3.36E-07	CARD9, TLR2, PF4, AZU1, NOD2, BPI, MYD88, TICAM1, RIPK2, BCL3, GHRL, RARA, CLEC7A, SPN	6.97E-05
GO:0060548~negative regulation of cell death	3.65E-07	MEF2C, XRCC4, SNCA, PRDX2, NFKB1, PRDX3, PTEN, CITED2, MYD88, CDKN2D, TPT1, IL1B, NRG1, MYC, IL1A, SOCS3, BCL2A1, PIM3, PIM2, SERPINB9, PROK2, BTG2, BNIP2, F3, TNFAIP8, SERPINB2, GHRL, RIPK2, UBB, TNFAIP3, ALOX12, YWHAZ, MCL1, ADORA2A, CLU, NFKBIA, PPT1, PF4, TCF7L2, AZU1, BCL3, TAF9, HSPA5, THBS1, CFLAR, IL6, CEBPB, NR4A2, ANXA1, SMAD3, ANXA5, SOD2, SON, HSP90B1, HDAC3, RNF7, HDAC1, GSK3B, MPO	7.15E-05

GO:0046649~lymphocyte activation	1.63E-06	GPR183, XRCC4, NBN, TNFSF14, PRDX2, IL15, HPRT1, HSH2D, CD48, IL23A, BCL11A, BCL3, SPN, RAB27A, GAPT, EGR1, ICAM1, KLF6, CRTAM, IKZF1, CEBPG, SMAD3, WAS, PRKCD, FOXP1, CD1D, NCK2, LAT2, CCND3, CD80, FYN, RPL22, IGBP1, IRF1, RIPK2, CLEC7A, HDAC9, LCP1	3.02E-04
GO:0042742~defense response to bacterium	2.80E-06	FGR, PGLYRP1, TLR2, CFP, AZU1, NOD2, CCL20, HIST1H2BJ, LTF, BCL3, SPN, IL6, CARD9, GNLY, HCK, CAMP, LYZ, S100A12, BPI, PPBP, HIST2H2BE, LYST, DEFA4, DEFA3, RIPK2, CTSG	4.93E-04
GO:0001817~regulation of cytokine production	3.37E-06	TLR2, NFKB1, PF4, AZU1, NOD2, MYD88, TICAM1, IL1B, BCL3, RARA, THBS1, CASP1, SPN, SRGN, IL1A, AKIRIN2, FCER1A, IL6, CRTAM, CARD9, CEBPB, CREB1, MAP2K3, CEBPG, SMAD3, NLRP3, CD83, BPI, EREG, CD80, IRF1, NLRP12, GHRL, RIPK2, CLEC7A	5.65E-04
GO:0008380~RNA splicing	5.07E-06	FUS, NCBP1, PPP4R2, RPL36A, POLR2E, TRA2B, LSM6, TRA2A, SNRPB2, CWC15, WBP11, NAA38, NONO, HNRNPL, HNRNPM, HNRNPK, PPP2CA, PCBP1, HNRNPF, DHX15, HNRNPD, U2AF1, LSM5, QKI, MAGOHB, HNRNPC, PABPC1, LUC7L3, PRPF40A, BCAS2, SNRPA1, SF3B14, MAGOH, HNRNPA2B1, PRPF3, HNRNPA0, HNRNPH3, HNRNPH2, AQR, SYF2, SLU7, RBM39, RNPC3, SNRNP27, RBM14, CPSF3, RBM17, SNRPG	8.12E-04
GO:0048584~positive	6.86E-06	IL16, CLU, TLR2, NFKBIA,	1.05E-03

regulation of response to stimulus		IL15, B2M, AZU1, CFP, NOD2, MYD88, TICAM1, IL1B, TAF9, SH2B2, THBS1, AXIN1, FCER1A, IRAK2, IL6, CRTAM, CARD9, IL8, CEBPG, SMAD3, SERPING1, UIMC1, CD1D, OSM, CD55, LAT2, EREG, FYN, F3, EEF1E1, C1RL, NLRP12, BRE, GHRL, RIPK2, CLEC7A, HLA-DRA	
GO:0042110~T cell activation	8.27E-06	XRCC4, TNFSF14, PRDX2, IL15, HSH2D, CD48, IL23A, BCL11A, BCL3, SPN, RAB27A, EGR1, ICAM1, CRTAM, IKZF1, SMAD3, WAS, CD1D, NCK2, CD80, CCND3, RPL22, FYN, IRF1, RIPK2, CLEC7A, LCP1	1.21E-03
GO:0001819~positive regulation of cytokine production	8.31E-06	IL6, CARD9, CREB1, TLR2, SMAD3, PF4, NLRP3, CD83, NOD2, MYD88, EREG, TICAM1, NLRP12, RIPK2, BCL3, IL1B, RARA, CLEC7A, CASP1, THBS1, IL1A, AKIRIN2	1.17E-03
GO:0002684~positive regulation of immune system process	8.46E-06	CLU, TLR2, NFKBIA, TNFSF14, IL15, PNP, B2M, CFP, CD47, MIA3, NOD2, MYD88, TICAM1, IL1B, RARA, SH2B2, THBS1, SPN, FCER1A, IRAK2, ICAM1, IL6, CRTAM, CARD9, IKZF1, SERPING1, FOXP1, CD1D, NCK2, CD83, CORO1A, CD55, CD37, LAT2, CD80, EREG, FYN, C1RL, RIPK2, CLEC7A, HLA-DRA	1.14E-03
GO:0002237~response to molecule of bacterial origin	1.43E-05	IL6, CARD9, PTGS2, SOCS3, SNCA, TLR2, NFKBIA, PRDX2, COMT, PRDX3, TRIB1, B2M, GCH1, NOD2, MYD88, TICAM1, RIPK2, IL1B, SERPINA1, IRG1, AKIRIN2	1.86E-03
GO:0032675~regulation of interleukin-6 production	1.61E-05	IL6, CEBPB, CARD9, TLR2, NOD2, BPI, EREG, TICAM1, NLRP12, RIPK2, IL1B, GHRL, AKIRIN2	2.02E-03

GO:0016071~mRNA metabolic process	1.89E-05	NCBP1, RPL36A, LSM6, CWC15, NONO, PCBP1, LSM5, U2AF1, QKI, MAGOHB, LUC7L3, TSEN15, ZFP36, SNRPA1, SF3B14, MAGOH, HNRNPA2B1, PRPF3, AQR, SLU7, CPSF6, RBM39, CPSF3, SNRPG, FUS, PPP4R2, POLR2E, TRA2B, SNRPB2, TRA2A, WBP11, NAA38, ZFP36L1, HNRNPL, HNRNPM, ZFP36L2, HNRNPK, HNRNPF, EIF3E, DHX15, HNRNPD, PABPC3, PABPC1, HNRNPC, PRPF40A, BCAS2, CEBPG, SSB, HNRNPA0, HNRNPH3, HNRNPH2, SYF2, RNPC3, SNRNP27, RBM14, RBM17	2.29E-03
GO:0002252~immune effector process	2.60E-05	XRCC4, NBN, YWHAZ, CLU, PRDX2, CFP, NOD2, MYD88, TICAM1, FCN1, BCL3, PTX3, RAB27A, ICAM1, IL6, CRTAM, CARD9, CEBPG, SERPING1, NLRP3, PRKCD, FOXP1, CD55, LAT2, LYST, C1RL, HLA-DRA	3.04E-03
GO:0006397~mRNA processing	2.82E-05	NCBP1, RPL36A, LSM6, CWC15, NONO, PCBP1, LSM5, U2AF1, QKI, MAGOHB, LUC7L3, TSEN15, ZFP36, SNRPA1, SF3B14, MAGOH, HNRNPA2B1, PRPF3, AQR, SLU7, CPSF6, RBM39, CPSF3, SNRPG, FUS, PPP4R2, POLR2E, TRA2B, SNRPB2, TRA2A, WBP11, NAA38, HNRNPL, HNRNPM, HNRNPK, HNRNPF, DHX15, HNRNPD, PABPC1, HNRNPC, PRPF40A, BCAS2, HNRNPA0, HNRNPH3, HNRNPH2, SYF2, RNPC3, SNRNP27, RBM14, RBM17	3.20E-03

GO:0006935~chemotaxis	3.48E-05	IL16, CXCL3, S100A9, CXCL2, FPR1, PF4, CCL7, SCRIB, CXCL10, AZU1, TYMP, CCL20, RAC2, CKLF, IL1B, FOSL1, SPN, IL6, RNASE2, CMTM2, AIMP1, IL8, PLAUR, PROK2, CORO1A, CCR7, PPBP, LYST, CMTM6, CMTM5	3.82E-03
GO:0042330~taxis	3.48E-05	IL16, CXCL3, S100A9, CXCL2, FPR1, PF4, CCL7, SCRIB, CXCL10, AZU1, TYMP, CCL20, RAC2, CKLF, IL1B, FOSL1, SPN, IL6, RNASE2, CMTM2, AIMP1, IL8, PLAUR, PROK2, CORO1A, CCR7, PPBP, LYST, CMTM6, CMTM5	3.82E-03
GO:0042254~ribosome biogenesis	4.16E-05	LSM6, GTPBP10, EIF2A, WBP11, FCF1, EBNA1BP2, RPL7, WDR12, RPL5, RPL7A, RPS24, NSA2, RPL35A, EXOSC8, GTPBP4, EXOSC9, EXOSC5, RPL26, EXOSC3, RPL24, RPS6, RPF1, RPS16, RPS14, NOP58	4.42E-03
GO:0043122~regulation of I-kappaB kinase/NF-kappaB cascade	4.26E-05	SECTM1, CFLAR, CARD9, SLC44A2, VAPA, C9ORF89, TFG, PIM2, TAB2, LGALS9, APOL3, TNFSF10, NOD2, TNFRSF10B, MYD88, MAP3K3, TICAM1, NLRP12, RIPK2, IL1B, TNFAIP3, CASP1, PPP5C	4.40E-03
GO:0045087~innate immune response	4.41E-05	SNCA, CLU, PGLYRP1, TLR2, CALCOCO2, GCH1, CFP, NOD2, TMEM173, IL23A, MYD88, FCGR1A, IL1RAP, DHX58, AKIRIN2, RAB27A, NCF2, NCF1, CEBPG, SERPING1, CD1D, CORO1A, CD55, LYST, C1RL, CLEC7A, TREML1	4.43E-03
GO:0009620~response to fungus	6.54E-05	CARD9, MYD88, GNLY, DEFA4, DEFA3, TLR2, CLEC7A, COTL1, PTX3, S100A12	6.37E-03

GO:0010942~positive regulation of cell death	6.87E-05	ITGB3BP, PTGS2, PDIA3, TLR2, TNFSF14, PMAIP1, PTEN, BTK, ARHGAP4, CASP6, CASP4, CD44, AEN, TICAM1, IL1B, CASP1, FOSL1, MYC, MAP2K6, SPN, WWOX, RAB27A, CRTAM, LGALS12, BCL2L13, NLRP3, NLRP1, STK3, TNFRSF10B, TIAL1, LYST, RIPK2, UBB, BID, ING3, ADORA2A, ADAMTSL4, STK17B, RRAGA, ZBTB16, SCRIB, GCH1, PLEKHG2, PPP2CA, BCL3, PLAGL2, B4GALT1, CFLAR, CEBPB, KLF10, CEBPG, SMAD3, NR4A1, RPS6, TNFSF8, TNFSF10, RNF7, DUSP1, EEF1E1, PDCD6	6.52E-03
GO:0012501~programmed cell death	7.28E-05	ITGB3BP, MEF2C, NFKB1, PMAIP1, PTEN, PDCD2, ARHGAP4, CASP5, CASP6, TMEM173, CASP4, GSN, CDKN2D, AEN, TICAM1, IL1B, CASP1, MYC, IL1A, LUC7L3, CYCS, BCL2A1, LGALS12, PIM2, NLRP3, BCL2L13, NLRP1, STK3, AHR, OSM, TNFRSF10B, BNIP2, TIAL1, F3, TNFAIP8, RIPK2, CDK11B, UBB, SIAH2, GADD45B, TNFAIP3, PPP1R15A, TRAF1, BID, MCL1, ADORA2A, ADAMTSL4, CLU, STK17B, RRAGA, NFKBIA, PPT1, ZBTB16, SCRIB, TSC22D3, PLEKHG2, ZC3H12A, XAF1, THBS1, SRGN, AXIN1, CFLAR, IL6, DNM1L, PDCD10, AIMP1, SMAD3, FAM188A, SOD2, TNFSF10, P2RX1, IFT57, CSRNP1, CYFIP2, NLRP12, BRE, EAF2, PDCD6	6.72E-03
GO:0006915~apoptosis	7.70E-05	ITGB3BP, MEF2C, NFKB1, PMAIP1, PTEN, PDCD2,	6.93E-03

		ARHGAP4, CASP5, CASP6, TMEM173, CASP4, GSN, AEN, TICAM1, IL1B, CASP1, MYC, IL1A, LUC7L3, CYCS, BCL2A1, LGALS12, PIM2, NLRP3, BCL2L13, NLRP1, STK3, AHR, OSM, TNFRSF10B, BNIP2, TIAL1, F3, TNFAIP8, RIPK2, CDK11B, UBB, SIAH2, GADD45B, TNFAIP3, PPP1R15A, TRAF1, BID, MCL1, ADORA2A, ADAMTSL4, CLU, STK17B, RRAGA, NFKBIA, PPT1, ZBTB16, SCRIB, TSC22D3, PLEKHG2, ZC3H12A, XAF1, THBS1, SRGN, AXIN1, CFLAR, IL6, DNM1L, PDCD10, AIMP1, SMAD3, FAM188A, SOD2, TNFSF10, P2RX1, IFT57, CSRNP1, CYFIP2, NLRP12, BRE, EAF2, PDCD6	
GO:0032760~positive regulation of tumor necrosis factor production	8.43E-05	NOD2, CARD9, MYD88, TICAM1, TLR2, RIPK2, PF4, CLEC7A	7.39E-03
GO:0043065~positive regulation of apoptosis	9.20E-05	ITGB3BP, PTGS2, PDIA3, TLR2, TNFSF14, PMAIP1, PTEN, BTK, ARHGAP4, CASP6, CASP4, CD44, AEN, TICAM1, IL1B, CASP1, FOSL1, MYC, MAP2K6, SPN, WWOX, RAB27A, CRTAM, LGALS12, BCL2L13, NLRP3, NLRP1, STK3, TNFRSF10B, TIAL1, LYST, RIPK2, UBB, BID, ING3, ADORA2A, ADAMTSL4, STK17B, ZBTB16, SCRIB, GCH1, PLEKHG2, PPP2CA, BCL3, PLAGL2, B4GALT1, CFLAR, CEBPB, KLF10, CEBPG, SMAD3, NR4A1, RPS6, TNFSF8, TNFSF10, RNF7, DUSP1, EEF1E1, PDCD6	7.87E-03
GO:0043068~positive	1.12E-04	ITGB3BP, PTGS2, PDIA3,	9.32E-03

regulation of programmed cell death		TLR2, TNFSF14, PMAIP1, PTEN, BTK, ARHGAP4, CASP6, CASP4, CD44, AEN, TICAM1, IL1B, CASP1, FOSL1, MYC, MAP2K6, SPN, WWOX, RAB27A, CRTAM, LGALS12, BCL2L13, NLRP3, NLRP1, STK3, TNFRSF10B, TIAL1, LYST, RIPK2, UBB, BID, ING3, ADORA2A, ADAMTSL4, STK17B, ZBTB16, SCRIB, GCH1, PLEKHG2, PPP2CA, BCL3, PLAGL2, B4GALT1, CFLAR, CEBPB, KLF10, CEBPG, SMAD3, NR4A1, RPS6, TNFSF8, TNFSF10, RNF7, DUSP1, EEF1E1, PDCD6	
GO:0016265~death	1.38E-04	ITGB3BP, MEF2C, SPG7, NFKB1, PMAIP1, PTEN, PDCD2, CASP5, ARHGAP4, CASP6, TMEM173, CASP4, ATXN10, GSN, CDKN2D, AEN, TICAM1, IL1B, CASP1, MYC, IL1A, LUC7L3, CYCS, BCL2A1, LYZ, LGALS12, PIM2, NLRP3, BCL2L13, NLRP1, STK3, AHR, OSM, TNFRSF10B, BNIP2, TIAL1, F3, TNFAIP8, RIPK2, CDK11B, UBB, SIAH2, GADD45B, TNFAIP3, PPP1R15A, TRAF1, FUS, BID, MCL1, ADORA2A, ADAMTSL4, KIAA0196, CLU, STK17B, RRAGA, NFKBIA, PPT1, ZBTB16, HPRT1, SCRIB, TSC22D3, PLEKHG2, TMEM123, ZC3H12A, XAF1, THBS1, SRGN, AXIN1, CFLAR, IL6, DNM1L, PDCD10, OLR1, AIMP1, NR4A2, SMAD3, FAM188A, SOD2, ATXN1, TNFSF10, P2RX1, IFT57, CSRNP1, CYFIP2, BRE, NLRP12, EAF2, PDCD6	1.12E-02

GO:0050900~leukocyte migration	1.45E-04	B4GALT1, ICAM1, IL6, IL16, IL8, AIMP1, CXCL3, S100A9, PF4, AZU1, CORO1A, LYST, CKLF, IL1B, MSN	1.16E-02
GO:0002443~leukocyte mediated immunity	1.63E-04	XRCC4, ICAM1, CRTAM, YWHAZ, NBN, IL6, CEBPG, CLU, SERPING1, PRKCD, CD55, NOD2, LAT2, MYD88, LYST, C1RL, BCL3, RAB27A, HLA-DRA	1.27E-02
GO:0052547~regulation of peptidase activity	1.63E-04	HERPUD1, ADORA2A, CYCS, SNCA, SMAD3, NR4A1, PMAIP1, NLRP3, BCL2L13, NLRP1, TNFRSF10B, P2RX1, F3, IFT57, CDKN2D, NLRP12, CSTA, HSPA5, MYC	1.27E-02
GO:0043281~regulation of caspase activity	1.72E-04	HERPUD1, ADORA2A, CYCS, SNCA, SMAD3, NR4A1, PMAIP1, NLRP3, BCL2L13, NLRP1, TNFRSF10B, P2RX1, F3, IFT57, CDKN2D, NLRP12, HSPA5, MYC	1.31E-02
GO:0008219~cell death	1.77E-04	ITGB3BP, MEF2C, SPG7, NFKB1, PMAIP1, PTEN, PDCD2, CASP5, ARHGAP4, CASP6, TMEM173, CASP4, ATXN10, GSN, CDKN2D, AEN, TICAM1, IL1B, CASP1, MYC, IL1A, LUC7L3, CYCS, BCL2A1, LYZ, LGALS12, PIM2, NLRP3, BCL2L13, NLRP1, STK3, AHR, OSM, TNFRSF10B, BNIP2, TIAL1, F3, TNFAIP8, RIPK2, CDK11B, UBB, SIAH2, GADD45B, TNFAIP3, PPP1R15A, TRAF1, FUS, BID, MCL1, ADORA2A, ADAMTSL4, KIAA0196, CLU, STK17B, RRAGA, NFKBIA, PPT1, ZBTB16, HPRT1, SCRIB, TSC22D3, PLEKHG2, TMEM123, ZC3H12A, XAF1, THBS1, SRGN, AXIN1, CFLAR, IL6, DNM1L, PDCD10, OLR1,	1.31E-02

		AIMP1, SMAD3, FAM188A, SOD2, ATXN1, TNFSF10, P2RX1, IFT57, CSRNP1, CYFIP2, BRE, NLRP12, EAF2, PDCD6	
GO:0010608~posttranscriptional regulation of gene expression	2.10E-04	SRP14, NCBP1, RBM3, EIF2A, PTEN, MTIF3, LUZP6, ZFP36L1, ASGR2, ZFP36L2, EIF3H, EIF3E, HNRNPD, QKI, BCL3, PABPC1, THBS1, ZFP36, GTPBP4, IL6, CCDC88C, MAGOH, SMAD3, EIF1B, RPS4X, PRKCD, EIF4B, NCK2, EIF4E, EIF2S1, NGDN, PPP1R15B, PPP1R15A, SRP9	1.53E-02
GO:0002221~pattern recognition receptor signaling pathway	2.20E-04	IRAK2, NOD2, MYD88, TICAM1, TLR2, NFKBIA, RIPK2, CLEC7A	1.57E-02
GO:0016072~rRNA metabolic process	2.35E-04	RPL35A, EXOSC8, EXOSC9, LSM6, EXOSC5, RPL26, EXOSC3, WBP11, MKI67IP, RPS6, FCF1, RPF1, RPL7, RPS16, RPS14, WDR12, NOP58, RPL5, NSA2, RPS24	1.64E-02
GO:0042035~regulation of cytokine biosynthetic process	2.50E-04	FCER1A, IL6, CARD9, CEBPB, CEBPG, MAP2K3, AZU1, CD80, EREG, TICAM1, NLRP12, IRF1, BCL3, IL1B, GHRL, IL1A, SPN	1.71E-02
GO:0000398~nuclear mRNA splicing, via spliceosome	2.57E-04	FUS, NCBP1, RPL36A, POLR2E, TRA2B, SNRPB2, CWC15, TRA2A, NAA38, HNRNPL, HNRNPM, HNRNPK, HNRNPF, PCBP1, HNRNPD, U2AF1, HNRNPC, SNRPA1, SF3B14, MAGOH, HNRNPA2B1, PRPF3, HNRNPA0, HNRNPH3, HNRNPH2, SLU7, CPSF3, SNRPG	1.72E-02
GO:0000375~RNA splicing, via transesterification reactions	2.57E-04	FUS, NCBP1, RPL36A, POLR2E, TRA2B, SNRPB2, CWC15, TRA2A, NAA38, HNRNPL, HNRNPM, HNRNPK, HNRNPF, PCBP1,	1.72E-02

		HNRNPD, U2AF1, HNRNPC, SNRPA1, SF3B14, MAGOH, HNRNPA2B1, PRPF3, HNRNPA0, HNRNPH3, HNRNPH2, SLU7, CPSF3, SNRPG	
GO:0000377~RNA splicing, via transesterification reactions with bulged adenosine as nucleophile	2.57E-04	FUS, NCBP1, RPL36A, POLR2E, TRA2B, SNRPB2, CWC15, TRA2A, NAA38, HNRNPL, HNRNPM, HNRNPK, HNRNPF, PCBP1, HNRNPD, U2AF1, HNRNPC, SNRPA1, SF3B14, MAGOH, HNRNPA2B1, PRPF3, HNRNPA0, HNRNPH3, HNRNPH2, SLU7, CPSF3, SNRPG	1.72E-02
GO:0043123~positive regulation of I-kappaB kinase/NF-kappaB cascade	2.70E-04	SECTM1, CFLAR, CARD9, SLC44A2, VAPA, TFG, PIM2, TAB2, LGALS9, APOL3, TNFSF10, NOD2, TNFRSF10B, MYD88, MAP3K3, TICAM1, RIPK2, IL1B, CASP1, PPP5C	1.78E-02
GO:0052548~regulation of endopeptidase activity	2.77E-04	HERPUD1, ADORA2A, CYCS, SNCA, SMAD3, NR4A1, PMAIP1, NLRP3, BCL2L13, NLRP1, TNFRSF10B, P2RX1, F3, IFT57, CDKN2D, NLRP12, HSPA5, MYC	1.79E-02
GO:0006364~rRNA processing	3.93E-04	RPL35A, EXOSC8, EXOSC9, LSM6, EXOSC5, RPL26, EXOSC3, WBP11, RPS6, FCF1, RPF1, RPL7, RPS16, RPS14, WDR12, NOP58, RPL5, NSA2, RPS24	2.48E-02
GO:0032496~response to lipopolysaccharide	4.03E-04	PTGS2, SOCS3, SNCA, NFKBIA, PRDX2, COMT, PRDX3, TRIB1, GCH1, NOD2, MYD88, TICAM1, RIPK2, IL1B, SERPINA1, IRG1, AKIRIN2	2.50E-02
GO:0002449~lymphocyte mediated immunity	4.28E-04	XRCC4, ICAM1, CRTAM, NBN, CEBPG, CLU, SERPING1, PRKCD, CD55, NOD2, MYD88, LYST, C1RL, BCL3, RAB27A,	2.61E-02

		HLA-DRA	
GO:0010033~response to organic substance	4.92E-04	AQP9, LDLR, PTGS2, RBM3, SNCA, TLR2, RHOQ, AP3S1, PRDX2, PMAIP1, PRDX3, UQCRFS1, PTEN, B2M, GNG8, CD48, CASP6, NOD2, MYD88, NDUFS4, CD44, GSN, TICAM1, IL1B, RARA, GNG2, SERPINA1, CASP1, MYC, FOSL1, AKIRIN2, EGR1, IRAK2, SOCS3, FBP1, JUNB, RETN, CD83, BTG2, GNB2, NCOA6, GHRL, RIPK2, PPP1R15A, PPP5C, HMGB2, MCL1, ADORA2A, CALCOCO2, NFKBIA, GNG11, COMT, HPRT1, TRIB1, GCH1, ACSL1, PPP2CA, SH2B2, TAF9, THBS1, IRG1, SDF4, HERPUD1, IL6, CARD9, PTPN2, SELL, KLF10, CREB1, IL1RN, BCKDHB, NR4A2, SMAD3, ANXA5, PTPN11, CORO1A, P2RX1, DUSP1, ID2, FYN, PRKAR1B, ALDH2, CLEC7A, RBM14, HDAC9	2.94E-02
GO:0002758~innate immune response-activating signal transduction	4.94E-04	IRAK2, NOD2, MYD88, TICAM1, TLR2, NFKBIA, RIPK2, CLEC7A	2.90E-02
GO:0032755~positive regulation of interleukin-6 production	4.94E-04	NOD2, IL6, CARD9, TICAM1, TLR2, IL1B, RIPK2, AKIRIN2	2.90E-02
GO:0002218~activation of innate immune response	4.94E-04	IRAK2, NOD2, MYD88, TICAM1, TLR2, NFKBIA, RIPK2, CLEC7A	2.90E-02
GO:0034660~ncRNA metabolic process	4.96E-04	FARS2, LSM6, MKI67IP, WBP11, QTRT1, FCF1, RPL7, THG1L, WDR12, RPL5, KIAA0391, POLG2, DUS1L, TSEN15, NSA2, RPS24, DUS3L, RPL35A, EXOSC8, EXOSC9, AIMP1, EXOSC5, RPL26, EXOSC3, SSB, DARS2, RPS6, RPF1, TRNT1, RPS16, RPS14, FARSB, NOP58, CARS2,	2.87E-02

		POP5	
GO:0030595~leukocyte chemotaxis	5.65E-04	AZU1, CORO1A, IL6, IL8, IL16, LYST, CXCL3, CKLF, S100A9, IL1B, PF4	3.21E-02
GO:0034470~ncRNA processing	5.71E-04	FARS2, LSM6, WBP11, QTRT1, FCF1, RPL7, THG1L, WDR12, RPL5, KIAA0391, DUS1L, TSEN15, NSA2, RPS24, DUS3L, RPL35A, EXOSC8, EXOSC9, EXOSC5, RPL26, SMAD3, EXOSC3, SSB, RPS6, RPF1, TRNT1, RPS16, RPS14, NOP58, POP5	3.19E-02
GO:0051098~regulation of binding	6.13E-04	TLR2, NFKBIA, PRDX2, PRDX3, TRIB1, KDM1A, NOD2, MYD88, GTF2A2, TICAM1, IL1B, PEX14, BCL3, NRG1, APEX1, IRAK2, ICAM1, IL6, GTPBP4, CEBPG, SMAD3, NLRP3, PRKCD, ID2, RIPK2, CALM2	3.37E-02
GO:0042273~ribosomal large subunit biogenesis	6.35E-04	RPL35A, RPL7, RPL26, WDR12, RPL5, RPL24	3.44E-02
GO:0050778~positive regulation of immune response	6.46E-04	CLU, TLR2, NFKBIA, IL15, B2M, CFP, NOD2, MYD88, TICAM1, IL1B, SH2B2, IRAK2, FCER1A, CRTAM, CARD9, SERPING1, CD1D, LAT2, CD55, EREG, FYN, C1RL, RIPK2, CLEC7A, HLA-DRA	3.44E-02
GO:0031349~positive regulation of defense response	6.86E-04	IRAK2, FCER1A, CRTAM, IL6, CARD9, TLR2, NFKBIA, CD1D, OSM, NOD2, MYD88, EREG, TICAM1, NLRP12, RIPK2, CLEC7A	3.59E-02
GO:0051101~regulation of DNA binding	7.31E-04	IRAK2, ICAM1, IL6, CEBPG, TLR2, NFKBIA, PRDX2, PRDX3, NLRP3, TRIB1, KDM1A, NOD2, MYD88, ID2, TICAM1, GTF2A2, PEX14, RIPK2, BCL3, IL1B, APEX1, CALM2	3.77E-02
GO:0060326~cell chemotaxis	8.87E-04	AZU1, CORO1A, IL6, IL8, IL16, LYST, CXCL3, CKLF, S100A9, IL1B, PF4	4.49E-02

GO:0045089~positive regulation of innate immune response	9.38E-04	IRAK2, NOD2, CRTAM, CARD9, MYD88, EREG, TICAM1, TLR2, NFKBIA, RIPK2, CLEC7A, CD1D	4.67E-02

Biological Process (BP_FAT) associated with genes having decreased expression in **HNP_RM** with respect to HNP_Monocyte

Term	PValue	Genes	Benjamini
GO:0006414~translational elongation	3.04E-48	RPL18, RPL17, RPL19, RPL14, RPL13, RPL15, RPLP2, RPS2, RPS3, RPS3A, RPLP0, RPLP1, RPL10, FAU, RPL11, RPL12, RPS27A, RPL35A, EEF2, RPS4X, RPS19, RPS16, RPL41, RPS14, RPS15, UBC, RPS12, EEF1G, RPS13, RPS10, RPS11, UBB, EEF1D, UBA52, TUFM, EEF1B2, RPL27A, RPL35, RPS15A, RPL36, RPL37, RPL38, RPS25, RPS27, RPL30, RPS28, RPL7, RPL32, RPS29, RPL31, RPL6, RPL34, RPL9, RPL8, RPL3, RPL5, RPL10A, RPL7A, RPL4, RPS20, RPS21, RPS23, RPS24, EEF1A1, RPSA, RPL26, RPL27, RPS9, RPL24, RPL23A, RPS6, RPS5, SELT, RPS8, RPL28, RPL29, RPS7, HNRNPH2, RPL23, RPL18A, RPL13A, RPL22, RPL37A	1.38E-44
GO:0006412~translation	9.29E-39	EIF6, RPL18, MRPS36, RPL17, RPL19, MRPS33, RPL14, RPL13, RBM3, RPL15, EIF5, EIF5A, RPS6KB2, RPLP2, RPS27L, RPL22L1, RPLP0, RPLP1, EIF1AY, FAU, RPL10, EIF1, RPL11, RPL12, MRPL33, RPS27A, EIF2B5, RPL36AL, RPL35A, MRPL52, MRPL51, YARS, EIF2S3, EEF2, RPS19, MRPS18C, RPS16, MRPS18A, RPS14, RPS15, RPS12, RPS13, RPS10, MRPL47, MRPL48, RPS11, UBA52, EEF1B2, MRPS15, FARS2, PABPC4, MRPS11,	2.10E-35

		KARS, RPS25, EIF3D, RPS27, RPS28, EIF3B, RPS29, RPL7, EIF3G, EIF3H, RPL6, RPL9, EIF3E, RPL8, EIF3F, RPL3, EIF3K, EIF3L, MRPL55, EIF3I, RPL5, RPS20, RPL10A, RPL7A, RPL4, RPS21, EIF3M, RPS23, RPS24, EEF1A1, RPSA, MRPS24, GARS, RPS9, RPL23A, RPS6, RPS5, RPS8, RPS7, EIF4B, HNRNPH2, EIF4E, RPL18A, EIF4H, RPL37A, EIF4E2, QARS, EIF2A, RPS2, RPS3, WARS, RPS3A, TNIP1, SARS, MRPS6, EIF1B, RPS4X, GTF2B, EIF4G2, TARS, RPL41, EIF4A2, EIF4A1, UBC, EEF1G, DHPS, UBB, EEF1D, TUFM, NACA, ABTB1, RPL27A, RPL35, RPL36, RPS15A, RPL37, RPL38, MRPL20, CYLD, RPL30, RPL32, RPL31, MRPL14, RPL34, MRPL18, RSL24D1, MARS, RPL26, RPL27, RPL24, ETF1, SELT, RPL28, RPL29, MRPL23, MRPL21, MRPL28, RPL23, RPL22, RPL13A	
GO:0008380~RNA splicing	3.00E-24	RALY, NCBP1, U2AF2, SNRPD3, RBM4, LSM7, RBM5, SNRPD1, SNRPD2, WTAP, SART1, NONO, DHX38, TARDBP, SRRM2, DNAJC8, U2AF1, LSM4, LUC7L3, PABPN1, CLNS1A, SNRPA1, SF3B14, EFTUD2, MAGOH, HNRNPA2B1, PTBP1, HNRNPU, SNRPB, SNRPA, SLU7, CELF1, SNRPC, SNRPF, SNRPE, SNRPG, FUS, NHP2L1, STRAP, SNRPB2, ZCRB1, XAB2, NAA38, HNRNPA3, HNRNPL, HNRNPM,	4.52E-21

		HNRNPK, FRG1, HNRNPF, ISY1, HNRNPD, HNRNPC, PABPC1, DDX41, ARL6IP4, PRPF40A, DHX9, SNW1, RNPS1, DDX5, HNRNPA1, RBMX, U2AF1L4, HNRNPA0, HNRNPH3, HNRNPH2, HNRNPH1, PUF60, CWC15, YBX1, PLRG1, PCBP1, PCBP2, QKI, PPP2R1A, SF1, PRPF3, MBNL1, EIF4A3, SNRNP40, RBM39, THOC2, CPSF1, PRPF38A, POLR2G, POLR2F, PPP4R2, POLR2E, POLR2L, TRA2B, POLR2I, TRA2A, SF3B5, IVNS1ABP, SF3B2, PRPF19, SF3B1, CIR1, CD2BP2, PRPF8, PPP2CA, USP39, NUDT21, DHX15, SCNM1, SNRNP70, RBM22, BCAS2, PAPOLA, JMJD6, SFPQ, SYF2, ZRANB2, PHF5A, TXNL4A, RBM17	
GO:0006397~mRNA processing	2.78E-22	RALY, NCBP1, U2AF2, SNRPD3, RBM4, LSM7, RBM5, SNRPD1, SNRPD2, WTAP, SART1, NONO, DHX38, SRRM2, TARDBP, DNAJC8, U2AF1, LSM4, LUC7L3, PABPN1, CLNS1A, SNRPA1, SF3B14, EFTUD2, MAGOH, HNRNPA2B1, PTBP1, HNRNPU, SNRPB, SNRPA, CELF2, SLU7, CELF1, SNRPC, SNRPF, SNRPE, SNRPG, FUS, FIP1L1, NHP2L1, STRAP, SNRPB2, ZCRB1, XAB2, NAA38, HNRNPA3, HNRNPL, HNRNPM, MOV10, HNRNPK, FRG1, HNRNPF, ISY1, HNRNPD, HNRNPC, PABPC1, DDX41, PRPF40A, DHX9,	3.15E-19

		SNW1, RNPS1, DDX5, HNRNPA1, RBMX, U2AF1L4, HNRNPA0, HNRNPH3, HNRNPH2, POP4, HNRNPH1, PUF60, ADAR, CWC15, YBX1, PLRG1, PCBP1, PCBP2, QKI, ZFP36, SF1, PRPF3, MBNL1, EIF4A3, CPSF6, SNRNP40, RBM39, THOC2, CPSF1, SSU72, PRPF38A, POLR2G, POLR2F, PPP4R2, POLR2E, POLR2L, TRA2B, POLR2I, TRA2A, SF3B5, KIN, SF3B2, PRPF19, SF3B1, CIR1, CD2BP2, PRPF8, USP39, NUDT21, DHX15, SCNM1, SNRNP70, RBM22, BCAS2, RBM23, PAPOLA, JMJD6, SFPQ, SYF2, ZRANB2, PHF5A, TXNL4A, RBM17	
GO:0006119~oxidative phosphorylation	7.13E-22	ATP5D, UQCRC2, ATP5E, ATP6V0E1, UQCRC1, ATP6AP1, ATP5B, SNCA, NDUFAB1, NDUFS6, NDUFS5, UQCR11, NDUFS4, ATP5L, ATP5O, NDUFS3, ATP5I, ATP5H, NDUFS2, ATP5J, NDUFB10, TAZ, NDUFC2, ATP6V1H, NDUFC1, NDUFA10, ATP6V1F, UQCRH, ATP5C1, UQCRB, NDUFB3, NDUFB4, NDUFB5, NDUFB7, NDUFB8, NDUFB9, ATP5G2, ATP6V1B2, ATP5G1, SDHAF2, ATP5G3, NDUFB1, ATP6V0B, NDUFB2, ATP6V0C, NDUFA4, TCIRG1, NDUFA5, NDUFA2, NDUFA3, NDUFA9, NDUFA6, NDUFA7, ATP5F1, NDUFA1, ATP6V1E1, NDUFV1, ATP5A1	6.45E-19
GO:0006091~generation of	9.13E-22	LDHB, LDHA, OXA1L,	6.89E-19

precursor metabolites and energy		ATP6AP1, SNCA, NDUFAB1, IDH3G, UQCR11, PDHA1, TXNL1, SUCLG1, TAZ, PPP1CB, COX6C, PPP1CA, ATP5C1, ATPIF1, SURF1, MDH2, MDH1, ACAA1, ALDOA, TXN2, COX7C, ATP6V1B2, SDHAF2, ATP6V0B, ATP6V0C, TPI1, COX8A, IDH3B, NDUFV1, COX6A1, UQCRC2, ATP5D, ACOX1, ATP5E, ATP6V0E1, UQCRC1, ATP5B, CYC1, PRKAG2, UQCRFS1, UQCRQ, NDUFS6, NDUFS5, NDUFS4, SLC25A3, ATP5L, ATP5O, ATP5I, NDUFS3, NDUFS2, ATP5H, COX17, ATP5J, NDUFB11, NDUFB10, PFKL, PHKG2, CYCS, NDUFC2, ATP6V1H, NDUFA13, COX4I1, NDUFC1, NDUFA10, NDUFA12, NDUFA11, ATP6V1F, ACADVL, UQCRH, TXN, GAA, GNAS, UQCRB, NDUFB3, NDUFB4, NDUFB5, ECH1, NDUFB7, ADPGK, NDUFB8, NDUFB9, HK2, HK1, ATP5G2, ATP5G1, ATP5G3, NDUFB1, NDUFB2, HK3, GAPDH, ETFB, GLRX, ETFA, ENO1, NDUFA4, TCIRG1, NDUFA5, NDUFA2, NDUFA3, NDUFA9, NDUFA6, NDUFA7, ATP5F1, NDUFA1, SOD2, SDHA, CYBA, SDHB, GPI, CYBB, PYGL, GSK3B, SDHC, ATP6V1E1, ATP5A1, PGK1	
GO:0016071~mRNA metabolic process	3.30E-21	RALY, NCBP1, U2AF2, SNRPD3, LSM7, RBM4, RBM5, SNRPD1, SNRPD2, WTAP, SART1, NONO,	2.13E-18

		DHX38, DNAJB11, SRRM2, WIBG, TARDBP, DNAJC8, U2AF1, LSM4, LUC7L3, PABPN1, CLNS1A, SNRPA1, SF3B14, EFTUD2, MAGOH, HNRNPA2B1, PTBP1, HNRNPU, VEGFA, SNRPB, SNRPA, CELF2, SLU7, CELF1, SNRPC, SNRPF, SNRPE, SNRPG, FUS, FIP1L1, NHP2L1, STRAP, SNRPB2, ZCRB1, MAPKAPK2, NAA38, XAB2, HNRNPA3, HNRNPL, HNRNPM, SLC11A1, MOV10, HNRNPK, FRG1, EIF3E, HNRNPF, ISY1, HNRNPD, PABPC3, HNRNPC, PABPC1, DDX41, PRPF40A, DHX9, SNW1, RNPS1, DDX5, HNRNPA1, RBMX, U2AF1L4, HNRNPA0, HNRNPH3, HNRNPH2, DCP2, POP4, HNRNPH1, PUF60, ADAR, CWC15, YBX1, PLRG1, PCBP1, PCBP2, QKI, ZFP36, SF1, PRPF3, MBNL1, EIF4A3, CPSF6, SNRNP40, RBM39, THOC2, CPSF1, SSU72, PRPF38A, POLR2G, POLR2F, PPP4R2, POLR2E, POLR2L, TRA2B, POLR2I, TRA2A, SF3B5, KIN, SF3B2, ZFP36L1, EXOSC10, PRPF19, SF3B1, CIR1, CD2BP2, PRPF8, USP39, NUDT21, DHX15, SCNM1, SNRNP70, BCAS2, RBM22, RBM23, PAPOLA, JMJD6, SFPQ, SYF2, ZRANB2, PHF5A, TXNL4A, RBM17	
GO:0000398~nuclear mRNA splicing, via spliceosome	9.66E-21	NCBP1, SNRPD3, U2AF2, CWC15, LSM7, RBM5, SNRPD1, SNRPD2, YBX1, DHX38, PCBP1, DNAJC8,	5.46E-18

		PCBP2, U2AF1, PABPN1, CLNS1A, SNRPA1, SF3B14, EFTUD2, MAGOH, PTBP1, HNRNPA2B1, SF1, PRPF3, MBNL1, HNRNPU, SNRPB, SNRPA, SLU7, CELF1, SNRNP40, SNRPC, SNRPF, SNRPE, CPSF1, SNRPG, FUS, POLR2G, POLR2F, POLR2E, POLR2L, NHP2L1, TRA2B, POLR2I, TRA2A, SNRPB2, SF3B5, NAA38, SF3B2, HNRNPL, HNRNPA3, HNRNPM, SF3B1, HNRNPK, CD2BP2, HNRNPF, PRPF8, USP39, NUDT21, HNRNPD, HNRNPC, SNRNP70, DHX9, SNW1, RNPS1, HNRNPA1, RBMX, HNRNPA0, PAPOLA, HNRNPH3, HNRNPH2, PHF5A, HNRNPH1, TXNL4A	
GO:0000375~RNA splicing, via transesterification reactions	9.66E-21	NCBP1, SNRPD3, U2AF2, CWC15, LSM7, RBM5, SNRPD1, SNRPD2, YBX1, DHX38, PCBP1, DNAJC8, PCBP2, U2AF1, PABPN1, CLNS1A, SNRPA1, SF3B14, EFTUD2, MAGOH, PTBP1, HNRNPA2B1, SF1, PRPF3, MBNL1, HNRNPU, SNRPB, SNRPA, SLU7, CELF1, SNRNP40, SNRPC, SNRPF, SNRPE, CPSF1, SNRPG, FUS, POLR2G, POLR2F, POLR2E, POLR2L, NHP2L1, TRA2B, POLR2I, TRA2A, SNRPB2, SF3B5, NAA38, SF3B2, HNRNPL, HNRNPA3, HNRNPM, SF3B1, HNRNPK, CD2BP2, HNRNPF, PRPF8, USP39, NUDT21, HNRNPD, HNRNPC, SNRNP70,	5.46E-18

		DHX9, SNW1, RNPS1, HNRNPA1, RBMX, HNRNPA0, PAPOLA, HNRNPH3, HNRNPH2, PHF5A, HNRNPH1, TXNL4A	
GO:0000377~RNA splicing, via transesterification reactions with bulged adenosine as nucleophile	9.66E-21	NCBP1, SNRPD3, U2AF2, CWC15, LSM7, RBM5, SNRPD1, SNRPD2, YBX1, DHX38, PCBP1, DNAJC8, PCBP2, U2AF1, PABPN1, CLNS1A, SNRPA1, SF3B14, EFTUD2, MAGOH, PTBP1, HNRNPA2B1, SF1, PRPF3, MBNL1, HNRNPU, SNRPB, SNRPA, SLU7, CELF1, SNRNP40, SNRPC, SNRPF, SNRPE, CPSF1, SNRPG, FUS, POLR2G, POLR2F, POLR2E, POLR2L, NHP2L1, TRA2B, POLR2I, TRA2A, SNRPB2, SF3B5, NAA38, SF3B2, HNRNPL, HNRNPA3, HNRNPM, SF3B1, HNRNPK, CD2BP2, HNRNPF, PRPF8, USP39, NUDT21, HNRNPD, HNRNPC, SNRNP70, DHX9, SNW1, RNPS1, HNRNPA1, RBMX, HNRNPA0, PAPOLA, HNRNPH3, HNRNPH2, PHF5A, HNRNPH1, TXNL4A	5.46E-18
GO:0006396~RNA processing	2.73E-20	RALY, NCBP1, NAF1, RPL14, RBM3, SNRPD3, U2AF2, LSM7, RBM4, RBM5, SNRPD1, RBM6, SNRPD2, WTAP, SART1, NONO, DDX17, DHX38, SRRM2, TARDBP, DNAJC8, U2AF1, LSM4, RPL11, IMP4, LUC7L3, CLNS1A, PABPN1, SNRPA1, RPL35A, SF3B14, MAGOH, EFTUD2, HNRNPA2B1, PTBP1, HNRNPU, RPS19, RPS16,	1.37E-17

		RPS14, RPS15, SNRPB, SNRPA, SLU7, CELF2, CELF1, SNRPC, SNRPF, SNRPE, SNRPG, FUS, CPSF3L, HSD17B10, FIP1L1, STRAP, NHP2L1, FARS2, PABPC4, SNRPB2, ZCRB1, NAA38, XAB2, HNRNPA3, HNRNPL, HNRNPM, MOV10, RPS28, HNRNPK, RPL7, FRG1, HNRNPF, ISY1, HNRNPD, RPL5, HNRNPC, PABPC1, RPL10A, DDX41, ARL6IP4, RPS24, PRPF40A, DHX9, SMAD3, SNW1, RNPS1, INTS10, RPS6, DDX5, NOP10, U2AF1L4, RBMX, HNRNPA1, HNRNPA0, RPS7, URM1, DDX56, HNRNPH3, HNRNPH2, POP4, HNRNPH1, PUF60, ADAR, CWC15, YBX1, DKC1, PLRG1, PCBP1, PCBP2, QKI, FTSJ1, DUS1L, DEDD2, ZFP36, PPP2R1A, EXOSC8, SARS, SF1, PRPF3, MBNL1, EXOSC1, EIF4A3, CPSF6, SNRNP40, RBM39, THOC2, CPSF1, SSU72, PRPF38A, POLR2G, POLR2F, PPP4R2, POLR2E, POLR2L, TRA2B, POLR2I, TRA2A, UTP6, SF3B5, IVNS1ABP, KIN, SF3B2, PRPF19, EXOSC10, SF3B1, SRRT, CIR1, CD2BP2, PPP2CA, PRPF8, USP39, NUDT21, DHX15, SCNM1, KIAA0391, SNRNP70, NSA2, BCAS2, RBM22, RBM23, RPL26, FBL, ATXN1, PAPOLA, JMJD6, SFPQ, SYF2, ZRANB2, NOP58, PHF5A, NOP56, RBM17, TXNL4A	
GO:0042981~regulation of apoptosis	3.76E-17	MEF2C, XRCC5, HRAS, PTGS2, STAT5A, SNCA,	1.70E-14

		SART1, CITED2, CUL3, MYD88, CD44, ILK, RPL11, PSENEN, PIM1, PIM3, POLB, PIM2, BCL2L13, BCL2L11, F3, VEGFA, MYO18A, SIVA1, STK17B, NFKBIA, AKAP13, PPT1, CALR, ADA, CD74, SLC11A2, PEA15, RAC1, DIABLO, ABR, TM2D1, BECN1, KLF10, LGALS1, SPHK1, SMAD3, SLAMF7, RPS6, TAX1BP1, RNF7, RTN4, PDIA3, NFKB1, PTEN, DYNLL1, BAG1, TICAM1, CHST11, DEDD2, CLN3, ARHGEF2, NDUFA13, SERPINB9, TNFRSF10B, PSEN1, CARD16, BNIP2, CARD17, SERPINB2, MAP3K11, BID, CSF2, YWHAZ, PML, ITM2B, GCH1, PLEKHG2, SQSTM1, PPP2CA, PPP3CC, BCL3, BCL6, THBS1, PHLDA1, IL6, YWHAB, BIRC6, STAT1, BIRC3, SOD1, YWHAE, BIRC2, SOD2, DUSP1, GSK3B, IFT57, BNIP3L, DNM2, RBM5, TLR2, TNFSF14, EIF5A, TNFSF13, RPS27L, PMAIP1, TGFB1, IL10, CRADD, CTNNBL1, CDKN2D, TPT1, IL1B, DAP, MX1, RPS27A, IL1A, SOCS3, MADD, RELA, BCL2A1, ACTN1, DDIT3, LYST, TIAL1, TNFAIP8, CSTB, RIPK2, TNFAIP3, UBA52, TRAF1, CCL2, MCL1, ADORA2A, CLU, SRC, BLOC1S2, MTCH1, NPM1, HSPE1, TAF9, RUNX3, TRAF3, B4GALT1, CFLAR, HERPUD1, CARD8, NR4A1, VAV1, PPIF,	

		TXNDC12, P2RX4, ATF5, CDKN1A, TNFSF10, HDAC3, VCP, HDAC1, IKBKB, PDCD5, PDCD6, DNAJB6, IFI6, HDAC6, PRDX5, PRDX2, PRDX1, RPS3, ARHGAP4, CASP4, RPS3A, CASP8, RHOA, NDUFS3, CASP1, NRG1, FOSL1, IRAK1, PPP2R1A, CYCS, IFI16, PRKCE, NLRP3, CDK5, NLRP1, TNFRSF9, ADRB2, TNFSF13B, BTG2, BTG1, CFL1, UBC, UBB, PRNP, GSTP1, BCLAF1, APH1A, MGMT, PF4, NR3C1, TCF7L2, SH3GLB1, DAD1, PYCARD, HSPA5, ERCC1, HSPA9, PTPRC, CEBPB, TMBIM6, ANXA1, SAP30BP, BAD, ANXA5, TNFSF8, HSP90B1, SON, PSMG2, NME1-NME2, PRLR, BAX, SMPD2	
GO:0051248~negative regulation of protein metabolic process	1.36E-16	HSP90AB1, SRP14, SNCA, NFKB1, TGFB1, IL10, EIF4EBP1, PSMD1, PSMD2, PSMD4, PSMD6, PSMD7, PSMD8, DNAJC1, RPS27A, EIF2B5, PSMD9, CLN3, PPP2R1A, GTPBP4, ANAPC5, RELA, NDUFA13, PRKCD, CDK5, FLNA, PSMA2, PSMA1, EIF4A3, EIF2AK1, PSMA6, PSME1, PSEN1, PSMA5, PSME2, PSMA4, PSMA3, UBC, UBB, UBA52, PSMB10, PML, ANAPC10, FKBP1A, ANAPC11, CALR, PSMA7, TIMP1, PSMB4, PSMF1, PSMB7, SET, PSMB6, PSMB1, EIF3E, PPP2CA, PSMB2, TAF9, THBS1, UBE2D1, TINF2, PTPRC, PAIP2, TAF7, YWHAB, ANKHD1, YWHAE, PSMC5, PSMD13, PSMC4, PSMC3,	4.57E-14

		PSMD11, PSMC2, BAX, VPS28, HDAC6, UBE2E1	
GO:0043067~regulation of programmed cell death	1.71E-16	MEF2C, XRCC5, HRAS, PTGS2, STAT5A, SNCA, SART1, CITED2, CUL3, MYD88, CD44, ILK, RPL11, PSENEN, PIM1, PIM3, POLB, PIM2, BCL2L13, BCL2L11, F3, VEGFA, MYO18A, SIVA1, STK17B, NFKBIA, AKAP13, PPT1, CALR, ADA, CD74, SLC11A2, PEA15, RAC1, DIABLO, ABR, TM2D1, BECN1, KLF10, LGALS1, SPHK1, SMAD3, SLAMF7, RPS6, TAX1BP1, RNF7, RTN4, PDIA3, NFKB1, PTEN, DYNLL1, BAG1, TICAM1, CHST11, DEDD2, CLN3, ARHGEF2, NDUFA13, SERPINB9, TNFRSF10B, PSEN1, CARD16, BNIP2, CARD17, SERPINB2, MAP3K11, BID, CSF2, YWHAZ, PML, ITM2B, GCH1, PLEKHG2, SQSTM1, PPP2CA, PPP3CC, BCL3, BCL6, THBS1, PHLDA1, IL6, YWHAB, BIRC6, STAT1, BIRC3, SOD1, YWHAE, BIRC2, SOD2, DUSP1, GSK3B, IFT57, BNIP3L, DNM2, RBM5, TLR2, TNFSF14, EIF5A, TNFSF13, RPS27L, PMAIP1, TGFB1, IL10, CRADD, CTNNBL1, CDKN2D, TPT1, IL1B, DAP, MX1, RPS27A, IL1A, SOCS3, MADD, RELA, BCL2A1, ACTN1, DDIT3, LYST, TIAL1, TNFAIP8, CSTB, RIPK2, TNFAIP3, UBA52, TRAF1, CCL2, MCL1, ADORA2A, CLU, SRC, BLOC1S2, MTCH1, NPM1, HSPE1, TAF9,	8.37E-14

		RUNX3, TRAF3, B4GALT1, CFLAR, HERPUD1, CARD8, NR4A1, VAV1, PPIF, TXNDC12, P2RX4, ATF5, CDKN1A, TNFSF10, HDAC3, PSMC5, VCP, HDAC1, IKBKB, PDCD5, PDCD6, DNAJB6, IFI6, HDAC6, PRDX5, PRDX2, PRDX1, RPS3, ARHGAP4, CASP4, RPS3A, CASP8, RHOA, NDUFS3, CASP1, NRG1, FOSL1, IRAK1, PPP2R1A, CYCS, IFI16, PRKCE, NLRP3, CDK5, NLRP1, TNFRSF9, ADRB2, TNFSF13B, BTG2, BTG1, CFL1, UBC, UBB, PRNP, GSTP1, BCLAF1, APH1A, MGMT, PF4, NR3C1, TCF7L2, SH3GLB1, DAD1, PYCARD, HSPA5, ERCC1, HSPA9, PTPRC, CEBPB, TMBIM6, ANXA1, SAP30BP, BAD, ANXA5, TNFSF8, HSP90B1, SON, PSMG2, NME1-NME2, PRLR, BAX, SMPD2	
GO:0010941~regulation of cell death	2.04E-16	MEF2C, XRCC5, HRAS, PTGS2, STAT5A, SNCA, SART1, CITED2, CUL3, MYD88, CD44, ILK, RPL11, PSENEN, PIM1, PIM3, POLB, PIM2, BCL2L13, BCL2L11, F3, VEGFA, MYO18A, SIVA1, STK17B, NFKBIA, AKAP13, PPT1, CALR, ADA, CD74, SLC11A2, PEA15, RAC1, DIABLO, ABR, TM2D1, BECN1, KLF10, LGALS1, SPHK1, SMAD3, SLAMF7, RPS6, TAX1BP1, RNF7, RTN4, PDIA3, NFKB1, PTEN, DYNLL1, BAG1, TICAM1, CHST11, DEDD2, CLN3, ARHGEF2, NDUFA13, SERPINB9, TNFRSF10B,	7.73E-14

		PSEN1, CARD16, BNIP2, CARD17, SERPINB2, MAP3K11, BID, CSF2, YWHAZ, PML, ITM2B, GCH1, PLEKHG2, SQSTM1, PPP2CA, PPP3CC, BCL3, BCL6, THBS1, PHLDA1, IL6, YWHAB, BIRC6, STAT1, BIRC3, SOD1, YWHAE, BIRC2, SOD2, DUSP1, GSK3B, IFT57, BNIP3L, DNM2, RBM5, TLR2, TNFSF14, EIF5A, TNFSF13, RPS27L, PMAIP1, TGFB1, IL10, CRADD, CTNNBL1, CDKN2D, TPT1, IL1B, DAP, MX1, RPS27A, IL1A, SOCS3, MADD, RELA, BCL2A1, ACTN1, DDIT3, LYST, TIAL1, TNFAIP8, CSTB, RIPK2, TNFAIP3, UBA52, TRAF1, CCL2, MCL1, ADORA2A, CLU, SRC, BLOC1S2, MTCH1, NPM1, HSPE1, TAF9, RUNX3, TRAF3, B4GALT1, CFLAR, HERPUD1, CARD8, NR4A1, VAV1, PPIF, TXNDC12, P2RX4, ATF5, CDKN1A, TNFSF10, HDAC3, PSMC5, VCP, HDAC1, IKBKB, PDCD5, PDCD6, DNAJB6, IFI6, HDAC6, PRDX5, PRDX2, PRDX1, RPS3, ARHGAP4, CASP4, RPS3A, CASP8, RHOA, NDUFS3, CASP1, NRG1, FOSL1, IRAK1, PPP2R1A, CYCS, IFI16, PRKCE, NLRP3, CDK5, NLRP1, TNFRSF9, ADRB2, TNFSF13B, BTG2, BTG1, CFL1, UBC, UBB, PRNP, GSTP1, BCLAF1, APH1A, MGMT, PF4, NR3C1, TCF7L2, SH3GLB1, DAD1, PYCARD, HSPA5, ERCC1,	

		HSPA9, PTPRC, CEBPB, TMBIM6, ANXA1, SAP30BP, BAD, ANXA5, TNFSF8, HSP90B1, SON, PSMG2, NME1-NME2, PRLR, BAX, SMPD2	
GO:0032269~negative regulation of cellular protein metabolic process	2.73E-16	HSP90AB1, SRP14, SNCA, NFKB1, TGFB1, IL10, EIF4EBP1, PSMD1, PSMD2, PSMD4, PSMD6, PSMD7, PSMD8, DNAJC1, RPS27A, EIF2B5, PSMD9, CLN3, PPP2R1A, GTPBP4, ANAPC5, NDUFA13, PRKCD, CDK5, PSMA2, PSMA1, EIF4A3, EIF2AK1, PSMA6, PSME1, PSEN1, PSMA5, PSME2, PSMA4, PSMA3, UBC, UBB, UBA52, PSMB10, PML, ANAPC10, FKBP1A, ANAPC11, CALR, PSMA7, TIMP1, PSMB4, PSMF1, PSMB7, SET, PSMB6, PSMB1, EIF3E, PPP2CA, PSMB2, TAF9, UBE2D1, THBS1, TINF2, PTPRC, PAIP2, TAF7, YWHAB, ANKHD1, YWHAE, PSMC5, PSMD13, PSMC4, PSMC3, PSMD11, PSMC2, BAX, VPS28, HDAC6, UBE2E1	7.18E-14
GO:0006955~immune response	9.45E-16	IL16, AQP9, SNCA, TLR2, TNFSF14, TNFSF13, IL15, PNP, TGFB1, IL10, CTNNBL1, B2M, CXCL10, CFP, MYD88, LILRA2, CLEC4E, LILRA4, LILRA5, LILRA6, CLEC4A, IL1B, CLEC4D, AKIRIN2, IL1A, DBNL, GTPBP1, GBP5, C5AR1, NCF2, BST2, BST1, NCF1, NCF4, RELA, RELB, HLA-A, HLA-C, TNFRSF14, HLA-DQA1, BCAP31, IGSF6, LILRB1, LILRB2, CCR7, LAT2, PPBP, CCR5, CST7, LYST, LILRB3, LILRB4, VEGFA,	3.02E-13

		GBP4, CLEC5A, CD300LB, GBP2, GBP1, CCL2, HLA-DRB1, IFITM2, IFITM3, TFE3, CLU, OAS1, FCGRT, PF4V1, FTH1, CD74, CCL7, ADA, IFI35, SLC11A1, IL23A, FCER1G, HLA-DRB5, DHX58, ARHGDIB, IL1RN, CD300E, MYO1F, SMAD3, CD300C, SLAMF7, VAV1, FOXP1, TRAF3IP2, LAT, CD55, TNFSF10, OASL, CORO1A, RGS1, FCAR, CD300A, OTUB1, CXCL16, POMP, CLEC7A, TREML1, TCF12, IFI6, KYNU, NBN, TOLLIP, LY86, PRDX2, NFKB2, PRDX1, CD97, TMEM173, CXCR4, IL4R, TICAM1, LTF, NFIL3, ICAM1, LAIR1, IK, SP100, LYN, LY96, INPPL1, CNPY3, STXBP2, ECSIT, NLRP3, CD164, WAS, PRKCD, CTSW, OSM, CD83, CD86, TNFSF13B, PSEN1, C1QBP, CTSC, TREM1, PTMS, LCP1, LCP2, CXCL1, PSMB10, HLA-DQB1, CSF3, CSF2, GPR183, SBNO2, YWHAZ, CCR1, CXCL3, CXCL2, CALCOCO2, RSAD2, PF4, CLEC10A, GCH1, TNFRSF1A, TNFRSF1B, CCL20, IL10RB, XBP1, SQSTM1, FCN1, BCL3, CD4, IL2RG, PTX3, THBS1, ERCC1, PTPRC, SECTM1, IL6, CEBPB, OLR1, IL8, SAMHD1, TRIM22, TNFSF8, CYBA, GPI, PRELID1, CYBB, ILF2, BNIP3L, ANXA11, IRF8, RNF19B, CD14	
GO:0022900~electron transport chain	1.83E-15	UQCRC2, UQCRC1, CYC1, SNCA, NDUFAB1, UQCRFS1, UQCRQ, NDUFS6, NDUFS5,	5.03E-13

		UQCR11, NDUFS4, NDUFS3, NDUFS2, TXNL1, NDUFB11, NDUFB10, TAZ, CYCS, NDUFC2, NDUFA13, NDUFC1, NDUFA10, NDUFA12, NDUFA11, UQCRH, TXN, UQCRB, NDUFB3, NDUFB4, NDUFB5, NDUFB7, NDUFB8, TXN2, NDUFB9, SDHAF2, NDUFB1, NDUFB2, ETFB, GLRX, ETFA, NDUFA4, NDUFA5, NDUFA2, NDUFA3, NDUFA9, NDUFA6, NDUFA7, NDUFA1, SOD2, SDHA, SDHB, CYBA, CYBB, NDUFV1, SDHC	
GO:0032268~regulation of cellular protein metabolic process	4.72E-15	NCBP1, SRP14, AURKAIP1, SNCA, EIF5, EIF5A, IL10, TGFB1, LUZP6, EIF4EBP1, ATG7, WIBG, IL1B, EIF1, DNAJC1, RPS27A, EIF2B5, DBNL, GTPBP4, ANAPC5, SOCS3, MAGOH, NRD1, TNFRSF14, SKP1, PSMA2, PSMA1, EIF2AK1, PSMA6, PSMA5, PSMA4, PSMA3, UBA52, MAP4K1, NFKBIA, ANAPC10, ITGB2, ANAPC11, CALR, PSMA7, TIMP1, NR1H2, PSMB4, PSMB7, PSMB6, EIF3B, PSMB1, EIF3H, EIF3E, PSMB2, PEMT, EIF3K, TAF9, UBE2D1, FCER1A, PAIP2, TAF7, RPS5, EIF4B, PSMC5, EIF4E, PSMC4, PSMC3, PSMC2, EIF4H, PRKAR1A, EIF4E2, UBE2E1, HDAC6, HSP90AB1, PRKAG2, ZEB2, NFKB1, EIF2A, PDCD4, NDUFS4, PSMD1, PSMD2, QKI, PSMD4, PAK1, PSMD6, PSMD7, PSMD8, PSMD9, PPP2R1A, CLN3, LYN, CCDC88C,	1.27E-12

		PKN1, NDUFA13, EIF1B, PRKCE, RPS4X, CDK5, PRKCD, UBE2N, SARNP, OSM, EIF4A3, NCK2, EIF4G2, PSEN1, PSME1, CCND3, PSME2, EIF4A2, UBC, MDM2, UBB, PPP1R15B, PPP1R15A, MAP3K11, PSMB10, CSF2, MKNK2, PML, FKBP1A, STUB1, ZFP36L1, PSMF1, SET, PTK2B, PPP2CA, BCL3, CD4, THBS1, TINF2, PTPRC, IL6, HCLS1, YWHAB, ANKHD1, ETF1, SELT, YWHAE, PSMD13, PSMD11, BAX, NGDN, SH3D19, VPS28	
GO:0051443~positive regulation of ubiquitin-protein ligase activity	1.70E-14	PSMB10, ANAPC10, ANAPC11, PSMA7, PSMB4, PSMF1, PSMB7, PSMB6, PSMB1, PSMD1, PSMB2, PSMD2, PSMD4, UBE2D1, PSMD6, PSMD7, RPS27A, PSMD8, PSMD9, ANAPC5, SKP1, UBE2N, PSMA2, PSMA1, PSMD13, PSMC5, PSMA6, PSME1, PSMC4, PSMA5, PSME2, PSMD11, PSMC3, PSMC2, PSMA4, PSMA3, UBC, UBB, UBA52, UBE2E1	4.30E-12
GO:0031400~negative regulation of protein modification process	1.82E-14	SNCA, TGFB1, PSMD1, PSMD2, PSMD4, PSMD6, PSMD7, PSMD8, RPS27A, PSMD9, PPP2R1A, GTPBP4, ANAPC5, PRKCD, CDK5, PSMA2, PSMA1, PSMA6, PSME1, PSEN1, PSMA5, PSME2, PSMA4, PSMA3, UBC, UBB, UBA52, PSMB10, FKBP1A, ANAPC10, ANAPC11, PSMA7, PSMB4, PSMF1, PSMB7, SET, PSMB6, PSMB1, PPP2CA, PSMB2, UBE2D1, TINF2, PTPRC, TAF7, YWHAB, YWHAE,	4.34E-12

		PSMD13, PSMC5, PSMC4, PSMC3, PSMD11, BAX, PSMC2, VPS28, UBE2E1	
GO:0043161~proteasomal ubiquitin-dependent protein catabolic process	1.88E-14	KIAA0368, PPP2R5C, OS9, CUL3, PSMD1, PSMD2, PSMD4, PSMD6, PSMD7, RPS27A, PSMD8, PSMD9, ANAPC5, PSMA2, PSMA1, SEC61B, PSME1, PSMA6, PSMA5, PSME2, PSMA4, PSMA3, UBC, UBB, UBA52, PSMB10, DERL2, DERL1, RAD23A, ANAPC10, ANAPC11, PSMA7, STUB1, PSMF1, PSMB4, PSMB7, PSMB6, PSMB1, PSMB2, UBE2D1, UBXN1, HSP90B1, PSMD13, PSMC5, PSMC4, VCP, PSMC3, PSMD11, PSMC2, UBE2E1	4.25E-12
GO:0010498~proteasomal protein catabolic process	1.88E-14	KIAA0368, PPP2R5C, OS9, CUL3, PSMD1, PSMD2, PSMD4, PSMD6, PSMD7, RPS27A, PSMD8, PSMD9, ANAPC5, PSMA2, PSMA1, SEC61B, PSME1, PSMA6, PSMA5, PSME2, PSMA4, PSMA3, UBC, UBB, UBA52, PSMB10, DERL2, DERL1, RAD23A, ANAPC10, ANAPC11, PSMA7, STUB1, PSMF1, PSMB4, PSMB7, PSMB6, PSMB1, PSMB2, UBE2D1, UBXN1, HSP90B1, PSMD13, PSMC5, PSMC4, VCP, PSMC3, PSMD11, PSMC2, UBE2E1	4.25E-12
GO:0031397~negative regulation of protein ubiquitination	3.10E-14	PSMB10, ANAPC10, ANAPC11, PSMA7, PSMB4, PSMF1, PSMB7, PSMB6, PSMB1, PSMD1, PSMB2, PSMD2, PSMD4, UBE2D1, PSMD6, PSMD7, RPS27A, PSMD8, PSMD9, GTPBP4, ANAPC5, CDK5, PSMA2, PSMA1, PSMD13, PSMC5, PSMA6, PSME1, PSMC4, PSMA5, PSME2,	6.68E-12

		PSMD11, PSMC3, PSMC2, PSMA4, PSMA3, UBC, UBB, VPS28, UBA52, UBE2E1	
GO:0031145~anaphase-promoting complex-dependent proteasomal ubiquitin-dependent protein catabolic process	3.19E-14	PSMB10, ANAPC10, ANAPC11, PSMA7, PSMB4, PSMF1, PSMB7, PSMB6, PSMB1, PSMD1, PSMB2, PSMD2, PSMD4, UBE2D1, PSMD6, PSMD7, RPS27A, PSMD8, PSMD9, ANAPC5, PSMA2, PSMA1, PSMD13, PSMC5, PSMA6, PSME1, PSMC4, PSMA5, PSME2, PSMD11, PSMC3, PSMC2, PSMA4, PSMA3, UBC, UBB, UBA52, UBE2E1	6.56E-12
GO:0051436~negative regulation of ubiquitin-protein ligase activity during mitotic cell cycle	3.19E-14	PSMB10, ANAPC10, ANAPC11, PSMA7, PSMB4, PSMF1, PSMB7, PSMB6, PSMB1, PSMD1, PSMB2, PSMD2, PSMD4, UBE2D1, PSMD6, PSMD7, RPS27A, PSMD8, PSMD9, ANAPC5, PSMA2, PSMA1, PSMD13, PSMC5, PSMA6, PSME1, PSMC4, PSMA5, PSME2, PSMD11, PSMC3, PSMC2, PSMA4, PSMA3, UBC, UBB, UBA52, UBE2E1	6.56E-12
GO:0051437~positive regulation of ubiquitin-protein ligase activity during mitotic cell cycle	3.24E-14	PSMB10, ANAPC10, ANAPC11, PSMA7, PSMB4, PSMF1, PSMB7, PSMB6, PSMB1, PSMD1, PSMB2, PSMD2, PSMD4, UBE2D1, PSMD6, PSMD7, RPS27A, PSMD8, PSMD9, ANAPC5, SKP1, PSMA2, PSMA1, PSMD13, PSMC5, PSMA6, PSME1, PSMC4, PSMA5, PSME2, PSMD11, PSMC3, PSMC2, PSMA4, PSMA3, UBC, UBB, UBA52, UBE2E1	6.36E-12
GO:0006915~apoptosis	6.83E-14	MEF2C, HRAS, TSPO, RBM5, TNFSF13, PMAIP1, CRADD, CTNNB1, CTNNBL1, IL1B, PSENEN,	1.29E-11

		DAP, LUC7L3, IL1A, RPS27A, CIB1, YARS, MADD, BCL2A1, TNFRSF14, PIM2, BCL2L13, BCL2L11, AHR, DDIT4, BCAP31, SLTM, RNF130, TIAL1, F3, TNFAIP8, RIPK2, PDCD6IP, TNFAIP3, UBA52, SIVA1, TRAF1, MCL1, LITAF, ADORA2A, CLU, STK17B, AKAP13, NFKBIA, ITGB2, ARF6, PPT1, RFFL, SLC11A2, FIS1, PEA15, ECE1, TRIM69, MTCH1, RAC1, DIABLO, HSPE1, DDX41, SRGN, TRAF3, PTPN6, CFLAR, CARD8, ABR, TM2D1, LGALS1, SRA1, DUSP22, SMAD3, VAV1, TAX1BP1, FXR1, TRAF3IP2, TXNDC12, TNFSF10, VCP, RABEP1, CSRNP1, CYFIP2, PDCD5, PDCD6, IFI6, PUF60, RTN4, LY86, NFKB1, PDCD4, PTEN, RTN3, RPS3, ARHGAP4, TMEM173, CASP4, BAG1, DYNLL1, GSN, CXCR4, TICAM1, CASP8, PAK1, CASP1, DEDD2, SGK1, ARHGEF2, CYCS, PIGT, NDUFA13, GZMB, NLRP3, CDK5, NLRP1, ELMO1, PPM1F, OSM, TNFRSF10B, PSEN1, BNIP2, CDK11A, UBC, UBB, GADD45B, PPP1R15A, BID, POLR2G, FKBP8, UBE2Z, APH1A, PML, ITM2B, TNFRSF1A, PLEKHG2, TNFRSF1B, TSC22D3, SH3GLB1, PTK2B, SQSTM1, SHISA5, DAD1, PYCARD, ZC3H12A, PPP3CC, THBS1, PHLDA2, PHLDA1, RNF144B, IL6, TMBIM6, YWHAB, BIRC6,	

		RAF1, SAP30BP, BAD, STAT1, SOD1, BIRC3, YWHAE, BIRC2, SOD2, MEF2D, JMJD6, BAX, IFT57, BNIP3L, BRE, DRAM1, CD14	
GO:0016192~vesicle-mediated transport	1.05E-13	SEPT5, HRAS, NRBP1, AP1G2, PDLIM7, CHMP5, SNCA, USE1, RAB1A, CTNNB1, PICALM, RABGEF1, SAR1B, SAR1A, FTL, DBNL, SCAMP2, VTI1B, OPTN, ERGIC1, YIF1A, VTI1A, ERGIC3, BCAP31, LAT2, CD36, ARRB2, VAMP8, LYST, TRAPPC5, VAMP5, VAMP3, RAB13, BIN1, TRAPPC1, TRAPPC3, RAB7A, ARFGAP2, ARFGAP3, STX8, ADORA2A, COPZ1, SNX2, CDC42SE1, RER1, NAPA, SNX4, ARF6, PPT1, ARF5, ABCA1, SNX3, FTH1, SRC, SLC11A1, MIA3, RAC1, BLOC1S1, FCER1G, TMED10, STX11, STX10, SDF4, AP2M1, GABARAPL2, GARS, DENND1A, MYO1F, VAV1, CORO1C, LAT, CORO1A, RABEP1, VCP, CDC42SE2, CXCL16, ARF3, ARF4, CPNE1, HGS, SEC13, CLEC7A, NPEPL1, CLTA, CLTB, LDLR, MARCKSL1, ATP5B, AP2S1, RTN3, ASGR2, TRAPPC6A, AP1S2, CD93, GSN, ACTR1A, COPB1, NECAP2, CAP1, CLINT1, KDELR1, AGAP3, STX5, CLN3, STX4, STX3, LYN, RAB4B, STXBP2, ATP6V1H, PI4KB, M6PR, CDK5, WAS, FLNA, LRPAP1, ELMO1, ADRB2, CHMP1A, PSEN1, RIN2, LRMP, TOM1, CUX1,	1.90E-11

		COPE, RIN3, AP1M1, YWHAZ, CYTH1, SNX17, CYTH2, CLEC10A, TMED2, SQSTM1, FCN1, EXOC4, EHD1, PTX3, TRIP10, THBS1, HSPA8, EHD4, RAB2A, PLEK, HCK, RUFY1, MARCH3, COG3, LMBR1L, MARCH2, LRP1, NME1-NME2, JMJD6, AP2A1, RAB34, SH3D19, GGA1, GGA2, CD14, DNM2	
GO:0051351~positive regulation of ligase activity	1.08E-13	PSMB10, ANAPC10, ANAPC11, PSMA7, PSMB4, PSMF1, PSMB7, PSMB6, PSMB1, PSMD1, PSMB2, PSMD2, PSMD4, UBE2D1, PSMD6, PSMD7, RPS27A, PSMD8, PSMD9, ANAPC5, SKP1, UBE2N, PSMA2, PSMA1, PSMD13, PSMC5, PSMA6, PSME1, PSMC4, PSMA5, PSME2, PSMD11, PSMC3, PSMC2, PSMA4, PSMA3, UBC, UBB, UBA52, UBE2E1	1.88E-11
GO:0051444~negative regulation of ubiquitin-protein ligase activity	1.17E-13	PSMB10, ANAPC10, ANAPC11, PSMA7, PSMB4, PSMF1, PSMB7, PSMB6, PSMB1, PSMD1, PSMB2, PSMD2, PSMD4, UBE2D1, PSMD6, PSMD7, RPS27A, PSMD8, PSMD9, ANAPC5, PSMA2, PSMA1, PSMD13, PSMC5, PSMA6, PSME1, PSMC4, PSMA5, PSME2, PSMD11, PSMC3, PSMC2, PSMA4, PSMA3, UBC, UBB, UBA52, UBE2E1	1.95E-11
GO:0051352~negative regulation of ligase activity	1.17E-13	PSMB10, ANAPC10, ANAPC11, PSMA7, PSMB4, PSMF1, PSMB7, PSMB6, PSMB1, PSMD1, PSMB2, PSMD2, PSMD4, UBE2D1, PSMD6, PSMD7, RPS27A, PSMD8, PSMD9, ANAPC5, PSMA2, PSMA1, PSMD13, PSMC5, PSMA6,	1.95E-11

		PSME1, PSMC4, PSMA5, PSME2, PSMD11, PSMC3, PSMC2, PSMA4, PSMA3, UBC, UBB, UBA52, UBE2E1	
GO:0022904~respiratory electron transport chain	1.17E-13	NDUFB3, NDUFB4, NDUFB5, UQCRC1, NDUFB7, NDUFB8, NDUFB9, SNCA, NDUFAB1, SDHAF2, NDUFB1, NDUFB2, NDUFS6, NDUFS5, NDUFS4, UQCR11, NDUFS3, NDUFS2, NDUFA4, NDUFA5, NDUFA2, NDUFA3, NDUFB10, NDUFA9, NDUFA6, NDUFA7, TAZ, NDUFC2, NDUFC1, NDUFA10, NDUFA1, SOD2, SDHA, SDHB, UQCRH, NDUFV1, UQCRB	1.89E-11
GO:0012501~programmed cell death	1.30E-13	MEF2C, HRAS, TSPO, RBM5, TNFSF13, PMAIP1, CRADD, CTNNB1, CTNNBL1, CDKN2D, IL1B, PSENEN, DAP, LUC7L3, IL1A, RPS27A, CIB1, YARS, MADD, BCL2A1, TNFRSF14, PIM2, BCL2L13, BCL2L11, AHR, DDIT4, BCAP31, SLTM, RNF130, TIAL1, F3, TNFAIP8, RIPK2, PDCD6IP, TNFAIP3, UBA52, SIVA1, TRAF1, MCL1, LITAF, ADORA2A, CLU, STK17B, AKAP13, NFKBIA, ITGB2, ARF6, PPT1, RFFL, SLC11A2, FIS1, PEA15, ECE1, TRIM69, MTCH1, RAC1, DIABLO, HSPE1, DDX41, SRGN, TRAF3, PTPN6, CFLAR, CARD8, ABR, TM2D1, LGALS1, SRA1, DUSP22, SMAD3, VAV1, TAX1BP1, FXR1, TRAF3IP2, TXNDC12,	2.03E-11

		TNFSF10, VCP, RABEP1, CSRNP1, CYFIP2, PDCD5, PDCD6, IFI6, PUF60, RTN4, LY86, NFKB1, PDCD4, PTEN, RTN3, RPS3, ARHGAP4, TMEM173, CASP4, BAG1, DYNLL1, GSN, CXCR4, TICAM1, CASP8, PAK1, CASP1, DEDD2, SGK1, ARHGEF2, CYCS, PIGT, NDUFA13, GZMB, NLRP3, CDK5, NLRP1, ELMO1, PPM1F, OSM, TNFRSF10B, PSEN1, BNIP2, CDK11A, UBC, UBB, GADD45B, PPP1R15A, BID, POLR2G, FKBP8, UBE2Z, APH1A, PML, ITM2B, TNFRSF1A, PLEKHG2, TNFRSF1B, TSC22D3, SH3GLB1, PTK2B, SQSTM1, SHISA5, DAD1, PYCARD, ZC3H12A, PPP3CC, THBS1, PHLDA2, PHLDA1, RNF144B, IL6, TMBIM6, YWHAB, BIRC6, RAF1, SAP30BP, BAD, STAT1, SOD1, BIRC3, YWHAE, BIRC2, SOD2, MEF2D, JMJD6, BAX, IFT57, BNIP3L, BRE, DRAM1, CD14	
GO:0008219~cell death	1.45E-13	MEF2C, HRAS, TSPO, ATP6AP1, RBM5, TNFSF13, PMAIP1, TGFB1, CRADD, CTNNB1, CTNNBL1, ATXN10, CDKN2D, TARDBP, IL1B, PSENEN, DAP, LUC7L3, RPS27A, IL1A, CIB1, YARS, MADD, BCL2A1, TNFRSF14, POLB, OPTN, PIM2, BCL2L13, BCL2L11, AHR, PNPLA6, DDIT4, BCAP31, SLTM, RNF130, TIAL1, F3, TNFAIP8, RIPK2, PDCD6IP, TNFAIP3, UBA52, FUS, SIVA1, TRAF1, MCL1,	2.19E-11

		LITAF, ADORA2A, CLU, STK17B, AKAP13, NFKBIA, ITGB2, ARF6, PPT1, RFFL, SLC11A2, FIS1, PEA15, ECE1, TRIM69, MTCH1, RAC1, DIABLO, HSPE1, DDX41, SRGN, TRAF3, PTPN6, CFLAR, CARD8, ABR, TM2D1, LGALS1, SRA1, GARS, DUSP22, TREX1, SMAD3, VAV1, TAX1BP1, FXR1, TRAF3IP2, TXNDC12, TNFSF10, RABEP1, VCP, UBA1, CSRNP1, CYFIP2, PDCD5, PDCD6, IFI6, PUF60, RTN4, SPG7, FOSL2, LY86, BSCL2, NFKB1, PTEN, PDCD4, RTN3, RPS3, ARHGAP4, TMEM173, CASP4, BAG1, DYNLL1, GSN, CXCR4, TICAM1, CASP8, PAK1, CASP1, DEDD2, CLN3, SGK1, ARHGEF2, CYCS, LYZ, PIGT, NDUFA13, GZMB, NLRP3, CDK5, NLRP1, ELMO1, PPM1F, OSM, EIF4G2, TNFRSF10B, PSEN1, BNIP2, CDK11A, UBC, UBB, GADD45B, PPP1R15A, MAP3K11, BID, POLR2G, FKBP8, UBE2Z, APH1A, PML, ITM2B, TNFRSF1A, PLEKHG2, TNFRSF1B, TSC22D3, SH3GLB1, SQSTM1, PTK2B, SHISA5, DAD1, PYCARD, PPP3CC, ZC3H12A, THBS1, PHLDA2, PHLDA1, RNF144B, IL6, OLR1, TMBIM6, YWHAB, BIRC6, RAF1, SAP30BP, BAD, STAT1, SOD1, BIRC3, YWHAE, BIRC2, SOD2, ATXN1, MEF2D, ATXN3, JMJD6, BAX, IFT57,	

		BNIP3L, BRE, SPG11, DRAM1, CD14	
GO:0042773~ATP synthesis coupled electron transport	2.00E-13	NDUFB3, NDUFB4, NDUFB5, UQCRC1, NDUFB7, NDUFB8, NDUFB9, SNCA, NDUFAB1, SDHAF2, NDUFB1, NDUFB2, NDUFS6, NDUFS5, NDUFS4, UQCR11, NDUFS3, NDUFS2, NDUFA4, NDUFA5, NDUFA2, NDUFA3, NDUFB10, NDUFA9, NDUFA6, NDUFA7, TAZ, NDUFC2, NDUFC1, NDUFA10, NDUFA1, UQCRH, NDUFV1, UQCRB	2.93E-11
GO:0042775~mitochondrial ATP synthesis coupled electron transport	2.00E-13	NDUFB3, NDUFB4, NDUFB5, UQCRC1, NDUFB7, NDUFB8, NDUFB9, SNCA, NDUFAB1, SDHAF2, NDUFB1, NDUFB2, NDUFS6, NDUFS5, NDUFS4, UQCR11, NDUFS3, NDUFS2, NDUFA4, NDUFA5, NDUFA2, NDUFA3, NDUFB10, NDUFA9, NDUFA6, NDUFA7, TAZ, NDUFC2, NDUFC1, NDUFA10, NDUFA1, UQCRH, NDUFV1, UQCRB	2.93E-11
GO:0051439~regulation of ubiquitin-protein ligase activity during mitotic cell cycle	2.07E-13	PSMB10, ANAPC10, ANAPC11, PSMA7, PSMB4, PSMF1, PSMB7, PSMB6, PSMB1, PSMD1, PSMB2, PSMD2, PSMD4, UBE2D1, PSMD6, PSMD7, RPS27A, PSMD8, PSMD9, ANAPC5, SKP1, PSMA2, PSMA1, PSMD13, PSMC5, PSMA6, PSME1, PSMC4, PSMA5, PSME2, PSMD11, PSMC3, PSMC2, PSMA4, PSMA3, UBC, UBB, UBA52, UBE2E1	2.93E-11

GO:0045333~cellular respiration	2.15E-13	UQCRC2, NDUFB3, NDUFB4, OXA1L, NDUFB5, UQCRC1, NDUFB7, NDUFB8, NDUFB9, SNCA, NDUFAB1, SDHAF2, NDUFB1, NDUFB2, NDUFS6, NDUFS5, UQCR11, NDUFS4, IDH3G, NDUFS3, NDUFS2, NDUFA4, NDUFA5, NDUFA2, NDUFA3, NDUFB10, NDUFA9, NDUFA6, SUCLG1, NDUFA7, TAZ, CYCS, NDUFC2, IDH3B, NDUFC1, NDUFA10, NDUFA1, SOD2, SDHA, SDHB, UQCRH, SDHC, NDUFV1, SURF1, MDH2, UQCRB, MDH1	2.95E-11
GO:0006916~anti-apoptosis	2.76E-13	MEF2C, STAT5A, SNCA, NFKB1, PRDX2, IL10, CITED2, MYD88, BAG1, CDKN2D, TPT1, IL1B, NRG1, IL1A, RPS27A, IRAK1, SOCS3, RELA, BCL2A1, POLB, PIM2, SERPINB9, PSEN1, TNFSF13B, BNIP2, F3, TNFAIP8, CFL1, VEGFA, UBC, SERPINB2, RIPK2, UBB, PRNP, TNFAIP3, MYO18A, UBA52, GSTP1, CSF2, YWHAZ, CCL2, MCL1, CLU, NFKBIA, TCF7L2, PEA15, SQSTM1, SH3GLB1, NPM1, DAD1, HSPA5, THBS1, HSPA9, CFLAR, CEBPB, BECN1, SPHK1, ANXA1, BIRC6, ANXA5, SOD1, BIRC3, TAX1BP1, SOD2, ATF5, SON, HDAC3, HSP90B1, RNF7, PRLR, HDAC1, GSK3B, BAX, BNIP3L, IKBKB, IFI6	3.68E-11
GO:0016265~death	2.91E-13	MEF2C, HRAS, TSPO, ATP6AP1, RBM5, TNFSF13, PMAIP1,	3.77E-11

		TGFB1, CRADD, CTNNB1, CTNNBL1, ATXN10, CDKN2D, TARDBP, IL1B, PSENEN, DAP, LUC7L3, RPS27A, IL1A, CIB1, YARS, MADD, BCL2A1, TNFRSF14, POLB, OPTN, PIM2, BCL2L13, BCL2L11, AHR, PNPLA6, DDIT4, BCAP31, SLTM, RNF130, TIAL1, F3, TNFAIP8, RIPK2, PDCD6IP, TNFAIP3, UBA52, FUS, SIVA1, TRAF1, MCL1, LITAF, ADORA2A, CLU, STK17B, AKAP13, NFKBIA, ITGB2, ARF6, PPT1, RFFL, SLC11A2, FIS1, PEA15, ECE1, TRIM69, MTCH1, RAC1, DIABLO, HSPE1, DDX41, SRGN, TRAF3, PTPN6, CFLAR, CARD8, ABR, TM2D1, LGALS1, SRA1, GARS, DUSP22, TREX1, SMAD3, VAV1, TAX1BP1, FXR1, TRAF3IP2, TXNDC12, TNFSF10, RABEP1, VCP, UBA1, CSRNP1, CYFIP2, PDCD5, PDCD6, IFI6, PUF60, RTN4, SPG7, FOSL2, LY86, BSCL2, NFKB1, PTEN, PDCD4, RTN3, RPS3, ARHGAP4, TMEM173, CASP4, BAG1, DYNLL1, GSN, CXCR4, TICAM1, CASP8, PAK1, CASP1, DEDD2, CLN3, SGK1, ARHGEF2, CYCS, LYZ, PIGT, NDUFA13, GZMB, NLRP3, CDK5, NLRP1, ELMO1, PPM1F, OSM, EIF4G2, TNFRSF10B, PSEN1, BNIP2, CDK11A, UBC, UBB, GADD45B, PPP1R15A, MAP3K11, BID, POLR2G, FKBP8, UBE2Z, APH1A, PML,	

		ITM2B, TNFRSF1A, PLEKHG2, TNFRSF1B, TSC22D3, SH3GLB1, SQSTM1, PTK2B, SHISA5, DAD1, PYCARD, PPP3CC, ZC3H12A, THBS1, PHLDA2, PHLDA1, RNF144B, IL6, OLR1, TMBIM6, YWHAB, BIRC6, RAF1, SAP30BP, BAD, STAT1, SOD1, BIRC3, YWHAE, BIRC2, SOD2, ATXN1, MEF2D, ATXN3, JMJD6, BAX, IFT57, BNIP3L, BRE, SPG11, DRAM1, CD14	
GO:0031398~positive regulation of protein ubiquitination	1.31E-12	PSMB10, FKBP1A, ANAPC10, ANAPC11, PSMA7, STUB1, PSMB4, PSMF1, PSMB7, PSMB6, PSMB1, PSMD1, PSMB2, PSMD2, PSMD4, UBE2D1, PSMD6, PSMD7, RPS27A, PSMD8, PSMD9, ANAPC5, SKP1, UBE2N, PSMA2, PSMA1, PSMD13, PSMC5, PSMA6, PSME1, PSMC4, PSMA5, PSME2, PSMD11, PSMC3, PSMC2, PSMA4, PSMA3, UBC, UBB, UBA52, UBE2E1	1.65E-10
GO:0046907~intracellular transport	1.55E-12	GNPTG, NCBP1, SRP14, TSPO, GRPEL1, NRBP1, AP1G2, XPO6, CHMP5, ATP6AP1, USE1, EIF5A, SRP19, TGFB1, AIP, STARD3, HOMER3, TIMM9, RPL11, SAR1B, SAR1A, FTL, PABPN1, SCAMP2, RAN, ERP29, VTI1B, OPTN, MYH9, ERGIC1, VTI1A, BCAP31, UXT, NPC2, SEC61B, VAMP8, ATP2C1, LYST, RPS15, SLC25A37, SDCBP, CAMK1, TRAPPC1, SEC61G, TRAPPC3, MYL6, DERL2, ARFGAP3, STX8, DERL1, AGFG1, CHKB, COPZ1, SNX2, NFKBIA,	1.90E-10

		RER1, NAPA, CTSA, MYO9B, ABCA1, RFFL, CALR, FTH1, CD74, CDC37, SLC11A1, TOMM7, TOMM5, BLOC1S1, NPM1, TMED10, STX11, STX10, TRAM1, SEC61A1, AP2M1, GABARAPL2, NXF1, HNRNPA1, PPIF, VCP, UCP2, HGS, SEC13, HDAC6, ATP5D, SPG7, NPEPL1, CLTA, CLTB, PDIA3, AP2S1, TIMM17A, TIMM17B, AP1S2, SRPR, COPB1, ANP32A, ATP5O, SLC25A1, VPS16, CLINT1, KDELR1, AGAP3, STX5, ARHGEF2, STX4, HSP90AA1, STX3, NDUFA13, M6PR, CDK5, WAS, FLNA, ADRB2, ATG4D, PSEN1, IPO5, TOMM20, THOC7, TOM1, SIL1, TOMM22, GNAS, THOC2, CUX1, KPNA2, COPE, NXT1, BID, AP1M1, YWHAZ, SNX17, PML, TIMM10, TPM1, SEC62, NUP214, SET, KLC1, SQSTM1, PEX16, EXOC4, RAB11A, BCL3, PAFAH1B1, BCL6, CCS, EHD1, HSPA8, HSPA9, RAB2A, RBM22, YWHAB, YWHAE, GABARAP, ATXN1, COG3, ERBB2IP, RPL23, AP2A1, GSK3B, BAX, YWHAQ, TRPC4AP, NOP58, SH3D19, GGA1, SSR4, GGA2, SSR2, DNM2, SSR3	
GO:0051438~regulation of ubiquitin-protein ligase activity	1.75E-12	PSMB10, ANAPC10, ANAPC11, PSMA7, PSMB4, PSMF1, PSMB7, PSMB6, PSMB1, PSMD1, PSMB2, PSMD2, PSMD4, UBE2D1, PSMD6, PSMD7, RPS27A, PSMD8, PSMD9, ANAPC5, SKP1, UBE2N,	2.08E-10

		PSMA2, PSMA1, PSMD13, PSMC5, PSMA6, PSME1, PSMC4, PSMA5, PSME2, PSMD11, PSMC3, PSMC2, PSMA4, PSMA3, UBC, UBB, UBA52, UBE2E1	
GO:0016044~membrane organization	3.30E-12	HRAS, OXA1L, VAPA, PDLIM7, SNCA, PICALM, ATG7, TIMM9, RABGEF1, SAR1B, FTL, DBNL, TAZ, VTI1B, CD36, ARRB2, VAMP8, CATSPER1, VAMP3, BIN1, RAB7A, STX8, ADORA2A, COPZ1, SNX2, CDC42SE1, NAPA, PPT1, SNX4, SNX3, ABCA1, FTH1, SLC11A1, DOCK2, BLOC1S1, RAC1, MTCH1, FCER1G, STX11, EMD, DENND1A, VAV1, PPIF, CORO1C, CORO1A, RABEP1, CDC42SE2, CXCL16, SEC13, CLEC7A, CLTA, LDLR, ATP5B, AP2S1, ASGR2, AP1S2, CD93, COPB1, NECAP2, CAP1, CLINT1, CLN3, STX4, HSP90AA1, STX3, ATP6V1H, PI4KB, CDK5, M6PR, ELMO1, ADRB2, RIN2, LRMP, TOM1, TOMM22, COPE, RIN3, SERP1, BID, AP1M1, SNX17, TIMM10, CYTH2, CLEC10A, CD9, SH3GLB1, FCN1, PAFAH1B1, HSPA4, THBS1, TRIP10, PTX3, EHD1, HSPA8, EHD4, HCK, RUFY1, LMNA, SOD1, MARCH3, KCNN4, LMBR1L, MARCH2, PLSCR1, LRP1, NME1-NME2, JMJD6, AP2A1, BAX, RAB34, SH3D19, GCA, CD14, DNM2	3.83E-10
GO:0015031~protein transport	6.03E-12	GNPTG, SRP14, GRPEL1, TSPO, AP1G2, XPO6, CHMP5, CHMP4B, ATP6AP1, USE1, RAB1B, EIF5A, VPS53, SRP19,	6.82E-10

		RAB1A, TGFB1, CXCL10, AIP, KIF13A, HOMER3, ATG7, TIMM9, RABGEF1, RAB24, RPL11, SAR1B, RAB20, SAR1A, SCAMP2, RAN, ERP29, VTI1B, OPTN, MYH9, YIF1A, VTI1A, BCAP31, CD36, SEC61B, ACAP1, LYST, SDCBP, RAB13, PDCD6IP, RAB10, SEC61G, MVP, CHMP2A, RAB7A, ARFGAP2, DERL2, ARFGAP3, DERL1, COPZ1, SNX2, NFKBIA, NAPA, SNX4, ARF6, CTSA, PPT1, ARF5, LMAN2, ABCA1, SNX3, RFFL, CALR, CD74, CDC37, SLC11A1, MIA3, TOMM7, TOMM6, TOMM5, SNF8, NPM1, TMED10, STX11, STX10, TRAM1, SEC61A1, AP2M1, GABARAPL2, RILP, RAB8B, ATG3, TRAF3IP2, RABEP1, VCP, ARF3, ARF4, HGS, SEC13, ATG16L2, HDAC6, NPEPL1, CLTA, CLTB, PDIA3, RAB5C, TSG101, TIMM17A, AP2S1, TIMM17B, CANX, AP1S2, RNF103, SRPR, COPB1, NECAP2, VPS16, KDELR1, AGAP3, STX5, ARHGEF2, STX4, STX3, LYN, NUP88, RAB4B, STXBP2, NDUFA13, NLRP3, CDK5, CHMP1A, ATG4D, CHMP1B, PSEN1, TNFSF13B, ATG4B, IPO5, TOMM20, TOM1, SIL1, TOMM22, GNAS, KPNA2, SNX10, COPE, LCP2, SERP1, VPS29, NXT1, BID, AP1M1, YWHAZ, NACA, SNX17, PML, TIMM10, NUP93, SEC62, SFT2D1, NUP214, TMED2, TMED3,	

		PEX16, EXOC4, RAB11A, BCL3, BCL6, SNX24, NACA2, HSPA9, RAB2A, RBM22, GDI1, GDI2, PLEK, YWHAB, RUFY1, YWHAE, GABARAP, COG3, RAB32, RAB31, HSP90B1, ERBB2IP, RPL23, AP2A1, GSK3B, RAB34, YWHAQ, TRPC4AP, NOP58, GGA1, SSR4, VPS28, SLC15A3, GGA2, SSR2, SSR3	
GO:0045184~establishment of protein localization	7.66E-12	CHMP5, USE1, RAB1B, VPS53, RAB1A, KIF13A, HOMER3, RAB24, RPL11, RAB20, RAN, VTI1B, OPTN, MYH9, YIF1A, VTI1A, CD36, ACAP1, RAB13, RAB10, NFKBIA, ARF6, PPT1, ARF5, RFFL, CALR, CDC37, CD74, SLC11A1, TMED10, STX11, TRAM1, STX10, RABEP1, ARF3, ARF4, HGS, PDIA3, TSG101, RAB5C, TIMM17A, AP2S1, TIMM17B, CANX, AP1S2, SRPR, COPB1, KDELR1, AGAP3, STX5, STX4, ARHGEF2, STX3, LYN, RAB4B, NDUFA13, FLNA, CHMP1A, CHMP1B, PSEN1, TOMM20, SIL1, TOMM22, GNAS, SERP1, NXT1, BID, YWHAZ, NACA, PML, SFT2D1, NUP214, PEX16, EXOC4, BCL3, RAB11A, BCL6, RBM22, RAB2A, GDI1, GDI2, PLEK, YWHAB, YWHAE, GABARAP, COG3, RAB32, RAB31, ERBB2IP, RPL23, AP2A1, GSK3B, RAB34, YWHAQ, NOP58, SSR4, SLC15A3, SSR2, SSR3, GNPTG, SRP14, TSPO, GRPEL1, XPO6, AP1G2, CHMP4B, ATP6AP1, EIF5A, SRP19,	8.46E-10

		TGFB1, CXCL10, AIP, ATG7, TIMM9, RABGEF1, SAR1B, SAR1A, SCAMP2, ERP29, BCAP31, SEC61B, LYST, SDCBP, PDCD6IP, SEC61G, MVP, CHMP2A, RAB7A, ARFGAP2, ARFGAP3, DERL2, DERL1, COPZ1, SNX2, CTSA, SNX4, NAPA, LMAN2, ABCA1, SNX3, MIA3, TOMM7, TOMM6, TOMM5, SNF8, NPM1, SEC61A1, AP2M1, RILP, GABARAPL2, RAB8B, ATG3, TRAF3IP2, VCP, SEC13, ATG16L2, HDAC6, NPEPL1, CLTA, CLTB, RNF103, NECAP2, VPS16, NUP88, STXBP2, NLRP3, CDK5, ATG4D, TNFSF13B, ATG4B, IPO5, TOM1, KPNA2, COPE, SNX10, LCP2, VPS29, AP1M1, SNX17, TIMM10, NUP93, SEC62, TMED2, TMED3, SNX24, NACA2, HSPA9, RUFY1, HSP90B1, TRPC4AP, VPS28, GGA1, GGA2	
GO:0051340~regulation of ligase activity	7.95E-12	PSMB10, ANAPC10, ANAPC11, PSMA7, PSMB4, PSMF1, PSMB7, PSMB6, PSMB1, PSMD1, PSMB2, PSMD2, PSMD4, UBE2D1, PSMD6, PSMD7, RPS27A, PSMD8, PSMD9, ANAPC5, SKP1, UBE2N, PSMA2, PSMA1, PSMD13, PSMC5, PSMA6, PSME1, PSMC4, PSMA5, PSME2, PSMD11, PSMC3, PSMC2, PSMA4, PSMA3, UBC, UBB, UBA52, UBE2E1	8.57E-10
GO:0006511~ubiquitin-dependent protein catabolic process	1.46E-11	KIAA0368, TSG101, PPP2R5C, BAP1, OS9, CUL3, USP53, PSMD1, PSMD2, PSMD4, PSMD6, USP15, PSMD7, PSMD8, RPS27A, PSMD9, UBE2A,	1.54E-09

		ANAPC5, UFD1L, UBE2I, SKP1, UBE2H, UBE2B, PSMA2, PSMA1, SEC61B, PSMA6, PSME1, UBE2K, PSME2, PSMA5, PSMA4, PSMA3, UBC, MDM2, UCHL3, UBB, UBA52, FBXO11, PSMB10, USP7, DERL2, DERL1, USP8, USP3, RAD23A, ANAPC10, NEDD8, ANAPC11, PSMA7, STUB1, PSMB4, PSMF1, CYLD, ARIH1, UBE2D3, ARIH2, PSMB7, PSMB6, UBE2D2, PSMB1, SQSTM1, USP39, PSMB2, USP36, UBE2D1, FBXO7, UBXN1, RNF144B, ATE1, HSP90B1, PSMC5, PSMD13, VCP, PSMC4, PSMC3, PSMD11, PSMC2, TCEB1, RNF40, UBE2E1	
GO:0006120~mitochondrial electron transport, NADH to ubiquinone	1.50E-11	NDUFB3, NDUFB4, NDUFB5, NDUFB7, NDUFB8, NDUFB9, NDUFAB1, NDUFB1, NDUFB2, NDUFS6, NDUFS5, NDUFS4, NDUFS3, NDUFS2, NDUFA4, NDUFA5, NDUFA2, NDUFA3, NDUFB10, NDUFA9, NDUFA6, NDUFA7, NDUFC2, NDUFC1, NDUFA10, NDUFA1, NDUFV1	1.55E-09
GO:0031396~regulation of protein ubiquitination	1.74E-11	PSMB10, FKBP1A, ANAPC10, ANAPC11, PSMA7, STUB1, PSMB4, PSMF1, PSMB7, PSMB6, PSMB1, PSMD1, PSMB2, PSMD2, PSMD4, UBE2D1, PSMD6, PSMD7, PSMD8, RPS27A, PSMD9, GTPBP4, ANAPC5, SKP1, CDK5, UBE2N, PSMA2, PSMA1, PSMD13, PSMC5, PSMA6, PSME1, PSMC4, PSMA5, PSME2, PSMD11, PSMC3, PSMC2, PSMA4, PSMA3,	1.75E-09

		UBC, UBB, VPS28, UBA52, UBE2E1	
GO:0010608~posttranscriptional regulation of gene expression	2.91E-11	NCBP1, SRP14, RBM3, EIF5, EIF5A, EIF2A, PTEN, YBX1, LUZP6, ASGR2, EIF4EBP1, WIBG, SND1, QKI, EIF1, DNAJC1, EIF2B5, ZFP36, GTPBP4, CCDC88C, MAGOH, NDUFA13, EIF1B, RPS4X, UBE2B, PRKCD, FLNA, HNRNPU, SARNP, EIF4G2, NCK2, EIF4A3, EIF2AK1, EIF4A2, AHSP, VEGFA, CELF1, PPP1R15B, PPP1R15A, MKNK2, PML, IFI30, MAPKAPK2, CALR, ZFP36L1, SRRT, SLC11A1, TNFRSF1B, MOV10, EIF3B, EIF3H, PTK2B, EIF3E, EIF3K, HNRNPD, BCL3, PABPC1, THBS1, FBXO7, DHX9, IL6, PAIP2, SMAD3, ANKHD1, ETF1, RPS5, SELT, COG3, EIF4B, EIF4E, EIF4H, NGDN, EIF4E2	2.87E-09
GO:0008104~protein localization	4.81E-11	OXA1L, CHMP5, USE1, VPS53, RAB1B, RAB1A, CTNNB1, KIF13A, HOMER3, RAB24, RPL11, RAB20, RAN, VTI1B, OPTN, MYH9, YIF1A, VTI1A, CD36, ACAP1, BIN3, RAB13, RAB10, NFKBIE, NFKBIB, NFKBIA, ARF6, PPT1, ARF5, RFFL, CALR, CDC37, CD74, SLC11A1, TMED10, STX11, TRAM1, STX10, RABEP1, ARF3, ARF4, HGS, PDIA3, RAB5C, TSG101, TIMM17A, AP2S1, TIMM17B, CANX, AP1S2, SRPR, COPB1, KDELR1, AGAP3, STX5, STX4, ARHGEF2, STX3, LYN, RAB4B, NDUFA13, FLNA, CHMP1A, CHMP1B,	4.63E-09

		PSEN1, TOMM20, SIL1, TMSB4X, TOMM22, GNAS, GRASP, SERP1, NXT1, BID, YWHAZ, NACA, PML, SFT2D1, NUP214, EZR, SQSTM1, PEX16, EXOC4, RAB11A, BCL3, BCL6, RBM22, RAB2A, GDI1, GDI2, PLEK, YWHAB, YWHAE, GABARAP, COG3, RAB32, RAB31, RPL23, ERBB2IP, AP2A1, GSK3B, RAB34, YWHAQ, NOP58, SSR4, SLC15A3, SSR2, SSR3, GNPTG, SRP14, TSPO, GRPEL1, AP1G2, XPO6, CHMP4B, ATP6AP1, EIF5A, SRP19, IL10, TGFB1, CXCL10, AIP, ATG7, TIMM9, RABGEF1, SAR1B, SAR1A, SCAMP2, ERP29, TMSB10, BCAP31, SEC61B, LYST, SDCBP, PDCD6IP, SEC61G, MVP, CHMP2A, RAB7A, ARFGAP2, DERL2, ARFGAP3, DERL1, COPZ1, SNX2, NAPA, CTSA, NEDD8, SNX4, LMAN2, ABCA1, SNX3, MIA3, TOMM7, TOMM6, TOMM5, SNF8, NPM1, SEC61A1, SRGN, AP2M1, GABARAPL2, RILP, RAB8B, ATG3, TRAF3IP2, VCP, SEC13, ATG16L2, HDAC6, TLN1, NPEPL1, CLTA, CLTB, OS9, CDC42, RNF103, NECAP2, VPS16, NUP88, STXBP2, NLRP3, CDK5, ATG4D, TNFSF13B, ATG4B, IPO5, TOM1, KPNA2, COPE, SNX10, LCP2, VPS29, AP1M1, SNX17, TIMM10, NUP93, SEC62, TMED2, SH3GLB1, TMED3, SNX24, TINF2, NACA2, HSPA9, RUFY1, HSP90B1, BAX, TRPC4AP,	

		VPS28, GGA1, GGA2	
GO:0015980~energy derivation by oxidation of organic compounds	4.93E-11	UQCRC2, OXA1L, UQCRC1, PRKAG2, SNCA, NDUFAB1, NDUFS6, NDUFS5, UQCR11, IDH3G, NDUFS4, NDUFS3, NDUFS2, NDUFB10, SUCLG1, PHKG2, TAZ, CYCS, NDUFC2, NDUFC1, NDUFA10, PPP1CB, ACADVL, PPP1CA, UQCRH, GAA, GNAS, SURF1, MDH2, MDH1, UQCRB, NDUFB3, NDUFB4, NDUFB5, NDUFB7, NDUFB8, NDUFB9, SDHAF2, NDUFB1, NDUFB2, NDUFA4, NDUFA5, NDUFA2, NDUFA3, NDUFA9, NDUFA6, NDUFA7, IDH3B, NDUFA1, SOD2, SDHA, SDHB, PYGL, GSK3B, NDUFV1, SDHC	4.65E-09
GO:0034613~cellular protein localization	1.25E-10	GNPTG, SRP14, TSPO, GRPEL1, OXA1L, AP1G2, XPO6, ATP6AP1, EIF5A, SRP19, TGFB1, AIP, CTNNB1, HOMER3, TIMM9, RPL11, SAR1B, SAR1A, RAN, ERP29, VTI1B, OPTN, VTI1A, BCAP31, SEC61B, SDCBP, SEC61G, DERL2, ARFGAP3, DERL1, COPZ1, SNX2, NFKBIA, NAPA, CTSA, RFFL, CALR, CDC37, CD74, SLC11A1, TOMM7, TOMM5, NPM1, STX11, STX10, TRAM1, SEC61A1, SRGN, AP2M1, VCP, HGS, SEC13, HDAC6, NPEPL1, CLTA, CLTB, PDIA3, AP2S1, TIMM17A, TIMM17B, CDC42, AP1S2, SRPR, COPB1, VPS16, KDELR1, AGAP3, STX5, STX4, ARHGEF2, STX3,	1.15E-08

		NDUFA13, CDK5, FLNA, ATG4D, IPO5, TOMM20, TOM1, SIL1, TOMM22, KPNA2, BID, NXT1, YWHAZ, AP1M1, SNX17, TIMM10, PML, SEC62, NUP214, SH3GLB1, PEX16, EXOC4, RAB11A, BCL3, BCL6, TINF2, HSPA9, RBM22, YWHAB, YWHAE, GABARAP, COG3, ERBB2IP, RPL23, AP2A1, GSK3B, BAX, YWHAQ, TRPC4AP, NOP58, GGA1, SSR4, GGA2, SSR2, SSR3	
GO:0006886~intracellular protein transport	1.35E-10	GNPTG, SRP14, TSPO, GRPEL1, XPO6, AP1G2, ATP6AP1, EIF5A, SRP19, TGFB1, AIP, HOMER3, TIMM9, RPL11, SAR1B, SAR1A, RAN, ERP29, VTI1B, OPTN, VTI1A, BCAP31, SEC61B, SDCBP, SEC61G, DERL2, ARFGAP3, DERL1, COPZ1, SNX2, NFKBIA, NAPA, CTSA, RFFL, CALR, CDC37, CD74, SLC11A1, TOMM7, TOMM5, NPM1, STX11, TRAM1, STX10, SEC61A1, AP2M1, VCP, HGS, SEC13, HDAC6, CLTA, NPEPL1, CLTB, PDIA3, AP2S1, TIMM17A, TIMM17B, AP1S2, SRPR, COPB1, VPS16, KDELR1, AGAP3, STX5, STX4, ARHGEF2, STX3, NDUFA13, CDK5, ATG4D, IPO5, TOMM20, TOM1, SIL1, TOMM22, KPNA2, BID, NXT1, YWHAZ, AP1M1, SNX17, TIMM10, PML, SEC62, NUP214, PEX16, EXOC4, BCL3, RAB11A, BCL6, HSPA9, RBM22, YWHAB, YWHAE, GABARAP, COG3, ERBB2IP, RPL23,	1.23E-08

		AP2A1, GSK3B, YWHAQ, TRPC4AP, NOP58, SSR4, GGA1, GGA2, SSR2, SSR3	
GO:0070727~cellular macromolecule localization	2.03E-10	GNPTG, SRP14, TSPO, GRPEL1, OXA1L, AP1G2, XPO6, ATP6AP1, EIF5A, SRP19, TGFB1, AIP, CTNNB1, HOMER3, TIMM9, RPL11, SAR1B, SAR1A, RAN, ERP29, VTI1B, OPTN, VTI1A, BCAP31, SEC61B, SDCBP, SEC61G, DERL2, ARFGAP3, DERL1, COPZ1, SNX2, NFKBIA, NAPA, CTSA, RFFL, CALR, CDC37, CD74, SLC11A1, TOMM7, TOMM5, NPM1, STX11, STX10, TRAM1, SEC61A1, SRGN, AP2M1, VCP, HGS, SEC13, HDAC6, NPEPL1, CLTA, CLTB, PDIA3, AP2S1, TIMM17A, TIMM17B, CDC42, AP1S2, SRPR, COPB1, VPS16, KDELR1, AGAP3, STX5, STX4, ARHGEF2, STX3, NDUFA13, CDK5, FLNA, ATG4D, IPO5, TOMM20, TOM1, SIL1, TOMM22, KPNA2, BID, NXT1, YWHAZ, AP1M1, SNX17, TIMM10, PML, SEC62, NUP214, SH3GLB1, PEX16, EXOC4, RAB11A, BCL3, BCL6, TINF2, HSPA9, RBM22, YWHAB, YWHAE, GABARAP, COG3, ERBB2IP, RPL23, AP2A1, GSK3B, BAX, YWHAQ, TRPC4AP, NOP58, GGA1, SSR4, GGA2, SSR2, SSR3	1.80E-08
GO:0044093~positive regulation of molecular function	5.03E-10	GNA15, TLR2, PMAIP1, IL10, TGFB1, MYD88, ILK, IL1B, PSENEN, PRKACA, RPS27A, DBNL, C5AR1, ANAPC5, MADD, RELA, PIM1, SKP1, BCL2L13,	4.38E-08

		PPP1CB, PSMA2, SPAG9, PSMA1, PSMA6, PSMA5, F3, PSMA4, PSMA3, EDF1, RIPK2, UBA52, SMARCA4, SIVA1, GNAI2, ADORA2A, MAP4K1, UBE2V1, ANAPC10, ANAPC11, PSMA7, CD74, SLC11A2, NR1H2, SLC11A1, PSMB4, PSMB7, PSMB6, PSMB1, SERINC1, PSMB2, MTCH1, RAC1, NPM1, DIABLO, HSPE1, UBE2D1, FCER1A, GABARAPL2, CARD8, MAP2K3, SPHK1, SMAD3, PTPN11, HDAC5, PSMC5, PSMC4, VCP, PSMC3, PSMC2, PRKAR1A, IKBKB, UBE2E1, GM2A, PRKAG2, PRDX2, ZEB2, SDC4, BUD31, RPS3, CDC42, CXCR4, PSMD1, GTF2A2, TICAM1, PSMD2, PSMD4, CAP1, PAK1, PSMD6, NRG1, PSMD7, PLCB2, PSMD8, PILRB, PSMD9, IRAK2, ICAM1, IRAK1, SP100, SSBP1, CYCS, PKN1, NLRP3, TMEM189, CDK5, NLRP1, UBE2N, ADRB2, TNFRSF10B, CCND3, EREG, PSEN1, PSME1, PSME2, GNB1, UBC, GNAS, UBB, GADD45B, MAP3K11, PSMB10, APH1A, FPR1, PML, FKBP1A, TPM1, GCH1, DGKA, PSMF1, PTK2B, RASGRP4, PYCARD, CD4, CCS, CERK, THBS1, PTPRC, IL6, PLEK, YWHAB, STAT1, SOD1, PSMD13, LRP1, NME1-NME2, PRLR, PSMD11, BAX, IFT57, CALM3, CALM2, ARAP1, CALM1	
GO:0043066~negative regulation of apoptosis	1.17E-09	XRCC5, MEF2C, HRAS, STAT5A, SNCA, IL10,	9.99E-08

		CITED2, MYD88, CDKN2D, ILK, TPT1, IL1B, IL1A, RPS27A, SOCS3, RELA, BCL2A1, PIM1, POLB, PIM3, PIM2, F3, TNFAIP8, VEGFA, RIPK2, TNFAIP3, MYO18A, UBA52, CCL2, MCL1, ADORA2A, CLU, NFKBIA, PPT1, ADA, CD74, PEA15, NPM1, TAF9, CFLAR, BECN1, SPHK1, SMAD3, TAX1BP1, ATF5, HDAC3, CDKN1A, RNF7, HDAC1, IKBKB, IFI6, PRDX5, NFKB1, PRDX2, PTEN, BAG1, CHST11, RHOA, NRG1, CLN3, IRAK1, SERPINB9, PSEN1, TNFSF13B, BTG2, BNIP2, CFL1, UBC, SERPINB2, UBB, PRNP, GSTP1, CSF2, YWHAZ, PF4, TCF7L2, SQSTM1, SH3GLB1, DAD1, BCL3, BCL6, HSPA5, THBS1, ERCC1, HSPA9, IL6, CEBPB, TMBIM6, ANXA1, BIRC6, BIRC3, SOD1, ANXA5, SOD2, HSP90B1, SON, PRLR, NME1-NME2, BAX, GSK3B, BNIP3L	
GO:0043069~negative regulation of programmed cell death	1.23E-09	XRCC5, MEF2C, HRAS, STAT5A, SNCA, IL10, CITED2, MYD88, CDKN2D, ILK, TPT1, IL1B, IL1A, RPS27A, SOCS3, RELA, BCL2A1, PIM1, POLB, PIM3, PIM2, F3, TNFAIP8, VEGFA, RIPK2, TNFAIP3, MYO18A, UBA52, CCL2, MCL1, ADORA2A, CLU, NFKBIA, PPT1, ADA, CD74, PEA15, NPM1, TAF9, CFLAR, BECN1, SPHK1, SMAD3, TAX1BP1, ATF5, HDAC3, CDKN1A, PSMC5, RNF7,	1.03E-07

		HDAC1, IKBKB, IFI6, PRDX5, NFKB1, PRDX2, PTEN, BAG1, CHST11, RHOA, NRG1, CLN3, IRAK1, SERPINB9, PSEN1, TNFSF13B, BTG2, BNIP2, CFL1, UBC, SERPINB2, UBB, PRNP, GSTP1, CSF2, YWHAZ, PF4, TCF7L2, SQSTM1, SH3GLB1, DAD1, BCL3, BCL6, HSPA5, THBS1, ERCC1, HSPA9, IL6, CEBPB, TMBIM6, ANXA1, BIRC6, BIRC3, SOD1, ANXA5, SOD2, HSP90B1, SON, PRLR, NME1-NME2, GSK3B, BAX, BNIP3L	
GO:0060548~negative regulation of cell death	1.45E-09	XRCC5, MEF2C, HRAS, STAT5A, SNCA, IL10, CITED2, MYD88, CDKN2D, ILK, TPT1, IL1B, IL1A, RPS27A, SOCS3, RELA, BCL2A1, PIM1, POLB, PIM3, PIM2, F3, TNFAIP8, VEGFA, RIPK2, TNFAIP3, MYO18A, UBA52, CCL2, MCL1, ADORA2A, CLU, NFKBIA, PPT1, ADA, CD74, PEA15, NPM1, TAF9, CFLAR, BECN1, SPHK1, SMAD3, TAX1BP1, ATF5, HDAC3, CDKN1A, PSMC5, RNF7, HDAC1, IKBKB, IFI6, PRDX5, NFKB1, PRDX2, PTEN, BAG1, CHST11, RHOA, NRG1, CLN3, IRAK1, SERPINB9, PSEN1, TNFSF13B, BTG2, BNIP2, CFL1, UBC, SERPINB2, UBB, PRNP, GSTP1, CSF2, YWHAZ, PF4, TCF7L2, SQSTM1, SH3GLB1, DAD1, BCL3, BCL6, HSPA5, THBS1, ERCC1, HSPA9, IL6, CEBPB, TMBIM6, ANXA1, BIRC6, BIRC3, SOD1, ANXA5,	1.19E-07

		SOD2, HSP90B1, SON, PRLR, NME1-NME2, GSK3B, BAX, BNIP3L	
GO:0009057~macromolecule catabolic process	1.96E-09	NCBP1, PPP2R5C, USE1, ZNRF1, TGFB1, ISG20, CUL3, USP53, ISG15, ATG7, WIBG, RABGEF1, RBCK1, PSENEN, RNF149, RPS27A, AUP1, ANAPC5, SOCS3, MAGOH, RELA, UBR4, UBE2J1, UBE2J2, SKP1, MAP1LC3B2, MYH9, PSMA2, PSMA1, CHID1, CD36, SEC61B, KDM2A, PSMA6, PSMA5, PSMA4, PSMA3, TNFAIP3, UBA52, FBXO11, DERL2, DERL1, RAD23A, PABPC4, UBA7, UBE2V1, ANAPC10, NEDD8, PPT1, CDC34, ANAPC11, RFFL, PSMA7, RBX1, ARIH1, PSMB4, ARIH2, PSMB7, UBE2D3, UBE2D2, PSMB6, PSMB1, EIF3E, PSMB2, HNRNPD, RNF167, UBE2D1, FBXO9, FBXO7, GUSB, UBE2L6, RNPS1, SUGT1, ATG3, ATE1, TXNDC12, URM1, RNF7, PSMC5, PSMC4, VCP, DCP2, PSMC3, UBA1, OTUB1, PSMC2, UBE2E2, HDAC6, UBE2E1, SPG7, KIAA0368, LDLR, TSG101, UFC1, BAP1, NFKB1, SENP5, OS9, MAP1LC3B, CASP8, PSMD1, PSMD2, KLHL21, NSMCE2, PSMD4, DDA1, PSMD6, PSMD7, USP15, DEDD2, PSMD8, PSMD9, ZFP36, CLN3, UBE2A, UFD1L, CCNH, CYCS, UBE2F, HERC4, UBE2I, UBE2H, TMEM189, CDK5, UBE2B, UBE2N, SENP3, EIF4A3, ATG4D, PSEN1, PSME1, UBE2K, PSME2, ATG4B, UBE2M, UBC,	1.58E-07

		GAA, MDM2, UCHL3, UBB, ADAM19, USP7, PSMB10, USP8, UBE2Z, APH1A, USP3, FKBP1A, STUB1, UBE2R2, ZFP36L1, EXOSC10, CYLD, PSMF1, SQSTM1, RNASET2, USP39, USP36, NEDD4L, TPRKB, ERCC1, TRIP12, UBXN1, RNF144B, UBL7, RNASE2, RNASE6, BIRC6, SOD1, MARCH6, UBE2Q1, MANBA, MARCH3, UBL5, WSB1, MARCH2, HSP90B1, PSMD13, KCMF1, PYGL, PSMD11, BAX, TCEB2, TCEB1, RNF19B, UBXN6, RNF40	
GO:0044092~negative regulation of molecular function	2.07E-09	CAST, OXA1L, SNCA, KDM1A, CDKN2D, ILK, IL1B, RPS27A, GTPBP4, ANAPC5, CST3, PIM1, DDIT3, PSMA2, PSMA1, PSMA6, PSMA5, PSMA4, PSMA3, CSTB, ATPIF1, CSTA, UBA52, SIVA1, GNAI2, ADORA2A, NFKBIA, ANAPC10, ANAPC11, PSMA7, PSMB4, PSMB7, PSMB6, PSMB1, PSMB2, UBE2D1, HERPUD1, TAF7, NR4A1, DUSP22, CDKN1A, NOSIP, PSMC5, RGS1, PSMC4, PSMC3, PSMC2, IFI6, DNAJB6, UBE2E1, HDAC6, PRKAG2, PRDX2, NFKB1, PDCD4, RPS3, AES, PSMD1, PSMD2, PSMD4, PSMD6, PSMD7, PSMD8, PSMD9, IRAK2, IRAK1, PPP2R1A, CLN3, SP100, NLRP3, PRKCD, FLNA, LRPAP1, ADRB2, PSEN1, PSME1, PSME2, UBC, UBB, GADD45B, PSMB10, FKBP1A, TRIB1, PSMF1, PPP2CA, HSPA5, PTPRC, PLEK, GPS2, DUSP2, PSMD13, ID2,	1.65E-07

		GSK3A, PSMD11, CALM3, CALM2, CALM1, DUSP6	
GO:0044265~cellular macromolecule catabolic process	3.72E-09	NCBP1, PPP2R5C, ZNRF1, ISG20, CUL3, USP53, ISG15, ATG7, WIBG, RABGEF1, RBCK1, PSENEN, RNF149, RPS27A, AUP1, ANAPC5, SOCS3, MAGOH, RELA, UBR4, UBE2J1, UBE2J2, SKP1, MAP1LC3B2, MYH9, PSMA2, PSMA1, CD36, SEC61B, KDM2A, PSMA6, PSMA5, PSMA4, PSMA3, TNFAIP3, UBA52, FBXO11, DERL2, DERL1, RAD23A, PABPC4, UBA7, UBE2V1, ANAPC10, NEDD8, PPT1, ANAPC11, RFFL, CDC34, PSMA7, RBX1, ARIH1, PSMB4, ARIH2, PSMB7, UBE2D3, UBE2D2, PSMB6, PSMB1, EIF3E, PSMB2, HNRNPD, RNF167, UBE2D1, FBXO9, FBXO7, UBE2L6, RNPS1, SUGT1, ATG3, ATE1, TXNDC12, URM1, RNF7, PSMC5, PSMC4, VCP, DCP2, PSMC3, UBA1, OTUB1, PSMC2, UBE2E2, HDAC6, UBE2E1, KIAA0368, LDLR, TSG101, UFC1, BAP1, NFKB1, SENP5, OS9, MAP1LC3B, CASP8, PSMD1, PSMD2, KLHL21, NSMCE2, PSMD4, DDA1, PSMD6, PSMD7, USP15, DEDD2, PSMD8, PSMD9, ZFP36, UBE2A, UFD1L, CCNH, CYCS, UBE2F, HERC4, UBE2I, UBE2H, TMEM189, UBE2B, UBE2N, SENP3, EIF4A3, ATG4D, PSEN1, PSME1, UBE2K, PSME2, ATG4B, UBE2M, UBC, MDM2, UCHL3, UBB, ADAM19, USP7, PSMB10, USP8, UBE2Z, APH1A,	2.90E-07

		USP3, STUB1, UBE2R2, ZFP36L1, EXOSC10, CYLD, PSMF1, SQSTM1, RNASET2, USP39, USP36, NEDD4L, ERCC1, TRIP12, UBXN1, RNF144B, UBL7, RNASE2, RNASE6, BIRC6, SOD1, MARCH6, UBE2Q1, MARCH3, UBL5, WSB1, MARCH2, HSP90B1, PSMD13, KCMF1, PSMD11, BAX, TCEB2, TCEB1, RNF19B, UBXN6, RNF40	
GO:0010605~negative regulation of macromolecule metabolic process	5.63E-09	MEF2C, SRP14, JDP2, RBM3, SNCA, CBX3, MAF1, IL10, TGFB1, CTNNB1, CITED2, EPC1, KDM1A, EIF4EBP1, SND1, SUPT5H, AKIRIN2, DNAJC1, RPS27A, EIF2B5, GTPBP4, ANAPC5, RELA, MTA2, CST3, MECP2, FOXN3, HES1, PSMA2, PSMA1, EIF2AK1, PSMA6, PSMA5, RPS14, PSMA4, PSMA3, TGIF1, RPS13, CELF1, LRCH4, UBA52, SMARCA4, HMGB2, HUS1, ANAPC10, ANAPC11, CALR, PSMA7, TIMP1, NR1H2, PSMB4, MOV10, PSMB7, PSMB6, PSMB1, DRAP1, EIF3E, PSMB2, NPM1, TAF9, BHLHE40, SKIL, UBE2D1, TCF25, HNRNPAB, IKZF1, PAIP2, KLF10, TAF7, S100A11, SMAD3, SNW1, KAT5, FOXP1, HDAC5, HDAC3, PSMC5, PSMC4, HDAC1, PSMC3, PSMC2, JAZF1, HIVEP1, HDAC9, RBPJ, KLF4, DNAJB6, ADAR, HDAC6, UBE2E1, HSP90AB1, TSG101, SPI1, ZEB2, NFKB1, HSBP1, PDCD4, RPS3, AES, PSMD1, PSMD2, PSMD4, LRRFIP1, PSMD6, NRG1,	4.32E-07

		PSMD7, DEDD2, PSMD8, PSMD9, PPP2R1A, CLN3, SP100, ARID5A, NDUFA13, UBE2I, FOSB, RBBP7, MBD2, CDK5, PRKCD, FLNA, EIF4A3, CHMP1A, EREG, PSEN1, PSME1, PSME2, UBC, MDM2, UBB, CUX1, KDM6B, PSMB10, SBNO2, BCLAF1, PML, FKBP1A, PF4, SRRT, PSMF1, CIR1, SET, PPP2CA, PER1, BCL3, BCL6, SUPT4H1, THBS1, TINF2, ERCC1, ENO1, PTPRC, IL6, JARID2, TRIM28, YWHAB, ILF3, ANKHD1, YWHAE, STAT3, GPS2, ATXN1, PSMD13, ID2, GSK3A, PSMD11, PHB2, BAX, IRF7, BNIP3L, IRF8, YWHAQ, HBEGF, IRF2, VPS28	
GO:0030163~protein catabolic process	6.44E-09	PPP2R5C, USE1, ZNRF1, CUL3, USP53, ISG15, ATG7, RABGEF1, RBCK1, PSENEN, RNF149, RPS27A, AUP1, ANAPC5, SOCS3, RELA, UBE2J1, UBR4, UBE2J2, SKP1, MAP1LC3B2, MYH9, PSMA2, PSMA1, SEC61B, KDM2A, PSMA6, PSMA5, PSMA4, PSMA3, TNFAIP3, UBA52, FBXO11, DERL2, DERL1, RAD23A, UBA7, UBE2V1, ANAPC10, NEDD8, PPT1, ANAPC11, RFFL, CDC34, PSMA7, RBX1, ARIH1, PSMB4, PSMB7, ARIH2, UBE2D3, PSMB6, UBE2D2, PSMB1, PSMB2, RNF167, UBE2D1, FBXO9, FBXO7, UBE2L6, SUGT1, ATG3, ATE1, URM1, RNF7, PSMC5, PSMC4, VCP, PSMC3, OTUB1, UBA1, PSMC2, UBE2E2, HDAC6, UBE2E1,	4.86E-07

		SPG7, KIAA0368, TSG101, UFC1, BAP1, NFKB1, SENP5, OS9, MAP1LC3B, PSMD1, CASP8, PSMD2, KLHL21, NSMCE2, PSMD4, DDA1, PSMD6, PSMD7, USP15, PSMD8, PSMD9, CLN3, UBE2A, UFD1L, HERC4, UBE2F, UBE2I, UBE2H, TMEM189, UBE2B, UBE2N, SENP3, ATG4D, PSEN1, PSME1, UBE2K, PSME2, ATG4B, UBE2M, UBC, MDM2, UCHL3, UBB, ADAM19, USP7, PSMB10, USP8, UBE2Z, APH1A, USP3, STUB1, UBE2R2, CYLD, PSMF1, SQSTM1, USP39, USP36, NEDD4L, TPRKB, TRIP12, UBXN1, UBL7, RNF144B, BIRC6, MARCH6, UBE2Q1, MARCH3, UBL5, WSB1, MARCH2, HSP90B1, PSMD13, KCMF1, PSMD11, TCEB2, RNF19B, TCEB1, UBXN6, RNF40	
GO:0043065~positive regulation of apoptosis	9.55E-09	PTGS2, RBM5, TLR2, TNFSF14, EIF5A, RPS27L, TNFSF13, PMAIP1, SART1, IL10, TGFB1, CRADD, CTNNBL1, CUL3, CD44, IL1B, PSENEN, RPL11, DAP, MX1, RPS27A, BCL2L13, BCL2L11, DDIT3, TIAL1, LYST, RIPK2, UBA52, SIVA1, ADORA2A, STK17B, AKAP13, SRC, RAC1, MTCH1, DIABLO, RUNX3, TRAF3, B4GALT1, CFLAR, ABR, TM2D1, KLF10, SMAD3, NR4A1, SLAMF7, RPS6, VAV1, TXNDC12, TNFSF10, CDKN1A, RNF7, PDCD5, PDCD6, HDAC6, PDIA3, PTEN, PRDX1, RPS3, ARHGAP4, CASP4,	7.09E-07

		DYNLL1, RPS3A, CASP8, TICAM1, CASP1, NDUFS3, FOSL1, DEDD2, PPP2R1A, ARHGEF2, NDUFA13, IFI16, NLRP3, PRKCE, CDK5, NLRP1, TNFRSF9, ADRB2, TNFRSF10B, PSEN1, UBC, UBB, MAP3K11, BID, BCLAF1, APH1A, PML, NR3C1, ITM2B, GCH1, PLEKHG2, SQSTM1, SH3GLB1, PPP2CA, PYCARD, PPP3CC, BCL3, BCL6, PHLDA1, PTPRC, CEBPB, YWHAB, BAD, SAP30BP, SOD1, STAT1, YWHAE, TNFSF8, DUSP1, BAX, BNIP3L, SMPD2, DNM2	
GO:0051603~proteolysis involved in cellular protein catabolic process	9.70E-09	PPP2R5C, ZNRF1, CUL3, USP53, ISG15, ATG7, RABGEF1, RBCK1, PSENEN, RNF149, RPS27A, AUP1, ANAPC5, SOCS3, RELA, UBE2J1, UBR4, UBE2J2, SKP1, MAP1LC3B2, MYH9, PSMA2, PSMA1, SEC61B, KDM2A, PSMA6, PSMA5, PSMA4, PSMA3, TNFAIP3, UBA52, FBXO11, DERL2, DERL1, RAD23A, UBA7, UBE2V1, ANAPC10, NEDD8, ANAPC11, RFFL, CDC34, PSMA7, RBX1, ARIH1, PSMB4, PSMB7, ARIH2, UBE2D3, PSMB6, UBE2D2, PSMB1, PSMB2, RNF167, UBE2D1, FBXO9, FBXO7, UBE2L6, SUGT1, ATG3, ATE1, URM1, RNF7, PSMC5, PSMC4, VCP, PSMC3, OTUB1, UBA1, PSMC2, UBE2E2, HDAC6, UBE2E1, KIAA0368, TSG101, UFC1, BAP1, NFKB1, SENP5, OS9, MAP1LC3B, PSMD1, CASP8, PSMD2, KLHL21, NSMCE2, PSMD4, DDA1,	7.08E-07

		PSMD6, PSMD7, USP15, PSMD8, PSMD9, UBE2A, UFD1L, HERC4, UBE2F, UBE2I, UBE2H, TMEM189, UBE2B, UBE2N, SENP3, ATG4D, PSEN1, PSME1, UBE2K, PSME2, ATG4B, UBE2M, UBC, MDM2, UCHL3, UBB, ADAM19, USP7, PSMB10, USP8, UBE2Z, APH1A, USP3, STUB1, UBE2R2, CYLD, PSMF1, SQSTM1, USP39, USP36, NEDD4L, TRIP12, UBXN1, UBL7, RNF144B, BIRC6, MARCH6, UBE2Q1, MARCH3, UBL5, WSB1, MARCH2, HSP90B1, PSMD13, KCMF1, PSMD11, TCEB2, RNF19B, TCEB1, UBXN6, RNF40	
GO:0015985~energy coupled proton transport, down electrochemical gradient	1.27E-08	ATP5D, TCIRG1, ATP5E, ATP6V0E1, ATP5B, ATP6AP1, ATP5F1, ATP6V1H, ATP5G2, ATP6V1B2, ATP5G1, ATP5G3, ATP6V0B, ATP6V1F, ATP6V0C, ATP6V1E1, ATP5C1, ATP5L, ATP5O, ATP5A1, ATP5I, ATP5H, ATP5J	9.11E-07
GO:0015986~ATP synthesis coupled proton transport	1.27E-08	ATP5D, TCIRG1, ATP5E, ATP6V0E1, ATP5B, ATP6AP1, ATP5F1, ATP6V1H, ATP5G2, ATP6V1B2, ATP5G1, ATP5G3, ATP6V0B, ATP6V1F, ATP6V0C, ATP6V1E1, ATP5C1, ATP5L, ATP5O, ATP5A1, ATP5I, ATP5H, ATP5J	9.11E-07
GO:0044257~cellular protein catabolic process	1.37E-08	PPP2R5C, ZNRF1, CUL3, USP53, ISG15, ATG7, RABGEF1, RBCK1, PSENEN, RNF149, RPS27A, AUP1, ANAPC5, SOCS3, RELA, UBE2J1, UBR4, UBE2J2, SKP1, MAP1LC3B2, MYH9,	9.72E-07

		PSMA2, PSMA1, SEC61B, KDM2A, PSMA6, PSMA5, PSMA4, PSMA3, TNFAIP3, UBA52, FBXO11, DERL2, DERL1, RAD23A, UBA7, UBE2V1, ANAPC10, NEDD8, ANAPC11, RFFL, CDC34, PSMA7, RBX1, ARIH1, PSMB4, PSMB7, ARIH2, UBE2D3, PSMB6, UBE2D2, PSMB1, PSMB2, RNF167, UBE2D1, FBXO9, FBXO7, UBE2L6, SUGT1, ATG3, ATE1, URM1, RNF7, PSMC5, PSMC4, VCP, PSMC3, OTUB1, UBA1, PSMC2, UBE2E2, HDAC6, UBE2E1, KIAA0368, TSG101, UFC1, BAP1, NFKB1, SENP5, OS9, MAP1LC3B, PSMD1, CASP8, PSMD2, KLHL21, NSMCE2, PSMD4, DDA1, PSMD6, PSMD7, USP15, PSMD8, PSMD9, UBE2A, UFD1L, HERC4, UBE2F, UBE2I, UBE2H, TMEM189, UBE2B, UBE2N, SENP3, ATG4D, PSEN1, PSME1, UBE2K, PSME2, ATG4B, UBE2M, UBC, MDM2, UCHL3, UBB, ADAM19, USP7, PSMB10, USP8, UBE2Z, APH1A, USP3, STUB1, UBE2R2, CYLD, PSMF1, SQSTM1, USP39, USP36, NEDD4L, TRIP12, UBXN1, UBL7, RNF144B, BIRC6, MARCH6, UBE2Q1, MARCH3, UBL5, WSB1, MARCH2, HSP90B1, PSMD13, KCMF1, PSMD11, TCEB2, RNF19B, TCEB1, UBXN6, RNF40	
GO:0043068~positive regulation of programmed cell death	1.45E-08	PTGS2, RBM5, TLR2, TNFSF14, EIF5A, RPS27L, TNFSF13, PMAIP1, SART1, IL10, TGFB1, CRADD, CTNNBL1, CUL3,	1.01E-06

		CD44, IL1B, PSENEN, RPL11, DAP, MX1, RPS27A, BCL2L13, BCL2L11, DDIT3, TIAL1, LYST, RIPK2, UBA52, SIVA1, ADORA2A, STK17B, AKAP13, SRC, RAC1, MTCH1, DIABLO, RUNX3, TRAF3, B4GALT1, CFLAR, ABR, TM2D1, KLF10, SMAD3, NR4A1, SLAMF7, RPS6, VAV1, TXNDC12, TNFSF10, CDKN1A, RNF7, PDCD5, PDCD6, HDAC6, PDIA3, PTEN, PRDX1, RPS3, ARHGAP4, CASP4, DYNLL1, RPS3A, CASP8, TICAM1, CASP1, NDUFS3, FOSL1, DEDD2, PPP2R1A, ARHGEF2, NDUFA13, IFI16, NLRP3, PRKCE, CDK5, NLRP1, TNFRSF9, ADRB2, TNFRSF10B, PSEN1, UBC, UBB, MAP3K11, BID, BCLAF1, APH1A, PML, NR3C1, ITM2B, GCH1, PLEKHG2, SQSTM1, SH3GLB1, PPP2CA, PYCARD, PPP3CC, BCL3, BCL6, PHLDA1, PTPRC, CEBPB, YWHAB, BAD, SAP30BP, SOD1, STAT1, YWHAE, TNFSF8, DUSP1, BAX, BNIP3L, SMPD2, DNM2	
GO:0031399~regulation of protein modification process	1.68E-08	PRKAG2, SNCA, ZEB2, PDCD4, TGFB1, NDUFS4, ATG7, PSMD1, PSMD2, IL1B, PSMD4, PAK1, PSMD6, PSMD7, RPS27A, PSMD8, PSMD9, PPP2R1A, DBNL, GTPBP4, LYN, ANAPC5, SOCS3, CCDC88C, PKN1, TNFRSF14, SKP1, PRKCE, PRKCD, CDK5, UBE2N, OSM, PSMA2, PSMA1, PSMA6, PSME1, PSEN1, CCND3, PSME2, PSMA5,	1.15E-06

		PSMA4, PSMA3, UBC, UBB, UBA52, MAP3K11, PSMB10, CSF2, PML, MAP4K1, ANAPC10, FKBP1A, ITGB2, ANAPC11, PSMA7, STUB1, PSMB4, PSMF1, PSMB7, SET, PSMB6, PSMB1, PTK2B, PPP2CA, PSMB2, CD4, UBE2D1, TINF2, FCER1A, PTPRC, IL6, HCLS1, TAF7, YWHAB, YWHAE, PSMC5, PSMD13, PSMC4, PSMD11, PSMC3, PSMC2, BAX, PRKAR1A, VPS28, UBE2E1	
GO:0010942~positive regulation of cell death	1.91E-08	PTGS2, RBM5, TLR2, TNFSF14, EIF5A, RPS27L, TNFSF13, PMAIP1, SART1, IL10, TGFB1, CRADD, CTNNBL1, CUL3, CD44, IL1B, PSENEN, RPL11, DAP, MX1, RPS27A, BCL2L13, BCL2L11, DDIT3, TIAL1, LYST, RIPK2, UBA52, SIVA1, ADORA2A, STK17B, AKAP13, SRC, RAC1, MTCH1, DIABLO, RUNX3, TRAF3, B4GALT1, CFLAR, ABR, TM2D1, KLF10, SMAD3, NR4A1, SLAMF7, RPS6, VAV1, TXNDC12, TNFSF10, CDKN1A, RNF7, PDCD5, PDCD6, HDAC6, PDIA3, PTEN, PRDX1, RPS3, ARHGAP4, CASP4, DYNLL1, RPS3A, CASP8, TICAM1, CASP1, NDUFS3, FOSL1, DEDD2, PPP2R1A, ARHGEF2, NDUFA13, IFI16, NLRP3, PRKCE, CDK5, NLRP1, TNFRSF9, ADRB2, TNFRSF10B, PSEN1, UBC, UBB, MAP3K11, BID, BCLAF1, APH1A, PML, NR3C1, ITM2B, GCH1, PLEKHG2,	1.29E-06

		SQSTM1, SH3GLB1, PPP2CA, PYCARD, PPP3CC, BCL3, BCL6, PHLDA1, PTPRC, CEBPB, YWHAB, BAD, SAP30BP, SOD1, STAT1, YWHAE, TNFSF8, DUSP1, BAX, BNIP3L, SMPD2, DNM2	
GO:0043085~positive regulation of catalytic activity	2.01E-08	GNA15, PMAIP1, TGFB1, ILK, IL1B, PSENEN, PRKACA, RPS27A, DBNL, C5AR1, ANAPC5, MADD, PIM1, SKP1, BCL2L13, PPP1CB, PSMA2, SPAG9, PSMA1, PSMA6, PSMA5, F3, PSMA4, PSMA3, UBA52, SIVA1, GNAI2, ADORA2A, MAP4K1, ANAPC10, ANAPC11, PSMA7, CD74, SLC11A2, NR1H2, SLC11A1, PSMB4, PSMB7, PSMB6, PSMB1, SERINC1, PSMB2, MTCH1, RAC1, NPM1, DIABLO, HSPE1, UBE2D1, FCER1A, GABARAPL2, CARD8, MAP2K3, SPHK1, SMAD3, PTPN11, PSMC5, PSMC4, VCP, PSMC3, PSMC2, PRKAR1A, UBE2E1, GM2A, PRKAG2, PRDX2, ZEB2, SDC4, BUD31, RPS3, CDC42, CXCR4, PSMD1, PSMD2, PSMD4, CAP1, PAK1, PSMD6, NRG1, PSMD7, PLCB2, PILRB, PSMD8, PSMD9, IRAK1, SSBP1, CYCS, PKN1, NLRP3, CDK5, NLRP1, UBE2N, ADRB2, TNFRSF10B, EREG, PSEN1, PSME1, CCND3, PSME2, GNB1, UBC, GNAS, UBB, GADD45B, MAP3K11, PSMB10, APH1A, FPR1, PML, TPM1, GCH1, DGKA, PSMF1, PTK2B, RASGRP4, PYCARD, CCS, CD4, CERK, THBS1,	1.34E-06

		PTPRC, PLEK, YWHAB, STAT1, SOD1, LRP1, PSMD13, PRLR, PSMD11, BAX, IFT57, ARAP1	
GO:0045321~leukocyte activation	2.52E-08	NBN, TOLLIP, STAT5A, SNCA, TLR2, TNFSF14, PRDX2, IL15, TGFB1, IL10, CTNNB1, CD48, CD93, CXCR4, TICAM1, IMPDH1, IMPDH2, RHOH, ICAM1, BST2, CD3D, LYN, RELB, STXBP2, MINK1, MYH9, WAS, PRKCD, NCK2, LAT2, CD86, CCND3, PSEN1, IGBP1, RIPK2, LCP1, LCP2, CSF2, GPR183, SBNO2, YWHAZ, FKBP1A, ITGAM, CD74, ADA, HSH2D, SLC11A1, DOCK2, IL23A, BCL11A, BCL3, BCL6, CD4, ERCC1, PTPRC, KLF6, IKZF1, IL8, SMAD3, MYO1F, SLAMF7, VAV1, FOXP1, HDAC5, LAT, PRLR, FYN, RPL22, JMJD6, BAX, IRF1, CLEC7A, HDAC9	1.65E-06
GO:0032270~positive regulation of cellular protein metabolic process	2.54E-08	AURKAIP1, PRKAG2, EIF5A, TGFB1, WIBG, ATG7, PSMD1, PSMD2, IL1B, PSMD4, PSMD6, PSMD7, PSMD8, RPS27A, EIF2B5, PSMD9, LYN, ANAPC5, NRD1, TNFRSF14, SKP1, RPS4X, UBE2N, OSM, PSMA2, PSMA1, PSMA6, PSME1, CCND3, PSEN1, PSMA5, PSME2, PSMA4, PSMA3, UBC, MDM2, UBB, UBA52, PSMB10, CSF2, PML, NFKBIA, ANAPC10, FKBP1A, ANAPC11, PSMA7, STUB1, NR1H2, PSMB4, PSMF1, PSMB7, PSMB6, PSMB1, PTK2B, PSMB2, PEMT, BCL3, CD4, UBE2D1, THBS1, FCER1A, IL6, HCLS1,	1.65E-06

		PSMC5, PSMD13, PSMC4, PSMC3, PSMD11, PSMC2, SH3D19, UBE2E1	
GO:0022613~ribonucleoprotein complex biogenesis	2.62E-08	EIF6, NAF1, NCBP1, RPL14, SNRPD3, RBM5, SNRPD1, SNRPD2, EIF2A, DKC1, RPLP0, RPL11, GNL2, FTSJ1, IMP4, CLNS1A, RPL35A, GTPBP4, EXOSC8, SF1, MBNL1, EXOSC1, EIF4A3, PIH1D1, RPS19, RPS16, RPS14, RPS15, SNRPB, SLU7, CELF1, SNRPC, SNRPF, SNRPE, SNRPG, NHP2L1, UTP6, EXOSC10, RPS28, RPL7, FRG1, USP39, NPM1, RPL5, RPL7A, RSL24D1, NSA2, RPS24, RPL26, RPL24, RPS6, NOP10, FBL, RPS7, DDX56, NOP58, NOP56, POP4, TXNL4A	1.67E-06
GO:0030036~actin cytoskeleton organization	3.76E-08	MTSS1, HRAS, TLN1, PDLIM7, S100A9, CAPZA1, RHOQ, RHOU, ACTG1, CDC42, DYNLL1, GSN, RHOA, RALA, CAP1, RHOF, FMNL1, ARHGEF2, ACTN1, TMSB10, MYH9, WAS, CDK5, VASP, FLNA, ARHGAP26, ELMO1, ARPC1A, EPB41L3, NCK2, ATP2C1, CFL1, SDCBP, BIN3, TMSB4X, FHOD1, LCP1, ALDOA, CXCL1, DIAPH1, SSH2, FHL3, NEDD9, MYO9B, ARPC4, ARF6, CYTH2, ARPC5, CALR, TPM1, CAPZB, PFN1, DOCK2, EZR, RAC2, INPP5K, PTK2B, RAC1, CNN2, PAFAH1B1, BCL6, SH2B2, TRIP10, ARHGDIB, PLEK, EVL, GAS7, CORO1A, ARAP1	2.36E-06
GO:0001775~cell activation	4.63E-08	NBN, TOLLIP, STAT5A, SNCA, TLR2, TNFSF14, PRDX2, NFKB2, IL15, IL10, TGFB1, CTNNB1,	2.87E-06

		CD48, CD93, CXCR4, TICAM1, IMPDH1, IMPDH2, RHOH, ICAM1, BST2, CD3D, LYN, RELB, STXBP2, MINK1, MYH9, WAS, PRKCD, NCK2, LAT2, CD86, PSEN1, CCND3, IGBP1, RIPK2, LCP1, LCP2, CSF2, GPR183, SBNO2, YWHAZ, ADORA2A, FKBP1A, PF4, ITGAM, ADA, CD74, HSH2D, CD9, SLC11A1, DOCK2, IL23A, BCL11A, BCL3, BCL6, CD4, ERCC1, PTPRC, KLF6, IL6, PLEK, IKZF1, IL8, SMAD3, MYO1F, SLAMF7, VAV1, FOXP1, HDAC5, P2RX4, PLSCR1, LAT, PRLR, JMJD6, FYN, RPL22, BAX, IRF1, CLEC7A, TREML1, HDAC9	
GO:0034220~ion transmembrane transport	5.51E-08	ATP5D, ATP5E, ATP6V0E1, ATP6AP1, ATP5B, ATP5G2, ATP6V1B2, ATP5G1, ATP5G3, ATP6V0B, SLC11A2, ATP6V0C, SLC11A1, ATP5L, ATP5O, ATP5I, ATP5H, ATP5J, TCIRG1, ATP5F1, ATP6V1H, ATP6V1F, ATP6V1E1, ATP5C1, ATP5A1	3.37E-06
GO:0006897~endocytosis	6.73E-08	HRAS, PDLIM7, LDLR, ATP5B, SNCA, ASGR2, PICALM, AP1S2, CD93, RABGEF1, NECAP2, CAP1, CLINT1, CLN3, DBNL, ATP6V1H, PI4KB, CDK5, M6PR, ELMO1, ADRB2, CD36, ARRB2, RIN2, TOM1, BIN1, RIN3, RAB7A, ADORA2A, SNX17, SNX2, CDC42SE1, PPT1, CYTH2, SNX4, ABCA1, SNX3, CLEC10A, SLC11A1, FCN1, RAC1, FCER1G, TRIP10, PTX3,	4.06E-06

		EHD1, THBS1, EHD4, HCK, DENND1A, RUFY1, VAV1, MARCH3, CORO1C, MARCH2, LMBR1L, CORO1A, LRP1, RABEP1, NME1-NME2, CDC42SE2, JMJD6, AP2A1, CXCL16, RAB34, CLEC7A, CD14, DNM2	
GO:0010324~membrane invagination	6.73E-08	HRAS, PDLIM7, LDLR, ATP5B, SNCA, ASGR2, PICALM, AP1S2, CD93, RABGEF1, NECAP2, CAP1, CLINT1, CLN3, DBNL, ATP6V1H, PI4KB, CDK5, M6PR, ELMO1, ADRB2, CD36, ARRB2, RIN2, TOM1, BIN1, RIN3, RAB7A, ADORA2A, SNX17, SNX2, CDC42SE1, PPT1, CYTH2, SNX4, ABCA1, SNX3, CLEC10A, SLC11A1, FCN1, RAC1, FCER1G, TRIP10, PTX3, EHD1, THBS1, EHD4, HCK, DENND1A, RUFY1, VAV1, MARCH3, CORO1C, MARCH2, LMBR1L, CORO1A, LRP1, RABEP1, NME1-NME2, CDC42SE2, JMJD6, AP2A1, CXCL16, RAB34, CLEC7A, CD14, DNM2	4.06E-06
GO:0051247~positive regulation of protein metabolic process	7.01E-08	AURKAIP1, PRKAG2, EIF5A, TGFB1, WIBG, ATG7, PSMD1, PSMD2, IL1B, PSMD4, PSMD6, PSMD7, PSMD8, RPS27A, EIF2B5, PSMD9, LYN, ANAPC5, NRD1, TNFRSF14, SKP1, RPS4X, UBE2N, OSM, PSMA2, PSMA1, PSMA6, PSME1, CCND3, PSEN1, PSMA5, PSME2, PSMA4, PSMA3, UBC, MDM2, UBB, UBA52, PSMB10, CSF2, PML, NFKBIA, EGLN2, ANAPC10, FKBP1A, ANAPC11, PSMA7,	4.18E-06

		STUB1, NR1H2, PSMB4, PSMF1, PSMB7, PSMB6, PSMB1, PTK2B, PSMB2, PEMT, BCL3, CD4, UBE2D1, THBS1, FCER1A, IL6, HCLS1, PSMC5, PSMD13, PSMC4, PSMC3, PSMD11, PSMC2, SH3D19, UBE2E1	
GO:0052547~regulation of peptidase activity	8.82E-08	SIVA1, ADORA2A, MGMT, SNCA, PML, PMAIP1, RPS3, SLC11A2, CDKN2D, MTCH1, PYCARD, DIABLO, HSPE1, HSPA5, CARD8, HERPUD1, CYCS, CST3, SMAD3, NRD1, NR4A1, BAD, STAT1, BCL2L13, NLRP3, NLRP1, TNFRSF10B, VCP, BAX, F3, IFT57, CSTB, CSTA, IFI6, DNAJB6	5.18E-06
GO:0019941~modification-dependent protein catabolic process	9.47E-08	PPP2R5C, ZNRF1, CUL3, USP53, ISG15, ATG7, RABGEF1, RBCK1, RNF149, RPS27A, AUP1, ANAPC5, SOCS3, UBE2J1, UBR4, UBE2J2, SKP1, MAP1LC3B2, PSMA2, PSMA1, SEC61B, KDM2A, PSMA6, PSMA5, PSMA4, PSMA3, TNFAIP3, UBA52, FBXO11, DERL2, DERL1, RAD23A, UBA7, UBE2V1, ANAPC10, NEDD8, ANAPC11, RFFL, CDC34, PSMA7, RBX1, ARIH1, PSMB4, PSMB7, ARIH2, UBE2D3, PSMB6, UBE2D2, PSMB1, PSMB2, RNF167, UBE2D1, FBXO9, FBXO7, UBE2L6, SUGT1, ATG3, ATE1, URM1, RNF7, PSMC5, PSMC4, VCP, OTUB1, UBA1, PSMC3, PSMC2, UBE2E2, UBE2E1, KIAA0368, TSG101, UFC1, BAP1, SENP5, OS9, MAP1LC3B, PSMD1, PSMD2, KLHL21,	5.50E-06

		NSMCE2, PSMD4, DDA1, PSMD6, PSMD7, USP15, PSMD8, PSMD9, UBE2A, UFD1L, HERC4, UBE2F, UBE2I, UBE2H, TMEM189, UBE2B, UBE2N, SENP3, ATG4D, PSME1, UBE2K, PSME2, ATG4B, UBE2M, UBC, MDM2, UCHL3, UBB, USP7, PSMB10, USP8, UBE2Z, USP3, STUB1, UBE2R2, CYLD, PSMF1, SQSTM1, USP39, USP36, NEDD4L, TRIP12, UBXN1, UBL7, RNF144B, BIRC6, MARCH6, UBE2Q1, MARCH3, UBL5, WSB1, MARCH2, HSP90B1, PSMD13, KCMF1, PSMD11, TCEB2, RNF19B, TCEB1, UBXN6, RNF40	
GO:0043632~modification-dependent macromolecule catabolic process	9.47E-08	PPP2R5C, ZNRF1, CUL3, USP53, ISG15, ATG7, RABGEF1, RBCK1, RNF149, RPS27A, AUP1, ANAPC5, SOCS3, UBE2J1, UBR4, UBE2J2, SKP1, MAP1LC3B2, PSMA2, PSMA1, SEC61B, KDM2A, PSMA6, PSMA5, PSMA4, PSMA3, TNFAIP3, UBA52, FBXO11, DERL2, DERL1, RAD23A, UBA7, UBE2V1, ANAPC10, NEDD8, ANAPC11, RFFL, CDC34, PSMA7, RBX1, ARIH1, PSMB4, PSMB7, ARIH2, UBE2D3, PSMB6, UBE2D2, PSMB1, PSMB2, RNF167, UBE2D1, FBXO9, FBXO7, UBE2L6, SUGT1, ATG3, ATE1, URM1, RNF7, PSMC5, PSMC4, VCP, OTUB1, UBA1, PSMC3, PSMC2, UBE2E2, UBE2E1, KIAA0368, TSG101, UFC1, BAP1, SENP5, OS9, MAP1LC3B, PSMD1, PSMD2, KLHL21,	5.50E-06

		NSMCE2, PSMD4, DDA1, PSMD6, PSMD7, USP15, PSMD8, PSMD9, UBE2A, UFD1L, HERC4, UBE2F, UBE2I, UBE2H, TMEM189, UBE2B, UBE2N, SENP3, ATG4D, PSME1, UBE2K, PSME2, ATG4B, UBE2M, UBC, MDM2, UCHL3, UBB, USP7, PSMB10, USP8, UBE2Z, USP3, STUB1, UBE2R2, CYLD, PSMF1, SQSTM1, USP39, USP36, NEDD4L, TRIP12, UBXN1, UBL7, RNF144B, BIRC6, MARCH6, UBE2Q1, MARCH3, UBL5, WSB1, MARCH2, HSP90B1, PSMD13, KCMF1, PSMD11, TCEB2, RNF19B, TCEB1, UBXN6, RNF40	
GO:0043122~regulation of I-kappaB kinase/NF-kappaB cascade	1.36E-07	SLC44A2, VAPA, LITAF, PPM1A, UBE2V1, FKBP1A, TNFRSF1A, MYD88, TMEM9B, SLC35B2, SQSTM1, CASP8, SHISA5, TICAM1, RHOA, IL1B, RHOC, CASP1, RHOH, SECTM1, CFLAR, CARD8, BST2, C9ORF89, RELA, LGALS1, PIM2, TMEM189, BIRC2, LGALS9, FLNA, UBE2N, TRAF3IP2, APOL3, TNFSF10, TNFRSF10B, ATP2C1, RIPK2, TNFAIP3, IKBKB, EEF1D	7.80E-06
GO:0043086~negative regulation of catalytic activity	1.96E-07	CAST, OXA1L, SNCA, PRKAG2, NFKB1, PDCD4, CDKN2D, ILK, PSMD1, PSMD2, IL1B, PSMD4, PSMD6, PSMD7, PSMD8, RPS27A, PSMD9, CLN3, PPP2R1A, ANAPC5, CST3, FLNA, PSMA2, PSMA1, PSMA6, PSME1, PSEN1, PSMA5, PSME2, PSMA4, PSMA3, UBC, CSTB, ATPIF1, UBB, CSTA,	1.11E-05

		GADD45B, UBA52, PSMB10, GNAI2, ADORA2A, ANAPC10, FKBP1A, ANAPC11, PSMA7, TRIB1, PSMB4, PSMF1, PSMB7, PSMB6, PSMB1, PPP2CA, PSMB2, HSPA5, UBE2D1, PTPRC, HERPUD1, PLEK, TAF7, DUSP22, NR4A1, GPS2, NOSIP, CDKN1A, RGS1, PSMC5, PSMD13, DUSP2, PSMC4, PSMC3, GSK3A, PSMD11, PSMC2, IFI6, DNAJB6, DUSP6, HDAC6, UBE2E1	
GO:0006917~induction of apoptosis	1.98E-07	TLR2, TNFSF14, EIF5A, TNFSF13, RPS27L, PMAIP1, PTEN, PRDX1, CRADD, TGFB1, SART1, RPS3, ARHGAP4, CUL3, CASP4, RPS3A, TICAM1, CASP8, PSENEN, RPL11, DAP, NDUFS3, MX1, CASP1, DEDD2, RPS27A, PPP2R1A, ARHGEF2, NDUFA13, IFI16, NLRP3, PRKCE, BCL2L13, NLRP1, BCL2L11, TNFRSF9, TNFRSF10B, PSEN1, TIAL1, LYST, UBC, UBB, UBA52, SIVA1, BID, BCLAF1, APH1A, ADORA2A, STK17B, PML, AKAP13, ITM2B, GCH1, PLEKHG2, SH3GLB1, SQSTM1, PPP2CA, RAC1, PYCARD, DIABLO, BCL3, RUNX3, PHLDA1, TRAF3, CFLAR, CEBPB, ABR, TM2D1, KLF10, SMAD3, NR4A1, BAD, SAP30BP, SLAMF7, STAT1, YWHAE, VAV1, TNFSF8, TXNDC12, CDKN1A, TNFSF10, RNF7, BAX, BNIP3L, PDCD5, PDCD6, SMPD2	1.10E-05
GO:0012502~induction of programmed cell death	2.29E-07	TLR2, TNFSF14, EIF5A, TNFSF13, RPS27L,	1.26E-05

		PMAIP1, PTEN, PRDX1, CRADD, TGFB1, SART1, RPS3, ARHGAP4, CUL3, CASP4, RPS3A, TICAM1, CASP8, PSENEN, RPL11, DAP, NDUFS3, MX1, CASP1, DEDD2, RPS27A, PPP2R1A, ARHGEF2, NDUFA13, IFI16, NLRP3, PRKCE, BCL2L13, NLRP1, BCL2L11, TNFRSF9, TNFRSF10B, PSEN1, TIAL1, LYST, UBC, UBB, UBA52, SIVA1, BID, BCLAF1, APH1A, ADORA2A, STK17B, PML, AKAP13, ITM2B, GCH1, PLEKHG2, SH3GLB1, SQSTM1, PPP2CA, RAC1, PYCARD, DIABLO, BCL3, RUNX3, PHLDA1, TRAF3, CFLAR, CEBPB, ABR, TM2D1, KLF10, SMAD3, NR4A1, BAD, SAP30BP, SLAMF7, STAT1, YWHAE, VAV1, TNFSF8, TXNDC12, CDKN1A, TNFSF10, RNF7, BAX, BNIP3L, PDCD5, PDCD6, SMPD2	
GO:0030029~actin filament-based process	2.51E-07	MTSS1, HRAS, TLN1, PDLIM7, S100A9, CAPZA1, RHOQ, RHOU, ACTG1, CDC42, DYNLL1, GSN, RHOA, RALA, CAP1, RHOF, FMNL1, ARHGEF2, ACTN1, TMSB10, MYH9, WAS, CDK5, VASP, FLNA, ARHGAP26, ELMO1, ARPC1A, EPB41L3, NCK2, ATP2C1, CFL1, SDCBP, BIN3, TMSB4X, FHOD1, LCP1, ALDOA, CXCL1, MYL6, DIAPH1, SSH2, FHL3, NEDD9, MYO9B, ARPC4, ARF6, CYTH2, ARPC5, CALR, TPM1, CAPZB, PFN1, DOCK2, EZR, RAC2, INPP5K, PTK2B, RAC1, CNN2,	1.37E-05

		PAFAH1B1, BCL6, SH2B2, TRIP10, ARHGDIB, PLEK, EVL, GAS7, CORO1A, ARAP1	
GO:0010627~regulation of protein kinase cascade	4.41E-07	SLC44A2, VAPA, ATP6AP2, ZEB2, PDCD4, PTEN, PRDX1, TGFB1, CTNNB1, MYD88, TMEM9B, ILK, TICAM1, CASP8, RHOA, IL1B, RHOC, PAK1, CASP1, RHOH, DBNL, PPP2R1A, BST2, LYN, C9ORF89, RELA, PKN1, PIM2, TMEM189, FLNA, UBE2N, OSM, ADRB2, TNFRSF10B, ATP2C1, F3, RIPK2, EEF1D, TNFAIP3, MAP3K11, CSF2, LITAF, PPM1A, UBE2V1, MAP4K1, FKBP1A, TNFRSF1A, SLC35B2, SQSTM1, PTK2B, PPP2CA, SHISA5, NENF, FCER1A, PTPRC, SECTM1, CFLAR, CARD8, IL6, HCLS1, LGALS1, TAOK3, DUSP22, BIRC2, LGALS9, GPS2, TRAF3IP2, APOL3, TNFSF10, HDAC3, HGS, IKBKB	2.37E-05
GO:0006417~regulation of translation	5.06E-07	SRP14, NCBP1, EIF5, MKNK2, PML, EIF5A, EIF2A, CALR, LUZP6, ZFP36L1, EIF4EBP1, EIF3B, PTK2B, EIF3H, WIBG, EIF3E, EIF3K, BCL3, QKI, EIF1, THBS1, DNAJC1, EIF2B5, IL6, PAIP2, MAGOH, NDUFA13, EIF1B, ANKHD1, RPS4X, ETF1, RPS5, SELT, SARNP, EIF4B, EIF4A3, NCK2, EIF4G2, EIF2AK1, EIF4E, EIF4A2, EIF4H, NGDN, PPP1R15B, PPP1R15A, EIF4E2	2.69E-05
GO:0065003~macromolecular complex assembly	5.97E-07	EIF6, NCBP1, OXA1L, HRAS, XPO6, TSPAN4,	3.14E-05

		SNRPD3, CAPZA1, RBM5, SNRPD1, SNRPD2, H1FX, TGFB1, SLC7A7, CTNNB1, PICALM, H2AFV, HIST1H2BK, ILK, H2AFZ, H2AFY, CLNS1A, H1F0, PCBD1, TAZ, H2AFJ, SPAG9, ADRM1, VAMP8, RPS14, RPS15, SNRPB, SLU7, TUBA4A, ATPIF1, VAMP3, CELF1, SNRPC, SNRPF, SNRPE, SURF1, SEPT9, SNRPG, TRAF1, HSD17B10, HMGB2, GRB2, DIAPH1, ARPC4, ABCA1, CALR, PDSS1, CD74, SRC, RAC1, NPM1, CDA, TAF9, SKIL, TAF7, SMAD3, GAS7, P2RX4, VCP, H3F3B, H3F3C, AP2S1, NAP1L1, MKI67IP, EIF2A, NDUFAF3, NDUFS5, NDUFS4, MAZ, GSN, GPX3, CASP8, GTF2A2, TUBB6, NRG1, TUBA1A, TUBA1B, TUBA1C, PILRB, IRAK1, PPP2R1A, ATPAF2, STX4, HSP90AA1, PFKL, HIST1H1C, CCNH, CCDC88C, SF1, DECR1, MBNL1, MBD2, NLRP3, SLC9A3R1, GTF2B, CDK5, HMGA1, WAS, FLNA, NCK2, TAF10, MED4, ADRB2, PIH1D1, DGAT1, TBCA, SBF2, UQCRH, IPO5, MDM2, TOMM22, PRNP, MAP3K11, HIST1H2AC, POLR2G, POLR2F, POLR2E, POLR2L, POLR2I, PML, FKBP1A, GCH1, TNFRSF1A, LPXN, SET, PTK2B, ALOX5AP, USP39, HSPA4, SCO2, TCP1, HIST1H2BD, YWHAB, RPL24, ANXA5, BIRC3, TRIM22, BIRC2, TRIM21, SOD2, CYBA, PSMG2,	

		PRLR, BAX, IRF7, TCEB2, TRPC4AP, TXNL4A	
GO:0034622~cellular macromolecular complex assembly	6.02E-07	EIF6, NCBP1, OXA1L, XPO6, SNRPD3, AP2S1, RBM5, NAP1L1, SNRPD1, SNRPD2, EIF2A, H1FX, TGFB1, NDUFAF3, NDUFS5, NDUFS4, PICALM, H2AFV, HIST1H2BK, GSN, GTF2A2, H2AFZ, TUBB6, H2AFY, NRG1, TUBA1A, TUBA1B, TUBA1C, PILRB, H1F0, CLNS1A, ATPAF2, HSP90AA1, HIST1H1C, TAZ, SF1, H2AFJ, MBNL1, MBD2, WAS, CDK5, FLNA, ADRM1, NCK2, ADRB2, PIH1D1, TBCA, RPS14, IPO5, RPS15, SNRPB, TUBA4A, SLU7, TOMM22, CELF1, SNRPC, SNRPF, SNRPE, SURF1, SNRPG, HIST1H2AC, HMGB2, DIAPH1, FKBP1A, ARPC4, CALR, SRC, SET, PTK2B, USP39, NPM1, RAC1, HSPA4, SCO2, TCP1, HIST1H2BD, SMAD3, RPL24, GAS7, CYBA, PSMG2, PRLR, H3F3B, H3F3C, TXNL4A	3.13E-05
GO:0031401~positive regulation of protein modification process	6.95E-07	PRKAG2, TGFB1, ATG7, PSMD1, PSMD2, IL1B, PSMD4, PSMD6, PSMD7, PSMD8, RPS27A, PSMD9, LYN, ANAPC5, TNFRSF14, SKP1, UBE2N, PSMA2, OSM, PSMA1, PSMA6, PSME1, CCND3, PSEN1, PSMA5, PSME2, PSMA4, PSMA3, UBC, UBB, UBA52, PSMB10, CSF2, PML, FKBP1A, ANAPC10, ANAPC11, PSMA7, STUB1, PSMB4, PSMF1, PSMB7, PSMB6, PSMB1, PSMB2, CD4, UBE2D1, FCER1A, IL6,	3.57E-05

		HCLS1, PSMC5, PSMD13, PSMC4, PSMC3, PSMD11, PSMC2, UBE2E1	
GO:0042110~T cell activation	7.72E-07	STAT5A, TNFSF14, PRDX2, FKBP1A, IL15, CD74, TGFB1, ADA, ITGAM, HSH2D, CTNNB1, CD48, SLC11A1, DOCK2, IL23A, CXCR4, BCL11A, BCL3, CD4, RHOH, ICAM1, PTPRC, CD3D, IKZF1, RELB, SMAD3, MINK1, MYH9, VAV1, WAS, NCK2, CD86, PRLR, CCND3, PSEN1, FYN, RPL22, JMJD6, BAX, IRF1, RIPK2, CLEC7A, LCP1	3.92E-05
GO:0006952~defense response	7.90E-07	S100A8, SNCA, S100A9, TLR2, IL15, TGFB1, IL10, CXCL10, CFP, CD48, MYD88, LILRA2, CD44, HIST1H2BK, IL1B, CSF3R, MX1, MX2, AKIRIN2, IL1A, PLD1, C5AR1, NCF2, NCF1, GNLY, RELA, CST3, HLA-C, TNFAIP6, LILRB2, CCR7, PPBP, CCR5, TIAL1, F3, LYST, LILRB3, DEFA3, MNDA, RIPK2, CLEC5A, C3AR1, CCL2, FGR, ADORA2A, CLU, ITGB2, CCL7, CD74, SLC11A1, IL23A, RAC1, DHX58, TYROBP, B4GALT1, BECN1, MAP2K3, IL1RN, MYO1F, CD300C, SLAMF7, S100A12, HDAC5, APOL3, CD55, CORO1A, CXCL16, CLEC7A, TREML1, HDAC9, KYNU, TOLLIP, LY86, PRDX5, PRDX2, NFKB1, PRDX1, CD97, FOS, TMEM173, HSF1, CXCR4, AOAH, TICAM1, ITIH4, LTF, MGLL, SERPINA1, TNIP1, FOSL1, IRAK2, NFKBIZ, SP100, LYN, LY96, CAMP, CNPY3, LYZ, ECSIT,	3.97E-05

		NLRP3, WAS, NLRP1, CD163, CD83, KDM6B, CXCL1, YWHAZ, NMI, CXCL3, CCR1, CXCL2, CALCOCO2, RSAD2, MLF2, GCH1, TNFRSF1A, TNFRSF1B, MEFV, CCL20, IL10RB, BCL3, THBS1, PTX3, TCIRG1, PTPRC, IL6, CEBPB, OLR1, IL8, HCK, RNASE6, ANXA1, SAMHD1, COTL1, STAT3, LSP1, KCNN4, CYBA, CYBB, IRF7, BNIP3L, LTA4H, CD14	
GO:0009144~purine nucleoside triphosphate metabolic process	8.16E-07	ATP5D, ALDOA, ATP5E, ATP6V0E1, ATP1B3, ATP5B, ATP6AP1, PRKAG2, RHOQ, ATP5G2, MYO9B, ATP5G1, ATP6V1B2, ATP5G3, ADA, TGFB1, ATP6V0B, GCH1, ATP2B1, ATP6V0C, ATP5L, RAB11A, ATP5O, ATP5I, ATP5H, ATP5J, TCIRG1, ATP5J2, NUDT1, ATP5F1, ATP6V1H, NADK, ATP1A1, DGUOK, ATP6V1F, NME1-NME2, ATP2A3, ATP2C1, ATP6V1E1, ADK, ATP5C1, ATP5A1	4.06E-05
GO:0008283~cell proliferation	9.56E-07	XRCC5, MORF4L1, TSPO, TNFSF14, IL15, CLK1, IL10, TGFB1, CTNNB1, ISG20, KDM1A, ILK, INSIG1, TGFBI, IL15RA, GNG2, IL1A, PDXK, BST2, PIM1, NRD1, VTI1B, PIM2, DCTN2, GLUL, VEGFA, RIPK2, BIN1, EMP1, CDV3, GNAI2, SIPA1, HDGF, FTH1, SRC, CD74, ITGAM, TAL1, SLC11A1, DOCK2, RPS27, IL23A, CKLF, NUMB, RAC1, PEMT, RUNX3, TFDP1, CSF1R, CRIP1, KLF10, SRA1, DUSP22, RAP1B, NBN, LY86, PRDX2, ZEB2,	4.70E-05

		FES, PTEN, PRDX1, DKC1, CXCR4, CREG1, NRG1, IMPDH1, IMPDH2, ARHGEF1, IFI16, MXD1, RBBP7, PRKCD, CDK5, LRPAP1, OSM, IFNAR2, CD86, EREG, PSEN1, TNFSF13B, CCND3, CDK11A, GNB1, TXN, MAPRE1, MAP3K11, CXCL1, USP8, FKBP1A, TCF7L2, PRPF19, SRRT, ANXA7, STAT4, PTK2B, RASGRP4, PAFAH1B1, ERCC1, PTPRC, RAF1, BAD, TNFSF8, LRP1, FYN, BAX, CKS2, ZNF259, IRF2	
GO:0051098~regulation of binding	9.79E-07	TLR2, PRDX2, TGFB1, IL10, RPS3, KDM1A, MYD88, AES, SMARCD3, GTF2A2, TICAM1, IL1B, NRG1, IRAK2, IRAK1, ICAM1, GTPBP4, SP100, PCBD1, RELA, PIM1, TMEM189, NLRP3, PRKCD, DDIT3, FLNA, LRPAP1, UBE2N, ADRB2, PSEN1, RIPK2, EDF1, SMARCA4, SIVA1, UBE2V1, NFKBIA, FKBP1A, TRIB1, NPM1, PYCARD, BCL3, APEX1, IL6, SMAD3, HDAC5, NME1-NME2, ID2, BAX, CALM3, IKBKB, CALM2, CALM1	4.76E-05
GO:0052548~regulation of endopeptidase activity	1.03E-06	SIVA1, ADORA2A, MGMT, SNCA, PML, PMAIP1, RPS3, SLC11A2, CDKN2D, MTCH1, PYCARD, DIABLO, HSPE1, HSPA5, CARD8, HERPUD1, CYCS, SMAD3, NRD1, NR4A1, BAD, STAT1, BCL2L13, NLRP3, NLRP1, TNFRSF10B, VCP, F3, BAX, IFT57, DNAJB6, IFI6	4.96E-05
GO:0006413~translational initiation	1.04E-06	EIF5, EIF2S3, EIF1B, RPS5, GTF2B, RPS3, EIF3D,	4.94E-05

		EIF4E, EIF3B, EIF3G, RPS3A, EIF3H, EIF3E, EIF3F, EIF1AY, EIF3K, EIF3L, EIF1, EIF3I, EIF3M, EIF4E2, EIF2B5	
GO:0046649~lymphocyte activation	1.18E-06	NBN, STAT5A, TNFSF14, PRDX2, IL15, TGFB1, IL10, CTNNB1, CD48, CXCR4, IMPDH1, IMPDH2, RHOH, ICAM1, BST2, CD3D, RELB, MINK1, MYH9, WAS, PRKCD, NCK2, CD86, LAT2, CCND3, PSEN1, IGBP1, RIPK2, LCP1, GPR183, FKBP1A, CD74, ADA, ITGAM, HSH2D, SLC11A1, DOCK2, IL23A, BCL11A, BCL3, CD4, BCL6, ERCC1, KLF6, PTPRC, IKZF1, SMAD3, SLAMF7, VAV1, FOXP1, HDAC5, PRLR, FYN, RPL22, JMJD6, BAX, IRF1, CLEC7A, HDAC9	5.59E-05
GO:0043281~regulation of caspase activity	1.37E-06	SIVA1, ADORA2A, MGMT, SNCA, PML, PMAIP1, RPS3, SLC11A2, CDKN2D, MTCH1, PYCARD, DIABLO, HSPE1, HSPA5, CARD8, HERPUD1, CYCS, SMAD3, NR4A1, BAD, STAT1, BCL2L13, NLRP3, NLRP1, TNFRSF10B, VCP, F3, BAX, IFT57, DNAJB6, IFI6	6.40E-05
GO:0009259~ribonucleotide metabolic process	1.77E-06	ATP5D, ALDOA, ATP5E, ATP6V0E1, ATP1B3, ATP5B, ATP6AP1, PRKAG2, RHOQ, MYO9B, ATP5G2, ATP5G1, ATP6V1B2, ATP5G3, TGFB1, ADA, ATP6V0B, GCH1, ATP2B1, ATP6V0C, ATP5L, RAB11A, ATP5O, ATP5I, ATP5H, IMPDH1, IMPDH2, ATP5J, TCIRG1, ATP5J2, NUDT5, ATP5F1, CECR1, ATP6V1H, NADK, ATP1A1, AMPD2, AMPD3,	8.17E-05

		ATP6V1F, NME1-NME2, ATP2A3, ATP2C1, ADK, ATP6V1E1, ATP5C1, DPYD, ATP5A1	
GO:0015992~proton transport	2.02E-06	ATP5D, ATP5E, ATP6V0E1, ATP5B, ATP6AP1, ATP5G2, ATP6V1B2, ATP6V1G1, ATP5G1, ATP5G3, ATP6V0B, ATP6V0C, ATP5L, ATP5O, ATP5I, ATP5H, ATP5J, TCIRG1, ATP5J2, ATP5F1, ATP6V1H, ATP6V1F, UCP2, ATP6V1E1, ATP5C1, ATP5A1	9.23E-05
GO:0009615~response to virus	2.11E-06	FGR, ZC3HAV1, RPS15A, RSAD2, IVNS1ABP, BANF1, IFI35, ISG20, IL23A, ISG15, CXCR4, TICAM1, BCL3, MX1, IFNGR2, FOSL1, MX2, IFNGR1, PTPRC, IL6, BST2, BECN1, RELA, SAMHD1, IFI44, IFI16, STAT1, NLRP3, TRIM22, LCN2, IRF9, LILRB1, PLSCR1, IFNAR2, IRF7, LYST, BNIP3L, DEFA3	9.54E-05
GO:0042254~ribosome biogenesis	2.25E-06	EIF6, NAF1, RPL14, NHP2L1, UTP6, EIF2A, EXOSC10, RPS28, DKC1, RPL7, FRG1, RPLP0, NPM1, RPL5, RPL11, RPL7A, RSL24D1, GNL2, FTSJ1, IMP4, NSA2, RPS24, RPL35A, GTPBP4, EXOSC8, RPL26, RPL24, EXOSC1, RPS6, NOP10, FBL, RPS7, EIF4A3, DDX56, RPS19, RPS16, RPS14, RPS15, NOP58, NOP56, POP4	1.01E-04
GO:0006793~phosphorus metabolic process	2.26E-06	STAT5A, SNCA, CLK1, CLK3, ILK, MAP3K8, TLK2, DBNL, C5AR1, PIM1, PIM3, PIM2, SPAG9, PPP1CA, EIF2AK1, MAPK6, ATP5C1, CAMK1, GNAI2, HUS1, STK17B,	1.00E-04

		DUSP10, ADRBK1, SDHAF2, CD74, ATP6V0B, ATP6V0C, FCER1A, PTPN18, MAP2K2, MAP2K3, TAOK3, DUSP22, PTPN12, PPA1, PTPN11, GRK6, GK, ATP5D, ATP5E, ATP6V0E1, TOLLIP, ATP5B, EIF2A, PTEN, HSF1, ATP5L, SYNJ2, ATP5O, ATP5I, ATP5H, ATP5J, NT5C, NDUFB10, LYN, INPPL1, PHKG2, DGUOK, PI4KB, NDUFA10, MTMR11, MTMR14, PSEN1, CDK11A, GADD45B, MAP3K11, PPP6C, MKNK2, HK2, PML, FPR1, ABI1, MTMR3, STAT4, STK40, PTK2B, PPP2CA, THBS1, CSNK1A1, TRIM28, NADK, STAT1, SOD1, DUSP5, DUSP3, DUSP2, DUSP1, RPS6KA1, CSNK1D, CSNK1E, FYN, GSK3A, ATP6V1E1, GSK3B, PTP4A1, PTP4A2, ARAF, ATP5A1, PGK1, DUSP6, NRBP1, ATP6AP1, NDUFAB1, RPS6KB2, CASK, TGFB1, UQCR11, AAK1, IL1B, PRKACA, MADD, STK24, CSNK1G2, TAZ, PPP1CB, MAP4K3, MAP4K4, RIOK3, RIPK2, STYXL1, CCL2, MVD, FGR, STK10, SSH2, MAPKAPK3, MAP4K1, MAPKAPK2, ATP6V1B2, SRC, CSF1R, PTPN6, PTPN2, PLK3, NDUFV1, PTPN1, IKBKB, PHPT1, MYLK, UQCRC2, IMPA2, UQCRC1, PRDX2, FES, NDUFS6, NDUFS5, NDUFS4, CXCR4, ACYP2, PAK1, NDUFS3, NDUFS2, AKT3, IRAK2, IRAK1,	

		PPP2R1A, SGK1, NDUFC2, MINK1, ATP6V1H, PKN1, NDUFC1, PRKCE, PRKCD, CDK5, GAK, ATP6V1F, TYK2, OSM, PPM1F, PPM1G, PRKD2, SBF1, UQCRH, SBF2, CFL1, UQCRB, NDUFB3, NDUFB4, NDUFB5, NDUFB7, NDUFB8, NDUFB9, PPM1A, ATP5G2, ATP5G1, ATP5G3, NDUFB1, NDUFB2, TRIB1, NDUFA4, TCIRG1, PTPRC, NDUFA5, NDUFA2, PTPRE, NDUFA3, NDUFA9, HCK, NDUFA6, NDUFA7, ATP5F1, RAF1, NDUFA1, NME1-NME2, PRLR, GMFG	
GO:0006796~phosphate metabolic process	2.26E-06	STAT5A, SNCA, CLK1, CLK3, ILK, MAP3K8, TLK2, DBNL, C5AR1, PIM1, PIM3, PIM2, SPAG9, PPP1CA, EIF2AK1, MAPK6, ATP5C1, CAMK1, GNAI2, HUS1, STK17B, DUSP10, ADRBK1, SDHAF2, CD74, ATP6V0B, ATP6V0C, FCER1A, PTPN18, MAP2K2, MAP2K3, TAOK3, DUSP22, PTPN12, PPA1, PTPN11, GRK6, GK, ATP5D, ATP5E, ATP6V0E1, TOLLIP, ATP5B, EIF2A, PTEN, HSF1, ATP5L, SYNJ2, ATP5O, ATP5I, ATP5H, ATP5J, NT5C, NDUFB10, LYN, INPPL1, PHKG2, DGUOK, PI4KB, NDUFA10, MTMR11, MTMR14, PSEN1, CDK11A, GADD45B, MAP3K11, PPP6C, MKNK2, HK2, PML, FPR1, ABI1, MTMR3, STAT4, STK40, PTK2B, PPP2CA,	1.00E-04

		THBS1, CSNK1A1, TRIM28, NADK, STAT1, SOD1, DUSP5, DUSP3, DUSP2, DUSP1, RPS6KA1, CSNK1D, CSNK1E, FYN, GSK3A, ATP6V1E1, GSK3B, PTP4A1, PTP4A2, ARAF, ATP5A1, PGK1, DUSP6, NRBP1, ATP6AP1, NDUFAB1, RPS6KB2, CASK, TGFB1, UQCR11, AAK1, IL1B, PRKACA, MADD, STK24, CSNK1G2, TAZ, PPP1CB, MAP4K3, MAP4K4, RIOK3, RIPK2, STYXL1, CCL2, MVD, FGR, STK10, SSH2, MAPKAPK3, MAP4K1, MAPKAPK2, ATP6V1B2, SRC, CSF1R, PTPN6, PTPN2, PLK3, NDUFV1, PTPN1, IKBKB, PHPT1, MYLK, UQCRC2, IMPA2, UQCRC1, PRDX2, FES, NDUFS6, NDUFS5, NDUFS4, CXCR4, ACYP2, PAK1, NDUFS3, NDUFS2, AKT3, IRAK2, IRAK1, PPP2R1A, SGK1, NDUFC2, MINK1, ATP6V1H, PKN1, NDUFC1, PRKCE, PRKCD, CDK5, GAK, ATP6V1F, TYK2, OSM, PPM1F, PPM1G, PRKD2, SBF1, UQCRH, SBF2, CFL1, UQCRB, NDUFB3, NDUFB4, NDUFB5, NDUFB7, NDUFB8, NDUFB9, PPM1A, ATP5G2, ATP5G1, ATP5G3, NDUFB1, NDUFB2, TRIB1, NDUFA4, TCIRG1, PTPRC, NDUFA5, NDUFA2, PTPRE, NDUFA3, NDUFA9, HCK, NDUFA6, NDUFA7, ATP5F1, RAF1, NDUFA1, NME1-NME2, PRLR, GMFG	
GO:0009141~nucleoside	2.45E-06	ATP5D, ALDOA, ATP5E,	1.08E-04

triphosphate metabolic process		ATP6V0E1, ATP1B3, ATP5B, ATP6AP1, PRKAG2, RHOQ, ATP5G2, MYO9B, ATP5G1, ATP6V1B2, ATP5G3, ADA, TGFB1, ATP6V0B, GCH1, ATP2B1, ATP6V0C, ATP5L, RAB11A, ATP5O, ATP5I, ATP5H, ATP5J, TCIRG1, ATP5J2, NUDT1, ATP5F1, ATP6V1H, NADK, ATP1A1, DGUOK, ATP6V1F, NME1-NME2, ATP2A3, ATP2C1, ATP6V1E1, ADK, ATP5C1, ATP5A1, DUT	
GO:0009260~ribonucleotide biosynthetic process	3.55E-06	ATP5D, ALDOA, ATP5E, ATP6V0E1, ATP1B3, ATP5B, ATP6AP1, PRKAG2, ATP5G2, ATP5G1, ATP6V1B2, ATP5G3, TGFB1, ADA, ATP6V0B, ATP2B1, ATP6V0C, ATP5L, ATP5O, ATP5I, ATP5H, IMPDH1, IMPDH2, ATP5J, TCIRG1, ATP5J2, ATP5F1, CECR1, ATP6V1H, ATP1A1, AMPD2, AMPD3, ATP6V1F, NME1-NME2, ATP2A3, ATP2C1, ATP6V1E1, ADK, ATP5C1, DPYD, ATP5A1	1.55E-04
GO:0006818~hydrogen transport	4.06E-06	ATP5D, ATP5E, ATP6V0E1, ATP5B, ATP6AP1, ATP5G2, ATP6V1B2, ATP6V1G1, ATP5G1, ATP5G3, ATP6V0B, ATP6V0C, ATP5L, ATP5O, ATP5I, ATP5H, ATP5J, TCIRG1, ATP5J2, ATP5F1, ATP6V1H, ATP6V1F, UCP2, ATP6V1E1, ATP5C1, ATP5A1	1.75E-04
GO:0009150~purine ribonucleotide metabolic process	4.30E-06	ATP5D, ALDOA, ATP5E, ATP6V0E1, ATP1B3, ATP5B, ATP6AP1, PRKAG2, RHOQ, MYO9B, ATP5G2, ATP5G1,	1.84E-04

		ATP6V1B2, ATP5G3, TGFB1, ADA, ATP6V0B, GCH1, ATP2B1, ATP6V0C, ATP5L, RAB11A, ATP5O, ATP5I, ATP5H, IMPDH1, IMPDH2, ATP5J, TCIRG1, ATP5J2, ATP5F1, CECR1, ATP6V1H, NADK, ATP1A1, AMPD2, AMPD3, ATP6V1F, NME1-NME2, ATP2A3, ATP2C1, ATP6V1E1, ATP5C1, ATP5A1	
GO:0009152~purine ribonucleotide biosynthetic process	5.15E-06	ATP5D, ALDOA, ATP5E, ATP6V0E1, ATP1B3, ATP5B, ATP6AP1, PRKAG2, ATP5G2, ATP5G1, ATP6V1B2, ATP5G3, TGFB1, ADA, ATP6V0B, ATP2B1, ATP6V0C, ATP5L, ATP5O, ATP5I, ATP5H, IMPDH1, IMPDH2, ATP5J, TCIRG1, ATP5J2, ATP5F1, CECR1, ATP6V1H, ATP1A1, AMPD2, AMPD3, ATP6V1F, NME1-NME2, ATP2A3, ATP2C1, ATP6V1E1, ATP5C1, ATP5A1	2.18E-04
GO:0007264~small GTPase mediated signal transduction	5.66E-06	HRAS, RAB5C, RASGEF1B, RAB1B, SHOC2, RHOQ, RGL4, RFXANK, RHOU, RAB1A, ARHGAP4, CDC42, RAB28, RHOA, RAB24, RALA, RHOC, RAPGEF2, RAB20, RHOF, SAR1A, RHOG, AGAP3, RHOH, GTPBP2, DBNL, RSU1, PLD1, ARHGEF1, RAP2C, RAB4B, ELMO1, CRKL, DOK3, GNB1, CFL1, RIN2, SDCBP, ARL8A, ARL8B, RAB13, RAB10, ARL4C, RAB7A, USP8, GRB2, MAPKAPK3, MYO9B, ARF6, MAPKAPK2, ARF5, ABCA1, SRC, RAC2, RASGRP4, RASGRP2,	2.37E-04

		RAC1, RAB11A, ARHGDIB, RAB2A, GDI1, GDI2, ABR, RAB8B, MAP2K2, YWHAB, RAF1, PARK7, RALGDS, RAB32, LAT, RAB31, ARF3, RAB34, ARF4, YWHAQ, RAP1A, RAP1B, RIT1	
GO:0043123~positive regulation of I-kappaB kinase/NF-kappaB cascade	6.98E-06	SLC44A2, VAPA, LITAF, PPM1A, UBE2V1, FKBP1A, TNFRSF1A, MYD88, TMEM9B, SLC35B2, CASP8, SHISA5, TICAM1, RHOA, IL1B, RHOC, CASP1, SECTM1, CFLAR, BST2, RELA, LGALS1, PIM2, TMEM189, BIRC2, LGALS9, FLNA, UBE2N, TRAF3IP2, APOL3, TNFSF10, TNFRSF10B, ATP2C1, RIPK2, EEF1D	2.90E-04
GO:0034621~cellular macromolecular complex subunit organization	7.20E-06	EIF6, NCBP1, OXA1L, XPO6, SNRPD3, AP2S1, RBM5, NAP1L1, SNRPD1, SNRPD2, EIF2A, H1FX, TGFB1, NDUFAF3, NDUFS5, NDUFS4, MAZ, PICALM, H2AFV, HIST1H2BK, GSN, GTF2A2, H2AFZ, TUBB6, H2AFY, NRG1, TUBA1A, TUBA1B, TUBA1C, PILRB, H1F0, CLNS1A, ATPAF2, HSP90AA1, HIST1H1C, TAZ, SF1, H2AFJ, MBNL1, MBD2, HMGA1, WAS, CDK5, FLNA, ADRM1, NCK2, ADRB2, PIH1D1, TBCA, RPS14, IPO5, RPS15, SNRPB, TUBA4A, SLU7, TOMM22, CELF1, SNRPC, SNRPF, SNRPE, SURF1, SNRPG, HIST1H2AC, HMGB2, DIAPH1, FKBP1A, ARPC4, CALR, SRC, SET, PTK2B, USP39, NPM1, RAC1, HSPA4, SCO2, TCP1, HIST1H2BD,	2.96E-04

		SMAD3, RPL24, EVL, ETF1, GAS7, CYBA, PSMG2, PRLR, H3F3B, H3F3C, TXNL4A	
GO:0006457~protein folding	7.33E-06	HSP90AB1, GRPEL1, PDIA6, CCT2, DNAJB12, CCT3, CANX, AIP, DNAJB11, DNAJC7, SEP15, DNAJC4, DNAJC1, CLN3, HSP90AA1, ERP29, CCT6A, LRPAP1, PFDN2, UXT, PFDN1, TBCA, PFDN5, TBCD, PFDN4, AHSP, SIL1, FKBP2, FKBP8, FKBP1A, CALR, STUB1, CD74, SH3GLB1, MPDU1, DNAJA1, HSPE1, QSOX1, HSPA8, HSPA9, TCP1, CCT7, PPIF, CCT5, HSP90B1, CCT4, PPIB, PPIA, CCT8, DNAJB2, AHSA1, DNAJB6	2.99E-04
GO:0006754~ATP biosynthetic process	7.60E-06	ATP5D, ALDOA, ATP5E, ATP6V0E1, ATP1B3, ATP5B, ATP6AP1, PRKAG2, ATP5G2, ATP6V1B2, ATP5G1, ATP5G3, TGFB1, ATP6V0B, ATP2B1, ATP6V0C, ATP5L, ATP5O, ATP5I, ATP5H, ATP5J, TCIRG1, ATP5J2, ATP5F1, ATP6V1H, ATP1A1, ATP6V1F, ATP2C1, ATP2A3, ATP6V1E1, ATP5C1, ATP5A1	3.07E-04
GO:0002237~response to molecule of bacterial origin	1.02E-05	CCL2, PTGS2, SNCA, TLR2, NFKBIA, PRDX2, COMT, IL10, GCH1, B2M, TRIB1, FOS, SLC11A1, TNFRSF1A, MYD88, HSF1, TICAM1, IL1B, SERPINA1, IRG1, AKIRIN2, IRAK1, IL6, LY96, SOCS3, RELA, STAT1, ADM, RIPK2, CD14, MGST1	4.10E-04
GO:0009145~purine nucleoside triphosphate biosynthetic process	1.14E-05	ATP5D, ALDOA, ATP5E, ATP6V0E1, ATP1B3, ATP5B, ATP6AP1, PRKAG2, ATP5G2,	4.53E-04

		ATP6V1B2, ATP5G1, ATP5G3, TGFB1, ATP6V0B, ATP2B1, ATP6V0C, ATP5L, ATP5O, ATP5I, ATP5H, ATP5J, TCIRG1, ATP5J2, ATP5F1, ATP6V1H, ATP1A1, ATP6V1F, NME1-NME2, ATP2C1, ATP2A3, ATP6V1E1, ADK, ATP5C1, ATP5A1	
GO:0043933~macromolecular complex subunit organization	1.19E-05	EIF6, NCBP1, OXA1L, HRAS, XPO6, TSPAN4, SNRPD3, CAPZA1, RBM5, SNRPD1, SNRPD2, H1FX, TGFB1, SLC7A7, CTNNB1, PICALM, H2AFV, HIST1H2BK, ILK, H2AFZ, H2AFY, CLNS1A, H1F0, PCBD1, TAZ, H2AFJ, SPAG9, ADRM1, VAMP8, RPS14, RPS15, SNRPB, SLU7, TUBA4A, ATPIF1, VAMP3, CELF1, SNRPC, SNRPF, SNRPE, SURF1, SEPT9, SNRPG, TRAF1, HSD17B10, HMGB2, GRB2, DIAPH1, ARPC4, ABCA1, CALR, PDSS1, CD74, SRC, RAC1, NPM1, CDA, TAF9, SKIL, TAF7, SMAD3, EVL, GAS7, P2RX4, VCP, H3F3B, H3F3C, AP2S1, NAP1L1, MKI67IP, EIF2A, NDUFAF3, NDUFS5, NDUFS4, MAZ, GSN, GPX3, CASP8, GTF2A2, TUBB6, TUBA1A, NRG1, TUBA1B, TUBA1C, PILRB, IRAK1, PPP2R1A, ATPAF2, STX4, HSP90AA1, PFKL, HIST1H1C, CCNH, CCDC88C, SF1, DECR1, MBNL1, MBD2, NLRP3, SLC9A3R1, GTF2B, CDK5, HMGA1, WAS, FLNA, NCK2, TAF10, MED4, ADRB2, PIH1D1, DGAT1,	4.70E-04

		TBCA, SBF2, UQCRH, IPO5, MDM2, TOMM22, PRNP, MAP3K11, HIST1H2AC, POLR2G, POLR2F, POLR2E, POLR2L, POLR2I, PML, FKBP1A, GCH1, TNFRSF1A, LPXN, SET, PTK2B, ALOX5AP, USP39, HSPA4, SCO2, TCP1, HIST1H2BD, YWHAB, RPL24, ANXA5, ETF1, BIRC3, TRIM22, BIRC2, TRIM21, SOD2, CYBA, PSMG2, PRLR, BAX, IRF7, TCEB2, TRPC4AP, TXNL4A	
GO:0009205~purine ribonucleoside triphosphate metabolic process	1.35E-05	ATP5D, ALDOA, ATP5E, ATP6V0E1, ATP1B3, ATP5B, ATP6AP1, PRKAG2, RHOQ, ATP5G2, MYO9B, ATP6V1B2, ATP5G1, ATP5G3, TGFB1, ATP6V0B, GCH1, ATP2B1, ATP6V0C, ATP5L, RAB11A, ATP5O, ATP5I, ATP5H, ATP5J, TCIRG1, ATP5J2, ATP5F1, ATP6V1H, NADK, ATP1A1, ATP6V1F, NME1-NME2, ATP2A3, ATP2C1, ATP6V1E1, ATP5C1, ATP5A1	5.27E-04
GO:0070661~leukocyte proliferation	1.38E-05	PTPRC, TNFSF14, FKBP1A, PRDX2, IL15, PRKCD, ITGAM, IL10, TGFB1, SLC11A1, DOCK2, CD86, IL23A, CCND3, CXCR4, FYN, BAX, RIPK2, IMPDH1, IMPDH2	5.33E-04
GO:0032943~mononuclear cell proliferation	1.38E-05	PTPRC, TNFSF14, FKBP1A, PRDX2, IL15, PRKCD, ITGAM, IL10, TGFB1, SLC11A1, DOCK2, CD86, IL23A, CCND3, CXCR4, FYN, BAX, RIPK2, IMPDH1, IMPDH2	5.33E-04
GO:0034097~response to cytokine stimulus	1.42E-05	KYNU, MCL1, PTGS2, SNCA, PML, CALCOCO2, SRC, GCH1, CTNNB1,	5.46E-04

		STAT6, FOS, SLC11A1, AES, MYD88, CASP8, TAF9, SERPINA1, FOSL1, IRAK2, IRAK1, SP100, SOCS3, RELA, STAT1, JUNB, STAT3, CORO1A, CXCL16, RIPK2	
GO:0042098~T cell proliferation	1.47E-05	PTPRC, TNFSF14, PRDX2, FKBP1A, IL15, ITGAM, SLC11A1, DOCK2, CD86, IL23A, CCND3, CXCR4, FYN, BAX, RIPK2	5.58E-04
GO:0016072~rRNA metabolic process	1.54E-05	NAF1, RPL14, UTP6, MKI67IP, EXOSC10, RPS28, DKC1, RPL7, FRG1, RPL5, RPL11, FTSJ1, IMP4, DEDD2, NSA2, RPS24, RPL35A, EXOSC8, RPL26, EXOSC1, RPS6, NOP10, FBL, RPS7, EIF4A3, DDX56, RPS19, RPS16, RPS14, RPS15, NOP58, NOP56, POP4	5.81E-04
GO:0006605~protein targeting	1.64E-05	GNPTG, SRP14, GRPEL1, TSPO, XPO6, PDIA3, TIMM17A, ATP6AP1, TIMM17B, EIF5A, SRP19, TGFB1, AIP, SRPR, HOMER3, TIMM9, RPL11, RAN, NDUFA13, OPTN, ATG4D, SEC61B, IPO5, TOMM20, SDCBP, TOMM22, KPNA2, SEC61G, NXT1, BID, YWHAZ, PML, TIMM10, NFKBIA, CALR, SEC62, CDC37, SLC11A1, NUP214, TOMM7, TOMM5, PEX16, EXOC4, BCL3, BCL6, TRAM1, SEC61A1, HSPA9, RBM22, YWHAB, YWHAE, GABARAP, RPL23, ERBB2IP, GSK3B, YWHAQ, NOP58, SSR2, SSR3	6.14E-04
GO:0009199~ribonucleoside triphosphate metabolic process	1.67E-05	ATP5D, ALDOA, ATP5E, ATP6V0E1, ATP1B3, ATP5B, ATP6AP1, PRKAG2, RHOQ, ATP5G2,	6.21E-04

		MYO9B, ATP6V1B2, ATP5G1, ATP5G3, TGFB1, ATP6V0B, GCH1, ATP2B1, ATP6V0C, ATP5L, RAB11A, ATP5O, ATP5I, ATP5H, ATP5J, TCIRG1, ATP5J2, ATP5F1, ATP6V1H, NADK, ATP1A1, ATP6V1F, NME1-NME2, ATP2A3, ATP2C1, ATP6V1E1, ATP5C1, ATP5A1	
GO:0006954~inflammatory response	1.95E-05	S100A8, TOLLIP, LY86, S100A9, TLR2, PRDX5, NFKB1, PRDX2, IL15, IL10, TGFB1, CXCL10, CD97, CFP, FOS, MYD88, CD44, CXCR4, AOAH, TICAM1, ITIH4, MGLL, IL1B, SERPINA1, IL1A, IRAK2, NFKBIZ, LYN, LY96, RELA, LYZ, NLRP3, CD163, TNFAIP6, CCR7, CCR5, F3, RIPK2, KDM6B, CXCL1, C3AR1, YWHAZ, CCL2, NMI, ADORA2A, CXCL3, CCR1, CXCL2, CLU, ITGB2, CCL7, TNFRSF1A, SLC11A1, TNFRSF1B, IL23A, MEFV, CCL20, IL10RB, RAC1, THBS1, PTX3, B4GALT1, IL6, CEBPB, OLR1, IL8, MAP2K3, IL1RN, ANXA1, STAT3, S100A12, HDAC5, CYBA, APOL3, CD55, CYBB, IRF7, LTA4H, CLEC7A, HDAC9, CD14	7.19E-04
GO:0009142~nucleoside triphosphate biosynthetic process	2.30E-05	ATP5D, ALDOA, ATP5E, ATP6V0E1, ATP1B3, ATP5B, ATP6AP1, PRKAG2, ATP5G2, ATP6V1B2, ATP5G1, ATP5G3, TGFB1, ATP6V0B, ATP2B1, ATP6V0C, ATP5L, ATP5O, ATP5I, ATP5H, ATP5J, TCIRG1, ATP5J2, ATP5F1, ATP6V1H, ATP1A1, ATP6V1F, NME1-NME2,	8.40E-04

		ATP2C1, ATP2A3, ATP6V1E1, ADK, ATP5C1, ATP5A1	
GO:0009206~purine ribonucleoside triphosphate biosynthetic process	2.47E-05	ATP5D, ALDOA, ATP5E, ATP6V0E1, ATP1B3, ATP5B, ATP6AP1, PRKAG2, ATP5G2, ATP6V1B2, ATP5G1, ATP5G3, TGFB1, ATP6V0B, ATP2B1, ATP6V0C, ATP5L, ATP5O, ATP5I, ATP5H, ATP5J, TCIRG1, ATP5J2, ATP5F1, ATP6V1H, ATP1A1, ATP6V1F, NME1-NME2, ATP2C1, ATP2A3, ATP6V1E1, ATP5C1, ATP5A1	8.95E-04
GO:0032496~response to lipopolysaccharide	2.52E-05	CCL2, PTGS2, SNCA, NFKBIA, PRDX2, COMT, IL10, GCH1, TRIB1, FOS, SLC11A1, TNFRSF1A, MYD88, HSF1, TICAM1, IL1B, SERPINA1, IRG1, AKIRIN2, IRAK1, LY96, SOCS3, RELA, STAT1, ADM, RIPK2, CD14, MGST1	9.03E-04
GO:0046651~lymphocyte proliferation	2.62E-05	PTPRC, TNFSF14, FKBP1A, PRDX2, IL15, PRKCD, ITGAM, IL10, SLC11A1, DOCK2, CD86, IL23A, CCND3, CXCR4, FYN, BAX, RIPK2, IMPDH1, IMPDH2	9.34E-04
GO:0010033~response to organic substance	2.75E-05	PTGS2, AQP9, RBM3, STAT5A, SNCA, RBM4, TLR2, PMAIP1, TGFB1, IL10, B2M, CTNNB1, CD48, EIF4EBP1, MYD88, CD44, IL1B, GNG2, RARA, PRKACA, DNAJC4, SUPT5H, GNG5, AKIRIN2, EIF2B5, PLD1, SOCS3, RELA, BAIAP2, FBP1, DDIT3, JUNB, RETN, GLUL, RIPK2, NFE2L2, DERL2, HMGB2, CCL2, DERL1, MCL1, ADORA2A, TXN2, GRB2, NFKBIA,	9.73E-04

		ADRBK1, NEDD8, ASL, MANF, SRC, SLC11A1, PEMT, DNAJA1, HSPE1, TAF9, SDF4, HERPUD1, PTPN2, KLF10, IL1RN, SMAD3, PTPN11, HDAC5, P2RX4, CDKN1A, CORO1A, VCP, CXCL16, PRKAR1A, ALDH2, DNAJB2, PTPN1, CLEC7A, HDAC9, DNAJB6, HDAC6, SLC9A1, HSP90AB1, KYNU, ATP6V0E1, UQCRC1, LDLR, RHOQ, PRDX2, UQCRFS1, PTEN, ASAH1, ACTR3, FOS, NDUFS4, AES, HSF1, GSN, GPX4, CASP8, TICAM1, SERPINA1, CASP1, FOSL1, IRAK2, IRAK1, PPP2R1A, HSP90AA1, SP100, LYN, PFKL, LY96, ADIPOR1, CDK5, UBE2B, CD83, BTG2, ADM, GNB2, GNB1, CFL1, CTSC, GNAS, PPP1R15A, PML, CALCOCO2, FKBP1A, COMT, NR3C1, ATP5G3, GCH1, TRIB1, STAT6, TNFRSF1A, SRRT, ACSL1, PLIN2, PTK2B, PPP2CA, SH2B2, CD4, HSPA4, THBS1, IRG1, HSPA8, IL6, SELL, HCLS1, ANXA5, STAT1, SOD1, BIRC2, STAT3, ID2, NME1-NME2, DUSP1, GSK3A, FYN, MGST1, CD14	
GO:0009201~ribonucleoside triphosphate biosynthetic process	3.11E-05	ATP5D, ALDOA, ATP5E, ATP6V0E1, ATP1B3, ATP5B, ATP6AP1, PRKAG2, ATP5G2, ATP6V1B2, ATP5G1, ATP5G3, TGFB1, ATP6V0B, ATP2B1, ATP6V0C, ATP5L, ATP5O, ATP5I, ATP5H, ATP5J, TCIRG1, ATP5J2, ATP5F1, ATP6V1H, ATP1A1, ATP6V1F, NME1-NME2,	1.09E-03

		ATP2C1, ATP2A3, ATP6V1E1, ATP5C1, ATP5A1	
GO:0010740~positive regulation of protein kinase cascade	3.20E-05	SLC44A2, VAPA, TGFB1, CTNNB1, TMEM9B, MYD88, TICAM1, CASP8, ILK, RHOA, IL1B, RHOC, CASP1, LYN, BST2, RELA, PIM2, TMEM189, FLNA, OSM, UBE2N, ADRB2, TNFRSF10B, ATP2C1, F3, RIPK2, EEF1D, CSF2, LITAF, PPM1A, UBE2V1, FKBP1A, TNFRSF1A, SLC35B2, SHISA5, NENF, CFLAR, PTPRC, SECTM1, IL6, HCLS1, LGALS1, TAOK3, DUSP22, BIRC2, LGALS9, TRAF3IP2, APOL3, TNFSF10	1.11E-03
GO:0016310~phosphorylation	3.27E-05	NRBP1, ATP6AP1, STAT5A, SNCA, NDUFAB1, RPS6KB2, CASK, CLK1, TGFB1, UQCR11, CLK3, AAK1, ILK, MAP3K8, IL1B, PRKACA, TLK2, DBNL, C5AR1, CSNK1G2, MADD, STK24, TAZ, PIM1, PIM3, PIM2, MAP4K3, SPAG9, MAP4K4, EIF2AK1, MAPK6, RIOK3, ATP5C1, RIPK2, CAMK1, CCL2, GNAI2, MVD, FGR, STK10, HUS1, STK17B, MAPKAPK3, MAP4K1, ADRBK1, ATP6V1B2, MAPKAPK2, SDHAF2, ATP6V0B, CD74, SRC, ATP6V0C, CSF1R, FCER1A, MAP2K2, MAP2K3, TAOK3, PTPN11, PLK3, NDUFV1, GRK6, IKBKB, MYLK, UQCRC2, ATP5D, ATP5E, ATP6V0E1, UQCRC1, TOLLIP, ATP5B, PRDX2, EIF2A, FES, NDUFS6, NDUFS5, NDUFS4, HSF1, CXCR4, ATP5L, ATP5O,	1.13E-03

		PAK1, ATP5I, NDUFS3, NDUFS2, ATP5H, AKT3, ATP5J, IRAK2, IRAK1, SGK1, NDUFB10, LYN, PHKG2, NDUFC2, MINK1, PKN1, ATP6V1H, NDUFC1, DGUOK, PI4KB, PRKCE, NDUFA10, CDK5, PRKCD, GAK, ATP6V1F, OSM, TYK2, PRKD2, PSEN1, UQCRH, CDK11A, CFL1, GADD45B, UQCRB, MAP3K11, NDUFB3, NDUFB4, NDUFB5, NDUFB7, NDUFB8, NDUFB9, MKNK2, HK2, FPR1, PML, ABI1, ATP5G2, ATP5G1, ATP5G3, NDUFB1, NDUFB2, TRIB1, STAT4, STK40, PTK2B, THBS1, TCIRG1, NDUFA4, CSNK1A1, PTPRC, NDUFA5, NDUFA2, PTPRE, NDUFA3, NDUFA9, NDUFA6, HCK, NDUFA7, TRIM28, ATP5F1, RAF1, NADK, STAT1, SOD1, NDUFA1, NME1-NME2, RPS6KA1, CSNK1D, PRLR, GSK3A, FYN, CSNK1E, GMFG, GSK3B, ATP6V1E1, ARAF, ATP5A1, PGK1	
GO:0042274~ribosomal small subunit biogenesis	3.97E-05	RPS28, RPS19, RPS16, RPS14, RPS15, NPM1, RPS6, RPS7, RPS24	1.36E-03
GO:0002366~leukocyte activation during immune response	4.27E-05	GPR183, ICAM1, YWHAZ, SBNO2, LYN, RELB, TLR2, MYO1F, STXBP2, ADA, LAT, SLC11A1, LAT2, PSEN1, TICAM1, BCL3, LCP1	1.45E-03
GO:0002263~cell activation during immune response	4.27E-05	GPR183, ICAM1, YWHAZ, SBNO2, LYN, RELB, TLR2, MYO1F, STXBP2, ADA, LAT, SLC11A1, LAT2, PSEN1, TICAM1, BCL3, LCP1	1.45E-03
GO:0046034~ATP metabolic	4.45E-05	ATP5D, ALDOA, ATP5E,	1.50E-03

process		ATP6V0E1, ATP1B3, ATP5B, ATP6AP1, PRKAG2, ATP5G2, MYO9B, ATP6V1B2, ATP5G1, ATP5G3, TGFB1, ATP6V0B, ATP2B1, ATP6V0C, ATP5L, ATP5O, ATP5I, ATP5H, ATP5J, TCIRG1, ATP5J2, ATP5F1, ATP6V1H, NADK, ATP1A1, ATP6V1F, ATP2C1, ATP2A3, ATP6V1E1, ATP5C1, ATP5A1	
GO:0006364~rRNA processing	4.51E-05	NAF1, RPL14, UTP6, EXOSC10, RPS28, DKC1, RPL7, FRG1, RPL5, RPL11, FTSJ1, IMP4, NSA2, RPS24, RPL35A, EXOSC8, RPL26, EXOSC1, RPS6, NOP10, FBL, RPS7, EIF4A3, DDX56, RPS19, RPS16, RPS14, RPS15, NOP58, POP4, NOP56	1.51E-03
GO:0045454~cell redox homeostasis	4.76E-05	GLRX3, TXNL1, SEPW1, P4HB, PDIA3, TXN2, C11ORF31, PDIA6, EGLN2, PRDX5, PRDX2, PRDX1, SELT, DDIT3, TXNDC12, TXNDC11, PRDX6, TXN, TXNRD2, SH3BGRL3, QSOX1, APEX1, SCO2, GLRX	1.58E-03
GO:0006909~phagocytosis	5.98E-05	ADORA2A, HCK, CDC42SE1, ABCA1, VAV1, ELMO1, CORO1C, SLC11A1, CORO1A, LRP1, CD36, CD93, JMJD6, CDC42SE2, FCN1, FCER1G, CLEC7A, THBS1, PTX3, CD14	1.97E-03
GO:0006163~purine nucleotide metabolic process	6.75E-05	ATP5D, ATP5E, ATP6V0E1, ATP1B3, ATP6AP1, ATP5B, PRKAG2, RHOQ, TGFB1, ATP2B1, ATP5L, ATP5O, ATP5I, ATP5H, IMPDH1, IMPDH2, ATP5J, NUDT1, CECR1, ATP6V1H, PDE4D, DGUOK, ATP6V1F, ADM,	2.21E-03

		ATP2C1, ADK, ATP5C1, ALDOA, ADORA2A, ATP5G2, MYO9B, ATP6V1B2, ATP5G1, ATP5G3, ADA, ATP6V0B, GCH1, ATP6V0C, RAB11A, TCIRG1, ATP5J2, ATP5F1, NADK, ATP1A1, AMPD2, AMPD3, NME1-NME2, ATP2A3, ATP6V1E1, ATP5A1, GUK1	
GO:0006461~protein complex assembly	7.18E-05	HRAS, OXA1L, XPO6, TSPAN4, CAPZA1, TGFB1, CTNNB1, SLC7A7, PICALM, ILK, PCBD1, TAZ, SPAG9, ADRM1, VAMP8, TUBA4A, VAMP3, ATPIF1, SURF1, SEPT9, TRAF1, HSD17B10, DIAPH1, GRB2, ARPC4, CALR, PDSS1, SRC, CD74, NPM1, RAC1, CDA, TAF9, SKIL, TAF7, SMAD3, GAS7, P2RX4, VCP, AP2S1, MKI67IP, NDUFAF3, NDUFS5, MAZ, NDUFS4, GSN, GPX3, CASP8, GTF2A2, TUBB6, NRG1, TUBA1A, TUBA1B, TUBA1C, PILRB, PPP2R1A, IRAK1, STX4, ATPAF2, HSP90AA1, PFKL, CCDC88C, CCNH, DECR1, NLRP3, MBD2, SLC9A3R1, CDK5, HMGA1, WAS, GTF2B, FLNA, NCK2, TAF10, MED4, ADRB2, TBCA, SBF2, UQCRH, IPO5, MDM2, TOMM22, PRNP, MAP3K11, POLR2G, POLR2F, POLR2E, POLR2L, POLR2I, PML, FKBP1A, GCH1, TNFRSF1A, LPXN, PTK2B, ALOX5AP, HSPA4, SCO2, TCP1, YWHAB, ANXA5, BIRC3, BIRC2, TRIM22, TRIM21, SOD2, CYBA,	2.33E-03

		PSMG2, PRLR, IRF7, BAX, TCEB2, TRPC4AP	
GO:0070271~protein complex biogenesis	7.18E-05	HRAS, OXA1L, XPO6, TSPAN4, CAPZA1, TGFB1, CTNNB1, SLC7A7, PICALM, ILK, PCBD1, TAZ, SPAG9, ADRM1, VAMP8, TUBA4A, VAMP3, ATPIF1, SURF1, SEPT9, TRAF1, HSD17B10, DIAPH1, GRB2, ARPC4, CALR, PDSS1, SRC, CD74, NPM1, RAC1, CDA, TAF9, SKIL, TAF7, SMAD3, GAS7, P2RX4, VCP, AP2S1, MKI67IP, NDUFAF3, NDUFS5, MAZ, NDUFS4, GSN, GPX3, CASP8, GTF2A2, TUBB6, NRG1, TUBA1A, TUBA1B, TUBA1C, PILRB, PPP2R1A, IRAK1, STX4, ATPAF2, HSP90AA1, PFKL, CCDC88C, CCNH, DECR1, NLRP3, MBD2, SLC9A3R1, CDK5, HMGA1, WAS, GTF2B, FLNA, NCK2, TAF10, MED4, ADRB2, TBCA, SBF2, UQCRH, IPO5, MDM2, TOMM22, PRNP, MAP3K11, POLR2G, POLR2F, POLR2E, POLR2L, POLR2I, PML, FKBP1A, GCH1, TNFRSF1A, LPXN, PTK2B, ALOX5AP, HSPA4, SCO2, TCP1, YWHAB, ANXA5, BIRC3, BIRC2, TRIM22, TRIM21, SOD2, CYBA, PSMG2, PRLR, IRF7, BAX, TCEB2, TRPC4AP	2.33E-03
GO:0051101~regulation of DNA binding	7.49E-05	SIVA1, TLR2, UBE2V1, NFKBIA, PRDX2, IL10, TGFB1, TRIB1, RPS3, KDM1A, MYD88, GTF2A2, TICAM1, NPM1, PYCARD, IL1B, BCL3, APEX1, IRAK2, ICAM1, IRAK1, IL6, SP100, RELA, PIM1,	2.42E-03

		TMEM189, NLRP3, DDIT3, FLNA, UBE2N, HDAC5, NME1-NME2, ID2, CALM3, EDF1, RIPK2, IKBKB, CALM2, CALM1, SMARCA4	
GO:0019221~cytokine-mediated signaling pathway	1.05E-04	CSF3, CCL2, CCR1, STAT5A, FKBP1A, PF4, STAT6, TNFRSF1A, STAT4, PYCARD, IL1B, SH2B2, IL1A, IRAK2, KLF6, PLP2, IRAK1, IL6, SP100, RELA, BAD, STAT1, STAT3, TXNDC17, EREG	3.36E-03
GO:0002274~myeloid leukocyte activation	1.11E-04	CSF2, YWHAZ, SBNO2, IL8, LYN, RELB, SNCA, TLR2, MYO1F, STXBP2, TGFB1, CD48, LAT, SLC11A1, LAT2, CD93, JMJD6, TICAM1, LCP2	3.53E-03
GO:0007265~Ras protein signal transduction	1.11E-04	HRAS, USP8, GRB2, MAPKAPK3, SHOC2, MYO9B, MAPKAPK2, ABCA1, RFXANK, RHOU, SRC, ARHGAP4, RASGRP2, RHOA, RALA, RHOG, ARHGDIB, DBNL, RSU1, PLD1, ARHGEF1, MAP2K2, YWHAB, RAF1, RALGDS, PARK7, ELMO1, LAT, CRKL, DOK3, GNB1, CFL1, SDCBP	3.52E-03
GO:0002696~positive regulation of leukocyte activation	1.36E-04	STAT5A, TNFSF14, TNFSF13, IL15, PNP, ADA, TGFB1, CD74, SART1, STAT6, CD47, IL4R, PVRL2, TICAM1, IL1B, RARA, IL2RG, BCL6, CD4, THBS1, FCER1A, PTPRC, IL6, IKZF1, BAD, NCK2, CD83, CORO1A, CDKN1A, CD86, TNFSF13B, RIPK2, SASH3	4.26E-03
GO:0043623~cellular protein complex assembly	1.39E-04	OXA1L, XPO6, DIAPH1, AP2S1, ARPC4, FKBP1A, CALR, SRC, TGFB1, NDUFAF3, NDUFS5, NDUFS4, PICALM, PTK2B, GSN, RAC1,	4.33E-03

		TUBB6, HSPA4, NRG1, TUBA1A, TUBA1B, PILRB, SCO2, TUBA1C, ATPAF2, TCP1, HSP90AA1, TAZ, SMAD3, MBD2, CDK5, GAS7, WAS, FLNA, NCK2, CYBA, ADRM1, ADRB2, PSMG2, PRLR, TBCA, IPO5, TUBA4A, TOMM22, SURF1	
GO:0006164~purine nucleotide biosynthetic process	1.45E-04	ATP5D, ALDOA, ATP5E, ATP6V0E1, ATP1B3, ADORA2A, ATP5B, ATP6AP1, PRKAG2, ATP5G2, ATP5G1, ATP6V1B2, ATP5G3, TGFB1, ADA, ATP6V0B, ATP2B1, ATP6V0C, ATP5L, ATP5O, ATP5I, ATP5H, IMPDH1, IMPDH2, ATP5J, TCIRG1, ATP5J2, ATP5F1, CECR1, ATP6V1H, ATP1A1, AMPD2, AMPD3, ATP6V1F, NME1-NME2, ADM, ATP2A3, ATP2C1, ADK, ATP6V1E1, ATP5C1, ATP5A1	4.49E-03
GO:0050867~positive regulation of cell activation	1.49E-04	STAT5A, TNFSF14, TNFSF13, IL15, PNP, ADA, TGFB1, CD74, SART1, STAT6, CD47, IL4R, PVRL2, TICAM1, IL1B, RARA, CD4, IL2RG, BCL6, THBS1, FCER1A, PTPRC, IL6, IKZF1, PLEK, BAD, NCK2, CD83, CORO1A, CDKN1A, CD86, TNFSF13B, RIPK2, SASH3	4.58E-03
GO:0051090~regulation of transcription factor activity	1.83E-04	SIVA1, TLR2, UBE2V1, NFKBIA, PRDX2, IL10, TGFB1, TRIB1, RPS3, KDM1A, MYD88, GTF2A2, TICAM1, NPM1, PYCARD, IL1B, IRAK2, ICAM1, IRAK1, IL6, SP100, RELA, PIM1, TMEM189, NLRP3, DDIT3, FLNA, UBE2N, HDAC5, ID2, RIPK2,	5.57E-03

		IKBKB, SMARCA4	
GO:0042273~ribosomal large subunit biogenesis	1.85E-04	RPL35A, RPL14, RPL7, NPM1, RPL26, RPL5, RPL11, RPL24	5.61E-03
GO:0006979~response to oxidative stress	1.88E-04	NDUFB4, PTGS2, TXN2, CLU, SNCA, PML, ROMO1, PRDX5, PRDX2, PDLIM1, ADRBK1, TPM1, PRDX1, ADA, SLC11A2, HMOX2, FOS, MSRA, PTK2B, GPX4, GPX3, FOSL1, NDUFS2, ERCC1, NAPRT1, NUDT1, OLR1, RELA, SELK, NDUFA6, STAT1, SOD1, NDUFA12, PARK7, DDIT3, SOD2, MTF1, DUSP1, PSEN1, PRDX6, UCP2, TXNRD2, PRNP, PPP1R15B, HDAC6	5.66E-03
GO:0006935~chemotaxis	2.13E-04	NRP2, CXCL1, C3AR1, HRAS, CCL2, IL16, CXCL3, CCR1, CXCL2, S100A9, FPR1, PF4, ITGB2, CCL7, IL10, ITGAM, CXCL10, CCRL2, DOCK2, TYMP, RAC2, CCL20, CXCR4, CKLF, RAC1, RALA, IL1B, FOSL1, PLP2, IL6, PLD1, RNASE2, C5AR1, IL8, PLAUR, CORO1A, CCR7, PPBP, CCR5, LYST, CXCL16, CMTM7, PLAU, CMTM5	6.35E-03
GO:0042330~taxis	2.13E-04	NRP2, CXCL1, C3AR1, HRAS, CCL2, IL16, CXCL3, CCR1, CXCL2, S100A9, FPR1, PF4, ITGB2, CCL7, IL10, ITGAM, CXCL10, CCRL2, DOCK2, TYMP, RAC2, CCL20, CXCR4, CKLF, RAC1, RALA, IL1B, FOSL1, PLP2, IL6, PLD1, RNASE2, C5AR1, IL8, PLAUR, CORO1A, CCR7, PPBP, CCR5, LYST, CXCL16, CMTM7, PLAU, CMTM5	6.35E-03
GO:0032446~protein modification by small protein conjugation	2.32E-04	TSG101, UBE2V1, NEDD8, CDC34, ANAPC11, STUB1, OS9, RBX1, PRPF19,	6.88E-03

		UBE2D3, UBE2D2, RNF167, NEDD4L, UBE2D1, FBXO9, RPS27A, TRIP12, RNF144B, UBE2A, UBE2F, UBE2H, TMEM189, ATG3, UBE2B, RBBP6, UBE2N, NOSIP, RNF7, VCP, UBE2M, UBC, MDM2, PAF1, UBB, RNF40, UBA52, UBE2E1, FBXO11, HDAC6	
GO:0022618~ribonucleoprotein complex assembly	2.34E-04	CLNS1A, EIF6, NCBP1, SNRPD3, RBM5, SF1, SNRPD1, SNRPD2, EIF2A, RPL24, MBNL1, PIH1D1, RPS14, RPS15, NPM1, USP39, SNRPB, SLU7, CELF1, SNRPC, SNRPF, SNRPE, TXNL4A, SNRPG	6.90E-03
GO:0006839~mitochondrial transport	2.34E-04	ATP5D, BID, TSPO, GRPEL1, YWHAZ, HSP90AA1, TIMM17A, CHKB, TIMM17B, TIMM10, AIP, PPIF, STARD3, TOMM7, PSEN1, UCP2, TOMM5, BAX, TIMM9, TOMM20, SLC25A37, ATP5O, TOMM22, SLC25A1	6.90E-03
GO:0030595~leukocyte chemotaxis	2.59E-04	IL6, CCL2, IL16, IL8, CXCL3, S100A9, PF4, ITGB2, ITGAM, IL10, CORO1A, DOCK2, CXCL16, LYST, CKLF, IL1B	7.59E-03
GO:0009165~nucleotide biosynthetic process	2.69E-04	ATP5D, ATP5E, NAMPT, KYNU, ATP6V0E1, ATP1B3, ATP6AP1, ATP5B, PRKAG2, PNP, TGFB1, ATP2B1, ATP5L, ATP5O, ATP5I, ATP5H, IMPDH1, IMPDH2, ATP5J, NAPRT1, NADSYN1, CECR1, ATP6V1H, ATP6V1F, ADM, ATP2C1, ADK, ATP5C1, ALDOA, ADORA2A, KMO, ATP5G2, ATP6V1B2, ATP5G1, ATP5G3, ADA, ATP6V0B, ATP6V0C,	7.82E-03

		TCIRG1, ATP5J2, ATP5F1, ATP1A1, AMPD2, AMPD3, NME1-NME2, ATP2A3, ATP6V1E1, DPYD, ATP5A1	
GO:0007242~intracellular signaling cascade	2.74E-04	HRAS, STAT5A, RAB1B, RAB1A, CTNNB1, HMHA1, RAB28, RAB24, GNG2, RARA, TLK2, RAPGEF2, RAB20, GNG5, GTPBP2, DBNL, PLD1, C5AR1, RAN, TANK, RAD1, SPAG9, CD36, SPAG5, ACAP1, ARL8A, RAB13, ARL8B, RAB10, GNAI2, NFKBIB, HUS1, DUSP10, STK17B, NFKBIA, AKAP13, ARF6, MYO9B, ARF5, CALR, CD74, NR1H2, RAC2, RAC1, TYROBP, FCER1A, ABR, MAP2K2, MAP2K3, TAOK3, SPHK1, DUSP22, RPS6, PTPN11, RGS1, ARF3, CNIH4, ARF4, RIT1, CLEC7A, TREML1, SLC9A1, RAB5C, TOLLIP, SHOC2, STAC3, RFXANK, NSMCE1, TICAM1, ANP32A, DEDD2, AGAP3, ZFP36, ARHGEF2, ARHGEF1, RAP2C, LYN, LY96, RAB4B, PI4KB, FLNA, ELMO1, MED4, DOK3, TNFRSF10B, ADM, PSEN1, GNB2, GNB1, RIN2, GNAS, GADD45B, ARL4C, MAP3K11, CXCL1, CARHSP1, USP8, NMI, CREM, CCR1, MKNK2, FPR1, PML, DGKA, STAT4, PTK2B, SQSTM1, PPP2CA, RAB11A, BCL3, THBS1, RAB2A, GDI1, GDI2, IL8, PLEK, HCLS1, YWHAB, STAT1, SOD1, YWHAE, STAT3, RAB32, RAB31, DUSP2, DUSP1, RPS6KA1, FYN, GSK3B, ARAF,	7.91E-03

		RAB34, YWHAQ, CHN2, APBB3, DUSP6, GNA15, RBM4, RASGEF1B, TLR2, TNFSF14, RPS6KB2, RGL4, RPS27L, PRKCSH, IL10, IL1B, RALA, PRKACA, SAR1A, SOCS3, MADD, FOXN3, MAP4K3, MAP4K4, LAT2, SDCBP, STYXL1, RAB7A, HMGB2, CCL2, ADORA2A, GRB2, SIPA1, MAPKAPK3, MAP4K1, ABCA1, MAPKAPK2, AZI2, SRC, DNAJA1, ARHGDIB, RAB8B, TAF7, KAT5, VAV1, TRAF3IP2, LAT, TXNDC12, PRKAR1A, RAP1A, RAP1B, IKBKB, NBN, PRKAG2, RHOQ, PRDX2, RHOU, BUD31, ARHGAP4, CDC42, NDUFS4, GSN, CXCR4, RHOA, RHOC, PAK1, PLCB1, TNIP2, RHOF, PLCB2, RHOG, RHOH, IRAK2, IRAK1, PPP2R1A, RSU1, SP100, MINK1, PKN1, ADIPOR1, IFI16, PRKCE, PRKCD, CDK5, MCTP1, TYK2, OSM, IFNAR2, PRKD2, ADRB2, CRKL, ITGB1BP1, DEF8, CFL1, TREM1, FKBP8, EGLN2, NR3C1, TRIB1, RASGRP4, RASGRP2, SH2B2, NFATC1, PTPRC, RAF1, RALGDS, PARK7, GPS2, WSB1, PRLR, CKS2, BRE, SMPD2	
GO:0006446~regulation of translational initiation	2.74E-04	NCBP1, PAIP2, EIF5, EIF1B, ANKHD1, EIF4B, EIF4G2, EIF4EBP1, EIF2AK1, EIF3B, EIF3H, EIF4A2, EIF3E, EIF4H, EIF3K, EIF1, EIF2B5	7.88E-03
GO:0007568~aging	2.89E-04	HSD17B10, LIMS1, HRAS, GRB2, TPRA1, ATP6AP1, PML, ROMO1, ADRBK1, IL15, CALR, PDCD4,	8.24E-03

		PTEN, ATP5G3, TGFB1, ADA, FOS, GSN, GPX4, ILK, NPM1, IL1B, ERCC1, POLG, SOCS3, RELA, SOD1, PRKCD, DDIT3, SOD2, LRP1, ADM, CTSC	
GO:0043388~positive regulation of DNA binding	2.97E-04	TLR2, UBE2V1, TGFB1, IL10, MYD88, NPM1, GTF2A2, TICAM1, PYCARD, IL1B, IRAK2, ICAM1, IRAK1, IL6, SP100, RELA, TMEM189, HDAC5, UBE2N, NME1-NME2, RIPK2, EDF1, CALM3, IKBKB, CALM2, CALM1, SMARCA4	8.43E-03
GO:0007010~cytoskeleton organization	3.02E-04	MTSS1, HRAS, PDLIM7, PRC1, CAPZA1, S100A9, CTNNB1, ACTG1, RALA, PPP4C, RAN, ACTN1, TMSB10, MYH9, DCTN2, ARPC1A, UXT, ATP2C1, SPAG5, CNTROB, SDCBP, BIN3, ALDOA, AGFG1, DIAPH1, SSH2, SIPA1, NEDD9, MYO9B, ARF6, ARPC4, ARPC5, CALR, CAPZB, PFN1, DOCK2, RAC2, BLOC1S2, NPM1, RAC1, ARHGDIB, EVL, GAS7, CORO1A, HDAC3, DNAJB6, TLN1, RHOQ, RHOU, OFD1, ARHGAP4, CDC42, DYNLL1, GSN, RHOA, MICAL1, PAK1, CAP1, RHOF, TUBA1B, FMNL1, ARHGEF2, UBE2B, CDK5, WAS, VASP, FLNA, ARHGAP26, ELMO1, NCK2, EPB41L3, CFL1, TMSB4X, FHOD1, LCP1, HAUS4, CXCL1, HAUS2, FHL3, CYTH2, TPM1, EZR, INPP5K, PTK2B, CNN2, SH2B2, BCL6, PAFAH1B1, TRIP10, PLEK, RAF1, SOD1, GABARAP, ERBB2IP, SVIL, CKS2, ARAP1	8.51E-03
GO:0010604~positive	3.08E-04	MEF2C, NCBP1, HRAS,	8.62E-03

regulation of macromolecule metabolic process		AURKAIP1, STAT5A, XRCC6, SNCA, RBM4, MORF4L2, EIF5A, CASK, TNFSF13, TGFB1, IL10, CTNNB1, CITED2, EPC1, SMARCD3, ATG7, WIBG, IL1B, SUPT5H, AKIRIN2, IL1A, RPS27A, EIF2B5, ANAPC5, PCBD1, RAN, RELA, MTA2, NRD1, TNFRSF14, SKP1, AHR, DDIT3, JUNB, HES1, PSMA2, PSMA1, MTF1, PSMA6, PSMA5, PSMA4, VEGFA, PSMA3, NFE2L2, UBA52, SMARCA4, CAMTA2, CNBP, HMGB2, TFE3, NFKBIA, ANAPC10, ANAPC11, CALR, PSMA7, NR1H2, SLC11A1, PSMB4, PSMB7, ECE1, PSMB6, PSMB1, BLOC1S2, PSMB2, PEMT, RNF10, TAF9, UBE2D1, RUNX1, HNRNPAB, FCER1A, KLF6, TESC, IKZF1, KLF13, MAFB, MAP2K3, TAF7, SRA1, NR4A1, SMAD3, KAT5, DDX5, USF2, HDAC5, ATF4, PSMC5, PSMC4, HDAC1, PSMC3, PSMC2, CSRNP1, ETS2, KLF4, HDAC6, UBE2E1, ENY2, CHURC1, PRKAG2, SPI1, RHOQ, NFKB1, FOS, TICAM1, GTF2A2, PSMD1, SERTAD3, PSMD2, PSMD4, PSMD6, PSMD7, FOSL1, SERTAD1, PSMD8, PSMD9, SERTAD2, IRAK1, SP100, LYN, CCNH, RPS4X, HMGA1, UBE2N, OSM, CD86, ADRB2, CCND3, TNFSF13B, EREG, PSEN1, PSME1, PSME2, UBC, MDM2, UBB, PSMB10, CSF2, PML, PPM1A, EGLN2, FKBP1A, PF4, STUB1, TCF7L2,	

		STAT6, TNFRSF1A, PSMF1, PTK2B, SQSTM1, BCL3, CD4, SUPT4H1, THBS1, TINF2, PTPRC, IL6, CEBPB, HCLS1, TRIM28, CREB5, ILF3, STAT3, ATXN1, PSMD13, ILF2, PSMD11, IRF1, BRE, PHF5A, SH3D19	
GO:0051251~positive regulation of lymphocyte activation	3.29E-04	STAT5A, TNFSF14, TNFSF13, IL15, PNP, ADA, TGFB1, CD74, SART1, STAT6, CD47, IL4R, TICAM1, IL1B, RARA, IL2RG, BCL6, CD4, PTPRC, IL6, IKZF1, BAD, CD83, NCK2, CORO1A, CDKN1A, CD86, TNFSF13B, RIPK2, SASH3	9.16E-03
GO:0002684~positive regulation of immune system process	3.42E-04	STAT5A, TLR2, TNFSF14, TNFSF13, IL15, PNP, SART1, TGFB1, B2M, CFP, CD47, MYD88, IL4R, TICAM1, IL1B, RARA, IRAK2, IRAK1, ICAM1, LYN, RELA, UBE2N, NCK2, CD83, CD37, CD86, LAT2, EREG, PSEN1, TNFSF13B, VEGFA, RIPK2, C3AR1, CLU, NFKBIA, CD74, ADA, STAT6, SLC11A1, MIA3, PVRL2, FCER1G, CD4, IL2RG, SH2B2, BCL6, THBS1, FCER1A, PTPRC, IL6, IKZF1, BAD, FOXP1, CORO1A, CD55, CDKN1A, FYN, CLEC7A, SASH3	9.45E-03
GO:0000302~response to reactive oxygen species	3.47E-04	PRDX5, ROMO1, ADRBK1, PRDX2, PRDX1, TPM1, ADA, FOS, PTK2B, GPX4, GPX3, FOSL1, OLR1, RELA, SOD1, STAT1, DDIT3, PARK7, SOD2, DUSP1, PRDX6, UCP2, TXNRD2, PPP1R15B, HDAC6	9.53E-03
GO:0034654~nucleobase, nucleoside, nucleotide and nucleic acid biosynthetic	3.52E-04	ATP5D, ATP5E, NAMPT, KYNU, ATP6V0E1, ATP1B3, ATP6AP1,	9.62E-03

process		ATP5B, PRKAG2, PNP, TGFB1, ATP2B1, ATP5L, ATP5O, ATP5I, ATP5H, IMPDH1, IMPDH2, ATP5J, NAPRT1, NADSYN1, CECR1, ATP6V1H, ATP6V1F, ADM, ATP2C1, ADK, ATP5C1, ALDOA, ADORA2A, KMO, ATP5G2, ATP6V1B2, ATP5G1, ATP5G3, ADA, ATP6V0B, ATP6V0C, CDA, TCIRG1, ATP5J2, ATP5F1, ATP1A1, AMPD2, AMPD3, NME1-NME2, ATP2A3, ATP6V1E1, DPYD, ATP5A1	
GO:0034404~nucleobase, nucleoside and nucleotide biosynthetic process	3.52E-04	ATP5D, ATP5E, NAMPT, KYNU, ATP6V0E1, ATP1B3, ATP6AP1, ATP5B, PRKAG2, PNP, TGFB1, ATP2B1, ATP5L, ATP5O, ATP5I, ATP5H, IMPDH1, IMPDH2, ATP5J, NAPRT1, NADSYN1, CECR1, ATP6V1H, ATP6V1F, ADM, ATP2C1, ADK, ATP5C1, ALDOA, ADORA2A, KMO, ATP5G2, ATP6V1B2, ATP5G1, ATP5G3, ADA, ATP6V0B, ATP6V0C, CDA, TCIRG1, ATP5J2, ATP5F1, ATP1A1, AMPD2, AMPD3, NME1-NME2, ATP2A3, ATP6V1E1, DPYD, ATP5A1	9.62E-03
GO:0034655~nucleobase, nucleoside, nucleotide and nucleic acid catabolic process	3.54E-04	ATP5D, ATP5E, FHIT, NUDT1, ATP5B, NUDT5, UPP1, RHOQ, MYO9B, PDE4D, AMPD3, PNP, ADA, GCH1, DERA, RAB11A, CDA, ATP5O, DPYD, ATP5A1, NT5C	9.60E-03
GO:0034656~nucleobase, nucleoside and nucleotide catabolic process	3.54E-04	ATP5D, ATP5E, FHIT, NUDT1, ATP5B, NUDT5, UPP1, RHOQ, MYO9B, PDE4D, AMPD3, PNP, ADA, GCH1, DERA, RAB11A, CDA, ATP5O,	9.60E-03

		DPYD, ATP5A1, NT5C	
GO:0006914~autophagy	3.66E-04	GABARAPL2, CLN3, BECN1, MAP1LC3B2, C12ORF44, ATG3, LAMP1, ATG4D, PSEN1, MAP1LC3B, ATG4B, ATG7, RAB24, ATG16L2, DRAM1, HDAC6	9.88E-03
GO:0006919~activation of caspase activity	3.68E-04	SIVA1, CYCS, PML, SMAD3, PMAIP1, NLRP3, BCL2L13, STAT1, NLRP1, RPS3, SLC11A2, TNFRSF10B, VCP, BAX, IFT57, F3, MTCH1, PYCARD, DIABLO, HSPE1	9.87E-03
GO:0044270~nitrogen compound catabolic process	4.03E-04	ATP5D, ATP5E, FHIT, NUDT1, ATP5B, NUDT5, UPP1, RHOQ, MYO9B, PDE4D, AMPD3, PNP, ADA, GCH1, BLVRA, HMOX2, DERA, RAB11A, CDA, ATP5O, DPYD, ATP5A1, NT5C	1.07E-02
GO:0008624~induction of apoptosis by extracellular signals	4.11E-04	BID, APH1A, ADORA2A, AKAP13, CRADD, ARHGAP4, PLEKHG2, SQSTM1, CASP8, RAC1, TICAM1, DIABLO, PSENEN, DAP, DEDD2, RPS27A, CFLAR, ARHGEF2, ABR, TM2D1, NDUFA13, BAD, VAV1, YWHAE, BCL2L11, TNFRSF10B, PSEN1, BAX, UBC, UBB, PDCD6, UBA52, SMPD2	1.09E-02
GO:0048193~Golgi vesicle transport	4.24E-04	STX8, AP1M1, CLTA, NRBP1, COPZ1, USE1, RER1, NAPA, FTH1, AP1S2, COPB1, BLOC1S1, TMED10, SAR1B, SAR1A, CLINT1, STX10, HSPA8, FTL, RAB2A, GABARAPL2, STX4, SCAMP2, VTI1B, OPTN, ERGIC1, COG3, VCP, VAMP8, AP2A1, SEC13, SH3D19, CUX1, TRAPPC1, TRAPPC3, COPE, DNM2	1.12E-02
GO:0010952~positive	4.57E-04	SIVA1, CARD8, CYCS,	1.20E-02

regulation of peptidase activity		PML, SMAD3, PMAIP1, NLRP3, BCL2L13, STAT1, NLRP1, RPS3, SLC11A2, TNFRSF10B, VCP, BAX, IFT57, F3, MTCH1, PYCARD, DIABLO, HSPE1	
GO:0043280~positive regulation of caspase activity	4.57E-04	SIVA1, CARD8, CYCS, PML, SMAD3, PMAIP1, NLRP3, BCL2L13, STAT1, NLRP1, RPS3, SLC11A2, TNFRSF10B, VCP, BAX, IFT57, F3, MTCH1, PYCARD, DIABLO, HSPE1	1.20E-02
GO:0010647~positive regulation of cell communication	4.62E-04	HRAS, SLC44A2, VAPA, PTGS2, SNCA, SHOC2, ZEB2, TGFB1, CTNNB1, CITED2, MYD88, TMEM9B, ILK, TICAM1, CASP8, RHOA, IL1B, RHOC, CASP1, RPS27A, BST2, LYN, RELA, PIM2, TMEM189, MBD2, FLNA, UBE2N, OSM, ADRB2, TNFRSF10B, EREG, ATP2C1, F3, VEGFA, UBC, RIPK2, UBB, EEF1D, UBA52, CSF2, FKBP8, CCL2, LITAF, ADORA2A, PPM1A, UBE2V1, FKBP1A, ADA, SRC, TNFRSF1A, ECE1, SLC35B2, RASGRP4, SHISA5, RAC1, CD4, NENF, THBS1, FCER1A, PTPRC, CFLAR, SECTM1, IL6, BECN1, HCLS1, LGALS1, TAOK3, DUSP22, BIRC2, LGALS9, PTPN11, TRAF3IP2, P2RX4, APOL3, TNFSF10, HDAC6	1.20E-02
GO:0045580~regulation of T cell differentiation	4.99E-04	PTPRC, IKZF1, STAT5A, PRDX2, BAD, IL15, SOD1, PNP, ADA, CD74, SART1, CLPTM1, CD83, CD86, IL4R, RARA, BCL6, IL2RG, SASH3	1.29E-02
GO:0006508~proteolysis	5.03E-04	SCPEP1, PPP2R5C, ZNRF1, CUL3, CFP, USP53, ISG15, PSENEN, ADAM8, AUP1, CAPNS1, ANAPC5, UBR4,	1.29E-02

		UBE2J1, MAP1LC3B2, SKP1, UBE2J2, MYH9, F3, RAD23A, UBA7, RFFL, CDC34, ARIH1, ARIH2, FBXO9, FBXO7, OSGEP, UBE2L6, CD55, RNF7, UBA1, PLAU, SPG7, KIAA0368, TSG101, RNPEPL1, UFC1, ANPEP, NFKB1, SENP5, MAP1LC3B, NSMCE2, DPP7, USP15, DPEP2, PEPD, UFD1L, MMP19, HERC4, GZMB, SENP3, PSEN1, CARD16, CARD17, ADAM19, RNPEP, ADAM15, USP7, USP8, USP3, SHFM1, CYLD, SQSTM1, USP39, USP36, TRIP12, UBXN1, BIRC6, CAPN2, MARCH6, CAPN1, MARCH3, DNPEP, MARCH2, PSMD13, KCMF1, PSMD11, TCEB2, TCEB1, RNF19B, UBXN6, ATP6AP2, CRADD, ATG7, RABGEF1, RBCK1, RNF149, RPS27A, SOCS3, RELA, NRD1, PSMA2, PSMA1, SEC61B, PSMA6, KDM2A, PSMA5, PSMA4, PSMA3, CLPP, TNFAIP3, UBA52, FBXO11, DERL2, DERL1, AMZ2, CLU, UBE2V1, ANAPC10, CTSA, NEDD8, ANAPC11, PSMA7, RBX1, PSMB4, PSMB7, UBE2D3, UBE2D2, PSMB6, ECE1, PSMB1, PSMB2, RNF167, UBE2D1, SRGN, CFLAR, SEC11A, SUGT1, ATG3, ATE1, URM1, APEH, PSMC5, CRBN, PSMC4, VCP, PSMC3, OTUB1, PSMC2, SPCS3, SPCS1, UBE2E2, HDAC6, UBE2E1, UQCRC2, NPEPL1, UQCRC1, BAP1, OS9, CASP4, TPP2, CASP8,	

		PSMD1, PSMD2, KLHL21, PSMD4, DDA1, PSMD6, CASP1, PSMD7, PSMD8, PSMD9, NRIP3, UBE2A, CTSZ, UBE2F, UBE2I, UBE2H, TMEM189, UBE2B, CTSW, UBE2N, LAP3, EML2, IMMP1L, ATG4D, PSME1, UBE2K, ATG4B, PSME2, UBE2M, UBC, MDM2, CTSC, UCHL3, UBB, CTSH, PSMB10, UBE2Z, APH1A, STUB1, UBE2R2, PSMF1, PYCARD, NEDD4L, UBL7, RNF144B, OLR1, CPVL, UBE2Q1, XPNPEP1, UBL5, WSB1, HSP90B1, LTA4H, RNF40	
GO:0060326~cell chemotaxis	5.09E-04	IL6, CCL2, IL16, IL8, CXCL3, S100A9, PF4, ITGB2, ITGAM, IL10, CORO1A, DOCK2, CXCL16, LYST, CKLF, IL1B	1.30E-02
GO:0051235~maintenance of location	5.47E-04	SRI, TLN1, PDIA3, NFKBIE, NFKBIB, YWHAB, PML, NFKBIA, TMSB10, CALR, FTH1, IL10, FLNA, OS9, HSP90B1, EZR, GAA, BCL3, IL1B, TMSB4X, KDELR1, SRGN	1.39E-02
GO:0051091~positive regulation of transcription factor activity	5.85E-04	IRAK2, IRAK1, ICAM1, IL6, SP100, RELA, TLR2, UBE2V1, TMEM189, IL10, TGFB1, UBE2N, HDAC5, MYD88, TICAM1, GTF2A2, NPM1, PYCARD, RIPK2, IL1B, IKBKB, SMARCA4	1.48E-02
GO:0009146~purine nucleoside triphosphate catabolic process	6.12E-04	ATP5D, ATP5E, NUDT1, ATP5B, RHOQ, RAB11A, ATP5O, MYO9B, ATP5A1, ADA, GCH1	1.54E-02
GO:0010466~negative regulation of peptidase activity	6.12E-04	HERPUD1, ADORA2A, CDKN2D, SNCA, CST3, CSTB, NR4A1, CSTA, HSPA5, IFI6, DNAJB6	1.54E-02
GO:0045582~positive	6.47E-04	PTPRC, IKZF1, STAT5A,	1.61E-02

regulation of T cell differentiation		BAD, PNP, CD74, SART1, ADA, CD83, CD86, IL4R, IL2RG, RARA, SASH3	
GO:0051099~positive regulation of binding	6.59E-04	TLR2, UBE2V1, FKBP1A, IL10, TGFB1, MYD88, GTF2A2, TICAM1, NPM1, PYCARD, IL1B, IRAK2, IRAK1, ICAM1, IL6, SP100, RELA, TMEM189, HDAC5, UBE2N, NME1-NME2, CALM3, EDF1, RIPK2, IKBKB, CALM2, CALM1, SMARCA4	1.64E-02
GO:0046496~nicotinamide nucleotide metabolic process	6.97E-04	NAMPT, LDHB, KYNU, TALDO1, PGD, NADSYN1, IDH3B, KMO, NADK, PNP, TPI1, PGLS, IDH3G, MDH2, MDH1, NAPRT1	1.72E-02
GO:0006769~nicotinamide metabolic process	6.97E-04	NAMPT, LDHB, KYNU, TALDO1, PGD, NADSYN1, IDH3B, KMO, NADK, PNP, TPI1, PGLS, IDH3G, MDH2, MDH1, NAPRT1	1.72E-02
GO:0048534~hemopoietic or lymphoid organ development	7.03E-04	XRCC5, STAT5A, PRDX2, NFKB2, IL15, TGFB1, IL10, CTNNB1, CDC42, CASP8, RHOH, CD3D, LYN, RELB, TAZ, MINK1, IFI16, MYH9, CD164, BCL2L11, CRKL, RPS19, PSEN1, RPS14, AHSP, VEGFA, CSF3, CSF2, GPR183, TIPARP, PML, CD74, ADA, TIMP1, TAL1, DOCK2, RASGRP4, BCL11A, BCL3, CD4, BCL6, RUNX1, ERCC1, EBP, KLF6, PTPRC, PLEK, IKZF1, JARID2, HCLS1, ROGDI, SOD1, FOXP1, SOD2, HDAC5, ID2, RPL22, JMJD6, BAX, IRF8, IRF1, HDAC9	1.72E-02
GO:0008284~positive regulation of cell proliferation	7.09E-04	NAMPT, NBN, HRAS, S100A6, FOSL2, PTGS2, MARCKSL1, STAT5A, ATP6AP1, NAP1L1, EIF5A, ROMO1, TNFSF13, IL15, PNP, TGFB1, CXCL10, CUL3, CD47, NDUFS4,	1.73E-02

		ILK, TICAM1, IL1B, NRG1, FOSL1, AKIRIN2, RHOG, SERTAD1, UBE2A, CAPNS1, LYN, RELA, RPS4X, PPP1CB, OSM, HES1, NCK2, CD86, ADRB2, ADM, EREG, TNFSF13B, F3, GRN, VEGFA, TGIF1, RIPK2, MDM2, DHPS, MCTS1, CSF3, CSF2, DERL2, CNBP, CCL2, MVD, GNAI2, CLU, RPS15A, CALR, ADA, TIMP1, RAC2, BLOC1S2, PTK2B, NPM1, CDC123, BCL6, TCIRG1, B4GALT1, PTPRC, ODC1, IL6, HCLS1, SPHK1, ROGDI, RPS9, BIRC6, STAT1, FOXP1, CAPN1, POLD4, CORO1A, CDKN1A, C19ORF10, ATF3, ID2, HDAC1, NME1-NME2, HBEGF, SASH3	
GO:0002520~immune system development	7.31E-04	XRCC5, NBN, STAT5A, XRCC6, PRDX2, NFKB2, IL15, TGFB1, IL10, CTNNB1, CTNNBL1, CDC42, CASP8, RHOH, CD3D, LYN, RELB, TAZ, MINK1, IFI16, MYH9, CD164, BCL2L11, CRKL, RPS19, PSEN1, RPS14, AHSP, VEGFA, CSF3, CSF2, GPR183, TIPARP, PML, CD74, ADA, TIMP1, TAL1, DOCK2, RASGRP4, BCL11A, BCL3, BCL6, CD4, RUNX1, ERCC1, EBP, KLF6, PTPRC, PLEK, IKZF1, JARID2, HCLS1, ROGDI, SOD1, FOXP1, SOD2, HDAC5, ID2, RPL22, JMJD6, BAX, IRF8, IRF1, HDAC9	1.77E-02
GO:0019674~NAD metabolic process	7.56E-04	NAMPT, LDHB, KYNU, IDH3G, NADSYN1, IDH3B, NADK, KMO, PNP, MDH2, NAPRT1, MDH1	1.82E-02

GO:0009611~response to wounding	7.74E-04	S100A8, S100A9, TLR2, IL15, IL10, TGFB1, CXCL10, LUZP6, CFP, MYD88, CD44, IL1B, IL1A, RELA, PLAUR, TNFAIP6, CCR7, CD36, CCR5, F3, RIPK2, C3AR1, CCL2, ADORA2A, TXN2, PABPC4, CLU, NINJ2, NINJ1, ITGB2, CCL7, SLC11A1, MIA3, IL23A, RAC1, B4GALT1, PTPN6, KLF6, MAP2K3, IL1RN, SMAD3, S100A12, HDAC5, APOL3, CD55, ITGA5, CLEC7A, TREML1, HDAC9, PLAU, TOLLIP, LY86, PRDX5, PRDX2, NFKB1, CD97, FOS, GSN, CXCR4, AOAH, TICAM1, ITIH4, MGLL, SERPINA1, NRG1, IRAK2, NFKBIZ, LYN, LY96, LYZ, NLRP3, CDK5, WAS, CD163, EREG, ADM, SERPINB2, KDM6B, CXCL1, YWHAZ, NMI, CXCL3, CCR1, CXCL2, PF4, TPM1, CD9, TNFRSF1A, TNFRSF1B, MEFV, CCL20, IL10RB, THBS1, PTX3, IL6, CEBPB, PLEK, IL8, OLR1, ANXA1, ANXA5, SOD1, STAT3, SOD2, CYBA, PLSCR1, CYBB, IRF7, BAX, HBEGF, LTA4H, CD14	1.86E-02
GO:0050900~leukocyte migration	7.96E-04	B4GALT1, ICAM1, IL6, CCL2, IL16, IL8, CXCL3, S100A9, ITGB2, PF4, MYH9, ITGAM, IL10, CORO1A, DOCK2, CXCL16, LYST, CKLF, IL1B, MSN	1.90E-02
GO:0032760~positive regulation of tumor necrosis factor production	8.54E-04	MYD88, TICAM1, TLR2, FCER1G, RIPK2, PF4, CLEC7A, SASH3, CD14	2.03E-02
GO:0043393~regulation of protein binding	8.55E-04	GTPBP4, PCBD1, FKBP1A, PRKCD, LRPAP1, HDAC5, ADRB2, AES, PSEN1, SMARCD3, BAX, TICAM1,	2.02E-02

		NRG1	
GO:0050871~positive regulation of B cell activation	9.15E-04	PTPRC, IL6, STAT5A, TNFSF13, BAD, TGFB1, ADA, STAT6, CDKN1A, TNFSF13B, TICAM1, BCL6, IL2RG, SASH3	2.15E-02
GO:0050863~regulation of T cell activation	9.41E-04	ADORA2A, STAT5A, TNFSF14, PRDX2, IL15, PNP, ADA, IL10, TGFB1, CD74, SART1, CD47, IL4R, IL1B, RARA, IL2RG, BCL6, CD4, PTPRC, IL6, IKZF1, TNFRSF14, BAD, SOD1, CLPTM1, LAT, CD83, NCK2, CORO1A, CD86, TNFSF13B, RIPK2, SASH3	2.19E-02
GO:0051092~positive regulation of NF-kappaB transcription factor activity	9.41E-04	IRAK2, IRAK1, ICAM1, IL6, RELA, TLR2, UBE2V1, TMEM189, TGFB1, UBE2N, MYD88, TICAM1, NPM1, PYCARD, RIPK2, IL1B, IKBKB	2.18E-02
GO:0009820~alkaloid metabolic process	9.41E-04	NAMPT, LDHB, KYNU, TALDO1, PGD, NADSYN1, IDH3B, KMO, NADK, PNP, TPI1, PGLS, IDH3G, MDH2, MDH1, NAPRT1	2.18E-02
GO:0008633~activation of pro-apoptotic gene products	9.65E-04	BID, TNFSF10, TNFRSF10B, DYNLL1, CASP8, YWHAB, SMAD3, PPP3CC, BAD, DEDD2, BCL2L11	2.23E-02
GO:0002694~regulation of leukocyte activation	9.66E-04	ADORA2A, STAT5A, SNCA, TNFSF14, PRDX2, TNFSF13, IL15, PNP, SART1, CD74, TGFB1, IL10, ADA, STAT6, CD47, IL4R, PVRL2, TICAM1, IL1B, RARA, CD4, BCL6, IL2RG, THBS1, FCER1A, PTPRC, IL6, IKZF1, STXBP2, TNFRSF14, BAD, SOD1, CLPTM1, LAT, NCK2, CD83, CORO1A, CDKN1A, CD86, TNFSF13B, PRAM1, RIPK2, SASH3	2.22E-02
GO:0006007~glucose catabolic process	1.01E-03	ALDOA, LDHB, LDHA, TALDO1, PFKL, ADPGK,	2.31E-02

		PGD, HK2, HK1, BAD, GPI, TPI1, PGLS, HK3, PDHA1, PGK1, GAPDH, MDH2, MDH1, ENO1	
GO:0000278~mitotic cell cycle	1.03E-03	MAD1L1, E2F4, PRC1, RHOU, CUL3, CDKN2D, TARDBP, PSMD1, PSMD2, PSMD4, PSMD6, PSMD7, PSMD8, RPS27A, PSMD9, CCNK, ARHGEF2, ANAPC5, RAN, PIM1, UBE2I, GPR132, SKP1, DCTN3, PPP1CB, FOXN3, EML4, DCTN2, PSMA2, PSMA1, CHMP1A, PSMA6, PSME1, SPAG5, CDK11A, PSMA5, PSME2, PSMA4, PSMA3, UBC, MDM2, MAPRE1, UBB, MAD2L2, KPNA2, UBA52, MAP3K11, HAUS4, PSMB10, PPP6C, HAUS2, NEDD9, ANAPC10, CDC34, ANAPC11, PSMA7, PSMB4, PSMF1, PSMB7, NIPBL, PSMB6, PSMB1, PSMB2, CDC123, PAFAH1B1, UBE2D1, NFATC1, SSSCA1, RPL24, PMF1, SUGT1, CDKN1A, PSMC5, PSMD13, PSMC4, PSMC3, PSMD11, PSMC2, MPHOSPH6, TXNL4A, DNM2, UBE2E1	2.33E-02
GO:0010551~regulation of specific transcription from RNA polymerase II promoter	1.03E-03	MEF2C, HMGB2, XRCC6, TFE3, NFKBIA, RHOQ, NFKB1, TCF7L2, CTNNB1, CITED2, NR1H2, KDM1A, TAL1, AES, GTF2A2, TAF9, IKZF1, TAF7, SMAD3, KAT5, USF2, GPS2, HDAC5, HES1, HDAC3, HDAC1, HDAC9, SMARCA4	2.33E-02
GO:0007049~cell cycle	1.05E-03	SEPT5, S100A6, PRC1, WTAP, SART1, TGFB1, CTNNB1, CUL3, MEI1, CDKN2D, TARDBP, ILK, MAP3K8, TLK2, SUPT5H, RPS27A, ANAPC5, RAN,	2.36E-02

		CHTF8, PIM1, GPR132, PIM2, SKP1, DCTN3, MYH9, PPP1CB, AHR, DDIT3, FOXN3, DCTN2, RAD1, PSMA2, PSMA1, REC8, PPP1CA, MAPK6, PSMA6, SPAG5, PSMA5, PSMA4, CNTROB, PSMA3, BIN3, PDCD6IP, MAD2L2, SEPT6, MCTS1, UBA52, SEPT9, ITGAE, NEDD9, ANAPC10, ANAPC11, CDC34, CALR, PSMA7, PSMB4, PSMB7, NIPBL, PSMB6, PSMB1, PSMB2, CDC123, NPM1, UBE2D1, LFNG, TFDP1, SSSCA1, CINP, SMAD3, PMF1, TET2, SUGT1, GAS7, CDKN1A, HDAC3, PLK3, PSMC5, RGS2, PSMC4, PSMC3, PSMC2, UBE2E1, MAD1L1, NBN, E2F4, TSG101, SENP5, RHOU, RNF103, PSMD1, PSMD2, PSMD4, PSMD6, PSMD7, PSMD8, PSMD9, CCNK, ARHGEF2, CCNH, SF1, UBE2I, CDK5, GAK, EML4, PPM1G, EIF4G2, PFDN1, CHMP1A, CHMP1B, CCND3, EREG, PSME1, PSME2, CDK11A, UBC, MDM2, G0S2, UBB, MAPRE1, KPNA2, PPP1R15A, MAP3K11, PSMB10, HAUS4, PPP6C, HAUS2, PML, ZNF655, TCF7L2, CYLD, PSMF1, PAFAH1B1, HBP1, THBS1, NFATC1, ERH, IL8, ANXA1, RPL24, ILF3, GPS2, RASSF4, PSMD13, ERBB2IP, DUSP1, PSMD11, GSK3B, PTP4A1, CKS2, CALM3, MPHOSPH6, CALM2, ARAP1, DNM2, CALM1, TXNL4A	
GO:0042127~regulation of	1.05E-03	RARRES3, S100A6, HRAS,	2.34E-02

cell proliferation		PTGS2, STAT5A, ATP6AP1, FGFRL1, RBM5, EIF5A, ROMO1, TNFSF13, IL15, PNP, IL10, TGFB1, CXCL10, CTNNB1, CUL3, CD47, MYD88, CDKN2D, ILK, IL1B, ASPH, AKIRIN2, IL1A, GTPBP4, CAPNS1, RELA, TNFRSF14, PPP1CB, HES1, EIF2AK1, F3, CD33, VEGFA, TGIF1, RIPK2, ATPIF1, EMP3, MCTS1, DERL2, CNBP, CCL2, GNAI2, MVD, IFITM1, ADORA2A, CLU, NFKBIA, IFI30, CALR, FTH1, ADA, TIMP1, BLOC1S2, RAC2, CDC123, NPM1, PEMT, RUNX3, B4GALT1, PTPN6, ODC1, TESC, BECN1, KLF10, SPHK1, S100A11, DUSP22, RPS9, ROGDI, SMAD3, TPD52L2, FOXP1, POLD4, CDKN1A, C19ORF10, CORO1A, ATF3, HDAC1, HGS, PLAU, KLF4, SASH3, NAMPT, NBN, FOSL2, E2F4, MARCKSL1, TSG101, NAP1L1, BAP1, PTEN, WARS, NDUFS4, HSF1, CHST11, TICAM1, NRG1, FOSL1, RHOG, SERTAD1, ARHGEF2, PTGER2, UBE2A, LYN, SF1, MBD2, RPS4X, CD164, OSM, TNFRSF9, NCK2, CD86, ADRB2, BTG2, TNFSF13B, EREG, ADM, BTG1, GRN, BTG3, MDM2, DHPS, CXCL1, CSF3, CSF2, PML, RPS15A, ABI1, COMT, TRIB1, STAT6, CD9, PTK2B, BCL6, THBS1, QSOX1, TINF2, TCIRG1, PTPRC, IL6, IL8, JARID2, HCLS1, ANXA1, BIRC6, SPARC, STAT1, CAPN1,	

		SOD2, ID2, NME1-NME2, BAX, HBEGF, ATP5A1	
GO:0050870~positive regulation of T cell activation	1.09E-03	PTPRC, IL6, IKZF1, STAT5A, TNFSF14, BAD, IL15, PNP, ADA, CD74, SART1, CD47, CD83, NCK2, CORO1A, CD86, TNFSF13B, IL4R, RIPK2, IL1B, RARA, CD4, IL2RG, SASH3	2.43E-02
GO:0001819~positive regulation of cytokine production	1.15E-03	ATP6AP2, TLR2, PF4, TGFB1, SLC11A1, MYD88, TICAM1, PYCARD, FCER1G, IL1B, BCL3, RARA, THBS1, CASP1, IL1A, AKIRIN2, IL6, CARD8, SMAD3, SOD1, NLRP3, CD83, EREG, RIPK2, CLEC7A, SASH3, CD14	2.55E-02
GO:0007005~mitochondrion organization	1.20E-03	BID, OXA1L, TSPO, SPG7, YWHAZ, GRPEL1, TIMM17A, CLU, SNCA, TIMM17B, TIMM10, MPV17, PMAIP1, NDUFAF3, AIP, FIS1, NDUFS5, TYMP, TOMM7, NDUFS4, SH3GLB1, TOMM5, TIMM9, SYNJ2, HSPA4, HSP90AA1, SSBP1, POLG, TAZ, SMAD3, PIM2, SOD2, PPIF, BAX, TOMM20, TOMM22, IFI6	2.65E-02
GO:0019362~pyridine nucleotide metabolic process	1.25E-03	NAMPT, LDHB, KYNU, TALDO1, PGD, NADSYN1, IDH3B, KMO, NADK, PNP, TPI1, PGLS, IDH3G, MDH2, MDH1, NAPRT1	2.74E-02
GO:0045767~regulation of anti-apoptosis	1.28E-03	SIVA1, RTN4, IL6, RPS27L, CDKN1A, LRP1, ADM, BTG2, DUSP1, PTK2B, BAX, TNFAIP8, BRE, RARA, SLC9A1	2.78E-02
GO:0016567~protein ubiquitination	1.28E-03	TSG101, UBE2V1, CDC34, ANAPC11, STUB1, OS9, PRPF19, UBE2D3, UBE2D2, RNF167, NEDD4L, UBE2D1, FBXO9, RPS27A, TRIP12,	2.78E-02

		RNF144B, UBE2A, UBE2H, TMEM189, UBE2B, RBBP6, ATG3, UBE2N, NOSIP, VCP, UBC, MDM2, PAF1, UBB, UBA52, RNF40, UBE2E1, FBXO11, HDAC6	
GO:0046700~heterocycle catabolic process	1.33E-03	ATP5D, ATP5E, FHIT, KYNU, NUDT1, ATP5B, NUDT5, RHOQ, MYO9B, KMO, PDE4D, AMPD3, PNP, ADA, GCH1, BLVRA, HMOX2, DERA, RAB11A, CDA, ATP5O, DPYD, ATP5A1, NT5C	2.86E-02
GO:0002252~immune effector process	1.36E-03	NBN, YWHAZ, CLU, RSAD2, PRDX2, PRDX1, CD74, CTNNBL1, CFP, SLC11A1, MYD88, FCN1, TICAM1, FCER1G, BCL3, PTX3, ERCC1, ICAM1, PTPRC, IL6, LYN, RELA, SAMHD1, MYO1F, STXBP2, SLAMF7, NLRP3, PRKCD, FOXP1, TRAF3IP2, LAT, CD55, LAT2, TNFSF13B, LYST, BNIP3L	2.93E-02
GO:0030097~hemopoiesis	1.47E-03	XRCC5, STAT5A, IL15, TGFB1, IL10, CTNNB1, CDC42, CASP8, RHOH, CD3D, LYN, RELB, TAZ, MINK1, IFI16, MYH9, CD164, RPS19, PSEN1, RPS14, AHSP, VEGFA, CSF3, CSF2, GPR183, TIPARP, PML, CD74, ADA, TIMP1, TAL1, DOCK2, RASGRP4, BCL11A, BCL3, CD4, BCL6, RUNX1, ERCC1, EBP, KLF6, PTPRC, PLEK, IKZF1, HCLS1, ROGDI, FOXP1, SOD2, HDAC5, ID2, RPL22, JMJD6, BAX, IRF8, IRF1, HDAC9	3.14E-02
GO:0051094~positive regulation of developmental process	1.47E-03	XRCC5, PDLIM7, STAT5A, MORF4L2, XRCC6, NFKB1, TNFSF13, PNP, SART1, TGFB1, CTNNB1,	3.13E-02

		ACTR3, SMAP1, IL4R, ILK, RHOA, IL1B, RARA, NRG1, FNDC3B, IL1A, ARHGEF1, LYN, SOCS3, RELA, PLXNB2, JUNB, CD83, ADRB2, CD86, CD36, BTG1, F3, TGIF1, CAMK1, TGIF2, GNAS, CSF2, CLU, PF4, CD74, ADA, PRPF19, TNFRSF1A, TAL1, JUND, NUMB, IL2RG, RUNX1, THBS1, B4GALT1, PTPRC, TESC, IL6, IKZF1, KLF10, SPHK1, SMAD3, BAD, NME1-NME2, ID2, BAX, HDAC9, SASH3	
GO:0009164~nucleoside catabolic process	1.54E-03	FHIT, CDA, DERA, DPYD, PNP, ADA	3.25E-02
GO:0050865~regulation of cell activation	1.58E-03	ADORA2A, STAT5A, SNCA, TNFSF14, PRDX2, TNFSF13, IL15, PNP, SART1, CD74, TGFB1, IL10, ADA, STAT6, CD47, IL4R, PVRL2, TICAM1, IL1B, RARA, CD4, BCL6, IL2RG, THBS1, FCER1A, PTPRC, IL6, PLEK, IKZF1, STXBP2, TNFRSF14, BAD, SOD1, CLPTM1, LAT, NCK2, CD83, CORO1A, CDKN1A, CD86, TNFSF13B, PRAM1, RIPK2, SASH3	3.33E-02
GO:0070647~protein modification by small protein conjugation or removal	1.60E-03	USP7, ENY2, TSG101, UBE2V1, NEDD8, CDC34, ANAPC11, STUB1, OS9, RBX1, PRPF19, UBE2D3, UBE2D2, RNF167, NEDD4L, UBE2D1, FBXO9, RPS27A, TRIP12, RNF144B, UBE2A, UBE2F, UBE2H, TMEM189, ATG3, UBE2B, RBBP6, UBE2N, TAF10, NOSIP, RNF7, VCP, UBE2M, UBC, MDM2, PAF1, UBB, RNF40, UBA52, HDAC6, UBE2E1, FBXO11	3.36E-02
GO:0009967~positive	1.63E-03	HRAS, SLC44A2, VAPA,	3.39E-02

regulation of signal transduction		SHOC2, ZEB2, TGFB1, CTNNB1, CITED2, MYD88, TMEM9B, ILK, TICAM1, CASP8, RHOA, IL1B, RHOC, CASP1, BST2, LYN, RELA, PIM2, TMEM189, MBD2, FLNA, UBE2N, OSM, ADRB2, TNFRSF10B, EREG, ATP2C1, F3, VEGFA, RIPK2, EEF1D, CSF2, FKBP8, LITAF, PPM1A, UBE2V1, FKBP1A, ADA, SRC, TNFRSF1A, ECE1, SLC35B2, RASGRP4, SHISA5, RAC1, CD4, NENF, THBS1, FCER1A, PTPRC, SECTM1, CFLAR, IL6, HCLS1, LGALS1, TAOK3, DUSP22, BIRC2, LGALS9, PTPN11, TRAF3IP2, P2RX4, APOL3, TNFSF10, HDAC6	
GO:0051147~regulation of muscle cell differentiation	1.71E-03	MORF4L2, EZH2, HDAC5, HDAC3, HDAC1, EREG, BTG1, ILK, UBC, CAMK1, UBB, HDAC9, NRG1, RPS27A, UBA52	3.53E-02
GO:0045730~respiratory burst	1.71E-03	CYBA, SLC11A1, CD55, CYBB, NCF2, NCF1, PRDX2, CD52	3.52E-02
GO:0032680~regulation of tumor necrosis factor production	1.71E-03	TLR2, PF4, IL10, MYD88, HSF1, TICAM1, RIPK2, BCL3, FCER1G, RARA, CLEC7A, CD14, SASH3	3.51E-02
GO:0009060~aerobic respiration	1.73E-03	UQCRC2, OXA1L, UQCRC1, SUCLG1, IDH3B, SDHA, SDHB, IDH3G, UQCRH, SDHC, SURF1, MDH2, UQCRB, MDH1	3.54E-02
GO:0045621~positive regulation of lymphocyte differentiation	1.73E-03	PTPRC, IKZF1, STAT5A, BAD, PNP, CD74, SART1, ADA, CD83, CD86, IL4R, IL2RG, RARA, SASH3	3.54E-02
GO:0042542~response to hydrogen peroxide	1.73E-03	OLR1, RELA, ADRBK1, PRDX2, SOD1, STAT1, PRDX1, ADA, DDIT3, PARK7, SOD2, DUSP1, PTK2B, PRDX6, GPX4,	3.52E-02

		GPX3, PPP1R15B, FOSL1, HDAC6	
GO:0034341~response to interferon-gamma	1.80E-03	SLC11A1, KYNU, SP100, CXCL16, SNCA, CALCOCO2, GCH1	3.64E-02
GO:0022402~cell cycle process	1.80E-03	PRC1, TGFB1, SART1, CTNNB1, CUL3, MEI1, CDKN2D, TARDBP, ILK, RPS27A, ANAPC5, RAN, PIM1, GPR132, PIM2, SKP1, DCTN3, MYH9, PPP1CB, DDIT3, FOXN3, DCTN2, RAD1, PSMA2, PSMA1, REC8, PSMA6, SPAG5, PSMA5, PSMA4, CNTROB, PSMA3, MAD2L2, UBA52, NEDD9, ANAPC10, ANAPC11, CDC34, CALR, PSMA7, PSMB4, PSMB7, PSMB6, NIPBL, PSMB1, PSMB2, NPM1, CDC123, UBE2D1, LFNG, SSSCA1, SMAD3, PMF1, SUGT1, GAS7, HDAC3, CDKN1A, PSMC5, PSMC4, PSMC3, PSMC2, UBE2E1, MAD1L1, NBN, E2F4, TSG101, RHOU, PSMD1, PSMD2, PSMD4, PSMD6, PSMD7, PSMD8, PSMD9, ARHGEF2, CCNK, SF1, UBE2I, EML4, EIF4G2, PPM1G, CHMP1A, EREG, PSME1, CDK11A, PSME2, UBC, MDM2, UBB, MAPRE1, KPNA2, PPP1R15A, MAP3K11, PSMB10, HAUS4, PPP6C, HAUS2, PML, ZNF655, TCF7L2, PSMF1, PAFAH1B1, HBP1, THBS1, NFATC1, IL8, RPL24, ILF3, PSMD13, PSMD11, GSK3B, CKS2, MPHOSPH6, ARAP1, TXNL4A, DNM2	3.63E-02
GO:0033365~protein localization in organelle	1.82E-03	BID, SRP14, TSPO, YWHAZ, GRPEL1, PDIA3, XPO6, TIMM17A, ATP6AP1, TIMM17B,	3.64E-02

		TIMM10, PML, NFKBIA, SRP19, TGFB1, AIP, SLC11A1, TOMM7, SRPR, TOMM5, TIMM9, BCL3, BCL6, SRGN, SEC61A1, RBM22, RAN, NDUFA13, OPTN, COG3, SEC61B, RPL23, IPO5, TOMM20, NOP58, TOMM22, KPNA2, SEC61G	
GO:0030100~regulation of endocytosis	1.98E-03	RAB5C, ATP6AP1, AP2S1, SNX17, SNCA, RUFY1, ARF6, PPT1, CD63, CALR, PTEN, TGFB1, NR1H2, SLC11A1, RAC1, NEDD4L, CLEC7A, BIN1, PTX3, EHD4	3.94E-02
GO:0009617~response to bacterium	2.15E-03	CCL2, PTGS2, FGR, SNCA, TLR2, NFKBIA, PRDX2, COMT, IL10, GCH1, TRIB1, B2M, CFP, TNFRSF1A, FOS, SLC11A1, MYD88, HIST1H2BK, HSF1, CCL20, TICAM1, BCL3, IL1B, LTF, SERPINA1, IRG1, AKIRIN2, IRAK1, IL6, PLD1, SOCS3, LY96, CAMP, RELA, HCK, GNLY, LYZ, MYO1F, STAT1, S100A12, PPBP, ADM, LYST, DEFA3, RIPK2, MGST1, CD14	4.26E-02
GO:0009143~nucleoside triphosphate catabolic process	2.16E-03	ATP5D, ATP5E, NUDT1, ATP5B, RHOQ, RAB11A, ATP5O, MYO9B, ATP5A1, ADA, GCH1	4.27E-02
GO:0043487~regulation of RNA stability	2.16E-03	ZFP36, ZFP36L1, SLC11A1, DHX9, TNFRSF1B, VEGFA, HNRNPD, MAPKAPK2, PABPC1, HNRNPU, YBX1	4.27E-02
GO:0030433~ER-associated protein catabolic process	2.16E-03	DERL2, HSP90B1, SEC61B, DERL1, KIAA0368, VCP, UBC, UBB, UBA52, RPS27A, OS9	4.27E-02
GO:0046634~regulation of alpha-beta T cell activation	2.32E-03	PTPRC, IKZF1, ADORA2A, TNFRSF14, PNP, ADA, CD83, CD86, IL4R, RIPK2,	4.56E-02

		BCL6, IL2RG, RARA, SASH3	
GO:0051153~regulation of striated muscle cell differentiation	2.35E-03	MORF4L2, EZH2, HDAC5, HDAC3, HDAC1, BTG1, ILK, UBC, UBB, HDAC9, NRG1, RPS27A, UBA52	4.59E-02
GO:0033157~regulation of intracellular protein transport	2.36E-03	NFKBIE, NFKBIB, NFKBIA, SMAD3, TNFSF14, NLRP3, CDK5, PRDX1, FLNA, TGFB1, IL10, PTPN11, GSK3B, RHOA, BCL3, IL1B, PRKACA, LCP1	4.59E-02
GO:0070555~response to interleukin-1	2.40E-03	IRAK2, IRAK1, AES, MYD88, RELA, SNCA, RIPK2, TAF9, SRC	4.64E-02
GO:0002221~pattern recognition receptor signaling pathway	2.40E-03	IRAK2, IRAK1, MYD88, RELA, TICAM1, TLR2, NFKBIA, RIPK2, CLEC7A	4.64E-02
GO:0007243~protein kinase cascade	2.43E-03	STAT5A, PRKAG2, TLR2, TNFSF14, RPS6KB2, PRDX2, PRKCSH, IL10, CXCR4, TICAM1, IL1B, PRKACA, PAK1, RAPGEF2, TNIP2, ZFP36, IRAK2, PPP2R1A, IRAK1, DBNL, C5AR1, SOCS3, MADD, LY96, MINK1, PKN1, CDK5, TANK, OSM, TYK2, MAP4K3, SPAG9, MAP4K4, IFNAR2, CRKL, TNFRSF10B, PSEN1, ITGB1BP1, GADD45B, MAP3K11, CCL2, NMI, GNAI2, GRB2, NFKBIB, STK17B, MKNK2, DUSP10, FPR1, MAP4K1, NFKBIA, MAPKAPK2, SRC, CD74, AZI2, TRIB1, STAT4, PTK2B, PPP2CA, BCL3, THBS1, FCER1A, PTPRC, PLEK, MAP2K3, TAOK3, DUSP22, STAT1, SOD1, STAT3, GPS2, PTPN11, DUSP2, PRLR, RPS6KA1, FYN, IKBKB, SLC9A1, SMPD2, DUSP6	4.68E-02
GO:0022415~viral reproductive process	2.44E-03	XRCC5, ICAM1, CCL2, DERL1, TSG101, XRCC6, SMAD3, BANF1, HMGA1,	4.67E-02

		USF2, TGFB1, CCR5, CXCR4, PPIA, IRF7, PVRL2, THOC7, CD4, THOC2, SUPT5H	
GO:0051223~regulation of protein transport	2.56E-03	ADORA2A, NFKBIE, NFKBIB, NFKBIA, TNFSF14, PRDX1, IL10, TGFB1, PYCARD, EXOC4, RHOA, IL1B, RAB11A, BCL3, PRKACA, CASP1, SRGN, IL1A, DNAJC1, SERGEF, IL6, CARD8, YWHAB, SMAD3, NLRP3, CDK5, FLNA, PTPN11, KCNN4, GSK3B, LCP1	4.88E-02

Biological Process (BP_FAT) associated with genes having decreased expression in **HNP_NeoHep** with respect to HNP_Monocyte

Term	PValue	Genes	Benjamini
GO:0006955~immune response	4.94E-18	KYNU, NBN, AQP9, IL16, IL19, TLR2, TNFSF14, NFKB2, IL15, PNP, CXCL10, B2M, CFP, TMEM173, LILRA1, CLEC4E, LILRA4, IL1RAP, CLEC4A, LTF, IL1B, CLEC4D, NFIL3, IL1A, GBP5, BST1, NCF1, HLA-A, HLA-C, GEM, NLRP3, WAS, CTSW, OSM, CD83, CCR7, PPBP, CCR5, TREM1, GBP4, GBP2, GBP1, HLA-DRA, LCP2, CSF3, CXCL1, CSF2, GPR183, IFIH1, YWHAZ, CCL2, IFITM2, CXCL3, CXCL2, RSAD2, OAS1, PF4, PF4V1, CLEC10A, ADA, CCL7, GCH1, IL23A, CCL20, XBP1, POU2F2, FCN1, BCL3, THBS1, PTX3, IL6, CEBPB, OLR1, IL8, IL1RN, IGJ, CD300E, CD1C, FOXP1, TNFSF8, TRAF3IP2, CORO1A, CD55, TNFSF10, OASL, RGS1, FCAR, CLEC7A, RNF19B, TREML1	1.42E-14
GO:0006414~translational elongation	2.02E-14	EEF1B2, RPL15, RPS15A, RPL37, RPS25, RPL7, RPL31, RPL34, RPL9, RPL3, RPL5, RPS20, RPS21, RPS23, RPS24, RPL35A, RPL26, RPL24, RPS6, RPS4X, SELT, RPS7, HNRNPH2, RPS16, RPL22, RPS14, RPL21, RPS13, RPS10	2.90E-11
GO:0006952~defense response	2.18E-13	KYNU, S100A8, S100A9, TLR2, IL15, CXCL10, CD48, CFP, TMEM173, LILRA1, CD44, IL1RAP, LTF, IL1B, FOSL1, TNIP1, IL1A, IRAK2, NFKBIZ, NCF1, GNLY, CHST2, HLA-C, NLRP3, WAS, NLRP1, CD83, INHBA, TNFAIP6, CCR7, PPBP, CCR5, HIST2H2BE, DEFA4, MNDA, DEFA3, RIPK2,	2.08E-10

		KDM6B, CLEC1B, HLA-DRA, TPST1, CXCL1, IFIH1, YWHAZ, CCL2, NMI, ADORA2A, CXCL3, CXCL2, RSAD2, CCL7, GCH1, IL23A, MEFV, CCL20, BCL3, PTX3, THBS1, B4GALT1, IL6, CEBPB, OLR1, AIMP1, IL8, MAP2K3, IL1RN, ANXA1, COTL1, S100A12, CORO1A, CD55, IRF7, MPO, CLEC7A, TREML1	
GO:0042981~regulation of apoptosis	2.73E-11	MEF2C, PTGS2, IL19, TLR2, TNFSF14, PMAIP1, PTEN, CRADD, CASP5, CUL5, CASP9, CD44, CDKN2D, TPT1, IL1B, NRG1, CASP1, MYC, FOSL1, IL1A, CD3E, SOCS3, BCL2A1, PIM3, PIM2, NLRP3, NLRP1, BCL2L11, SERPINB9, INHBA, ADRB2, BTG2, CARD16, BTG1, TNFAIP8, CARD17, SERPINB2, RIPK2, TNFAIP3, ALOX12, TRAF1, BID, CSF2, HMGB1, IFIH1, ING3, YWHAZ, CCL2, MCL1, ADORA2A, STK17B, NFKBIA, PF4, ADA, GCH1, PLEKHG2, ERCC6, RB1CC1, SOS2, BCL3, PPP3CC, HSPE1, TAF9, THBS1, PHLDA1, B4GALT1, IL6, CEBPB, KLF10, ANXA1, NR4A2, CIDEB, NR4A1, BIRC3, RPS6, SOD2, TNFSF8, PPIF, TNFSF10, RNF7, PRLR, DUSP1, MPO, DNAJB6	1.96E-08
GO:0043067~regulation of programmed cell death	4.45E-11	MEF2C, PTGS2, IL19, TLR2, TNFSF14, PMAIP1, PTEN, CRADD, CASP5, CUL5, CASP9, CD44, CDKN2D, TPT1, IL1B, NRG1, CASP1, MYC, FOSL1, IL1A, CD3E, SOCS3, BCL2A1, PIM3, PIM2, NLRP3, NLRP1, BCL2L11, SERPINB9, INHBA, ADRB2, BTG2, CARD16, BTG1, TNFAIP8,	2.56E-08

		CARD17, SERPINB2, RIPK2, TNFAIP3, ALOX12, TRAF1, BID, CSF2, HMGB1, IFIH1, ING3, YWHAZ, CCL2, MCL1, ADORA2A, STK17B, NFKBIA, PF4, ADA, GCH1, PLEKHG2, ERCC6, RB1CC1, SOS2, BCL3, PPP3CC, HSPE1, TAF9, THBS1, PHLDA1, B4GALT1, IL6, CEBPB, KLF10, ANXA1, NR4A2, CIDEB, NR4A1, BIRC3, RPS6, SOD2, TNFSF8, PPIF, TNFSF10, RNF7, PRLR, DUSP1, MPO, DNAJB6	
GO:0010941~regulation of cell death	5.41E-11	MEF2C, PTGS2, IL19, TLR2, TNFSF14, PMAIP1, PTEN, CRADD, CASP5, CUL5, CASP9, CD44, CDKN2D, TPT1, IL1B, NRG1, CASP1, MYC, FOSL1, IL1A, CD3E, SOCS3, BCL2A1, PIM3, PIM2, NLRP3, NLRP1, BCL2L11, SERPINB9, INHBA, ADRB2, BTG2, CARD16, BTG1, TNFAIP8, CARD17, SERPINB2, RIPK2, TNFAIP3, ALOX12, TRAF1, BID, CSF2, HMGB1, IFIH1, ING3, YWHAZ, CCL2, MCL1, ADORA2A, STK17B, NFKBIA, PF4, ADA, GCH1, PLEKHG2, ERCC6, RB1CC1, SOS2, BCL3, PPP3CC, HSPE1, TAF9, THBS1, PHLDA1, B4GALT1, IL6, CEBPB, KLF10, ANXA1, NR4A2, CIDEB, NR4A1, BIRC3, RPS6, SOD2, TNFSF8, PPIF, TNFSF10, RNF7, PRLR, DUSP1, MPO, DNAJB6	2.59E-08
GO:0006954~inflammatory response	1.56E-10	TPST1, CXCL1, YWHAZ, CCL2, NMI, S100A8, ADORA2A, CXCL3, CXCL2, S100A9, TLR2, IL15, CCL7, CXCL10, CFP, IL23A, CD44, CCL20, MEFV, IL1RAP, IL1B, PTX3, THBS1, IL1A,	6.40E-08

		B4GALT1, IRAK2, NFKBIZ, IL6, CEBPB, AIMP1, IL8, OLR1, MAP2K3, IL1RN, ANXA1, CHST2, NLRP3, S100A12, TNFAIP6, CD55, CCR7, CCR5, IRF7, RIPK2, CLEC7A, KDM6B	
GO:0006412~translation	2.90E-10	MRPS36, NACA, EEF1B2, RPL15, RPS15A, RPL37, RPL22L1, RPS25, CYLD, RPL7, RPL31, EIF3H, RPL9, RPL34, EIF3F, EIF1AY, RPL3, RPL5, RPS20, RSL24D1, RPS21, MRPL32, TNIP1, EIF3M, RPS23, RPL36AL, RPS24, RPL35A, AIMP1, RPL26, MTRF1L, EIF1B, RPL24, RPS6, RPS4X, GTF2B, SELT, RPS7, EIF4E, HNRNPH2, RPS16, RPL22, RPS14, RPL21, RPS13, RPS10	1.04E-07
GO:0009611~response to wounding	5.71E-10	S100A8, F13A1, S100A9, TLR2, IL15, CXCL10, GP9, LUZP6, CFP, DYSF, CD44, IL1RAP, IL1B, NRG1, IL1A, IRAK2, NFKBIZ, CHST2, NLRP3, WAS, PLAUR, TNFAIP6, CCR7, THBD, CCR5, EREG, SERPINB2, RIPK2, KDM6B, TPST1, CXCL1, YWHAZ, NMI, CCL2, ADORA2A, CXCL3, CXCL2, PF4, CCL7, IL23A, CCL20, MEFV, PTX3, THBS1, B4GALT1, KLF6, IL6, CEBPB, AIMP1, IL8, OLR1, MAP2K3, IL1RN, ANXA1, S100A12, SOD2, CD55, IRF7, HBEGF, CLEC7A, TREML1	1.82E-07
GO:0008380~RNA splicing	1.01E-09	NCBP1, PPP4R2, STRAP, POLR2K, TRA2B, TRA2A, SNRPB2, SNRPD1, WTAP, HNRNPL, NONO, HNRNPA3, HNRNPK, PCBP1, TARDBP, DHX15, HNRNPD, U2AF1, QKI, HNRNPC, LSM1, BCAS2, SNRPA1, CSTF3, SF3B14,	2.89E-07

		MAGOH, HNRNPA2B1, PRPF3, DDX5, HNRNPU, EIF4A3, PCF11, HNRNPH3, HNRNPH2, ZRANB2, SYF2, PHF5A, RBM39, HNRNPH1, THOC2, SNRPG	
GO:0006397~mRNA processing	1.16E-08	NCBP1, PPP4R2, STRAP, POLR2K, TRA2B, TRA2A, SNRPB2, SNRPD1, WTAP, HNRNPL, NONO, HNRNPA3, HNRNPK, PCBP1, TARDBP, DHX15, HNRNPD, U2AF1, QKI, HNRNPC, LSM1, ZFP36, BCAS2, SNRPA1, CSTF3, SF3B14, MAGOH, HNRNPA2B1, PRPF3, DDX5, HNRNPU, EIF4A3, PCF11, HNRNPH3, HNRNPH2, ZRANB2, SYF2, PHF5A, RBM39, HNRNPH1, THOC2, SNRPG	3.02E-06
GO:0006396~RNA processing	3.20E-08	NAF1, NCBP1, GAR1, SNRPD1, WTAP, NONO, TARDBP, PCBP1, U2AF1, QKI, LSM1, ZFP36, RPL35A, SNRPA1, EXOSC8, SF3B14, MAGOH, HNRNPA2B1, PRPF3, HNRNPU, EIF4A3, PCF11, RPS16, LARP7, RPS14, RBM39, THOC2, SNRPG, PPP4R2, POLR2K, STRAP, TRA2B, TRA2A, SNRPB2, HNRNPL, HNRNPA3, ZFC3H1, HNRNPK, RPL7, DHX15, HNRNPD, NPM3, RPL5, HNRNPC, NSA2, RPS24, BCAS2, CSTF3, RPL26, RPS6, DDX5, RPS7, HNRNPH3, HNRNPH2, ZRANB2, SYF2, PHF5A, HNRNPH1	7.66E-06
GO:0006935~chemotaxis	4.87E-08	CXCL1, CCL2, IL16, CXCL3, S100A9, CXCL2, FPR1, PF4, CCL7, CXCL10, CCRL2, TYMP, CCL20, IL1B, FOSL1, PLP2, IL6, RNASE2, CMTM2, IL8, AIMP1, PLAUR, CORO1A, CCR7,	1.07E-05

		CCR5, PPBP, CMTM5	
GO:0042330~taxis	4.87E-08	CXCL1, CCL2, IL16, CXCL3, S100A9, CXCL2, FPR1, PF4, CCL7, CXCL10, CCRL2, TYMP, CCL20, IL1B, FOSL1, PLP2, IL6, RNASE2, CMTM2, IL8, AIMP1, PLAUR, CORO1A, CCR7, CCR5, PPBP, CMTM5	1.07E-05
GO:0000375~RNA splicing, via transesterification reactions	7.88E-08	NCBP1, POLR2K, TRA2B, SNRPB2, TRA2A, SNRPD1, HNRNPL, HNRNPA3, HNRNPK, PCBP1, U2AF1, HNRNPD, HNRNPC, SNRPA1, CSTF3, SF3B14, MAGOH, HNRNPA2B1, PRPF3, HNRNPU, PCF11, HNRNPH3, HNRNPH2, PHF5A, HNRNPH1, SNRPG	1.61E-05
GO:0000398~nuclear mRNA splicing, via spliceosome	7.88E-08	NCBP1, POLR2K, TRA2B, SNRPB2, TRA2A, SNRPD1, HNRNPL, HNRNPA3, HNRNPK, PCBP1, U2AF1, HNRNPD, HNRNPC, SNRPA1, CSTF3, SF3B14, MAGOH, HNRNPA2B1, PRPF3, HNRNPU, PCF11, HNRNPH3, HNRNPH2, PHF5A, HNRNPH1, SNRPG	1.61E-05
GO:0000377~RNA splicing, via transesterification reactions with bulged adenosine as nucleophile	7.88E-08	NCBP1, POLR2K, TRA2B, SNRPB2, TRA2A, SNRPD1, HNRNPL, HNRNPA3, HNRNPK, PCBP1, U2AF1, HNRNPD, HNRNPC, SNRPA1, CSTF3, SF3B14, MAGOH, HNRNPA2B1, PRPF3, HNRNPU, PCF11, HNRNPH3, HNRNPH2, PHF5A, HNRNPH1, SNRPG	1.61E-05
GO:0001775~cell activation	1.45E-07	CSF2, GPR183, NBN, YWHAZ, ADORA2A, TLR2, TNFSF14, PF4, NFKB2, IL15, ADA, HSH2D, CTNNB1, CD48, IL23A, CD93, BCL3, RHOH, GAPT, EGR1, KLF6, IL6, CD3D, IL8, IKZF1, CRIP3, CD3E, SLAMF1, WAS, FOXP1, PRLR, RPL22, IRF1, RIPK2, CLEC7A, TREML1, LCP2	2.77E-05

GO:0006916~anti-apoptosis	2.09E-07	MEF2C, CSF2, HMGB1, YWHAZ, CCL2, MCL1, NFKBIA, CDKN2D, TPT1, IL1B, THBS1, NRG1, MYC, IL1A, CEBPB, SOCS3, BCL2A1, ANXA1, PIM2, BIRC3, SOD2, SERPINB9, RNF7, PRLR, TNFAIP8, SERPINB2, MPO, RIPK2, TNFAIP3, ALOX12	3.75E-05
GO:0016071~mRNA metabolic process	2.25E-07	NCBP1, PPP4R2, STRAP, POLR2K, TRA2B, TRA2A, SNRPB2, SNRPD1, WTAP, ZFP36L1, HNRNPL, NONO, HNRNPA3, HNRNPK, PCBP1, TARDBP, DHX15, HNRNPD, U2AF1, QKI, HNRNPC, LSM1, ZFP36, BCAS2, SNRPA1, CSTF3, SF3B14, MAGOH, HNRNPA2B1, PRPF3, DDX5, HNRNPU, EIF4A3, PCF11, HNRNPH3, HNRNPH2, ZRANB2, SYF2, PHF5A, RBM39, HNRNPH1, THOC2, SNRPG	3.81E-05
GO:0043066~negative regulation of apoptosis	4.95E-07	MEF2C, CSF2, HMGB1, YWHAZ, CCL2, MCL1, ADORA2A, NFKBIA, PF4, PTEN, ADA, CDKN2D, RB1CC1, TPT1, BCL3, IL1B, TAF9, NRG1, THBS1, MYC, IL1A, IL6, CEBPB, SOCS3, BCL2A1, ANXA1, NR4A2, PIM3, PIM2, BIRC3, SOD2, SERPINB9, RNF7, PRLR, BTG2, TNFAIP8, SERPINB2, MPO, RIPK2, TNFAIP3, ALOX12	7.89E-05
GO:0045321~leukocyte activation	6.76E-07	CSF2, GPR183, NBN, YWHAZ, TLR2, TNFSF14, IL15, ADA, HSH2D, CTNNB1, CD48, IL23A, CD93, BCL3, RHOH, GAPT, EGR1, KLF6, CD3D, IL8, IKZF1, CRIP3, CD3E, SLAMF1, WAS, FOXP1, PRLR, RPL22, IRF1, RIPK2, CLEC7A, LCP2	1.02E-04
GO:0043069~negative	7.09E-07	MEF2C, CSF2, HMGB1,	1.02E-04

regulation of programmed cell death		YWHAZ, CCL2, MCL1, ADORA2A, NFKBIA, PF4, PTEN, ADA, CDKN2D, RB1CC1, TPT1, BCL3, IL1B, TAF9, NRG1, THBS1, MYC, IL1A, IL6, CEBPB, SOCS3, BCL2A1, ANXA1, NR4A2, PIM3, PIM2, BIRC3, SOD2, SERPINB9, RNF7, PRLR, BTG2, TNFAIP8, SERPINB2, MPO, RIPK2, TNFAIP3, ALOX12	
GO:0060548~negative regulation of cell death	7.69E-07	MEF2C, CSF2, HMGB1, YWHAZ, CCL2, MCL1, ADORA2A, NFKBIA, PF4, PTEN, ADA, CDKN2D, RB1CC1, TPT1, BCL3, IL1B, TAF9, NRG1, THBS1, MYC, IL1A, IL6, CEBPB, SOCS3, BCL2A1, ANXA1, NR4A2, PIM3, PIM2, BIRC3, SOD2, SERPINB9, RNF7, PRLR, BTG2, TNFAIP8, SERPINB2, MPO, RIPK2, TNFAIP3, ALOX12	1.05E-04
GO:0010608~posttranscriptional regulation of gene expression	1.15E-06	NCBP1, CPEB2, PTEN, LUZP6, ZFP36L1, ASGR2, EIF3H, HNRNPD, QKI, BCL3, THBS1, ZFP36, IL6, GTPBP4, CCDC88C, PAIP2, MAGOH, EIF1B, RPS4X, SELT, UBE2B, HNRNPU, COG3, EIF4A3, EIF4E, AHSP, NGDN, PPP1R15B, PPP1R15A	1.50E-04
GO:0012501~programmed cell death	2.81E-06	MEF2C, IL19, PMAIP1, PTEN, CRADD, CTNNB1, CASP5, TMEM173, CASP9, CDKN2D, IL1B, CASP1, MYC, IL1A, BCL2A1, PIM2, GZMH, NLRP3, NLRP1, BCL2L11, OSM, TNFAIP8, RIPK2, TNFAIP3, GADD45B, PPP1R15A, TRAF1, BID, MCL1, ADORA2A, STK17B, NFKBIA, GLRX2, TSC22D3, PLEKHG2, SOS2, PPP3CC, ZC3H12A, HSPE1, XAF1, THBS1, SRGN, PHLDA2, PHLDA1, RNF144B, IL6,	3.51E-04

		AIMP1, SRA1, RYBP, CIDEB, BIRC3, SOD2, FXR1, TRAF3IP2, TNFSF10, CSRNP1, DRAM1	
GO:0006915~apoptosis	3.82E-06	MEF2C, IL19, PMAIP1, PTEN, CRADD, CTNNB1, CASP5, TMEM173, CASP9, IL1B, CASP1, MYC, IL1A, BCL2A1, PIM2, GZMH, NLRP3, NLRP1, BCL2L11, OSM, TNFAIP8, RIPK2, TNFAIP3, GADD45B, PPP1R15A, TRAF1, BID, MCL1, ADORA2A, STK17B, NFKBIA, GLRX2, TSC22D3, PLEKHG2, SOS2, PPP3CC, ZC3H12A, HSPE1, XAF1, THBS1, SRGN, PHLDA2, PHLDA1, RNF144B, IL6, AIMP1, SRA1, RYBP, CIDEB, BIRC3, SOD2, FXR1, TRAF3IP2, TNFSF10, CSRNP1, DRAM1	4.56E-04
GO:0042110~T cell activation	9.54E-06	EGR1, CRIP3, CD3D, IKZF1, CD3E, TNFSF14, IL15, WAS, ADA, CTNNB1, HSH2D, CD48, IL23A, PRLR, RPL22, IRF1, RIPK2, BCL3, CLEC7A, RHOH	1.09E-03
GO:0046649~lymphocyte activation	1.14E-05	GPR183, NBN, TNFSF14, IL15, ADA, CTNNB1, HSH2D, CD48, IL23A, BCL3, RHOH, GAPT, EGR1, KLF6, IKZF1, CRIP3, CD3D, CD3E, SLAMF1, WAS, FOXP1, PRLR, RPL22, IRF1, RIPK2, CLEC7A	1.25E-03
GO:0009615~response to virus	1.77E-05	IFIH1, IL6, ZC3HAV1, RSAD2, RPS15A, IFI44, NLRP3, ISG20, LCN2, IRAK3, IL23A, ISG15, IRF7, DEFA3, BCL3, FOSL1, IFNGR2, IFNGR1	1.88E-03
GO:0042254~ribosome biogenesis	2.21E-05	NAF1, RPL35A, GTPBP4, EXOSC8, GAR1, RPL26, RPL24, RPS6, RPS7, EIF4A3, RPL7, RPS16, RPS14, NPM3, RPL5, RSL24D1, GNL2, NSA2, RPS24	2.26E-03
GO:0043065~positive	2.52E-05	BID, ING3, PTGS2,	2.50E-03

regulation of apoptosis		ADORA2A, IL19, STK17B, TLR2, TNFSF14, PMAIP1, PTEN, CRADD, GCH1, CUL5, PLEKHG2, ERCC6, CD44, CASP9, SOS2, IL1B, PPP3CC, BCL3, CASP1, MYC, FOSL1, PHLDA1, B4GALT1, CEBPB, CD3E, KLF10, CIDEB, NR4A1, RPS6, NLRP3, NLRP1, BCL2L11, TNFSF8, INHBA, ADRB2, TNFSF10, RNF7, DUSP1, RIPK2	
GO:0043068~positive regulation of programmed cell death	2.95E-05	BID, ING3, PTGS2, ADORA2A, IL19, STK17B, TLR2, TNFSF14, PMAIP1, PTEN, CRADD, GCH1, CUL5, PLEKHG2, ERCC6, CD44, CASP9, SOS2, IL1B, PPP3CC, BCL3, CASP1, MYC, FOSL1, PHLDA1, B4GALT1, CEBPB, CD3E, KLF10, CIDEB, NR4A1, RPS6, NLRP3, NLRP1, BCL2L11, TNFSF8, INHBA, ADRB2, TNFSF10, RNF7, DUSP1, RIPK2	2.82E-03
GO:0010942~positive regulation of cell death	3.30E-05	BID, ING3, PTGS2, ADORA2A, IL19, STK17B, TLR2, TNFSF14, PMAIP1, PTEN, CRADD, GCH1, CUL5, PLEKHG2, ERCC6, CD44, CASP9, SOS2, IL1B, PPP3CC, BCL3, CASP1, MYC, FOSL1, PHLDA1, B4GALT1, CEBPB, CD3E, KLF10, CIDEB, NR4A1, RPS6, NLRP3, NLRP1, BCL2L11, TNFSF8, INHBA, ADRB2, TNFSF10, RNF7, DUSP1, RIPK2	3.05E-03
GO:0016265~death	5.20E-05	MEF2C, IL19, PMAIP1, PTEN, CRADD, CTNNB1, CASP5, TMEM173, CASP9, CDKN2D, TARDBP, IL1B, CASP1, MYC, IL1A, BCL2A1, PIM2, GZMH, NLRP3, NLRP1, BCL2L11, OSM, TNFAIP8, RIPK2, GADD45B, TNFAIP3,	4.65E-03

		PPP1R15A, TRAF1, BID, MCL1, ADORA2A, STK17B, NFKBIA, GLRX2, TSC22D3, PLEKHG2, SOS2, PPP3CC, ZC3H12A, HSPE1, XAF1, THBS1, SRGN, PHLDA2, PHLDA1, RNF144B, IL6, AIMP1, OLR1, SRA1, RYBP, CIDEB, NR4A2, BIRC3, FXR1, SOD2, TRAF3IP2, TNFSF10, CSRNP1, DRAM1	
GO:0009617~response to bacterium	5.86E-05	IL6, CCL2, PTGS2, SOCS3, GNLY, TLR2, NFKBIA, S100A12, TRIB1, B2M, GCH1, CFP, IRAK3, THBD, PPBP, CCL20, HIST2H2BE, DEFA4, DEFA3, LTF, RIPK2, BCL3, IL1B, IRG1	5.08E-03
GO:0048534~hemopoietic or lymphoid organ development	6.13E-05	CSF3, CSF2, GPR183, TIPARP, IL15, NFKB2, ADA, CTNNB1, TAL1, RASGRP4, BCL3, RUNX1, RHOH, EGR1, KLF6, CD3D, IKZF1, JARID2, CD3E, ROGDI, FOXP1, BCL2L11, SOD2, INHBA, ID2, RPL22, RPS14, AHSP, IRF1	5.16E-03
GO:0007626~locomotory behavior	6.24E-05	CXCL1, CCL2, IL16, ADORA2A, CXCL3, S100A9, CXCL2, FPR1, PF4, CCL7, CXCL10, CCRL2, TYMP, CCL20, IL1B, FOSL1, PLP2, IL6, RNASE2, CMTM2, AIMP1, IL8, NR4A2, SOD2, PLAUR, CORO1A, CCR7, PPBP, CCR5, CMTM5	5.10E-03
GO:0002520~immune system development	7.11E-05	CSF3, CSF2, GPR183, NBN, TIPARP, NFKB2, IL15, ADA, CTNNB1, TAL1, RASGRP4, BCL3, RUNX1, RHOH, EGR1, KLF6, CD3D, IKZF1, JARID2, CD3E, ROGDI, FOXP1, BCL2L11, SOD2, INHBA, ID2, RPL22, RPS14, AHSP, IRF1	5.65E-03
GO:0008219~cell death	8.14E-05	MEF2C, IL19, PMAIP1, PTEN, CRADD, CTNNB1, CASP5, TMEM173, CASP9, CDKN2D, TARDBP, IL1B, CASP1, MYC, IL1A,	6.29E-03

		BCL2A1, PIM2, GZMH, NLRP3, NLRP1, BCL2L11, OSM, TNFAIP8, RIPK2, GADD45B, TNFAIP3, PPP1R15A, TRAF1, BID, MCL1, ADORA2A, STK17B, NFKBIA, GLRX2, TSC22D3, PLEKHG2, SOS2, PPP3CC, ZC3H12A, HSPE1, XAF1, THBS1, SRGN, PHLDA2, PHLDA1, RNF144B, IL6, AIMP1, OLR1, SRA1, RYBP, CIDEB, BIRC3, FXR1, SOD2, TRAF3IP2, TNFSF10, CSRNP1, DRAM1	
GO:0010033~response to organic substance	8.77E-05	KYNU, AQP9, LDLR, PTGS2, IGFBP7, TLR2, PMAIP1, UQCRFS1, PTEN, B2M, CTNNB1, CD48, CD44, IL1B, RARA, GNG2, CASP1, FOSL1, MYC, IRAK2, EGR1, SOCS3, UBE2B, JUNB, CD83, RETN, THBD, BTG2, RIPK2, PPP1R15A, HMGB2, CCL2, MCL1, ADORA2A, NFKBIA, GNG11, GCH1, GLRX2, TRIB1, IRAK3, DNAJA1, TAF9, HSPE1, THBS1, IRG1, IL6, PTPN2, SELL, KLF10, IL1RN, NR4A2, NR4A3, PTPN11, CORO1A, ID2, DUSP1, ADCY9, CLEC7A, DNAJB6	6.60E-03
GO:0009620~response to fungus	1.01E-04	GNLY, DEFA4, DEFA3, TLR2, CLEC7A, COTL1, PTX3, S100A12	7.37E-03
GO:0006417~regulation of translation	1.05E-04	NCBP1, IL6, CPEB2, PAIP2, MAGOH, EIF1B, RPS4X, SELT, LUZP6, ZFP36L1, EIF4A3, EIF4E, EIF3H, QKI, NGDN, BCL3, PPP1R15B, THBS1, PPP1R15A	7.53E-03
GO:0006364~rRNA processing	1.26E-04	NAF1, RPL35A, EXOSC8, GAR1, RPL26, RPS6, RPS7, EIF4A3, RPL7, RPS16, RPS14, NPM3, RPL5, NSA2, RPS24	8.82E-03
GO:0022613~ribonucleoprotein complex biogenesis	1.64E-04	RPL35A, NCBP1, NAF1, EXOSC8, GTPBP4, GAR1, RPL26, SNRPD1, RPL24,	1.12E-02

		RPS6, RPS7, EIF4A3, RPL7, RPS16, RPS14, NPM3, RPL5, RSL24D1, GNL2, NSA2, RPS24, SNRPG	
GO:0001817~regulation of cytokine production	1.78E-04	FCER1A, IL6, CEBPB, CD3E, MAP2K3, TLR2, PF4, NLRP3, CD83, INHBA, IRAK3, EREG, IRF1, RIPK2, BCL3, IL1B, RARA, CLEC7A, THBS1, CASP1, IL1A, SRGN	1.18E-02
GO:0030097~hemopoiesis	1.94E-04	CSF3, CSF2, GPR183, TIPARP, IL15, ADA, CTNNB1, TAL1, RASGRP4, BCL3, RUNX1, RHOH, EGR1, KLF6, IKZF1, CD3D, CD3E, ROGDI, FOXP1, SOD2, INHBA, ID2, RPL22, RPS14, AHSP, IRF1	1.26E-02
GO:0016072~rRNA metabolic process	2.01E-04	NAF1, RPL35A, EXOSC8, GAR1, RPL26, RPS6, RPS7, EIF4A3, RPL7, RPS16, RPS14, NPM3, RPL5, NSA2, RPS24	1.27E-02
GO:0002684~positive regulation of immune system process	2.22E-04	TLR2, TNFSF14, NFKBIA, IL15, PNP, ADA, B2M, CFP, CD47, IRAK3, IL1B, RARA, THBS1, IRAK2, FCER1A, IL6, IKZF1, CD3E, FOXP1, CD83, CD55, CORO1A, EREG, RIPK2, CLEC7A, HLA-DRA	1.37E-02
GO:0002237~response to molecule of bacterial origin	2.34E-04	IL6, CCL2, PTGS2, SOCS3, TLR2, NFKBIA, GCH1, B2M, TRIB1, IRAK3, THBD, RIPK2, IL1B, IRG1	1.42E-02
GO:0050870~positive regulation of T cell activation	2.66E-04	IL6, IKZF1, CD3E, TNFSF14, IL15, PNP, ADA, CD83, CD47, CORO1A, RIPK2, IL1B, RARA	1.58E-02
GO:0042127~regulation of cell proliferation	2.83E-04	NBN, PTGS2, MARCKSL1, IGFBP7, FGFRL1, NAP1L1, IL15, PTEN, PNP, CXCL10, CTNNB1, CD47, CUL5, CDKN2D, IL1B, NRG1, FOSL1, MYC, IL1A, GTPBP4, UBE2A, PTGER2, CD3E, RPS4X, MBD2, OSM, ADRB2, BTG2, EREG, BTG1, TGIF1, RIPK2, ALOX12,	1.64E-02

		CSF3, CXCL1, CSF2, CCL2, MVD, IFITM1, ADORA2A, RPS15A, NFKBIA, ADA, TRIB1, THBS1, B4GALT1, TESC, IL6, AIMP1, IL8, JARID2, KLF10, ANXA1, ROGDI, SLAMF1, FOXP1, SOD2, CORO1A, ID2, HBEGF, KLF4	
GO:0030595~leukocyte chemotaxis	3.17E-04	CORO1A, IL6, CCL2, IL8, IL16, CXCL3, S100A9, IL1B, PF4	1.80E-02
GO:0050900~leukocyte migration	3.58E-04	B4GALT1, CORO1A, IL6, CCL2, AIMP1, IL8, IL16, CXCL3, S100A9, IL1B, PF4	1.99E-02
GO:0001819~positive regulation of cytokine production	3.70E-04	IL6, TLR2, PF4, NLRP3, CD83, EREG, RIPK2, BCL3, IL1B, RARA, CLEC7A, CASP1, THBS1, IL1A	2.02E-02
GO:0034097~response to cytokine stimulus	3.85E-04	IRAK2, KYNU, PTGS2, MCL1, SOCS3, JUNB, GCH1, CTNNB1, IRAK3, CORO1A, RIPK2, TAF9, FOSL1	2.06E-02
GO:0060326~cell chemotaxis	4.64E-04	CORO1A, IL6, CCL2, IL8, IL16, CXCL3, S100A9, IL1B, PF4	2.44E-02
GO:0019221~cytokine-mediated signaling pathway	4.96E-04	IRAK2, CSF3, PLP2, KLF6, IRAK3, STAT4, IL6, CCL2, EREG, IL1B, PF4, IL1A	2.56E-02
GO:0045637~regulation of myeloid cell differentiation	4.96E-04	ZFP36, INHBA, TAL1, TESC, HMGB3, ID2, IKZF1, KLF10, NFKBIA, PF4, RUNX1, CTNNB1	2.56E-02
GO:0002696~positive regulation of leukocyte activation	5.70E-04	FCER1A, IL6, IKZF1, CD3E, TNFSF14, IL15, PNP, ADA, CD47, CD83, CORO1A, RIPK2, IL1B, RARA, THBS1	2.88E-02
GO:0006357~regulation of transcription from RNA polymerase II promoter	6.19E-04	MEF2C, CASK, CTNNB1, EPC1, GTF2A2, SIK1, FOSL1, MYC, EGR1, CCNH, PKIG, FOSB, MBD2, JUNB, MED10, RBBP8, OSM, INHBA, ADRB2, MTF1, RPS14, TGIF1, LCOR, KDM6B, HMGB1, HMGB2, ZNF76, NFKBIA, TAL1, BCL3, CHD1, TAF9, RUNX1, ETV3, KLF6, IL6, NFE2, CEBPB, IKZF1, SUB1, KLF13, JARID2, KLF10,	3.07E-02

		SRA1, RYBP, NR4A2, NR4A1, NR4A3, FOXP1, SOD2, ID2, IRF7, CSRNP1, IRF1, HIVEP1, KLF4	
GO:0045639~positive regulation of myeloid cell differentiation	8.49E-04	INHBA, TAL1, TESC, ID2, IKZF1, KLF10, PF4, RUNX1	4.12E-02
GO:0006917~induction of apoptosis	8.61E-04	BID, ADORA2A, IL19, TLR2, STK17B, TNFSF14, PMAIP1, PTEN, CRADD, GCH1, CUL5, PLEKHG2, ERCC6, SOS2, BCL3, CASP1, MYC, PHLDA1, CEBPB, CD3E, KLF10, CIDEB, NR4A1, NLRP3, NLRP1, BCL2L11, TNFSF8, INHBA, TNFSF10, RNF7	4.10E-02
GO:0042273~ribosomal large subunit biogenesis	9.00E-04	RPL35A, RPL7, RPL26, RPL5, RPL24	4.21E-02
GO:0050867~positive regulation of cell activation	9.08E-04	FCER1A, IL6, IKZF1, CD3E, TNFSF14, IL15, PNP, ADA, CD47, CD83, CORO1A, RIPK2, IL1B, RARA, THBS1	4.18E-02
GO:0012502~induction of programmed cell death	9.11E-04	BID, ADORA2A, IL19, TLR2, STK17B, TNFSF14, PMAIP1, PTEN, CRADD, GCH1, CUL5, PLEKHG2, ERCC6, SOS2, BCL3, CASP1, MYC, PHLDA1, CEBPB, CD3E, KLF10, CIDEB, NR4A1, NLRP3, NLRP1, BCL2L11, TNFSF8, INHBA, TNFSF10, RNF7	4.13E-02
GO:0002221~pattern recognition receptor signaling pathway	9.90E-04	IRAK2, IRAK3, TLR2, NFKBIA, RIPK2, CLEC7A	4.41E-02
GO:0051094~positive regulation of developmental process	1.00E-03	CSF2, PF4, PNP, ADA, CTNNB1, TAL1, IL1B, RARA, THBS1, NRG1, RUNX1, IL1A, FNDC3B, B4GALT1, IL6, TESC, IKZF1, SOCS3, KLF10, JUNB, CD83, INHBA, ADRB2, ID2, BTG1, TGIF1, TGIF2	4.39E-02
GO:0048584~positive regulation of response to stimulus	1.11E-03	IRAK2, FCER1A, IL6, IL16, IL8, CD3E, TLR2, NFKBIA, IL15, ADA, UIMC1, B2M, CFP, OSM, IRAK3, CD55, EREG, IRF7, RIPK2, IL1B,	4.77E-02

		TAF9, CLEC7A, THBS1, HLA-DRA	

Biological Process (BP_FAT) associated with genes having increased expression in **H_RM** with respect to H_Monocyte

Term	PValue	Genes	Benjamini
GO:0070482~response to oxygen levels	1.46E-05	KCNMA1, ACE, EPAS1, TFRC, PDPN, EGLN3, BNIP3, BCL2L1, MMP14, MMP2, ANGPTL4	0.02
GO:0044243~multicellular organismal catabolic process	1.81E-05	MMP10, ACE, MMP9, MMP3, MMP2, MMP1	0.01
GO:0044259~multicellular organismal macromolecule metabolic process	4.43E-05	MMP10, ACE, MMP9, MMP3, MMP2, MMP1	0.02
GO:0001666~response to hypoxia	6.03E-05	KCNMA1, ACE, EPAS1, TFRC, EGLN3, BNIP3, BCL2L1, MMP14, MMP2, ANGPTL4	0.02
GO:0033344~cholesterol efflux	9.01E-05	NPC1, APOE, APOC1, SCARB1, ABCG1	0.03
GO:0044236~multicellular organismal metabolic process	1.07E-04	MMP10, ACE, MMP9, MMP3, MMP2, MMP1	0.03
GO:0030574~collagen catabolic process	1.11E-04	MMP10, MMP9, MMP3, MMP2, MMP1	0.03
GO:0009611~response to wounding	1.76E-04	A2M, C3, PDPN, IDO1, C1QC, IL10, CD9, C1QB, TFRC, NUPR1, CD59, PLA2G7, MGLL, SCARB1, ID3, PLA2G4C, TFPI2, IGFBP4, SPP1	0.03
GO:0006954~inflammatory response	3.13E-04	A2M, C3, PDPN, IDO1, C1QC, IL10, C1QB, TFRC, NUPR1, PLA2G7, MGLL, PLA2G4C, IGFBP4, SPP1	0.05
GO:0010033~response to organic substance	4.14E-04	ME1, KCNMA1, A2M, ACP5, IDO1, BCL2L1, MMP3, MMP14, IL10, ABCG1, C1QB, GLUL, TFRC, APOE, ENO2, SRR, FABP3, IDH1, SCARB1, ID3, SPP1, ADAM9	0.06
GO:0034375~high-density lipoprotein particle remodeling	4.30E-04	APOE, APOC1, SCARB1, ABCG1	0.06
GO:0032963~collagen metabolic process	4.35E-04	MMP10, MMP9, MMP3, MMP2, MMP1	0.06
GO:0051043~regulation of membrane protein ectodomain proteolysis	5.53E-04	APOE, SH3D19, IL10, ADAM9	0.07
GO:0031329~regulation of	1.13E-03	NPC1, APOE, APOC1,	0.12

cellular catabolic process		SH3D19, IL10, ADAM9	
GO:0015918~sterol transport	1.28E-03	NPC1, APOE, APOC1, SCARB1, ABCG1	0.13
GO:0030301~cholesterol transport	1.28E-03	NPC1, APOE, APOC1, SCARB1, ABCG1	0.13
GO:0006979~response to oxidative stress	1.32E-03	EPAS1, APOE, GPX3, IDH1, BCL2L1, SEPP1, MMP14, NQO1, ADAM9	0.12
GO:0008283~cell proliferation	1.60E-03	PDPN, MMP14, RACGAP1, IL10, VEGFB, SHB, DAB2, GLUL, STAT4, ACE, CD81, CD274, SCARB1, IGFBP4, EMP1	0.14
GO:0002526~acute inflammatory response	1.69E-03	C1QB, A2M, NUPR1, TFRC, C3, IDO1, C1QC	0.14
GO:0034369~plasma lipoprotein particle remodeling	2.06E-03	APOE, APOC1, SCARB1, ABCG1	0.16
GO:0034368~protein-lipid complex remodeling	2.06E-03	APOE, APOC1, SCARB1, ABCG1	0.16
GO:0034367~macromolecular complex remodeling	2.06E-03	APOE, APOC1, SCARB1, ABCG1	0.16
GO:0009719~response to endogenous stimulus	2.35E-03	KCNMA1, ME1, A2M, BCL2L1, MMP3, MMP14, ABCG1, IL10, C1QB, FABP3, ENO2, IDH1, SPP1, ADAM9	0.17
GO:0006952~defense response	2.59E-03	A2M, DEFB124, PDPN, C3, BNIP3, IDO1, C1QC, IL10, C1QB, INHBA, NUPR1, TFRC, HAMP, PLA2G7, MGLL, PLA2G4C, IGFBP4, SPP1	0.18
GO:0006869~lipid transport	2.78E-03	ACE, NPC1, APOE, FABP3, APOC1, SCARB1, ABCG1, PLTP	0.18
GO:0045834~positive regulation of lipid metabolic process	2.89E-03	APOE, CD81, APOC1, ABCG1, NR1H3	0.18
GO:0009725~response to hormone stimulus	2.96E-03	KCNMA1, ME1, A2M, BCL2L1, MMP14, ABCG1, IL10, C1QB, FABP3, ENO2, IDH1, SPP1, ADAM9	0.18
GO:0002250~adaptive immune response	3.19E-03	C1QB, IL18BP, C3, LY9, C1QC, IL10	0.19
GO:0002460~adaptive immune response based on	3.19E-03	C1QB, IL18BP, C3, LY9, C1QC, IL10	0.19

somatic recombination of immune receptors built from immunoglobulin superfamily domains			
GO:0048545~response to steroid hormone stimulus	3.53E-03	KCNMA1, C1QB, A2M, ENO2, IDH1, MMP14, IL10, ADAM9, SPP1	0.20
GO:0006955~immune response	3.62E-03	HLA-DQB1, CSF3, CSF2, HLA-DRB1, CXCL5, C3, BNIP3, ACP5, LY9, C1QC, IL10, HLA-DQA1, C1QB, IL18BP, FCGR2B, HAMP, HMHB1, CD274, TREM2	0.19
GO:0001558~regulation of cell growth	3.75E-03	VEGFB, INHBA, DAB2, PLXNA3, APOE, HTRA4, CISH, IGFBP4, SPP1	0.19
GO:0055114~oxidation reduction	3.83E-03	ME1, PTGR1, AIFM2, HSD17B14, SCD, EGLN3, IL4I1, CYB5A, IDO1, VAT1, DHRS3, P4HA2, CYP27A1, P4HA1, GPX3, IDH1, SPR, NQO1	0.19
GO:0010876~lipid localization	4.32E-03	ACE, NPC1, APOE, FABP3, APOC1, SCARB1, ABCG1, PLTP	0.21
GO:0010872~regulation of cholesterol esterification	4.43E-03	APOE, APOC1, ABCG1	0.20
GO:0032101~regulation of response to external stimulus	4.63E-03	A2M, NPC1, C3, PDPN, APOE, IDO1, IL10, SPP1	0.21
GO:0030162~regulation of proteolysis	4.84E-03	A2M, APOE, SH3D19, IL10, ADAM9	0.21
GO:0046486~glycerolipid metabolic process	5.12E-03	SERINC2, PIGK, APOE, CD81, FABP3, APOC1, PLA2G4C, NR1H3	0.21
GO:0010035~response to inorganic substance	5.22E-03	KCNMA1, A2M, NPC1, TFRC, APOE, GPX3, BCL2L1, MT1H, ADAM9	0.21
GO:0051044~positive regulation of membrane protein ectodomain proteolysis	5.64E-03	APOE, SH3D19, ADAM9	0.22
GO:0048771~tissue remodeling	5.89E-03	CTSK, ACE, EPAS1, ACP5, MMP14	0.23
GO:0008203~cholesterol metabolic process	6.80E-03	CYP27A1, APOE, APOC1, LSS, SCARB1, ABCG1	0.25
GO:0033700~phospholipid efflux	6.99E-03	APOE, APOC1, ABCG1	0.25
GO:0050866~negative regulation of cell activation	7.96E-03	INHBA, APOE, CD274, IDO1, IL10	0.27

GO:0009894~regulation of catabolic process	8.11E-03	NPC1, APOE, APOC1, SH3D19, IL10, ADAM9	0.27
GO:0010885~regulation of cholesterol storage	8.48E-03	SCARB1, ABCG1, NR1H3	0.28
GO:0016125~sterol metabolic process	9.99E-03	CYP27A1, APOE, APOC1, LSS, SCARB1, ABCG1	0.31

Biological Process (BP_FAT) associated with genes having increased expression in **H_NeoHep** with respect to H_Monocyte

Term	PValue	Genes	Benjamini
GO:0006979~response to oxidative stress	0.00	ALS2, EPAS1, APOE, CYGB, SEPP1, MMP14, ETV5, ADAM9, DHCR24	0.38
GO:0002526~acute inflammatory response	0.00	C1QA, C1QB, A2M, NUPR1, TFRC, C3, C1QC	0.34
GO:0070482~response to oxygen levels	0.00	KCNMA1, EPAS1, TFRC, PDPN, EGLN3, MMP14, MMP2, DPP4	0.29
GO:0001501~skeletal system development	0.00	BGLAP, HOXA3, CMKLR1, MMP9, ACP5, MMP14, GPNMB, PAPSS2, MMP2, IGFBP4, SPP1	0.51
GO:0051605~protein maturation by peptide bond cleavage	0.00	C1QA, C1QB, C3, MMP14, C1QC, DHCR24	0.45
GO:0006954~inflammatory response	0.00	C1QA, C1QB, A2M, NUPR1, TFRC, C3, PDPN, MGLL, C1QC, IGFBP4, SPP1	0.42
GO:0001666~response to hypoxia	0.00	KCNMA1, EPAS1, TFRC, EGLN3, MMP14, MMP2, DPP4	0.45
GO:0006958~complement activation, classical pathway	0.00	C1QA, C1QB, C3, C1QC	0.46
GO:0009611~response to wounding	0.00	A2M, C3, PDPN, C1QC, C1QA, C1QB, TFRC, NUPR1, CD59, MGLL, CTSB, PAPSS2, IGFBP4, SPP1	0.46
GO:0002455~humoral immune response mediated by circulating immunoglobulin	0.00	C1QA, C1QB, C3, C1QC	0.45
GO:0002449~lymphocyte mediated immunity	0.01	C1QA, C1QB, CADM1, C3, C1QC	0.56
GO:0005976~polysaccharide metabolic process	0.01	IL6ST, PPP1R1A, UGDH, B3GNT1, FUCA1, CHIT1	0.53
GO:0016485~protein processing	0.01	C1QA, C1QB, C3, MMP14, C1QC, DHCR24	0.52
GO:0032101~regulation of response to external stimulus	0.01	A2M, NPC1, C3, PDPN, APOE, IL6ST, SPP1	0.50
GO:0046486~glycerolipid metabolic process	0.01	SERINC2, PLA2G15, APOE, IL6ST, FABP3, APOC1, NR1H3	0.50
GO:0002460~adaptive immune response based on somatic	0.01	C1QA, C1QB, CADM1, C3, C1QC	0.55

recombination of immune receptors built from immunoglobulin superfamily domains			
GO:0002250~adaptive immune response	0.01	C1QA, C1QB, CADM1, C3, C1QC	0.55
GO:0051604~protein maturation	0.01	C1QA, C1QB, C3, MMP14, C1QC, DHCR24	0.55
GO:0006959~humoral immune response	0.01	C1QA, C1QB, C3, TREM2, C1QC	0.54
GO:0060348~bone development	0.01	BGLAP, ACP5, MMP14, GPNMB, MMP2, SPP1	0.52
GO:0006956~complement activation	0.01	C1QA, C1QB, C3, C1QC	0.50

Biological Process (BP_FAT) associated with genes having increased expression in **HNP_NeoHep** with respect to H_Monocyte

Term	PValue	Genes	Benjamini
GO:0002526~acute inflammatory response	1.87E-06	C1QA, C1QB, APOA2, A2M, NUPR1, TFRC, C3, SAA1, VSIG4, C1QC, FN1, ACVR1	0.00
GO:0034381~lipoprotein particle clearance	7.66E-06	APOA2, MSR1, APOE, APOC3, APOC1, SCARB1	0.01
GO:0042592~homeostatic process	8.60E-06	ATOX1, OXT, CSF1, SLC7A8, ACP5, GPX1, APOA2, DNAJC16, HAMP, APOE, SAA1, APOC3, TGM2, SCARB1, SLC39A4, MT1H, AKR1C1, EIF2B4, MT3, SLC12A6, LPL, LIPA, EPAS1, ACO1, SLC12A5, RAD50, CTSK, P2RX7, TFRC, TXNRD3, FABP4, TXNRD1, PARP1	0.01
GO:0006979~response to oxidative stress	1.11E-05	EPAS1, ATOX1, GPX1, UCP2, APOE, NDUFS8, GPX3, IDH1, CYGB, SEPP1, NQO1, ETV5, DHCR24, MT3	0.01
GO:0032368~regulation of lipid transport	1.54E-05	APOA2, P2RX7, APOE, OXT, APOC3, APOC1, NR1H3	0.01
GO:0009636~response to toxin	1.54E-05	GPX1, PTGR1, NUPR1, SLC7A8, CDH1, BPHL, NQO1, MT1H, AKR1C1	0.01
GO:0055114~oxidation reduction	1.99E-05	TM7SF2, HSD17B14, HSD3B7, EHHADH, ALDH1L2, GPX1, PLOD1, DHCR7, GPX3, NDUFS8, VKORC1, IDH1, NQO1, BDH1, AKR1C1, DHCR24, PTGR1, AKR1E2, SCD, VAT1, DIO2, CYP27A1, ALOX15B, TXNRD3, PHGDH, FDX1L, ASPHD1, TXNRD1, DCXR	0.01
GO:0034368~protein-lipid complex remodeling	2.56E-05	LPL, APOA2, APOE, APOC3, APOC1, SCARB1	0.01
GO:0034367~macromolecular complex remodeling	2.56E-05	LPL, APOA2, APOE, APOC3, APOC1, SCARB1	0.01
GO:0034369~plasma lipoprotein particle remodeling	2.56E-05	LPL, APOA2, APOE, APOC3, APOC1, SCARB1	0.01

GO:0034375~high-density lipoprotein particle remodeling	5.02E-05	APOA2, APOE, APOC3, APOC1, SCARB1	0.01
GO:0015918~sterol transport	5.39E-05	APOA2, MSR1, APOE, APOC3, APOC1, SCARB1, AKR1C1	0.01
GO:0030301~cholesterol transport	5.39E-05	APOA2, MSR1, APOE, APOC3, APOC1, SCARB1, AKR1C1	0.01
GO:0006869~lipid transport	7.88E-05	APOA2, P2RX7, ACE, MSR1, APOE, APOC3, FABP3, APOC1, SCARB1, AKR1C1, PLTP, SLC27A4	0.01
GO:0008202~steroid metabolic process	9.80E-05	TM7SF2, LIPA, HSD17B14, HSD3B7, APOC1, AFP, APOA2, CYP27A1, APOE, DHCR7, APOC3, SCARB1, AKR1C1, DHCR24	0.02
GO:0016125~sterol metabolic process	1.04E-04	TM7SF2, APOA2, LIPA, CYP27A1, APOE, DHCR7, APOC3, APOC1, SCARB1, DHCR24	0.02
GO:0006641~triglyceride metabolic process	1.29E-04	LPL, GPX1, APOA2, APOE, APOC3, APOC1, NR1H3	0.02
GO:0010876~lipid localization	1.60E-04	APOA2, P2RX7, ACE, MSR1, APOE, APOC3, FABP3, APOC1, SCARB1, AKR1C1, PLTP, SLC27A4	0.02
GO:0048878~chemical homeostasis	2.12E-04	SLC12A6, LPL, EPAS1, ACO1, ATOX1, OXT, SLC12A5, SLC7A8, APOA2, P2RX7, TFRC, SAA1, HAMP, APOE, APOC3, TGM2, FABP4, SCARB1, SLC39A4, MT1H, AKR1C1, EIF2B4, MT3	0.03
GO:0006639~acylglycerol metabolic process	2.70E-04	LPL, GPX1, APOA2, APOE, APOC3, APOC1, NR1H3	0.03
GO:0008203~cholesterol metabolic process	2.97E-04	TM7SF2, APOA2, CYP27A1, APOE, DHCR7, APOC3, APOC1, SCARB1, DHCR24	0.03
GO:0006638~neutral lipid metabolic process	3.02E-04	LPL, GPX1, APOA2, APOE, APOC3, APOC1, NR1H3	0.03
GO:0009611~response to wounding	3.36E-04	A2M, LIPA, C3, GAL, C1QC, COL5A1, C1QA, GPX1, C1QB, APOA2, CCL22, P2RX7, LAMB2, NUPR1, TFRC, SAA1, CD59, SCARB1, CTSB,	0.03

		VSIG4, ACVR1, FN1, SPP1	
GO:0055088~lipid homeostasis	3.37E-04	LPL, APOA2, APOE, APOC3, FABP4, SCARB1, AKR1C1	0.03
GO:0006662~glycerol ether metabolic process	3.37E-04	LPL, GPX1, APOA2, APOE, APOC3, APOC1, NR1H3	0.03
GO:0033344~cholesterol efflux	3.55E-04	APOA2, APOE, APOC3, APOC1, SCARB1	0.03
GO:0006954~inflammatory response	3.61E-04	A2M, LIPA, C3, GAL, C1QC, C1QA, C1QB, APOA2, CCL22, P2RX7, NUPR1, TFRC, SAA1, VSIG4, FN1, SPP1, ACVR1	0.03
GO:0018904~organic ether metabolic process	4.16E-04	LPL, GPX1, APOA2, APOE, APOC3, APOC1, NR1H3	0.03
GO:0032374~regulation of cholesterol transport	4.37E-04	APOA2, APOE, APOC3, APOC1, NR1H3	0.03
GO:0032371~regulation of sterol transport	4.37E-04	APOA2, APOE, APOC3, APOC1, NR1H3	0.03
GO:0050727~regulation of inflammatory response	5.00E-04	GPX1, A2M, C3, SAA1, APOE, CD276, TGM2, FABP4	0.04
GO:0055092~sterol homeostasis	6.32E-04	APOA2, APOE, APOC3, FABP4, SCARB1, AKR1C1	0.04
GO:0042632~cholesterol homeostasis	6.32E-04	APOA2, APOE, APOC3, FABP4, SCARB1, AKR1C1	0.04
GO:0033700~phospholipid efflux	6.80E-04	APOA2, APOE, APOC3, APOC1	0.05
GO:0046486~glycerolipid metabolic process	8.61E-04	LPL, SERINC2, GPX1, APOA2, PLA2G15, APOE, APOC3, FABP3, APOC1, PIP5K1C, NR1H3	0.06
GO:0010885~regulation of cholesterol storage	9.22E-04	LPL, MSR1, SCARB1, NR1H3	0.06
GO:0032370~positive regulation of lipid transport	9.22E-04	P2RX7, APOE, OXT, NR1H3	0.06
GO:0065005~protein-lipid complex assembly	1.21E-03	APOA2, APOE, APOC3, APOC1	0.07
GO:0034377~plasma lipoprotein particle assembly	1.21E-03	APOA2, APOE, APOC3, APOC1	0.07
GO:0010033~response to organic substance	1.85E-03	A2M, OXT, ACP5, CDH1, ACP2, EDEM3, APOA2, APOE, COL6A2, ENO2, VKORC1, SRR, IDH1, SCARB1, EIF2B4, SPP1, AARS, GAL, C1QB, AFP, P2RX7, TFRC, FABP3, HSPB1, FABP4, PARP1	0.11
GO:0051346~negative	1.85E-03	GPX1, APOA2, APOC3,	0.10

regulation of hydrolase activity		APOC1, GCHFR, DHCR24	
GO:0030500~regulation of bone mineralization	2.14E-03	P2RX7, BGLAP, CD276, ANKH, ACVR1	0.12
GO:0016053~organic acid biosynthetic process	2.36E-03	LPL, PLOD1, PTGDS, ASS1, HSD3B7, SDS, ALOX15B, SCD, SRR, PHGDH	0.12
GO:0046394~carboxylic acid biosynthetic process	2.36E-03	LPL, PLOD1, PTGDS, ASS1, HSD3B7, SDS, ALOX15B, SCD, SRR, PHGDH	0.12
GO:0032369~negative regulation of lipid transport	2.41E-03	APOA2, APOC3, APOC1, NR1H3	0.12
GO:0044259~multicellular organismal macromolecule metabolic process	2.42E-03	P2RX7, ACE, MMP9, MMP2, COL5A1	0.12
GO:0055080~cation homeostasis	2.59E-03	ACO1, ATOX1, OXT, SLC12A5, SLC7A8, P2RX7, TFRC, SAA1, APOE, HAMP, TGM2, SLC39A4, MT1H, MT3	0.13
GO:0019725~cellular homeostasis	2.61E-03	SLC12A6, ACO1, ATOX1, OXT, SLC12A5, GPX1, P2RX7, DNAJC16, TFRC, SAA1, HAMP, APOE, TXNRD3, TGM2, TXNRD1, SLC39A4, MT1H, EIF2B4, MT3	0.12
GO:0070167~regulation of biomineral formation	2.73E-03	P2RX7, BGLAP, CD276, ANKH, ACVR1	0.13
GO:0030003~cellular cation homeostasis	2.75E-03	ACO1, ATOX1, OXT, SLC12A5, P2RX7, TFRC, APOE, HAMP, SAA1, TGM2, SLC39A4, MT1H, MT3	0.12
GO:0032101~regulation of response to external stimulus	2.80E-03	GPX1, A2M, C3, SAA1, APOE, VKORC1, CD276, TGM2, FABP4, SPP1	0.12
GO:0006518~peptide metabolic process	2.88E-03	GPX1, ACE, GPX3, TXNRD3, MME, IDH1	0.12
GO:0043691~reverse cholesterol transport	2.92E-03	APOA2, APOE, APOC3, SCARB1	0.12
GO:0015914~phospholipid transport	3.06E-03	APOA2, P2RX7, APOE, APOC3, APOC1	0.13
GO:0030278~regulation of ossification	3.18E-03	P2RX7, BGLAP, OXT, CSF1, CD276, ANKH, ACVR1	0.13
GO:0010886~positive regulation of cholesterol storage	3.28E-03	LPL, MSR1, SCARB1	0.13

GO:0034382~chylomicron remnant clearance	3.28E-03	APOE, APOC3, APOC1	0.13
GO:0060249~anatomical structure homeostasis	3.49E-03	P2RX7, CTSK, LIPA, EPAS1, CSF1, ACP5, PARP1, RAD50	0.13
GO:0048771~tissue remodeling	3.67E-03	CTSK, ACE, LIPA, EPAS1, TGM2, ACP5	0.14
GO:0006631~fatty acid metabolic process	3.83E-03	LPL, PTGR1, PLA2G15, LIPA, PTGDS, ALOX15B, SCD, EHHADH, FABP3, FABP4, SLC27A4	0.14
GO:0031348~negative regulation of defense response	4.22E-03	GPX1, A2M, SAA1, APOE, CD276	0.15
GO:0019218~regulation of steroid metabolic process	4.22E-03	APOA2, APOE, DHCR7, APOC1, GAL	0.15
GO:0044236~multicellular organismal metabolic process	4.66E-03	P2RX7, ACE, MMP9, MMP2, COL5A1	0.16
GO:0016485~protein processing	4.73E-03	C1QA, C1QB, P2RX7, C3, PARP1, VSIG4, C1QC, DHCR24	0.16
GO:0034384~high-density lipoprotein particle clearance	4.86E-03	APOA2, APOE, SCARB1	0.16
GO:0048871~multicellular organismal homeostasis	4.86E-03	GPX1, P2RX7, CTSK, LIPA, EPAS1, CSF1, ACP5	0.16
GO:0016042~lipid catabolic process	4.91E-03	LPL, APOA2, PLA2G15, LIPA, HSD17B14, APOE, PLA2G12A, EHHADH, APOC3, SCARB1	0.16
GO:0051259~protein oligomerization	5.06E-03	P2RX7, GPX3, SRR, TGM2, HR, CDH1, QPRT, AKR1C1, GCHFR, PARVA	0.16
GO:0001894~tissue homeostasis	6.09E-03	P2RX7, CTSK, LIPA, EPAS1, CSF1, ACP5	0.19
GO:0010883~regulation of lipid storage	6.48E-03	LPL, MSR1, SCARB1, NR1H3	0.19
GO:0032375~negative regulation of cholesterol transport	6.72E-03	APOA2, APOC3, APOC1	0.20
GO:0032372~negative regulation of sterol transport	6.72E-03	APOA2, APOC3, APOC1	0.20
GO:0060192~negative regulation of lipase activity	6.72E-03	APOA2, APOC3, APOC1	0.20
GO:0010873~positive regulation of cholesterol esterification	6.72E-03	APOA2, APOE, APOC1	0.20
GO:0006956~complement activation	7.35E-03	C1QA, C1QB, C3, VSIG4, C1QC	0.21
GO:0051604~protein	7.50E-03	C1QA, C1QB, P2RX7, C3,	0.21

maturation		PARP1, VSIG4, C1QC, DHCR24	
GO:0002541~activation of plasma proteins involved in acute inflammatory response	7.99E-03	C1QA, C1QB, C3, VSIG4, C1QC	0.22
GO:0034599~cellular response to oxidative stress	7.99E-03	GPX1, EPAS1, GPX3, ETV5, MT3	0.22
GO:0070328~triglyceride homeostasis	8.85E-03	LPL, APOC3, SCARB1	0.24
GO:0006563~L-serine metabolic process	8.85E-03	SDS, SRR, PHGDH	0.24
GO:0010872~regulation of cholesterol esterification	8.85E-03	APOA2, APOE, APOC1	0.24
GO:0050801~ion homeostasis	9.25E-03	SLC12A6, ACO1, ATOX1, OXT, SLC12A5, SLC7A8, P2RX7, TFRC, SAA1, HAMP, APOE, TGM2, SLC39A4, MT1H, EIF2B4, MT3	0.24
GO:0046890~regulation of lipid biosynthetic process	9.37E-03	APOE, DHCR7, APOC3, APOC1, NR1H3	0.24
GO:0045778~positive regulation of ossification	9.47E-03	P2RX7, OXT, CD276, ACVR1	0.24
GO:0030005~cellular di-, tri-valent inorganic cation homeostasis	9.72E-03	P2RX7, TFRC, ATOX1, SAA1, HAMP, ACO1, APOE, OXT, TGM2, SLC39A4, MT1H	0.25
GO:0006873~cellular ion homeostasis	9.84E-03	SLC12A6, ACO1, ATOX1, OXT, SLC12A5, P2RX7, TFRC, SAA1, APOE, HAMP, TGM2, SLC39A4, MT1H, EIF2B4, MT3	0.25

Cellular Component (CC_FAT) associated with genes having decreased expression in **H_RM** with respect to H_Monocyte

Term	PValue	Genes	Benjamini
GO:0031091~platelet alpha granule	9.41E-06	F5, PPBP, GP1BB, CLU, PF4, TREML1, THBS1, ITGA2B	0.00
GO:0030141~secretory granule	1.60E-04	AZU1, ACRBP, F5, PPBP, GP1BB, CAMP, CLU, PF4, TREML1, THBS1, ITGA2B	0.02

Cellular Component (CC_FAT) associated with genes having decreased expression in **H_NeoHep** with respect to H_Monocyte

Term	PValue	Genes	Benjamini
GO:0005829~cytosol	2.49E-17	ITGB3BP, RPL18, NCBP1, FHIT, RPL17, XRCC4, RPL36A, CHMP4B, PDLIM5, SNCA, RPL15, PTTG1, SLC7A5, PNP, VCL, ACTG1, MYD88, MAP3K8, PDE4B, PSIP1, RAB24, PELI2, RPL35A, CSNK1G2, NCF1, FBP1, PDE4D, JUP, PSMA2, NFU1, PSMA1, RPS16, PSMA5, RPS14, EIF2S1, TUBA4A, RPS13, RIPK2, RPS10, GRAP2, PPP5C, SNRPG, EEF1B2, GNAI2, MVD, DIAPH2, NFKBIA, ANAPC10, PPT1, PSMA7, CHMP2B, RBX1, RPS25, RPS27, RILPL2, RPL7, EIF3H, RPL6, RPL9, EIF3E, EIF3F, HNRNPD, RPL3, CDA, RPL5, RPS20, RPL7A, PABPC1, UBE2D1, RPS21, RPS23, AXIN1, RPS24, OSBPL5, UPB1, SMAD3, ARFIP1, RPS6, ATG3, S100A12, PTPN11, EIF4B, HNRNPH2, EIF4E, TXNDC17, HDAC1, PPIA, PRKAR1B, RAP1A, GK, UBE2E1, TLN1, NIPSNAP3A, NFKB1, PRDX3, NFKB2, PTEN, RAB3IP, PEX7, AFMID, ARHGAP4, OFD1, CASP6, NOD2, MAP1LC3A, NUBP1, GSN, COPB1, SDPR, TICAM1, PLCB1, FOSL1, ZFP36, C9ORF89, RAB4A, CYCS, RPS4X, PRKCD, VASP, PFDN2, SERPINB9, CHMP1B, PSME1, ADK, ITGB1BP1, PFDN4, FARSB, UBB, LCP1, LCP2, ALOX12, BID, AP1M2, SNX15, VIM, RPS15A, RPL37, ZBTB16, COMT, RPL38, HPRT1, TCF7L2, GCH1, ZFP36L1, ANXA7, MTHFS, PSMF1,	1.33E-14

		PLEKHG2, TYMP, NUP214, ACSL1, RPL31, HK3, PPP2CA, RPL34, RASGRP2, CAMK2D, UCK2, RPIA, HSPA5, HBB, PSTPIP2, GLRX, DCTD, CSNK1A1, ICA1, DNM1L, AIMP1, PLEK, NAT1, RPL26, RPL27, RGS19, NADK, RPL24, CEP63, TAB2, RALGDS, HSP90B1, ID2, RPL22, FYN, RPL13A, GSK3B, EEF1E1, RPL21, ALOX5, CALM2	
GO:0030529~ribonucleoprotein complex	2.88E-17	RPL18, MRPS36, SRP14, RPL17, NCBP1, MRPL42, RPL36A, LSM6, RPL15, BTBD2, U2AF1, LSM5, MRPL39, MRPL32, MRPL33, RPL36AL, SNRPA1, RPL35A, MAGOH, HNRNPA2B1, RSL1D1, PSMA1, AQR, RPS16, EIF2S1, RPS14, SLU7, RPS13, RPS10, SNRPG, SNRPB2, NAA38, HNRNPL, RPS25, HNRNPM, RPS27, HNRNPK, RPL7, RPL6, HNRNPF, RPL9, RPL3, HNRNPD, RPL5, TAF9, PABPC1, RPL7A, RPS20, HNRNPC, RPS21, RPS23, RPS24, HNRNPAB, MRPS28, SSB, RPS6, HNRNPA0, HNRNPH3, HNRNPH2, RNPC3, POP5, CWC15, PCBP1, MRPL1, MRPL3, PRPF3, RPS4X, WRAP53, CPSF6, UBB, SRP9, SERP1, RPS15A, RPL37, RPL38, QTRT1, RPL31, RPL34, MRPL16, MRPL19, DHX15, MSI2, MRPL18, WDR12, APEX1, NSA2, BCAS2, RPL26, RPL27, RPL24, MRPL21, ILF2, RPL13A, RPL22, RPL21, SYF2, NOP58, RBM14, RBM17	7.71E-15
GO:0005840~ribosome	1.94E-13	MRPS36, RPL18, RPL17, MRPL42, RPL36A, RPL15, MRPL39, MRPL32, MRPL33, RPL36AL, MRPL1, RPL35A,	3.45E-11

		MRPL3, RPS4X, RSL1D1, RPS16, RPS14, RPS13, RPS10, UBB, SERP1, RPS15A, RPL37, QTRT1, RPL38, RPS25, RPS27, RPL7, RPL31, RPL6, RPL9, RPL34, MRPL16, RPL3, MRPL19, MRPL18, RPL5, RPL7A, RPS20, RPS21, APEX1, RPS23, RPS24, MRPS28, RPL26, RPL27, RPL24, RPS6, HNRNPH2, MRPL21, RPL13A, RPL22, RPL21	
GO:0033279~ribosomal subunit	3.59E-12	RPL18, MRPS36, RPL17, MRPL42, RPS15A, RPL37, RPL38, RPS25, RPS27, RPL7, RPL31, RPL6, RPL34, MRPL16, RPL3, RPL5, RPL7A, RPS20, RPS21, MRPL32, RPS23, RPS24, MRPL1, MRPS28, MRPL3, RPL26, RPL24, RPS6, RPS4X, RPS16, RPL22, RPL13A, RPL21, RPS14, RPS13, RPS10, UBB	4.80E-10
GO:0022626~cytosolic ribosome	2.41E-11	RPL18, RPS15A, RPL37, RPL38, RPS25, RPS27, RPL7, RPL6, RPL31, RPL34, RPL3, RPL5, RPL7A, RPS20, RPS21, RPS23, RPS24, RPL26, RPL24, RPS6, RPS4X, RPS16, RPL22, RPS14, RPL21, RPS13, RPS10, UBB	2.58E-09
GO:0043233~organelle lumen	3.33E-11	MEF2C, ITGB3BP, MRPL42, PTGS2, S100A9, PDLIM1, ISG20, EPC1, PRIM2, U2AF1, ERAP2, MRPL39, MRPL32, TBPL1, LUC7L3, GTPBP4, NCF2, SERPING1, MED10, F5, PPBP, RPS14, RPS13, ACAA1, FUS, LMNB1, FARS2, AHCTF1, ACAT1, RPS25, TAL1, VRK1, PRPF40A, SUB1, SMAD3, NXF1, RPS6, RPF2, RPF1, S100A12, OASL, JAZF1, POP5, KLF4, ENY2, NKAP, TAF1C, ATP5E, PDIA3, TSG101, EZH2, GTPBP10, PDIA5, NFKB1, NFKB2,	2.97E-09

		SENP5, FANCI, MCCC1, AEN, QKI, SERPINA1, TWISTNB, MYC, MRPL1, SYMPK, EXOSC8, EXOSC9, MRPL3, SLC25A5, CCNH, EXOSC5, EXOSC3, DARS2, MBD2, RBBP7, GTF2B, RBBP6, C19ORF33, TAF13, NOL11, RBM39, PMPCB, RBM34, POLR2E, SHFM1, ZBTB16, ZNF655, FCF1, MRPL16, WDR12, MRPL18, ACTL6A, THAP2, THBS1, NSA2, ETFA, BCAS2, CES1, BCKDHB, SOD2, ILF2, RBM19, NOP58, ATP5A1, RBM14, MPHOSPH6, CALM2, DUSP6, MRPS36, NCBP1, RPL36A, F13A1, KLHL7, KDM1A, EBNA1BP2, GTF2E2, MUTYH, AKIRIN2, MAGOH, HNRNPA2B1, MTA1, SP140, JUNB, RSL1D1, SLU7, AKAP8, PCCA, SNRPG, HMGB2, MCL1, MVD, CLU, ANAPC10, ZNF330, HNRNPL, HNRNPM, HNRNPK, RPL9, EIF3E, HNRNPF, RPL3, RPL5, TAF9, UBE2D1, RUNX2, SDF4, SRGN, MRPS28, NFE2, MAFB, TAF8, NR4A1, SSB, FOXP1, PPIF, TST, HDAC3, HNRNPH2, EAF1, HDAC1, TMLHE, ALDH2, CARS2, EAF2, HDAC9, UBE2E1, NBN, LYAR, MKI67IP, CASP6, MCM7, GTF2A2, POLG2, MSN, TSEN15, INO80B, ELP4, NOL7, CYCS, ZFX, PRPF3, CDK7, C1QBP, WRAP53, NCOA6, CPSF6, UBB, CPSF3, OAT, ING5, UTP3, SUPT3H, ING3, NFYC, WBP11, PF4, TCF7L2, KIAA0020, MUS81, GTF3C6, HSPA5, GTF3C1, APEX1, PINX1, CEBPB, CEBPG, CREB1, ATP5F1, MED31, ATXN1, MPG, HSP90B1,	

		SON, MED30, ALOX5	
GO:0031974~membrane-enclosed lumen	6.81E-11	MEF2C, ITGB3BP, MRPL42, PTGS2, S100A9, PDLIM1, ISG20, EPC1, PRIM2, U2AF1, ERAP2, MRPL39, MRPL32, TBPL1, LUC7L3, GTPBP4, NCF2, SERPING1, MED10, F5, PPBP, RPS14, RPS13, ACAA1, FUS, LMNB1, FARS2, AHCTF1, ACAT1, RPS25, TAL1, VRK1, PRPF40A, SUB1, SMAD3, NXF1, RPS6, RPF2, RPF1, S100A12, OASL, JAZF1, POP5, KLF4, ENY2, NKAP, TAF1C, ATP5E, PDIA3, TSG101, EZH2, GTPBP10, PDIA5, NFKB1, NFKB2, SENP5, ACN9, FANCI, MCCC1, AEN, QKI, SERPINA1, TWISTNB, MYC, COX17, MRPL1, SYMPK, EXOSC8, EXOSC9, MRPL3, SLC25A5, CCNH, EXOSC5, EXOSC3, DARS2, MBD2, RBBP7, GTF2B, RBBP6, C19ORF33, TAF13, NOL11, RBM39, PMPCB, RBM34, POLR2E, SHFM1, ZBTB16, ZNF655, FCF1, MRPL16, WDR12, MRPL18, ACTL6A, THAP2, THBS1, NSA2, ETFA, BCAS2, CES1, BCKDHB, SOD2, ILF2, RBM19, NOP58, ATP5A1, RBM14, MPHOSPH6, CALM2, DUSP6, MRPS36, NCBP1, RPL36A, F13A1, KLHL7, KDM1A, EBNA1BP2, GTF2E2, MUTYH, AKIRIN2, MAGOH, HNRNPA2B1, MTA1, SP140, JUNB, RSL1D1, SLU7, AKAP8, PCCA, SNRPG, HMGB2, MCL1, MVD, CLU, ANAPC10, ZNF330, HNRNPL, HNRNPM, HNRNPK, RPL9, EIF3E, HNRNPF, RPL3, RPL5, TAF9, UBE2D1, RUNX2, SDF4, SRGN, MRPS28, NFE2, MAFB, TAF8, NR4A1, SSB,	5.21E-09

		FOXP1, PPIF, TST, HDAC3, HNRNPH2, EAF1, HDAC1, TMLHE, ALDH2, CARS2, EAF2, HDAC9, UBE2E1, NBN, LYAR, MKI67IP, CASP6, MCM7, GTF2A2, POLG2, MSN, TSEN15, INO80B, ELP4, NOL7, CYCS, ZFX, PRPF3, CDK7, C1QBP, WRAP53, NCOA6, CPSF6, UBB, CPSF3, OAT, ING5, UTP3, SUPT3H, ING3, NFYC, WBP11, PF4, TCF7L2, KIAA0020, MUS81, GTF3C6, HSPA5, GTF3C1, APEX1, PINX1, CEBPB, CEBPG, CREB1, ATP5F1, MED31, ATXN1, MPG, HSP90B1, SON, MED30, ALOX5	
GO:0070013~intracellular organelle lumen	6.31E-10	MEF2C, ITGB3BP, MRPL42, PTGS2, S100A9, PDLIM1, ISG20, EPC1, PRIM2, U2AF1, ERAP2, MRPL39, MRPL32, TBPL1, LUC7L3, GTPBP4, NCF2, MED10, RPS14, RPS13, ACAA1, FUS, LMNB1, FARS2, AHCTF1, ACAT1, RPS25, TAL1, VRK1, PRPF40A, SUB1, SMAD3, NXF1, RPS6, RPF2, RPF1, S100A12, OASL, JAZF1, POP5, KLF4, ENY2, NKAP, TAF1C, ATP5E, PDIA3, TSG101, EZH2, GTPBP10, PDIA5, NFKB1, NFKB2, SENP5, FANCI, MCCC1, AEN, QKI, TWISTNB, MYC, MRPL1, SYMPK, EXOSC8, EXOSC9, MRPL3, SLC25A5, CCNH, EXOSC5, EXOSC3, DARS2, MBD2, RBBP7, GTF2B, RBBP6, C19ORF33, TAF13, NOL11, RBM39, PMPCB, RBM34, POLR2E, SHFM1, ZBTB16, ZNF655, FCF1, MRPL16, MRPL18, WDR12, ACTL6A, THAP2, NSA2, ETFA, BCAS2, CES1, BCKDHB, SOD2, ILF2, RBM19, NOP58, ATP5A1,	4.22E-08

		RBM14, MPHOSPH6, CALM2, DUSP6, MRPS36, NCBP1, RPL36A, KLHL7, KDM1A, EBNA1BP2, GTF2E2, MUTYH, AKIRIN2, MAGOH, HNRNPA2B1, MTA1, SP140, JUNB, RSL1D1, SLU7, AKAP8, PCCA, SNRPG, HMGB2, MVD, MCL1, ANAPC10, ZNF330, HNRNPL, HNRNPM, HNRNPK, RPL9, EIF3E, HNRNPF, RPL3, RPL5, TAF9, UBE2D1, RUNX2, SDF4, NFE2, MRPS28, MAFB, TAF8, NR4A1, SSB, FOXP1, PPIF, TST, HDAC3, HNRNPH2, EAF1, HDAC1, TMLHE, ALDH2, CARS2, EAF2, HDAC9, UBE2E1, NBN, LYAR, MKI67IP, CASP6, MCM7, GTF2A2, POLG2, MSN, TSEN15, INO80B, ELP4, NOL7, CYCS, ZFX, PRPF3, CDK7, C1QBP, WRAP53, NCOA6, CPSF6, UBB, CPSF3, OAT, ING5, UTP3, SUPT3H, ING3, NFYC, WBP11, TCF7L2, KIAA0020, GTF3C6, MUS81, HSPA5, GTF3C1, APEX1, PINX1, CEBPB, CEBPG, CREB1, ATP5F1, ATXN1, MED31, MPG, HSP90B1, SON, MED30, ALOX5	
GO:0044445~cytosolic part	1.05E-08	RPL18, RPS15A, RPL37, RPL38, PRDX3, TCF7L2, RPS25, RPS27, RPL7, RPL6, RPL31, RPL34, RPL3, RPL5, HSPA5, RPL7A, RPS20, RPS21, HBB, RPS23, RPS24, RPL26, RGS19, RPL24, RPS6, RPS4X, PFDN2, RPS16, RPL22, RPS14, RPL21, PFDN4, RPS13, RPS10, UBB	6.24E-07
GO:0031981~nuclear lumen	1.94E-07	MEF2C, ITGB3BP, NCBP1, RPL36A, S100A9, PDLIM1, ISG20, KLHL7, EPC1, EBNA1BP2, KDM1A, GTF2E2, MUTYH, PRIM2, U2AF1, AKIRIN2, LUC7L3,	1.04E-05

		TBPL1, GTPBP4, NCF2, MAGOH, HNRNPA2B1, MTA1, SP140, MED10, JUNB, RSL1D1, RPS14, RPS13, SLU7, AKAP8, SNRPG, FUS, HMGB2, LMNB1, MCL1, AHCTF1, ANAPC10, ZNF330, RPS25, HNRNPL, TAL1, HNRNPM, VRK1, HNRNPK, RPL9, HNRNPF, EIF3E, RPL3, RPL5, TAF9, UBE2D1, RUNX2, PRPF40A, NFE2, SUB1, MAFB, TAF8, NR4A1, SMAD3, SSB, NXF1, RPS6, RPF2, RPF1, FOXP1, S100A12, OASL, HDAC3, HNRNPH2, EAF1, HDAC1, JAZF1, EAF2, POP5, HDAC9, KLF4, UBE2E1, NKAP, ENY2, TAF1C, NBN, TSG101, LYAR, GTPBP10, EZH2, MKI67IP, NFKB1, NFKB2, SENP5, CASP6, MCM7, FANCI, AEN, GTF2A2, QKI, MSN, TWISTNB, MYC, TSEN15, INO80B, ELP4, SYMPK, EXOSC8, EXOSC9, CCNH, EXOSC5, NOL7, ZFX, EXOSC3, PRPF3, CDK7, RBBP7, MBD2, GTF2B, RBBP6, C19ORF33, TAF13, WRAP53, NCOA6, NOL11, CPSF6, UBB, RBM39, CPSF3, UTP3, ING5, SUPT3H, RBM34, ING3, POLR2E, SHFM1, NFYC, WBP11, ZBTB16, ZNF655, TCF7L2, FCF1, KIAA0020, GTF3C6, MUS81, WDR12, ACTL6A, THAP2, GTF3C1, APEX1, PINX1, NSA2, BCAS2, CEBPB, CEBPG, CREB1, MED31, ATXN1, MPG, SON, MED30, ILF2, RBM19, NOP58, ALOX5, MPHOSPH6, RBM14, CALM2, DUSP6	
GO:0015934~large ribosomal subunit	3.97E-07	RPL18, MRPL1, RPL17, MRPL3, RPL26, RPL37, RPL24, RPL38, RPL7, RPL13A, RPL6, RPL22,	1.93E-05

		RPL31, RPL34, RPL21, MRPL16, RPL3, RPL5, RPL7A, MRPL32	
GO:0043232~intracellular non-membrane-bounded organelle	1.74E-06	ITGB3BP, RPL18, HMGN1, DYNC1LI1, XRCC4, RPL17, MRPL42, HMGN3, HMGN2, VAPA, PDLIM5, SNCA, RPL15, S100A9, PDLIM2, PDLIM1, H1FX, AURKB, CITED2, ACTG1, IFT20, PRIM2, HIST1H2BJ, H2AFZ, NUP37, MRPL39, MRPL32, MRPL33, STAG2, H1F0, GTPBP4, NCF2, DCTN6, MAP1LC3B2, H2AFJ, JUP, SUV420H2, BAZ1A, RPS16, RPS14, TUBA4A, RPS13, RPS10, ARL8B, FUS, LMNB1, STK17B, AHCTF1, RPS25, FAM65B, TAL1, VRK1, CDC42EP2, RPS27, RPS20, RPS21, CDC42EP3, RPS23, RPS24, IKZF1, SUB1, RPS6, RPF2, RPF1, S100A12, OASL, H3F3A, H3F3C, POP5, KLF4, NKAP, TSG101, GTPBP10, TTLL5, MLH3, SENP5, OFD1, MAP1LC3A, FANCI, AEN, QKI, TWISTNB, MYC, SYMPK, MRPL1, EXOSC8, EXOSC9, MRPL3, SLC25A5, ACTA2, EXOSC5, EXOSC3, MBD2, RBBP6, WAS, C19ORF33, SS18, HIST2H2BE, CLIC4, NOL11, TMSB4X, RBM39, SERP1, HIST1H2AC, RBM34, YWHAZ, FHL3, RPL37, ZNF655, QTRT1, ZBTB16, RPL38, FCF1, SUMO2, GPHN, MEFV, RPL31, RPL34, PPP2CA, MRPL16, MRPL19, WDR12, MRPL18, THAP2, NSA2, BCAS2, RPL26, RPL27, RPL24, MRPL21, ILF2, RPL22, RPL21, IFT57, SVIL, NOP58, RBM19, APBB3, RBM14, MPHOSPH6, CALM2, MRPS36, RPL36A, TNNC2, PRC1, CBX3, PNP, VCL,	7.78E-05

		KLHL7, EBNA1BP2, TPT1, STK39, DISC1, RPL36AL, RPL35A, HNRNPA2B1, MTA1, PDE4D, PLK1S1, SP140, JUNB, ARPC1A, RSL1D1, TNNT1, LYST, IGBP1, AKAP8, CSTA, TNFAIP3, HMGB2, ADORA2A, SSH2, CDC42SE1, NEDD9, C14ORF166, MYL9, ZNF330, HNRNPM, PSMB4, HNRNPK, ARPC3, RPL7, RPL6, EIF3E, RPL9, HNRNPF, HNRNPD, RPL3, RPL5, RPL7A, ARHGDIB, MRPS28, NFE2, SMCHD1, FSCN1, MYO1G, SSB, MYL12A, FOXP1, HDAC3, CORO1A, HNRNPH2, HDAC1, TLN1, NBN, HINT1, LYAR, RHOQ, MKI67IP, RHOU, RAB3IP, MCM7, GSN, ACTR1B, DLG4, MSN, POLG2, TSEN15, IFNGR1, INO80B, UBE2A, NOL7, ZFX, MID1IP1, ARHGAP24, RPS4X, VASP, EML2, NCOA6, MAPRE2, UBB, HAUS8, LCP1, UTP3, PPP4R2, VIM, HAUS1, CALCOCO2, RPS15A, TPM1, TPM3, KIAA0020, MUS81, SH2B2, CNN2, APEX1, PSTPIP2, PINX1, CENPN, CREB1, CEBPG, ANXA1, CEP63, COTL1, RGS14, ATXN1, ID2, RPL13A, JAK1, JAK3	
GO:0043228~non-membrane-bounded organelle	1.74E-06	ITGB3BP, RPL18, HMGN1, DYNC1LI1, XRCC4, RPL17, MRPL42, HMGN3, HMGN2, VAPA, PDLIM5, SNCA, RPL15, S100A9, PDLIM2, PDLIM1, H1FX, AURKB, CITED2, ACTG1, IFT20, PRIM2, HIST1H2BJ, H2AFZ, NUP37, MRPL39, MRPL32, MRPL33, STAG2, H1F0, GTPBP4, NCF2, DCTN6, MAP1LC3B2, H2AFJ, JUP, SUV420H2, BAZ1A, RPS16,	7.78E-05

		RPS14, TUBA4A, RPS13, RPS10, ARL8B, FUS, LMNB1, STK17B, AHCTF1, RPS25, FAM65B, TAL1, VRK1, CDC42EP2, RPS27, RPS20, RPS21, CDC42EP3, RPS23, RPS24, IKZF1, SUB1, RPS6, RPF2, RPF1, S100A12, OASL, H3F3A, H3F3C, POP5, KLF4, NKAP, TSG101, GTPBP10, TTLL5, MLH3, SENP5, OFD1, MAP1LC3A, FANCI, AEN, QKI, TWISTNB, MYC, SYMPK, MRPL1, EXOSC8, EXOSC9, MRPL3, SLC25A5, ACTA2, EXOSC5, EXOSC3, MBD2, RBBP6, WAS, C19ORF33, SS18, HIST2H2BE, CLIC4, NOL11, TMSB4X, RBM39, SERP1, HIST1H2AC, RBM34, YWHAZ, FHL3, RPL37, ZNF655, QTRT1, ZBTB16, RPL38, FCF1, SUMO2, GPHN, MEFV, RPL31, RPL34, PPP2CA, MRPL16, MRPL19, WDR12, MRPL18, THAP2, NSA2, BCAS2, RPL26, RPL27, RPL24, MRPL21, ILF2, RPL22, RPL21, IFT57, SVIL, NOP58, RBM19, APBB3, RBM14, MPHOSPH6, CALM2, MRPS36, RPL36A, TNNC2, PRC1, CBX3, PNP, VCL, KLHL7, EBNA1BP2, TPT1, STK39, DISC1, RPL36AL, RPL35A, HNRNPA2B1, MTA1, PDE4D, PLK1S1, SP140, JUNB, ARPC1A, RSL1D1, TNNT1, LYST, IGBP1, AKAP8, CSTA, TNFAIP3, HMGB2, ADORA2A, SSH2, CDC42SE1, NEDD9, C14ORF166, MYL9, ZNF330, HNRNPM, PSMB4, HNRNPK, ARPC3, RPL7, RPL6, EIF3E, RPL9, HNRNPF, HNRNPD, RPL3, RPL5, RPL7A, ARHGDIB, MRPS28, NFE2, SMCHD1, FSCN1,	

		MYO1G, SSB, MYL12A, FOXP1, HDAC3, CORO1A, HNRNPH2, HDAC1, TLN1, NBN, HINT1, LYAR, RHOQ, MKI67IP, RHOU, RAB3IP, MCM7, GSN, ACTR1B, DLG4, MSN, POLG2, TSEN15, IFNGR1, INO80B, UBE2A, NOL7, ZFX, MID1IP1, ARHGAP24, RPS4X, VASP, EML2, NCOA6, MAPRE2, UBB, HAUS8, LCP1, UTP3, PPP4R2, VIM, HAUS1, CALCOCO2, RPS15A, TPM1, TPM3, KIAA0020, MUS81, SH2B2, CNN2, APEX1, PSTPIP2, PINX1, CENPN, CREB1, CEBPG, ANXA1, CEP63, COTL1, RGS14, ATXN1, ID2, RPL13A, JAK1, JAK3	
GO:0022625~cytosolic large ribosomal subunit	2.68E-06	RPL18, RPL26, RPL37, RPL24, RPL38, RPL7, RPL6, RPL22, RPL31, RPL21, RPL34, RPL3, RPL5, RPL7A	1.10E-04
GO:0022627~cytosolic small ribosomal subunit	5.15E-06	RPS15A, RPS4X, RPS6, RPS25, RPS27, RPS16, RPS14, RPS13, RPS10, UBB, RPS20, RPS21, RPS23, RPS24	1.97E-04
GO:0015935~small ribosomal subunit	1.54E-05	MRPS36, MRPL42, MRPS28, RPS15A, RPS6, RPS4X, RPS25, RPS27, RPS16, RPS14, RPS13, RPS10, UBB, RPS20, RPS21, RPS23, RPS24	5.50E-04
GO:0005654~nucleoplasm	6.84E-05	ITGB3BP, MEF2C, NCBP1, RPL36A, PDLIM1, ISG20, KDM1A, EPC1, MUTYH, GTF2E2, PRIM2, U2AF1, TBPL1, LUC7L3, AKIRIN2, MAGOH, HNRNPA2B1, MTA1, MED10, SP140, SLU7, SNRPG, HMGB2, MCL1, AHCTF1, ANAPC10, HNRNPL, TAL1, HNRNPK, EIF3E, HNRNPF, TAF9, UBE2D1, RUNX2, PRPF40A, MAFB, SUB1, TAF8, SMAD3, NR4A1, NXF1, HDAC3, HNRNPH2, HDAC1, EAF1, JAZF1, EAF2, HDAC9, KLF4,	2.28E-03

		UBE2E1, ENY2, TAF1C, NBN, EZH2, NFKB1, MKI67IP, NFKB2, CASP6, MCM7, FANCI, AEN, GTF2A2, MYC, ELP4, SYMPK, CCNH, PRPF3, CDK7, MBD2, RBBP7, GTF2B, TAF13, WRAP53, NCOA6, CPSF6, RBM39, UBB, CPSF3, ING5, SUPT3H, ING3, POLR2E, SHFM1, NFYC, WBP11, ZBTB16, TCF7L2, GTF3C6, WDR12, ACTL6A, APEX1, GTF3C1, CREB1, ATXN1, MED31, MPG, SON, MED30, RBM14, CALM2, DUSP6	
GO:0030530~heterogeneous nuclear ribonucleoprotein complex	1.52E-04	HNRNPL, RPL36A, HNRNPH3, HNRNPH2, HNRNPK, HNRNPF, HNRNPA2B1, HNRNPD, HNRNPA0	4.79E-03
GO:0044451~nucleoplasm part	4.08E-04	ENY2, MEF2C, EZH2, PDLIM1, ISG20, KDM1A, EPC1, GTF2E2, MCM7, GTF2A2, U2AF1, MYC, AKIRIN2, TBPL1, LUC7L3, ELP4, CCNH, MAGOH, MTA1, PRPF3, CDK7, RBBP7, MBD2, GTF2B, MED10, TAF13, WRAP53, NCOA6, CPSF6, SLU7, RBM39, ING5, SUPT3H, ING3, POLR2E, SHFM1, NFYC, WBP11, ZBTB16, TCF7L2, TAL1, EIF3E, GTF3C6, TAF9, ACTL6A, GTF3C1, RUNX2, PRPF40A, MAFB, SUB1, TAF8, CREB1, SMAD3, NXF1, ATXN1, MED31, HDAC3, SON, MED30, EAF1, HDAC1, JAZF1, EAF2, RBM14, HDAC9, KLF4	1.21E-02
GO:0045259~proton-transporting ATP synthase complex	6.94E-04	ATP5E, ATP5S, ATP5F1, ATP5L, ATPIF1, ATP5A1, ATP5H, ATP5J	1.93E-02
GO:0005730~nucleolus	1.19E-03	NKAP, RPL36A, NBN, TSG101, LYAR, GTPBP10, S100A9, MKI67IP, SENP5, KLHL7, EBNA1BP2, AEN, QKI, MSN, TWISTNB,	3.14E-02

		TSEN15, INO80B, GTPBP4, EXOSC8, EXOSC9, NCF2, ZFX, NOL7, HNRNPA2B1, EXOSC5, MTA1, EXOSC3, RBBP6, JUNB, RSL1D1, C19ORF33, RPS14, NCOA6, RPS13, NOL11, AKAP8, FUS, UTP3, RBM34, HMGB2, ZNF655, ZBTB16, FCF1, ZNF330, RPS25, HNRNPM, KIAA0020, VRK1, HNRNPK, HNRNPF, RPL9, RPL3, MUS81, WDR12, RPL5, THAP2, NSA2, PINX1, BCAS2, NFE2, SUB1, CEBPG, SSB, RPS6, RPF2, FOXP1, RPF1, S100A12, ATXN1, OASL, HNRNPH2, ILF2, NOP58, RBM19, RBM14, POP5, MPHOSPH6, KLF4	
GO:0005739~mitochondrion	1.44E-03	MRPS36, MRPL42, CMC1, PMAIP1, HIBADH, MRPL39, MRPL32, MRPL33, WWOX, OMA1, LYRM4, TMEM126A, BCL2L13, COX6C, NFU1, ATPIF1, PCCA, MCL1, ADHFE1, MTX2, FARS2, COX7B, CA5B, MTX1, CLU, COX7C, OAS2, MTIF3, ACAT1, FAM65B, MRPS28, CRAT, ABCB7, PPA2, PTPN11, PPIF, TRNT1, TST, TOMM40L, TMLHE, ALDH2, COQ10B, FOXRED1, CARS2, GK, SLC25A16, UQCRC2, ATP5E, SPG7, PRDX3, UQCRFS1, PTEN, ACN9, TMEM173, NDUFS4, MCCC1, ATP5S, SLC25A3, ATP5L, YRDC, POLG2, ATP5H, COX17, ATP5J, FUNDC2, MRPL1, MRPL3, LIMK2, SLC25A5, CYCS, LGALS12, TMEM70, NDUFC1, CDK7, DARS2, COQ7, COQ6, IMMP1L, C1QBP, CLIC4, UQCRH, SLC27A3, OAT, PMPCB, UQCRB, BID, YWHAZ, QTRT1, COX7A2L, TSC22D3, ACSL1, PPP2CA,	3.61E-02

		MRPL16, THG1L, MRPL19, MRPL18, MYCBP, XAF1, KIAA0391, ETFA, DNM1L, NDUFA6, CREB1, NDUFA7, BCKDHB, ATP5F1, SOD2, MRPL21, FYN, SDHD, ATP5A1	
GO:0044429~mitochondrial part	1.55E-03	MRPS36, UQCRC2, ATP5E, MRPL42, UQCRFS1, ACN9, TMEM173, NDUFS4, MCCC1, ATP5S, SLC25A3, ATP5L, POLG2, MRPL39, MRPL32, COX17, ATP5H, ATP5J, MRPL1, MRPL3, SLC25A5, CYCS, TMEM70, DARS2, NDUFC1, COX6C, IMMP1L, C1QBP, UQCRH, ATPIF1, OAT, PCCA, PMPCB, UQCRB, BID, MCL1, MTX2, FARS2, MTX1, COX7B, COX7C, COX7A2L, ACAT1, ACSL1, MRPL16, MRPL18, ETFA, DNM1L, MRPS28, NDUFA6, NDUFA7, BCKDHB, ATP5F1, CRAT, ABCB7, SOD2, TST, PPIF, FYN, SDHD, TMLHE, TOMM40L, ALDH2, CARS2, ATP5A1, GK, SLC25A16	3.71E-02
GO:0033256~I-kappaB/NF-kappaB complex	1.71E-03	NFKBIA, BCL3, NFKB1, NFKB2	3.90E-02
GO:0005625~soluble fraction	1.89E-03	LDHB, YWHAZ, CYB5R2, ATG12, FARS2, CXCL2, CALCOCO2, COMT, HPRT1, RAB1A, GCH1, ACTG1, ANXA7, ACSL1, SPINT2, RASGRP4, PPP2CA, PDE4B, SDF4, IK, PLEK, LIMK2, CSNK1G2, NCF1, RAB4A, FBP1, MTL5, PDE4D, MANBA, SOD2, PTPN11, JUP, CD55, TNFSF10, CLIC4, FARSB, MAPK8IP3, ALOX5, SYTL1, DUSP6	4.13E-02
GO:0005753~mitochondrial proton-transporting ATP synthase complex	2.31E-03	ATP5E, ATP5F1, ATP5L, ATPIF1, ATP5A1, ATP5H, ATP5J	4.83E-02
GO:0005759~mitochondrial matrix	2.46E-03	MRPS36, ATP5E, MRPL42, FARS2, ACAT1, MCCC1, MRPL16, MRPL18, MRPL39,	4.94E-02

		POLG2, MRPL32, ETFA, MRPL1, MRPS28, MRPL3, SLC25A5, BCKDHB, CYCS, ATP5F1, DARS2, SOD2, PPIF, TST, C1QBP, TMLHE, ALDH2, CARS2, ATP5A1, OAT, PCCA, PMPCB	
GO:0031980~mitochondrial lumen	2.46E-03	MRPS36, ATP5E, MRPL42, FARS2, ACAT1, MCCC1, MRPL16, MRPL18, MRPL39, POLG2, MRPL32, ETFA, MRPL1, MRPS28, MRPL3, SLC25A5, BCKDHB, CYCS, ATP5F1, DARS2, SOD2, PPIF, TST, C1QBP, TMLHE, ALDH2, CARS2, ATP5A1, OAT, PCCA, PMPCB	4.94E-02

Cellular Component (CC_FAT) associated with genes having decreased expression in **HNP_RM** with respect to HNP_Monocyte

Term	PValue	Genes	Benjamini
GO:0005829~cytosol	5.35E-70	RPL18, FHIT, RPL17, LDHA, RPL19, RPL14, RPL13, RPL15, RPLP2, SART1, CTNNB1, ACTG1, RPLP0, RPLP1, MAP3K8, FAU, RPL10, RPL11, RPL12, NCF1, NCF4, EIF2S3, SKP1, MYH9, BCL2L11, UXT, GLUL, PPP1CA, RPS19, RPS16, RPS14, RPS15, RPS12, RPS13, RPS10, RPS11, TRAPPC3, GNAI2, NFKBIB, NFKBIA, PPT1, RPS25, RPS27, RPS28, RILPL2, RPS29, RAC1, DIABLO, RPS20, PABPC1, RPS21, RPS23, RPS24, RPSA, UNC119, ODC1, MAP2K2, SPHK1, GARS, RPS9, HBA2, HBA1, RPS6, RPS5, RPS8, RPS7, HGS, GK, BACH1, AP2S1, QARS, OFD1, AP1S2, DYNLL1, NUBP1, COPB1, TICAM1, DPP7, ARHGEF2, STX4, LYN, DGUOK, CCT6A, FLNA, ELMO1, PFDN2, SERPINB9, PFDN1, RPL41, SERPINB6, SERPINB8, EIF4A2, PFDN5, EIF4A1, TXN, PFDN4, EEF1G, EEF1D, RPL35, HK2, RPL36, HK1, RPL37, ABI1, RPL38, DGKA, RPL30, NUP214, PLEKHG2, ACSL1, EZR, RPL32, RPL31, RPL34, PPP2CA, HK3, PPP3CC, MARS, RPL26, RPL27, NADK, RPL24, RPL28, RPL29, CSNK1D, RPL23, CSNK1E, RPL22, GSK3A, GSK3B, ARAF, TCEB2, DPYD, TCEB1, SPG11, SSNA1, NCBP1, SNRPD3, EIF5, SNRPD1, SNRPD2, SLC7A5, VCL, EIF4EBP1, PDE4B, RPS27A, RPL35A, PDXK, STK24, RELA, FBP1, NRD1, PNPLA2, PDE4D, CORO7, PDCD6IP, NFE2L2, UBA52, ARFGAP3, CNBP, MAPKAPK3, MAPKAPK2, DOCK2, RPL7, RPL6, RPL9, RPL8, RPL3,	3.62E-67

		HNRNPD, RPL5, RPL7A, RPL4, RPL10A, AP2M1, RPL23A, ATG3, VAV1, NMT1, NOSIP, CDKN1A, HNRNPH2, HDAC1, VCP, PRKAR1A, RPL37A, IKBKB, CYB5R3, MAD1L1, KYNU, PRDX5, FES, RPS2, RPS3, CDC42, RNF103, RPS3A, CASP8, IMPDH1, FOSL1, IMPDH2, PPP2R1A, NUP88, SARS, CYCS, ATP6V1H, STXBP2, RPS4X, CDK5, HMGA1, VASP, PRDX6, UBC, HBG2, MAPRE1, UBB, PTMS, LCP1, LCP2, AP1M1, HAUS2, VIM, UPP1, RPS15A, NR3C1, ANXA7, INPP5K, AKR1A1, PAFAH1B1, HSPA5, HBB, HSPA8, WDFY1, RAF1, BAD, XPNPEP1, HSP90B1, ID2, RPL13A, BAX, VPS28, SCPEP1, CHMP5, PDLIM5, SNCA, CCT2, CCT3, MYD88, NT5C2, RAB24, PELI2, FTL, CLNS1A, DBNL, ANAPC5, RAN, DCTN3, DCTN2, AHSP, TUBA4A, MDH1, TALDO1, PFKFB3, AKAP13, ADRBK1, CALR, BANF1, ADA, CDC37, TPI1, CDA, AMD1, ABR, SMAD3, TKT, PTPN12, S100A12, PPA1, PTPN11, CCT7, CCT5, CCT4, RPL18A, CCT8, POMP, IDI1, AHSA1, ANPEP, NFKB1, NFKB2, PTEN, CKB, BAG1, GPX4, TOP2B, AGPAT4, NAPRT1, NT5C, ZFP36, INPPL1, UFD1L, HERC4, GZMB, CHMP1B, ADK, UROD, BID, PPP6C, SDCCAG8, RPL27A, FKBP1A, ATP6V1G1, GCH1, ZFP36L1, SET, KLC1, SQSTM1, TPRKB, CSNK1A1, PLEK, YWHAB, SOD1, BIRC2, YWHAE, PSMD13, AP2A1, FYN, PSMD11, ATP6V1E1, CALM3, CALM2, CALM1, CHMP4B, EIF5A, CASK, PNP, AIP, NANS, PRKACA, MX1, RNF31, CSNK1G2, BCAP31, PSMA2, PSMA1, ISCU, PSMA5, PSMA4,	

		PSMA3, SNRPB, RIPK2, SDCBP, SNRPF, SNRPE, SNRPG, CHMP2A, EEF1B2, MVD, GRB2, COPZ1, KMO, ANAPC10, ANAPC11, ATP6V1B2, PSMA7, FTH1, KARS, SRC, RBX1, EIF3D, PSMB7, EIF3B, PSMB6, PSMB1, BLOC1S2, EIF3G, EIF3H, EIF3E, BLOC1S1, PSMB2, NPM1, EIF3F, EIF3K, EIF3I, UBE2D1, GABARAPL2, PTPN6, EEF1A1, EVL, EIF4B, URM1, EIF4E, TXNDC17, PSMC4, PPIA, EIF4H, RAP1A, RAP1B, PTPN1, WDR1, FPGS, PHPT1, GUK1, UBE2E1, DUT, NAMPT, TLN1, PRKAG2, PDCD4, ARHGAP4, GSN, MAPKAP1, ACTR1A, PSMD2, PSMD4, PAK1, TUBA1A, PSMD6, PLCB1, PLCB2, CLINT1, PSMD8, IRAK1, HSP90AA1, PFKL, C9ORF89, NADSYN1, PRKCD, TARS, DGAT1, PSME1, ITGB1BP1, NUCB2, MDM2, TOM1, DHPS, COPE, PSMB10, COMT, TCF7L2, PSMF1, TYMP, RASGRP2, PYCARD, PIK3R5, GLRX, ENO1, ACTB, TCP1, PARK7, RALGDS, NME1-NME2, TRPC4AP, SH3D19	
GO:0030529~ribonucleoprotein complex	2.69E-55	RPL18, RPL17, NAF1, RPL19, RPL14, RPL13, U2AF2, RPL15, LSM7, RPLP2, RPL22L1, SART1, DHX38, RPLP0, RPLP1, U2AF1, RPL10, FAU, LSM4, MRPL38, RPL11, RPL12, MRPL33, FTL, MRPL53, MRPL52, MRPL51, PTBP1, RPS19, RPS16, RPS14, RPS15, RPS12, RPS13, RPS10, MRPL47, MRPL48, RPS11, NHP2L1, PABPC4, ZCRB1, CALR, NAA38, XAB2, RPS25, HNRNPA3, RPS27, MOV10, RPS28, RPS29, FRG1, MRPL54, ISY1, MRPL55, RPS20, PABPC1, RPS21, RPS23, RPS24, HNRNPAB, RPSA, DHX9, SRA1, RPS9, HBA2, HBA1, DDX5,	9.10E-53

		RPS6, U2AF1L4, HNRNPA1, NOP10, RPS5, RPS8, HNRNPA0, FXR1, RPS7, RPL18A, DCP2, POP4, CWC15, YBX1, SF1, EIF4A3, PIH1D1, RPL41, SNRNP40, PRPF38A, SERP1, RPL35, RPL27A, RPL36, RPL37, RPL38, SF3B5, SF3B2, MRPL20, PRPF19, SF3B1, CYLD, RPL30, RPL32, RPL31, MRPL14, RPL34, DHX15, MRPL18, SNRNP70, NSA2, RBM22, BCAS2, RPL26, RPL27, RPL24, ILF3, RPL28, TRIM21, RPL29, MRPL23, MRPL21, RPL23, ILF2, MRPL28, RPL22, SYF2, NOP58, PHF5A, NOP56, RBM17, TXNL4A, RALY, MRPS36, SRP14, NCBP1, MRPS33, SNRPD3, RBM4, RBM5, SNRPD1, RPS6KB2, SNRPD2, RPS27L, SRP19, SND1, SRRM2, IMP4, RPS27A, RPL36AL, PABPN1, RPL35A, SNRPA1, EFTUD2, MAGOH, FMR1, HNRNPA2B1, EEF2, HNRNPU, PSMA1, MRPS18C, MRPS18A, SNRPB, SNRPA, SLU7, CELF1, SNRPC, SNRPF, SNRPE, UBA52, MVP, SNRPG, MRPS15, STRAP, MRPS11, SNRPB2, HNRNPL, HNRNPM, HNRNPK, RPL7, RPL6, RPL9, HNRNPF, RPL8, NPM1, HNRNPD, RPL3, RPL5, TAF9, RPL7A, RPL10A, HNRNPC, RPL4, DDX41, MRPS28, MRPS24, SNW1, RPL23A, RBMX, HNRNPH3, HNRNPH2, RPL37A, HNRNPH1, PUF60, EDC4, RPS2, RPS3, PLRG1, DKC1, RPS3A, PCBP1, PCBP2, CASP8, MRPS6, PRPF3, RPS4X, NCL, UBC, CPSF6, UBB, RPS15A, IVNS1ABP, PRPF8, MSI2, RSL24D1, APEX1, HSPA8, ACTB, FBL, RPL13A	
GO:0022626~cytosolic ribosome	9.44E-40	RPL18, RPL19, RPL14, RPL13, RPLP2, RPS2, RPS3, RPS3A, RPLP0, RPLP1, RPL10, FAU, RPL11, RPS27A, FTL, RPS4X,	2.13E-37

		RPS19, RPL41, RPS16, RPS14, RPS15, UBC, RPS12, RPS13, RPS10, RPS11, UBB, UBA52, RPL27A, RPL35, RPS15A, RPL36, RPL37, RPL38, RPS25, RPL30, RPS27, RPS28, RPL7, RPL32, RPS29, RPL31, RPL6, RPL34, RPL8, RPL3, RPL5, RPL7A, RPL4, RPS20, RPS21, RPS23, RPS24, RPSA, RPL26, RPS9, RPL23A, HBA2, RPL24, HBA1, RPS6, RPS5, RPS8, RPL28, RPL29, RPS7, RPL18A, RPL22	
GO:0044445~cytosolic part	3.74E-37	CYB5R3, RPL18, RPL19, RPL14, PRDX5, RPLP2, CCT2, CCT3, RPS2, RPS3, CTNNB1, RPS3A, RPLP0, RPLP1, RPL10, FAU, RPL11, RPS27A, FTL, PFKL, CCT6A, RPS4X, UXT, PFDN2, PFDN1, RPS19, RPS16, RPL41, RPS14, PFDN5, PFDN4, AHSP, RPS15, UBC, RPS12, RPS13, RPS10, RPS11, UBB, HBG2, UBA52, RPL27A, RPL35, RPS15A, RPL36, RPL37, RPL38, TCF7L2, RPS25, RPS27, RPL30, RPS28, RPL7, RPL32, BLOC1S2, RPS29, RPL31, RPL6, RPL34, BLOC1S1, RPL8, PYCARD, RPL3, RPL5, RPL7A, RPL4, HSPA5, RPS20, RPS21, HBB, RPS23, RPS24, ENO1, RPSA, TCP1, RPL26, RPS9, RPL24, HBA2, RPL23A, HBA1, RPS6, RPS5, RPS8, RPL28, RPL29, RPS7, CCT7, CCT5, CCT4, RPL18A, RPL22, BAX, IKBKB	6.32E-35
GO:0005840~ribosome	1.98E-36	RPL18, MRPS36, RPL17, MRPS33, RPL19, RPL14, RPL13, RPL15, RPLP2, RPS6KB2, RPS27L, RPL22L1, RPLP0, RPLP1, FAU, RPL10, RPL11, MRPL38, RPL12, MRPL33, RPS27A, RPL36AL, FTL, MRPL53, RPL35A, MRPL52, MRPL51, FMR1, RPS19, MRPS18C, RPS16, MRPS18A, RPS14, RPS15, RPS12, RPS13, RPS10, MRPL47, RPS11,	2.68E-34

		MRPL48, UBA52, MRPS15, MRPS11, RPS25, RPS27, RPS28, RPL7, RPS29, RPL6, RPL9, NPM1, RPL8, RPL3, MRPL54, MRPL55, RPL5, RPL10A, RPL7A, RPL4, RPS20, RPS21, RPS23, RPS24, RPSA, MRPS28, MRPS24, RPS9, HBA2, RPL23A, HBA1, RPS6, RPS5, RPS8, RPS7, HNRNPH2, RPL18A, RPL37A, RPS2, RPS3, RPS3A, SF1, MRPS6, RPS4X, RPL41, UBC, UBB, SERP1, RPL27A, RPL35, RPL36, RPS15A, RPL37, RPL38, MRPL20, CYLD, RPL30, RPL32, RPL31, MRPL14, RPL34, MRPL18, RSL24D1, APEX1, RPL26, RPL27, RPL24, RPL28, RPL29, MRPL23, MRPL21, MRPL28, RPL23, RPL13A, RPL22	
GO:0033279~ribosomal subunit	7.45E-36	MRPS36, RPL18, RPL17, RPL19, MRPS33, RPL14, RPLP2, RPS2, RPS3, RPS3A, RPLP0, RPLP1, RPL10, FAU, RPL11, RPS27A, FTL, MRPL52, MRPL51, MRPS6, RPS4X, RPS19, MRPS18C, RPS16, RPL41, MRPS18A, RPS14, RPS15, UBC, RPS12, RPS13, RPS10, RPS11, UBB, UBA52, MRPS15, MRPS11, RPL27A, RPL35, RPS15A, RPL36, RPL37, RPL38, MRPL20, RPS25, RPS27, RPL30, RPS28, RPL7, RPL32, RPS29, RPL31, RPL6, RPL34, NPM1, RPL8, RPL3, MRPL55, RPL5, RPL7A, RPL4, RPS20, RPS21, RPS23, RPS24, RPSA, MRPS28, MRPS24, RPL26, RPS9, RPL24, RPL23A, HBA2, HBA1, RPS6, RPS5, RPS8, RPL28, RPL29, RPS7, MRPL23, RPL18A, RPL13A, RPL22	8.39E-34
GO:0031966~mitochondrial membrane	3.88E-26	HCCS, TSPO, OXA1L, NDUFAB1, MPV17, STOML2, COX5A, COX5B, MFF, UQCR11, TIMM9, SLC25A28, SLC25A26, CRLS1, POLG, SUCLG1, BCL2L11, COX6C, ATP5C1,	3.74E-24

		SLC25A37, ATPIF1, SLC25A39, SURF1, MDH2, HSD17B10, MCL1, CHKB, COX7B, CHCHD3, COX7C, KMO, HADHA, HADHB, FIS1, TOMM7, TOMM6, MTCH2, TOMM5, GSTK1, MTCH1, COX6B1, ATP5J2, COX8A, AK2, VDAC2, PPIF, SLC25A11, USMG5, UCP2, NDUFV1, COX6A1, GK, SPNS1, SLC25A16, UQCRC2, ATP5D, CYB5R3, ATP5E, UQCRC1, ATP5B, TIMM17A, CYC1, TIMM17B, UQCRFS1, UQCRQ, NDUFAF3, NDUFS6, TMEM173, NDUFS5, NDUFS4, PARL, GPX4, CASP8, SLC25A3, ATP5L, SYNJ2, ATP5O, SLC25A1, ATP5I, NDUFS3, NDUFS2, ATP5H, COX16, ATP5J, SQRDL, NDUFB11, NDUFB10, LYN, SLC25A5, NDUFC2, NDUFA13, COX4I1, NDUFC1, PI4KB, NDUFA10, NDUFA12, NDUFA11, ACADVL, IMMP1L, PSEN1, UQCRH, TOMM20, TOMM22, UQCRB, BID, NDUFB3, NDUFB4, NDUFB5, NDUFB7, NDUFB8, NDUFB9, HK2, TIMM10, HK1, ATP5G2, ATP5G1, COX7A2L, ATP5G3, NDUFB1, NDUFB2, ACSL1, SH3GLB1, CSDE1, ACSL3, SCO2, ACSL5, NDUFA4, NDUFA5, NDUFA2, COX7A2, NDUFA3, NDUFA9, NDUFA6, NDUFA7, ATP5F1, RAF1, BAD, NDUFA1, SOD2, SDHA, SDHB, PHB2, SDHC, BAX, ATP5A1, MGST1	
GO:0005740~mitochondrial envelope	4.42E-26	HCCS, OXA1L, TSPO, NDUFAB1, MPV17, STOML2, COX5A, COX5B, MFF, UQCR11, TIMM9, SLC25A28, SLC25A26, CRLS1, POLG, SUCLG1, BCL2L11, COX6C, ATP5C1, SLC25A37, ATPIF1, SLC25A39, SURF1, MDH2, HSD17B10,	3.74E-24

		MCL1, CHKB, COX7B, CHCHD3, COX7C, KMO, HADHA, HADHB, FIS1, TOMM7, TOMM6, MTCH2, TOMM5, GSTK1, MTCH1, COX6B1, ATP5J2, COX8A, AK2, VDAC2, PPIF, SLC25A11, TXNDC12, USMG5, UCP2, NDUFV1, COX6A1, GK, SPNS1, SLC25A16, UQCRC2, ATP5D, CYB5R3, ATP5E, UQCRC1, ATP5B, TIMM17A, CYC1, TIMM17B, UQCRFS1, UQCRQ, NDUFAF3, NDUFS6, TMEM173, NDUFS5, NDUFS4, PARL, GPX4, CASP8, SLC25A3, ATP5L, SYNJ2, ATP5O, SLC25A1, ATP5I, NDUFS3, NDUFS2, ATP5H, COX17, COX16, ATP5J, SQRDL, NDUFB11, NDUFB10, LYN, SLC25A5, CYCS, NDUFC2, COX4I1, NDUFA13, NDUFC1, PI4KB, NDUFA10, NDUFA12, NDUFA11, ACADVL, IMMP1L, PSEN1, UQCRH, TOMM20, TOMM22, UQCRB, BID, NDUFB3, NDUFB4, FKBP8, NDUFB5, NDUFB7, NDUFB8, NDUFB9, HK2, TIMM10, HK1, ATP5G2, ATP5G1, COX7A2L, ATP5G3, NDUFB1, NDUFB2, ACSL1, SH3GLB1, CSDE1, ACSL3, SCO2, ACSL5, NDUFA4, NDUFA5, NDUFA2, COX7A2, NDUFA3, NDUFA9, NDUFA6, NDUFA7, ATP5F1, RAF1, BAD, NDUFA1, SOD2, SDHA, SDHB, FYN, PHB2, SDHC, BAX, BNIP3L, ATP5A1, MGST1	
GO:0044429~mitochondrial part	1.39E-25	HCCS, MRPS36, OXA1L, GRPEL1, TSPO, MRPS33, NDUFAB1, MPV17, STOML2, COX5A, COX5B, MFF, UQCR11, IDH3G, LRRC59, TIMM9, SLC25A28, SLC25A26, PDHA1, MRPL52, MRPL51, CRLS1, POLG, SUCLG1, BCL2L11, COX6C, MRPS18C, MRPS18A, CLPP, ATP5C1, SLC25A37,	1.04E-23

		ATPIF1, MRPL47, SLC25A39, SURF1, MDH2, HSD17B10, MRPS15, MCL1, FARS2, CHKB, MRPS11, COX7B, CHCHD3, COX7C, KMO, KARS, HADHA, HADHB, FIS1, TOMM7, MTCH2, TOMM6, TOMM5, GSTK1, MTCH1, COX6B1, MRPL55, HSPE1, SUPV3L1, ATP5J2, MRPS28, MRPS24, COX8A, GARS, AK2, IDH3B, VDAC2, PPIF, SLC25A11, TXNDC12, USMG5, UCP2, GLS, NDUFV1, ALDH2, COX6A1, GK, SLC25A16, SPNS1, UQCRC2, ATP5D, CYB5R3, ATP5E, UQCRC1, ATP5B, TIMM17A, TIMM17B, CYC1, QARS, UQCRFS1, UQCRQ, NDUFAF3, NDUFS6, TMEM173, ALAS1, NDUFS5, NDUFS4, PARL, GPX4, CASP8, SLC25A3, ATP5L, SYNJ2, SLC25A1, ATP5O, ATP5I, NDUFS3, NDUFS2, ATP5H, COX17, COX16, ATP5J, SQRDL, NDUFB11, NDUFB10, LYN, SSBP1, SLC25A5, CYCS, NDUFC2, COX4I1, NDUFA13, NDUFC1, PI4KB, NDUFA10, NDUFA12, NDUFA11, IMMP1L, ACADVL, PSEN1, C1QBP, UQCRH, TOMM20, TOMM22, UQCRB, BID, TUFM, NDUFB3, NDUFB4, FKBP8, NDUFB5, NDUFB7, NDUFB8, NDUFB9, ETHE1, HK2, TIMM10, HK1, ATP5G2, ATP5G1, COX7A2L, NR3C1, ATP5G3, NDUFB1, MRPL20, NDUFB2, ACSL1, SH3GLB1, CSDE1, MRPL18, ACSL3, SCO2, ETFB, HSPA9, ACSL5, ETFA, NDUFA4, NDUFA5, NDUFA2, COX7A2, NDUFA3, NDUFA9, NDUFA6, NDUFA7, ATP5F1, RAF1, BAD, SOD1, NDUFA1, SOD2, SDHA, SDHB, MRPL23, MRPL28, FYN, PHB2, SDHC, BAX, BNIP3L, ATP5A1, MGST1	

GO:0019866~organelle inner membrane	4.33E-24	HCCS, EIF6, OXA1L, NDUFAB1, MPV17, CBX3, STOML2, COX5A, COX5B, UQCR11, TIMM9, SLC25A28, SLC25A26, CRLS1, POLG, SUCLG1, COX6C, ATP5C1, SLC25A37, ATPIF1, SLC25A39, SURF1, MDH2, HSD17B10, COX7B, CHCHD3, COX7C, KMO, HADHA, HADHB, MTCH2, GSTK1, MTCH1, COX6B1, ATP5J2, COX8A, SMAD3, AK2, PPIF, SLC25A11, USMG5, UCP2, NDUFV1, COX6A1, SPNS1, SLC25A16, CYB5R3, UQCRC2, ATP5D, ATP5E, UQCRC1, ATP5B, TIMM17A, CYC1, TIMM17B, UQCRFS1, UQCRQ, NDUFAF3, NDUFS6, NDUFS5, NDUFS4, PARL, NARF, GPX4, SLC25A3, ATP5L, ATP5O, SLC25A1, ATP5I, NDUFS3, NDUFS2, ATP5H, ATP5J, SQRDL, NDUFB11, NDUFB10, LYN, SLC25A5, NDUFC2, NDUFA13, COX4I1, NDUFC1, NDUFA10, NDUFA12, NDUFA11, ACADVL, IMMP1L, PSEN1, UQCRH, TOMM22, MATR3, UQCRB, NDUFB3, NDUFB4, NDUFB5, NDUFB7, NDUFB8, NDUFB9, TIMM10, ATP5G2, ATP5G1, COX7A2L, ATP5G3, NDUFB1, NDUFB2, CSDE1, SCO2, ACSL5, NDUFA4, NDUFA5, NDUFA2, COX7A2, NDUFA3, NDUFA9, NDUFA6, NDUFA7, ATP5F1, LMNA, NDUFA1, SOD2, SDHA, SDHB, PHB2, SDHC, ATP5A1, MGST1	2.93E-22
GO:0005743~mitochondrial inner membrane	5.36E-24	HCCS, OXA1L, NDUFAB1, MPV17, STOML2, COX5A, COX5B, UQCR11, TIMM9, SLC25A28, SLC25A26, CRLS1, POLG, SUCLG1, COX6C, ATP5C1, SLC25A37, ATPIF1, SLC25A39, SURF1, MDH2, HSD17B10, COX7B, CHCHD3, COX7C, KMO, HADHA, HADHB, MTCH2, GSTK1,	3.29E-22

		MTCH1, COX6B1, ATP5J2, COX8A, AK2, PPIF, SLC25A11, USMG5, UCP2, NDUFV1, COX6A1, SPNS1, SLC25A16, CYB5R3, UQCRC2, ATP5D, ATP5E, UQCRC1, ATP5B, TIMM17A, CYC1, TIMM17B, UQCRFS1, UQCRQ, NDUFAF3, NDUFS6, NDUFS5, NDUFS4, PARL, GPX4, SLC25A3, ATP5L, ATP5O, SLC25A1, ATP5I, NDUFS3, NDUFS2, ATP5H, ATP5J, SQRDL, NDUFB11, NDUFB10, LYN, SLC25A5, NDUFC2, NDUFA13, COX4I1, NDUFC1, NDUFA10, NDUFA12, NDUFA11, ACADVL, IMMP1L, PSEN1, UQCRH, TOMM22, UQCRB, NDUFB3, NDUFB4, NDUFB5, NDUFB7, NDUFB8, NDUFB9, TIMM10, ATP5G2, ATP5G1, COX7A2L, ATP5G3, NDUFB1, NDUFB2, CSDE1, SCO2, ACSL5, NDUFA4, NDUFA5, NDUFA2, COX7A2, NDUFA3, NDUFA9, NDUFA6, NDUFA7, ATP5F1, NDUFA1, SOD2, SDHA, SDHB, PHB2, SDHC, ATP5A1, MGST1	
GO:0031967~organelle envelope	3.26E-23	HCCS, EIF6, OXA1L, TSPO, S100A6, XPO6, NDUFAB1, MPV17, EIF5A, STOML2, CBX3, WTAP, COX5A, ANKLE2, COX5B, EPC1, MFF, UQCR11, TIMM9, SLC25A28, SLC25A26, CRLS1, POLG, RAN, SUCLG1, BCL2L11, COX6C, ATP5C1, SLC25A37, ATPIF1, SLC25A39, SURF1, MDH2, MVP, HSD17B10, MCL1, HAX1, AGFG1, CHKB, COX7B, CHCHD3, COX7C, KMO, TAGLN2, HADHA, HADHB, FIS1, TOMM7, MTCH2, TOMM6, RAC2, TOMM5, GSTK1, MTCH1, COX6B1, EMD, ATP5J2, COX8A, AK2, TREX1, SMAD3, NXF1, VDAC2, PPIF, SLC25A11, TXNDC12, USMG5, UCP2, NDUFV1, SEC13,	1.84E-21

		COX6A1, GK, SLC25A16, SPNS1, UQCRC2, ATP5D, RTN4, CYB5R3, MAD1L1, ATP5E, UQCRC1, ATP5B, TIMM17A, TIMM17B, CYC1, UQCRFS1, UQCRQ, NDUFAF3, NDUFS6, TMEM173, NDUFS5, NDUFS4, PARL, GPX4, NARF, CASP8, SLC25A3, ATP5L, SYNJ2, SLC25A1, ATP5O, ATP5I, NDUFS3, NDUFS2, ATP5H, COX17, COX16, ATP5J, SQRDL, NDUFB11, NDUFB10, LYN, SLC25A5, NUP88, CYCS, NDUFC2, COX4I1, NDUFA13, NDUFC1, PI4KB, NDUFA10, NDUFA12, TMEM38B, NDUFA11, IMMP1L, ACADVL, PSEN1, BNIP2, UQCRH, NUCB2, IPO5, TOMM20, TOMM22, KPNA2, MATR3, UQCRB, NXT1, BID, NDUFB3, NDUFB4, FKBP8, NDUFB5, NDUFB7, NDUFB8, NDUFB9, HK2, PML, TIMM10, NUP93, HK1, ATP5G2, ATP5G1, COX7A2L, ATP5G3, NDUFB1, NDUFB2, ANXA7, NUP214, ACSL1, SH3GLB1, ALOX5AP, SHISA5, CSDE1, PAFAH1B1, ACSL3, SCO2, ACSL5, NDUFA4, NDUFA5, SLC2A9, NDUFA2, COX7A2, NDUFA3, NDUFA9, NDUFA6, NDUFA7, ATP5F1, LMNA, RAF1, BAD, NDUFA1, SOD2, SDHA, SDHB, FYN, PHB2, SDHC, BAX, BNIP3L, ANXA11, ATP5A1, MGST1	
GO:0031975~envelope	4.94E-23	HCCS, EIF6, OXA1L, TSPO, S100A6, XPO6, NDUFAB1, MPV17, EIF5A, STOML2, CBX3, WTAP, COX5A, ANKLE2, COX5B, EPC1, MFF, UQCR11, TIMM9, SLC25A28, SLC25A26, CRLS1, POLG, RAN, SUCLG1, BCL2L11, COX6C, ATP5C1, SLC25A37, ATPIF1, SLC25A39, SURF1, MDH2, MVP, HSD17B10, MCL1, HAX1, AGFG1, CHKB, COX7B,	2.57E-21

		CHCHD3, COX7C, KMO, TAGLN2, HADHA, HADHB, FIS1, TOMM7, MTCH2, TOMM6, RAC2, TOMM5, GSTK1, MTCH1, COX6B1, EMD, ATP5J2, COX8A, AK2, TREX1, SMAD3, NXF1, VDAC2, PPIF, SLC25A11, TXNDC12, USMG5, UCP2, NDUFV1, SEC13, COX6A1, GK, SLC25A16, SPNS1, UQCRC2, ATP5D, RTN4, CYB5R3, MAD1L1, ATP5E, UQCRC1, ATP5B, TIMM17A, TIMM17B, CYC1, UQCRFS1, UQCRQ, NDUFAF3, NDUFS6, TMEM173, NDUFS5, NDUFS4, PARL, GPX4, NARF, CASP8, SLC25A3, ATP5L, SYNJ2, SLC25A1, ATP5O, ATP5I, NDUFS3, NDUFS2, ATP5H, COX17, COX16, ATP5J, SQRDL, NDUFB11, NDUFB10, LYN, SLC25A5, NUP88, CYCS, NDUFC2, COX4I1, NDUFA13, NDUFC1, PI4KB, NDUFA10, NDUFA12, TMEM38B, NDUFA11, IMMP1L, ACADVL, PSEN1, BNIP2, UQCRH, NUCB2, IPO5, TOMM20, TOMM22, KPNA2, MATR3, UQCRB, NXT1, BID, NDUFB3, NDUFB4, FKBP8, NDUFB5, NDUFB7, NDUFB8, NDUFB9, HK2, PML, TIMM10, NUP93, HK1, ATP5G2, ATP5G1, COX7A2L, ATP5G3, NDUFB1, NDUFB2, ANXA7, NUP214, ACSL1, SH3GLB1, ALOX5AP, SHISA5, CSDE1, PAFAH1B1, ACSL3, SCO2, ACSL5, NDUFA4, NDUFA5, SLC2A9, NDUFA2, COX7A2, NDUFA3, NDUFA9, NDUFA6, NDUFA7, ATP5F1, LMNA, RAF1, BAD, NDUFA1, SOD2, SDHA, SDHB, FYN, PHB2, SDHC, BAX, BNIP3L, ANXA11, ATP5A1, MGST1	
GO:0044455~mitochondrial membrane part	2.17E-22	ATP5D, UQCRC2, ATP5E, OXA1L, UQCRC1, TIMM17A, ATP5B, TIMM17B, NDUFAB1,	1.05E-20

		UQCRFS1, NDUFS6, NDUFS5, NDUFS4, TIMM9, SYNJ2, ATP5L, ATP5O, NDUFS3, ATP5I, ATP5H, NDUFS2, ATP5J, NDUFB10, LYN, NDUFC2, NDUFA13, NDUFC1, NDUFA10, UQCRH, TOMM20, ATP5C1, ATPIF1, TOMM22, SURF1, UQCRB, NDUFB3, NDUFB4, NDUFB5, NDUFB7, NDUFB8, NDUFB9, COX7B, TIMM10, ATP5G2, COX7A2L, ATP5G1, ATP5G3, NDUFB1, NDUFB2, FIS1, TOMM7, TOMM5, NDUFA4, NDUFA5, ATP5J2, NDUFA2, NDUFA3, COX7A2, NDUFA9, NDUFA6, NDUFA7, ATP5F1, NDUFA1, SDHA, NDUFV1, COX6A1, ATP5A1	
GO:0005739~mitochondrion	3.79E-21	HCCS, OXA1L, PNKD, MPV17, STOML2, C14ORF2, MRPL38, PDHA1, MRPL33, MRPL53, MRPL52, MRPL51, CRLS1, POLG, SUCLG1, LYRM4, KIAA0141, BCL2L13, BCL2L11, GLUL, ATP5C1, MRPL47, ATPIF1, MRPL48, SURF1, MDH2, SIVA1, HSD17B10, TXN2, CHKB, FARS2, CHCHD3, CHCHD2, OAS1, SDHAF2, HADHA, HADHB, MRPL54, COX6B1, PEMT, MRPL55, DIABLO, SUPV3L1, TAOK3, GARS, VDAC2, PTPN11, CHCHD10, HEBP2, COX6A1, GK, SPNS1, ATP5D, ATP5E, SPG7, TIMM17A, ATP5B, TIMM17B, QARS, PTEN, CKB, PARL, GPX4, SLC25A3, SYNJ2, ATP5L, SLC25A1, ATP5O, ATP5I, ATP5H, COX17, COX16, ATP5J, FUNDC2, CLN3, NDUFB11, NDUFB10, LYN, SLC25A5, NDUFA13, DGUOK, DECR1, PI4KB, ECSIT, NDUFA10, NDUFA12, NDUFA11, PSEN1, CLIC4, TXN, TOMM20, TOMM22, SLC27A3, TUFM, BID, YWHAZ, ECH1, HK2, HK1, COX7A2L, MRPL20,	1.71E-19

		ACSL1, MRPL14, PPP2CA, CSDE1, MRPL18, KIAA0391, ACSL3, ETFB, ETFA, ACSL5, COX7A2, HCLS1, ILF3, SOD1, YWHAE, SOD2, SDHA, SDHB, RAB32, PRELID1, MRPL23, MRPL21, MRPL28, FYN, ATP6V1E1, SDHC, ARAF, BNIP3L, ATP5A1, MRPS36, TSPO, GRPEL1, CMC1, MRPS33, NDUFAB1, ROMO1, PMAIP1, COX5A, COX5B, MTHFD1L, MFF, UQCR11, IDH3G, TIMM9, LRRC59, SLC25A28, PRKACA, SLC25A26, PABPN1, TAZ, TMEM126A, PISD, COX6C, ISCU, MRPS18C, PSMA6, MRPS18A, CLPP, SLC25A37, SLC25A39, MRPS15, MCL1, HAX1, CLU, COX7B, MRPS11, COX7C, KMO, CTSA, KARS, FIS1, TOMM7, MTCH2, TOMM6, TOMM5, C12ORF10, GSTK1, MTCH1, HSPE1, AP2M1, ATP5J2, MRPS28, FIBP, MMADHC, MRPS24, COX8A, AK2, IDH3B, PPIF, TXNDC12, SLC25A11, USMG5, PSMC4, UCP2, NDUFV1, GLS, ALDH2, COQ10B, SLC25A16, FPGS, IFI6, DUT, CYB5R3, UQCRC2, ACOX1, KYNU, UQCRC1, GM2A, CYC1, HINT2, PRDX5, UQCRFS1, PRDX1, UQCRQ, NDUFAF2, NDUFAF3, ACOT9, NDUFS6, TMEM173, NDUFS5, ALAS1, NDUFS4, CASP8, NDUFS3, NDUFS2, SQRDL, PPP2R1A, ATPAF2, SSBP1, CYCS, NDUFC2, COX4I1, MRPS6, NDUFC1, LAP3, IMMP1L, ACADVL, C1QBP, UQCRH, GRN, TXNRD2, UQCRB, NDUFB3, NDUFB4, PGS1, FKBP8, NDUFB5, NDUFB7, NDUFB8, NDUFB9, ETHE1, TIMM10, ATP5G2, ATP5G1, NR3C1, ATP5G3, NDUFB1, NDUFB2, TSC22D3, SH3GLB1, PTS, LACTB, SCO2,	

		HSPA9, NDUFA4, NDUFA5, NDUFA2, NDUFA3, PSAP, NDUFA9, NDUFA6, NDUFA7, ATP5F1, RAF1, BAD, NDUFA1, PARK7, CYBA, CYBB, BAX, PHB2, MGST1	
GO:0015935~small ribosomal subunit	4.91E-21	MRPS36, MRPS33, MRPS15, MRPS11, RPS15A, RPS2, RPS3, RPS25, RPS27, RPS28, RPS29, RPS3A, NPM1, FAU, RPS20, RPS21, RPS23, RPS27A, RPS24, FTL, RPSA, MRPS28, MRPS24, RPS9, MRPS6, HBA2, HBA1, RPS6, RPS4X, RPS5, RPS8, RPS7, MRPS18C, RPS19, RPS16, MRPS18A, RPS14, RPS15, UBC, RPS12, RPS13, RPS10, RPS11, UBB, UBA52	2.08E-19
GO:0043233~organelle lumen	7.61E-21	MEF2C, XRCC5, PTGS2, XRCC6, WTAP, ISG20, CTNNB1, WDR74, EPC1, PQBP1, RPL11, PDHA1, LUC7L3, MCRS1, NCF2, PTBP1, SKP1, TOX4, PPP1CA, RPS19, RPS14, RPS15, FLII, RPS13, MYO18A, ACAA1, CPSF3L, NFKBIE, NHP2L1, ZCRB1, XAB2, HADHA, HADHB, RPS25, ISY1, PRPF40A, DHX9, GARS, RPS9, NXF1, VDAC2, RPS6, FXR1, RPS7, POP4, ZNF385A, BACH1, PDIA3, TSG101, EZH2, PDIA6, QARS, RPS19BP1, RTN3, HSF1, CREG1, QKI, SERPINA1, STX5, EXOSC8, NDUFA13, EXOSC1, NDUFA10, LRPAP1, C19ORF33, TAF10, EIF4A3, SIL1, TUFM, CREM, PML, RPL35, RPL36, ZNF655, SF3B1, SRRT, WDR13, USP36, SUPT4H1, ETFB, PHLDA1, NSA2, ETFA, CES1, TRIM28, LMNA, TRIM25, MEF2D, DUSP3, RPS6KA1, RPL23, SFPQ, ZNF259, TCEB2, PHF5A, TCEB1, MPHOSPH6, NCLN, DUSP6, EIF6, NCBP1, SNRPD3, RBM4, SNRPD1, RBM5, NDUFAB1, RPS6KB2, SNRPD2, SRP19, TGFB1,	3.03E-19

		CTNNBL1, KDM1A, IDH3G, SMARCD3, DDX24, LRRC59, IMP4, RPS27A, CIB1, PABPN1, STK24, RELA, MTA2, FMR1, HNRNPA2B1, ACTN1, JUNB, KDM2A, EDF1, UBA52, HMGB2, ZNF330, TIMP1, CXXC1, HNRNPL, HNRNPM, HNRNPK, DDX3X, RPL9, HNRNPF, RPL3, TAF9, HSPE1, RPL5, SDF4, TFDP1, TAF7, AKAP8L, IDH3B, NR4A1, HDAC5, POLD4, ATF5, DDX56, ATF4, CDKN1A, HDAC3, HNRNPH2, ATF3, HDAC1, VCP, EAF1, HNRNPH1, HDAC9, HDAC6, NBN, FOSL2, E2F4, RPS2, ZNF207, ALAS1, PLRG1, DKC1, RPS3A, GTF2A2, CASP8, INO80B, ATPAF2, SSBP1, CYCS, CCNL1, PRPF3, IFI16, NCL, HMGA1, ACADVL, ADRB2, IPO5, UBC, CPSF6, UBB, CPSF1, ETHE1, PF4, NR3C1, IVNS1ABP, PRPF8, GTF3C6, GTF3C5, CD4, HSPA5, CHD4, HSPA9, CEBPB, PSAP, ATP5F1, FBL, HSP90B1, S100A4, S100A9, CCT2, PDLIM1, DNAJB11, U2AF1, SUPT5H, MRPL52, GTPBP4, MRPL51, ANAPC5, RAN, POLG, SUCLG1, POLB, PPBP, VEGFA, ATP5C1, MRPL47, LRCH4, MDH2, FUS, SIVA1, TXN2, FARS2, CALR, HNRNPA3, TAL1, FRG1, MRPL55, SUPV3L1, SUB1, SRA1, SMAD3, PMF1, DDX5, HNRNPA1, U2AF1L4, NOP10, S100A12, OASL, CCT5, NOP16, JAZF1, RBPJ, TCF12, KLF4, ADAR, ENY2, ATP5D, ATP5E, ATP5B, NFKB1, ANPEP, NFKB2, SENP5, NARF, TOP2B, DEDD2, POLR1D, CCNH, SLC25A5, SF1, RBBP7, ECSIT, MBD2, RBBP6, GTF2B, SENP3, MED4, CHMP1A, RBM39, POLR2G, RBM34, POLR2F, POLR2E,	

		POLR2L, POLR2I, SHFM1, YPEL3, KIN, MRPL20, STAT6, PRPF19, STAT4, SET, CIR1, SQSTM1, MRPL18, THAP2, FAM32A, THBS1, ETV6, RBM22, BCAS2, RBM23, RRP12, YWHAB, ILF3, SOD1, STAT1, STAT3, SOD2, MRPL23, PAPOLA, ILF2, MRPL28, CALM3, NOP58, NOP56, ATP5A1, CALM2, CALM1, MORF4L1, MRPS36, GRPEL1, MRPS33, MORF4L2, KLHL7, WIBG, SRRM2, TARDBP, SEP15, GNL2, AKIRIN2, MAGOH, ERP29, PPP1CB, MRPS18C, MRPS18A, CLPP, SNRPB, CSTB, SLU7, SNRPC, PAF1, SNRPF, SNRPE, SNRPG, MRPS15, MCL1, MVD, CLU, MRPS11, ANAPC10, ANAPC11, KARS, DAZAP1, TRIM69, GSTK1, EIF3E, NPM1, EIF3L, UBE2D1, SRGN, P4HB, MRPS28, MAFB, MRPS24, SAP18, SNW1, RNPS1, KAT5, INTS10, MED13L, FOXP1, PPIF, TXNDC12, PSMC5, PPIB, GLS, ALDH2, CIRBP, ASNA1, UBE2E1, PRKAG2, MKI67IP, OS9, FUBP1, FOS, MSN, PSMD8, ELP2, SP100, NOL7, UBE2I, PCGF1, C1QBP, CFL1, MDM2, PSME4, KPNA2, MATR3, GLTSCR2, UBE2Z, UTP6, MGMT, TCF7L2, EXOSC10, NUDT21, RSL24D1, APEX1, TINF2, ERCC1, ACTB, NDUFA9, SPARC, GPS2, ATXN1, SON, ATXN3, ANXA11, PSPC1, TRPC4AP, SH3D19	
GO:0022627~cytosolic small ribosomal subunit	8.58E-21	RPS15A, RPS2, RPS3, RPS25, RPS27, RPS28, RPS29, RPS3A, FAU, RPS20, RPS21, RPS27A, RPS23, FTL, RPS24, RPSA, RPS9, HBA2, HBA1, RPS6, RPS4X, RPS5, RPS8, RPS7, RPS19, RPS16, RPS14, RPS15, UBC, RPS12, RPS13, RPS10, RPS11, UBB, UBA52	3.22E-19

GO:0031974~membrane-enclosed lumen	1.08E-20	MEF2C, XRCC5, PTGS2, XRCC6, WTAP, ISG20, CTNNB1, WDR74, EPC1, PQBP1, RPL11, PDHA1, LUC7L3, MCRS1, NCF2, PTBP1, SKP1, TOX4, PPP1CA, RPS19, RPS14, RPS15, FLII, RPS13, MYO18A, ACAA1, CPSF3L, NFKBIE, NHP2L1, ZCRB1, XAB2, HADHA, HADHB, RPS25, ISY1, PRPF40A, DHX9, GARS, RPS9, NXF1, VDAC2, RPS6, FXR1, RPS7, POP4, ZNF385A, BACH1, PDIA3, TSG101, EZH2, PDIA6, QARS, RPS19BP1, RTN3, HSF1, CREG1, QKI, SERPINA1, STX5, EXOSC8, LYN, NDUFA13, EXOSC1, NDUFA10, LRPAP1, C19ORF33, TAF10, EIF4A3, SIL1, TUFM, CREM, PML, RPL35, RPL36, ZNF655, SF3B1, SRRT, WDR13, USP36, SUPT4H1, ETFB, PHLDA1, NSA2, ETFA, CES1, TRIM28, LMNA, TRIM25, MEF2D, DUSP3, RPS6KA1, RPL23, SFPQ, ZNF259, TCEB2, PHF5A, TCEB1, MPHOSPH6, NCLN, DUSP6, EIF6, NCBP1, SNRPD3, RBM4, SNRPD1, RBM5, NDUFAB1, RPS6KB2, SNRPD2, SRP19, TGFB1, CTNNBL1, KDM1A, IDH3G, SMARCD3, TIMM9, DDX24, LRRC59, IMP4, RPS27A, CIB1, PABPN1, STK24, RELA, MTA2, FMR1, HNRNPA2B1, ACTN1, JUNB, KDM2A, EDF1, UBA52, HMGB2, ZNF330, TIMP1, CXXC1, HNRNPL, HNRNPM, HNRNPK, DDX3X, RPL9, HNRNPF, RPL3, TAF9, HSPE1, RPL5, SDF4, TFDP1, TAF7, AKAP8L, IDH3B, NR4A1, HDAC5, POLD4, ATF5, HDAC3, DDX56, ATF4, CDKN1A, HNRNPH2, ATF3, HDAC1, VCP, EAF1, HNRNPH1, HDAC9, HDAC6, NBN, FOSL2, E2F4,	3.84E-19

		RPS2, ZNF207, ALAS1, PLRG1, DKC1, RPS3A, GTF2A2, CASP8, INO80B, ATPAF2, SSBP1, CYCS, CCNL1, PRPF3, IFI16, NCL, HMGA1, ACADVL, ADRB2, IPO5, UBC, CPSF6, UBB, CPSF1, ETHE1, TIMM10, PF4, NR3C1, IVNS1ABP, PRPF8, GTF3C6, GTF3C5, CD4, HSPA5, CHD4, HSPA9, CEBPB, PSAP, ATP5F1, FBL, HSP90B1, S100A4, S100A9, CCT2, PDLIM1, DNAJB11, U2AF1, SUPT5H, MRPL52, GTPBP4, MRPL51, ANAPC5, RAN, POLG, SUCLG1, POLB, PPBP, VEGFA, ATP5C1, MRPL47, LRCH4, MDH2, FUS, SIVA1, TXN2, FARS2, CALR, HNRNPA3, TAL1, FRG1, COX6B1, MRPL55, SUPV3L1, SUB1, SRA1, SMAD3, PMF1, DDX5, HNRNPA1, U2AF1L4, NOP10, S100A12, OASL, CCT5, NOP16, JAZF1, RBPJ, TCF12, KLF4, ADAR, ENY2, ATP5D, ATP5E, ATP5B, NFKB1, ANPEP, NFKB2, SENP5, NARF, TOP2B, COX17, DEDD2, POLR1D, CCNH, SLC25A5, SF1, RBBP7, ECSIT, MBD2, RBBP6, GTF2B, SENP3, MED4, CHMP1A, RBM39, POLR2G, RBM34, POLR2F, POLR2E, POLR2L, POLR2I, SHFM1, YPEL3, KIN, MRPL20, STAT6, PRPF19, STAT4, SET, CIR1, SQSTM1, MRPL18, THAP2, FAM32A, THBS1, ETV6, RBM22, BCAS2, RBM23, RRP12, YWHAB, ILF3, SOD1, STAT1, STAT3, SOD2, MRPL23, PAPOLA, ILF2, MRPL28, CALM3, NOP58, NOP56, ATP5A1, CALM2, CALM1, MORF4L1, MRPS36, GRPEL1, MRPS33, MORF4L2, KLHL7, WIBG, SRRM2, TARDBP, SEP15, GNL2, AKIRIN2, MAGOH, ERP29, PPP1CB, MRPS18C, MRPS18A, CLPP,	

		SNRPB, CSTB, SLU7, SNRPC, PAF1, SNRPF, SNRPE, SNRPG, MRPS15, MCL1, MVD, CLU, MRPS11, ANAPC10, ANAPC11, KARS, DAZAP1, TRIM69, GSTK1, EIF3E, NPM1, EIF3L, UBE2D1, SRGN, P4HB, MRPS28, MAFB, MRPS24, SAP18, AK2, SNW1, RNPS1, KAT5, INTS10, MED13L, FOXP1, PPIF, TXNDC12, PSMC5, PPIB, GLS, ALDH2, CIRBP, ASNA1, UBE2E1, PRKAG2, MKI67IP, OS9, FUBP1, FOS, MSN, PSMD8, ELP2, SP100, NOL7, UBE2I, PCGF1, C1QBP, CFL1, MDM2, PSME4, KPNA2, MATR3, GLTSCR2, UBE2Z, UTP6, MGMT, TCF7L2, EXOSC10, NUDT21, RSL24D1, APEX1, TINF2, ERCC1, ACTB, NDUFA9, SPARC, GPS2, ATXN1, SON, ATXN3, ANXA11, PSPC1, TRPC4AP, SH3D19	
GO:0070469~respiratory chain	1.64E-20	UQCRC2, NDUFB3, NDUFB4, OXA1L, NDUFB5, UQCRC1, NDUFB7, NDUFB8, NDUFB9, CYC1, COX7B, NDUFAB1, COX7A2L, UQCRFS1, UQCRQ, NDUFB1, NDUFB2, NDUFS6, NDUFS5, UQCR11, NDUFS4, NDUFS3, NDUFS2, NDUFA4, NDUFA5, NDUFB11, NDUFA2, NDUFA3, COX7A2, NDUFB10, NDUFA9, NDUFA6, NDUFA7, CYCS, NDUFC2, NDUFA13, NDUFC1, NDUFA10, NDUFA1, NDUFA12, NDUFA11, SDHA, UQCRH, NDUFV1, SDHC, COX6A1, SURF1, UQCRB	5.55E-19
GO:0070013~intracellular organelle lumen	3.29E-20	MEF2C, XRCC5, PTGS2, XRCC6, WTAP, ISG20, CTNNB1, WDR74, EPC1, PQBP1, RPL11, PDHA1, LUC7L3, MCRS1, NCF2, PTBP1, SKP1, TOX4, PPP1CA, RPS19, RPS14, RPS15, FLII, RPS13, MYO18A, ACAA1, CPSF3L, NFKBIE, NHP2L1, ZCRB1, XAB2, HADHA, HADHB,	

		RPS25, ISY1, PRPF40A, DHX9, GARS, RPS9, NXF1, VDAC2, RPS6, FXR1, RPS7, POP4, ZNF385A, BACH1, PDIA3, TSG101, EZH2, PDIA6, QARS, RPS19BP1, RTN3, HSF1, CREG1, QKI, STX5, EXOSC8, NDUFA13, EXOSC1, NDUFA10, LRPAP1, C19ORF33, TAF10, EIF4A3, SIL1, TUFM, CREM, PML, RPL35, RPL36, ZNF655, SF3B1, SRRT, WDR13, USP36, SUPT4H1, ETFB, PHLDA1, NSA2, ETFA, CES1, TRIM28, LMNA, TRIM25, MEF2D, DUSP3, RPS6KA1, RPL23, SFPQ, ZNF259, TCEB2, PHF5A, TCEB1, MPHOSPH6, NCLN, DUSP6, EIF6, NCBP1, SNRPD3, RBM4, SNRPD1, RBM5, NDUFAB1, RPS6KB2, SNRPD2, SRP19, TGFB1, CTNNBL1, KDM1A, IDH3G, SMARCD3, DDX24, LRRC59, IMP4, RPS27A, CIB1, PABPN1, STK24, RELA, MTA2, FMR1, HNRNPA2B1, ACTN1, JUNB, KDM2A, EDF1, UBA52, HMGB2, ZNF330, CXXC1, HNRNPL, HNRNPM, HNRNPK, DDX3X, RPL9, HNRNPF, RPL3, TAF9, HSPE1, RPL5, SDF4, TFDP1, TAF7, AKAP8L, IDH3B, NR4A1, HDAC5, POLD4, ATF5, DDX56, ATF4, CDKN1A, HDAC3, HNRNPH2, ATF3, HDAC1, VCP, EAF1, HDAC9, HNRNPH1, HDAC6, NBN, FOSL2, E2F4, RPS2, ZNF207, ALAS1, PLRG1, DKC1, RPS3A, GTF2A2, CASP8, INO80B, ATPAF2, SSBP1, CYCS, CCNL1, PRPF3, IFI16, NCL, HMGA1, ACADVL, ADRB2, IPO5, UBC, CPSF6, UBB, CPSF1, ETHE1, NR3C1, IVNS1ABP, PRPF8, GTF3C6, GTF3C5, CD4, HSPA5, CHD4, HSPA9, CEBPB, PSAP, ATP5F1, FBL, HSP90B1, S100A4, S100A9, CCT2,	

		PDLIM1, DNAJB11, U2AF1, SUPT5H, MRPL52, GTPBP4, MRPL51, ANAPC5, RAN, POLG, SUCLG1, POLB, ATP5C1, MRPL47, LRCH4, MDH2, SIVA1, FUS, TXN2, FARS2, CALR, HNRNPA3, TAL1, FRG1, MRPL55, SUPV3L1, SUB1, SRA1, SMAD3, PMF1, DDX5, HNRNPA1, U2AF1L4, NOP10, S100A12, OASL, CCT5, NOP16, JAZF1, RBPJ, TCF12, KLF4, ADAR, ENY2, ATP5D, ATP5E, ATP5B, NFKB1, NFKB2, SENP5, NARF, TOP2B, DEDD2, POLR1D, CCNH, SLC25A5, SF1, ECSIT, MBD2, RBBP7, RBBP6, GTF2B, SENP3, MED4, CHMP1A, RBM39, POLR2G, RBM34, POLR2F, POLR2E, POLR2L, POLR2I, SHFM1, YPEL3, KIN, MRPL20, STAT6, PRPF19, STAT4, SET, CIR1, SQSTM1, MRPL18, THAP2, FAM32A, ETV6, RBM22, BCAS2, RBM23, RRP12, YWHAB, ILF3, SOD1, STAT1, STAT3, SOD2, MRPL23, PAPOLA, ILF2, MRPL28, CALM3, NOP58, NOP56, ATP5A1, CALM2, CALM1, MORF4L1, MRPS36, GRPEL1, MRPS33, MORF4L2, KLHL7, WIBG, SRRM2, TARDBP, SEP15, GNL2, AKIRIN2, MAGOH, ERP29, PPP1CB, MRPS18C, MRPS18A, CLPP, SNRPB, CSTB, SLU7, SNRPC, PAF1, SNRPF, SNRPE, SNRPG, MRPS15, MCL1, MVD, MRPS11, ANAPC10, ANAPC11, KARS, DAZAP1, TRIM69, GSTK1, EIF3E, NPM1, EIF3L, UBE2D1, P4HB, MRPS28, MAFB, MRPS24, SAP18, SNW1, RNPS1, INTS10, MED13L, KAT5, FOXP1, PPIF, TXNDC12, PSMC5, PPIB, GLS, ALDH2, CIRBP, ASNA1, UBE2E1, PRKAG2, MKI67IP, OS9, FUBP1,	

		FOS, MSN, PSMD8, ELP2, SP100, NOL7, UBE2I, PCGF1, C1QBP, CFL1, MDM2, PSME4, KPNA2, MATR3, GLTSCR2, UBE2Z, MGMT, UTP6, TCF7L2, EXOSC10, NUDT21, RSL24D1, APEX1, TINF2, ERCC1, ACTB, NDUFA9, GPS2, ATXN1, SON, ATXN3, ANXA11, PSPC1, TRPC4AP, SH3D19	
GO:0031090~organelle membrane	5.60E-20	HCCS, OXA1L, VAPA, MPV17, STOML2, WTAP, B2M, EPC1, CRLS1, POLG, SUCLG1, VTI1B, BCL2L11, MGAT1, CD36, ATP2C1, ATP5C1, ARL8A, ATPIF1, ARL8B, EXT1, SURF1, TRAPPC3, MDH2, ITGA2B, HSD17B10, MFNG, CHKB, CHCHD3, RER1, TAGLN2, HADHA, HADHB, SLC11A2, SLC11A1, SERINC1, COX6B1, PEMT, TMED10, STX10, TRAM1, EMD, LFNG, DENND1A, SMAD3, VDAC2, CD63, COX6A1, GK, SPNS1, RTN4, ATP5D, ATP5E, LDLR, ORMDL1, TIMM17A, ATP5B, AP2S1, TIMM17B, BSCL2, ANPEP, ACTR3, AP1S2, PARL, MAP1LC3B, SRPR, GPX4, NARF, COPB1, SLC25A3, SYNJ2, ATP5L, SLC25A1, ATP5O, ATP5I, ATP5H, COX16, ATP5J, STX5, CLN3, NDUFB11, NDUFB10, LYN, SLC25A5, NDUFA13, PI4KB, NDUFA10, WAS, NDUFA12, NDUFA11, NCK2, PSEN1, TOMM20, LRMP, GNAS, TOMM22, BID, PML, HK2, HK1, FKBP1A, ATP6V1G1, COX7A2L, ACSL1, ALOX5AP, PEX16, MPDU1, CSDE1, QSOX1, ACSL3, PHLDA1, ACSL5, COX7A2, LMNA, GABARAP, GJB2, SOD2, SDHA, SDHB, AP2A1, SDHC, DPM1, ATP5A1, SSR4, SLC15A3, SSR2, SSR3, GNPTG, EIF6, TSPO, AP1G2, NDUFAB1, CBX3, COX5A, COX5B, FDFT1,	1.72E-18

		ST3GAL1, MFF, ST3GAL3, UQCR11, TIMM9, ST3GAL6, RPN1, SLC25A28, RPN2, ASPH, SLC25A26, SCAMP2, SPTLC2, HLA-A, CORO7, PNPLA6, COX6C, BCAP31, PNPLA8, SEC61B, VAMP8, SLC25A37, SLC25A39, SEC61G, DERL2, STX8, DERL1, MCL1, HAX1, GRB2, COX7B, COPZ1, COX7C, KMO, FIS1, MIA3, ECE1, LAPTM5, TOMM7, MTCH2, TOMM6, TOMM5, GSTK1, SNF8, MTCH1, SEC61A1, AP2M1, GABARAPL2, RILP, ATP5J2, HERPUD1, SEC11A, COX8A, AK2, PPIF, SLC25A11, LAMP2, CORO1A, USMG5, UCP2, NDUFV1, SPCS3, SPCS1, SLC25A16, CYB5R3, UQCRC2, ACOX1, CLTA, UQCRC1, CLTB, CYC1, UQCRFS1, UQCRQ, OS9, NDUFAF3, NDUFS6, TMEM173, NDUFS5, NDUFS4, RNF103, CASP8, NECAP2, VPS16, NDUFS3, NDUFS2, SQRDL, NDUFC2, COX4I1, PIGT, ATP6V1H, KTN1, NDUFC1, KRTCAP2, ATP6V1F, TMEM38B, IMMP1L, ACADVL, UQCRH, SBF2, NUCB2, MDM2, CUX1, MATR3, COPE, UQCRB, NDUFB3, NDUFB4, OSTC, FKBP8, AP1M1, NDUFB5, NDUFB7, NDUFB8, NDUFB9, TIMM10, ATP5G2, ATP5G1, ATP5G3, NDUFB1, NDUFB2, CD9, TMED2, SH3GLB1, SHISA5, DAD1, PAFAH1B1, CD4, HSPA5, SCO2, NDUFA4, PLP2, NDUFA5, NDUFA2, NDUFA3, NDUFA9, NDUFA6, NDUFA7, ATP5F1, RAF1, BAD, NDUFA1, HSP90B1, BAX, PHB2, MGST1	
GO:0022625~cytosolic large ribosomal subunit	2.49E-19	RPL18, RPL19, RPL14, RPL27A, RPL35, RPLP2, RPL36, RPL37, RPL38, RPL30, RPL32, RPL7, RPL6, RPL31, RPL34, RPLP0, RPL8, RPLP1, RPL3, RPL10,	7.31E-18

		RPL5, RPL11, RPL4, RPL7A, RPL26, RPL23A, RPL24, RPL28, RPL29, RPL41, RPL18A, RPL22	
GO:0005681~spliceosome	3.95E-17	RALY, SNRPD3, U2AF2, CWC15, RBM5, SNRPD1, SNRPD2, SART1, DHX38, PLRG1, SRRM2, U2AF1, SNRPA1, EFTUD2, MAGOH, HNRNPA2B1, SF1, PRPF3, HNRNPU, EIF4A3, SNRPB, SNRPA, SLU7, SNRNP40, SNRPF, SNRPE, PRPF38A, SNRPG, NHP2L1, STRAP, SNRPB2, ZCRB1, IVNS1ABP, SF3B5, XAB2, SF3B2, PRPF19, HNRNPA3, HNRNPM, SF3B1, HNRNPK, FRG1, HNRNPF, PRPF8, DHX15, ISY1, SNRNP70, PABPC1, HNRNPC, DDX41, RBM22, BCAS2, SNW1, DDX5, HNRNPA1, RBMX, U2AF1L4, SYF2, PHF5A, HNRNPH1, TXNL4A, RBM17	1.11E-15
GO:0005746~mitochondrial respiratory chain	1.19E-16	UQCRC2, NDUFB3, NDUFB4, OXA1L, NDUFB5, UQCRC1, NDUFB7, NDUFB8, NDUFB9, COX7B, NDUFAB1, COX7A2L, UQCRFS1, NDUFB1, NDUFB2, NDUFS6, NDUFS5, NDUFS4, NDUFS3, NDUFS2, NDUFA4, NDUFA5, NDUFA2, NDUFA3, NDUFB10, COX7A2, NDUFA9, NDUFA6, NDUFA7, NDUFC2, NDUFA13, NDUFC1, NDUFA10, NDUFA1, SDHA, UQCRH, NDUFV1, COX6A1, SURF1, UQCRB	3.00E-15
GO:0015934~large ribosomal subunit	1.33E-16	RPL18, RPL17, RPL19, RPL14, RPL27A, RPL35, RPLP2, RPL36, RPL37, RPL38, MRPL20, RPL30, RPL32, RPL7, RPL31, RPL6, RPL34, RPLP0, RPLP1, RPL8, NPM1, RPL3, RPL10, MRPL55, RPL5, RPL11, RPL4, RPL7A, MRPL52, MRPL51, MRPS24, RPL26, RPL23A, RPL24, RPL28, RPL29, MRPL23, RPL18A, RPL41, RPL22, RPL13A	2.89E-15
GO:0043228~non-membrane-bounded organelle	1.46E-16	XRCC5, RPL18, RPL17, RPL19, RPL14, RPL13, XRCC6, RPL15,	2.78E-15

		STOML2, RPLP2, H1FX, RPL22L1, WTAP, KIFC3, CITED2, CTNNB1, ACTG1, WDR74, HIST1H2BK, RPLP0, ILK, RPLP1, PQBP1, FAU, RPL10, RPL11, RPL12, H1F0, MCRS1, NCF2, PTBP1, MECP2, TOX4, MYH9, CTNNA1, UXT, RPS19, RPS16, SPAG5, RPS14, RPS15, RPS12, RPS13, FLII, ARL8A, RPS10, ARL8B, RPS11, MYO18A, NHP2L1, NFKBIE, HADHA, HADHB, RPS25, PEA15, PFN1, RPS27, RPS28, RPS29, ISY1, RPS20, RPS21, CDC42EP3, RPS23, RPS24, DHX9, RPSA, RPS9, HBA2, HBA1, RPS6, VDAC2, RPS5, GAS7, RPS8, FXR1, RPS7, H3F3B, POP4, H3F3C, ZNF385A, BACH1, TSG101, RPS19BP1, RTN3, OFD1, HSF1, DYNLL1, MAP1LC3B, QKI, SYNJ2, MICAL1, PARVG, STX5, EXOSC8, ARHGEF2, HIST1H1C, LYN, EXOSC1, WAS, FLNA, ELMO1, C19ORF33, EPB41L3, RPL41, CLIC4, PFDN5, TMSB4X, PARVB, TUFM, HIST1H2AC, FHL3, PML, RPL35, RPL36, ABI1, RPL37, ZNF655, RPL38, RPL30, EZR, RPL32, RPL31, PTK2B, RPL34, PPP2CA, EXOC4, WDR13, USP36, TRIP10, PHLDA1, NSA2, HIST1H2BD, TRIM28, RPL26, LMNA, RPL27, TRIM25, RPL24, RPL28, RPL29, LSP1, MEF2D, RPL23, RPL22, PTP4A1, ZNF259, MPHOSPH6, SSNA1, NCLN, DNM2, EIF6, MTSS1, PRC1, SNRPD3, CAPZA1, RBM4, RBM5, RPS6KB2, RPS27L, SRP19, CTNNBL1, VCL, IDH3G, LRRC59, DDX24, IMP4, RPS27A, PABPN1, RPL35A, TWF2, MTA2, FMR1, HNRNPA2B1, ACTN1, PDE4D, TMSB10, JUNB, ARPC1A, ARPC1B, IGBP1, PDCD6IP,	

		TNFAIP3, UBA52, SMARCA4, HMGB2, LITAF, ADORA2A, CDC42SE1, C14ORF166, ARPC4, ARPC5, ZNF330, HNRNPM, DOCK2, HNRNPK, RPL7, ARPC3, ARPC2, RPL6, HNRNPF, RPL9, JUND, RPL8, RPL3, HNRNPD, RPL5, RPL7A, RPL4, RPL10A, ARHGDIB, UBXN11, TAF7, MYO1G, AKAP8L, MYO1F, MYL12B, RPL23A, SMEK1, MYL12A, MPRIP, P2RX4, HDAC3, DDX56, HNRNPH2, ATF3, VCP, HDAC1, CDC42SE2, SEC13, RPL37A, HNRNPH1, HDAC6, MAD1L1, NBN, FOSL2, HINT1, RHOQ, RPS2, RHOU, RPS3, ZNF207, DKC1, RPS3A, CASP8, RHOA, LRRFIP1, VPS16, RHOF, INO80B, PPP2R1A, SSBP1, IFI16, SLC9A3R1, RPS4X, NCL, CDK5, HMGA1, VASP, ACADVL, ADRB2, ATG4D, TBCB, ATG4B, TBCA, TBCD, FRMD4B, IPO5, UBC, UBB, MAPRE1, FHOD1, LCP1, HAUS4, HAUS2, VIM, RPS15A, CALCOCO2, NR3C1, IVNS1ABP, SEC62, CHD2, PAFAH1B1, SH2B2, CHD4, HSPA9, ANXA1, DRG1, COTL1, FBL, ID2, RPL13A, S100A4, FAM110A, HMGN3, VAPA, PDLIM7, PDLIM5, S100A9, SNCA, CCT2, PDLIM1, CCT3, KIF13A, H2AFV, IFT20, HOMER3, H2AFZ, H2AFY, MRPL38, MRPL33, FTL, CLNS1A, MRPL53, MRPL52, DBNL, GTPBP4, MRPL51, RAN, POLG, UBR4, POLB, H2AFJ, MAP1LC3B2, DCTN6, DCTN3, DCTN2, SUV420H2, REC8, BAZ1A, CNTROB, TUBA4A, MRPL47, BIN3, PDE4DIP, MRPL48, BIN1, FUS, ALDOA, TXN2, STK17B, MYO9B, CAPZB, BANF1, HNRNPA3, TAL1, FRG1, CEP170, MRPL54,	

		MRPL55, SUPV3L1, EMD, IKZF1, SUB1, PMF1, DDX5, NOP10, HNRNPA1, S100A12, CCT5, OASL, RPL18A, NOP16, RBPJ, KLF4, ADAR, GYPC, KIAA0368, FERMT3, ATP5B, TTLL4, SENP5, ACTR3, BAG1, NARF, TUBB6, CAP1, TOP2B, DEDD2, SLC25A5, INPPL1, POLR1D, SF1, MBD2, RBBP6, SENP3, CHMP1A, MED4, PSEN1, RBM39, SERP1, MAP3K11, RBM34, SDCCAG8, POLR2F, YWHAZ, YPEL3, RPL27A, MRPL20, STAT6, CYLD, STAT4, CIR1, MEFV, MRPL14, KLC1, MRPL18, BCL6, THAP2, ETV6, FAM32A, BCAS2, RBM22, RBM23, RRP12, ILF3, STAT1, BIRC2, GABARAP, STAT3, MRPL23, PAPOLA, MRPL21, MRPL28, ILF2, IFT57, SVIL, CALM3, NOP58, APBB3, NOP56, UBXN6, CALM2, CALM1, MORF4L1, SEPT5, MRPS36, MRPS33, MORF4L2, CASK, CBX3, PNP, KLHL7, TARDBP, WIBG, TPT1, GNL2, PPP4C, RPL36AL, BASP1, TNNT1, MRPS18C, MRPS18A, LYST, PSMA3, CSTB, SDCBP, CSTA, MAP7D1, SEPT6, DYNLRB1, SEPT9, MYL6, MRPS15, HAX1, DIAPH1, SSH2, MRPS11, NEDD9, MYL9, DAZAP1, PSMB4, BLOC1S2, EIF3E, NPM1, EIF3L, GABARAPL1, MRPS28, MRPS24, EVL, SNW1, KAT5, SUGT1, FOXP1, CORO1C, CORO1A, PSMC5, WDR1, ASNA1, TLN1, MKI67IP, FUBP1, FOS, PCGF5, GSN, ACTR1A, MSN, ZYX, TUBA1A, TUBA1B, PSMD8, IFNGR1, TUBA1C, ELP2, UBE2A, SP100, MPP1, NOL7, UBE2I, MRPS6, DYNLT1, ARHGAP24, UBE2B, ARHGAP26, PCGF1, EML4, EML2, TYK2, EML3, CFL1,	

		MDM2, TOM1, GLTSCR2, PPP4R2, UBE2Z, UTP6, TPM1, TPM4, TPM3, EXOSC10, NUDT21, PSTPIP1, CNN2, RSL24D1, TINF2, APEX1, ERCC1, ACTB, TCP1, ATXN1, NME1-NME2, ANXA11, PSPC1, SH3D19, RNF40, ACTR10	
GO:0043232~intracellular non-membrane-bounded organelle	1.46E-16	XRCC5, RPL18, RPL17, RPL19, RPL14, RPL13, XRCC6, RPL15, STOML2, RPLP2, H1FX, RPL22L1, WTAP, KIFC3, CITED2, CTNNB1, ACTG1, WDR74, HIST1H2BK, RPLP0, ILK, RPLP1, PQBP1, FAU, RPL10, RPL11, RPL12, H1F0, MCRS1, NCF2, PTBP1, MECP2, TOX4, MYH9, CTNNA1, UXT, RPS19, RPS16, SPAG5, RPS14, RPS15, RPS12, RPS13, FLII, ARL8A, RPS10, ARL8B, RPS11, MYO18A, NHP2L1, NFKBIE, HADHA, HADHB, RPS25, PEA15, PFN1, RPS27, RPS28, RPS29, ISY1, RPS20, RPS21, CDC42EP3, RPS23, RPS24, DHX9, RPSA, RPS9, HBA2, HBA1, RPS6, VDAC2, RPS5, GAS7, RPS8, FXR1, RPS7, H3F3B, POP4, H3F3C, ZNF385A, BACH1, TSG101, RPS19BP1, RTN3, OFD1, HSF1, DYNLL1, MAP1LC3B, QKI, SYNJ2, MICAL1, PARVG, STX5, EXOSC8, ARHGEF2, HIST1H1C, LYN, EXOSC1, WAS, FLNA, ELMO1, C19ORF33, EPB41L3, RPL41, CLIC4, PFDN5, TMSB4X, PARVB, TUFM, HIST1H2AC, FHL3, PML, RPL35, RPL36, ABI1, RPL37, ZNF655, RPL38, RPL30, EZR, RPL32, RPL31, PTK2B, RPL34, PPP2CA, EXOC4, WDR13, USP36, TRIP10, PHLDA1, NSA2, HIST1H2BD, TRIM28, RPL26, LMNA, RPL27, TRIM25, RPL24, RPL28, RPL29, LSP1, MEF2D, RPL23, RPL22, PTP4A1, ZNF259, MPHOSPH6, SSNA1, NCLN,	2.78E-15

		DNM2, EIF6, MTSS1, PRC1, SNRPD3, CAPZA1, RBM4, RBM5, RPS6KB2, RPS27L, SRP19, CTNNBL1, VCL, IDH3G, LRRC59, DDX24, IMP4, RPS27A, PABPN1, RPL35A, TWF2, MTA2, FMR1, HNRNPA2B1, ACTN1, PDE4D, TMSB10, JUNB, ARPC1A, ARPC1B, IGBP1, PDCD6IP, TNFAIP3, UBA52, SMARCA4, HMGB2, LITAF, ADORA2A, CDC42SE1, C14ORF166, ARPC4, ARPC5, ZNF330, HNRNPM, DOCK2, HNRNPK, RPL7, ARPC3, ARPC2, RPL6, HNRNPF, RPL9, JUND, RPL8, RPL3, HNRNPD, RPL5, RPL7A, RPL4, RPL10A, ARHGDIB, UBXN11, TAF7, MYO1G, AKAP8L, MYO1F, MYL12B, RPL23A, SMEK1, MYL12A, MPRIP, P2RX4, HDAC3, DDX56, HNRNPH2, ATF3, VCP, HDAC1, CDC42SE2, SEC13, RPL37A, HNRNPH1, HDAC6, MAD1L1, NBN, FOSL2, HINT1, RHOQ, RPS2, RHOU, RPS3, ZNF207, DKC1, RPS3A, CASP8, RHOA, LRRFIP1, VPS16, RHOF, INO80B, PPP2R1A, SSBP1, IFI16, SLC9A3R1, RPS4X, NCL, CDK5, HMGA1, VASP, ACADVL, ADRB2, ATG4D, TBCB, ATG4B, TBCA, TBCD, FRMD4B, IPO5, UBC, UBB, MAPRE1, FHOD1, LCP1, HAUS4, HAUS2, VIM, RPS15A, CALCOCO2, NR3C1, IVNS1ABP, SEC62, CHD2, PAFAH1B1, SH2B2, CHD4, HSPA9, ANXA1, DRG1, COTL1, FBL, ID2, RPL13A, S100A4, FAM110A, HMGN3, VAPA, PDLIM7, PDLIM5, S100A9, SNCA, CCT2, PDLIM1, CCT3, KIF13A, H2AFV, IFT20, HOMER3, H2AFZ, H2AFY, MRPL38, MRPL33, FTL, CLNS1A, MRPL53, MRPL52,	

		DBNL, GTPBP4, MRPL51, RAN, POLG, UBR4, POLB, H2AFJ, MAP1LC3B2, DCTN6, DCTN3, DCTN2, SUV420H2, REC8, BAZ1A, CNTROB, TUBA4A, MRPL47, BIN3, PDE4DIP, MRPL48, BIN1, FUS, ALDOA, TXN2, STK17B, MYO9B, CAPZB, BANF1, HNRNPA3, TAL1, FRG1, CEP170, MRPL54, MRPL55, SUPV3L1, EMD, IKZF1, SUB1, PMF1, DDX5, NOP10, HNRNPA1, S100A12, CCT5, OASL, RPL18A, NOP16, RBPJ, KLF4, ADAR, GYPC, KIAA0368, FERMT3, ATP5B, TTLL4, SENP5, ACTR3, BAG1, NARF, TUBB6, CAP1, TOP2B, DEDD2, SLC25A5, INPPL1, POLR1D, SF1, MBD2, RBBP6, SENP3, CHMP1A, MED4, PSEN1, RBM39, SERP1, MAP3K11, RBM34, SDCCAG8, POLR2F, YWHAZ, YPEL3, RPL27A, MRPL20, STAT6, CYLD, STAT4, CIR1, MEFV, MRPL14, KLC1, MRPL18, BCL6, THAP2, ETV6, FAM32A, BCAS2, RBM22, RBM23, RRP12, ILF3, STAT1, BIRC2, GABARAP, STAT3, MRPL23, PAPOLA, MRPL21, MRPL28, ILF2, IFT57, SVIL, CALM3, NOP58, APBB3, NOP56, UBXN6, CALM2, CALM1, MORF4L1, SEPT5, MRPS36, MRPS33, MORF4L2, CASK, CBX3, PNP, KLHL7, TARDBP, WIBG, TPT1, GNL2, PPP4C, RPL36AL, BASP1, TNNT1, MRPS18C, MRPS18A, LYST, PSMA3, CSTB, SDCBP, CSTA, MAP7D1, SEPT6, DYNLRB1, SEPT9, MYL6, MRPS15, HAX1, DIAPH1, SSH2, MRPS11, NEDD9, MYL9, DAZAP1, PSMB4, BLOC1S2, EIF3E, NPM1, EIF3L, GABARAPL1, MRPS28, MRPS24, EVL, SNW1, KAT5, SUGT1, FOXP1,	

		CORO1C, CORO1A, PSMC5, WDR1, ASNA1, TLN1, MKI67IP, FUBP1, FOS, PCGF5, GSN, ACTR1A, MSN, ZYX, TUBA1A, TUBA1B, PSMD8, IFNGR1, TUBA1C, ELP2, UBE2A, SP100, MPP1, NOL7, UBE2I, MRPS6, DYNLT1, ARHGAP24, UBE2B, ARHGAP26, PCGF1, EML4, EML2, TYK2, EML3, CFL1, MDM2, TOM1, GLTSCR2, PPP4R2, UBE2Z, UTP6, TPM1, TPM4, TPM3, EXOSC10, NUDT21, PSTPIP1, CNN2, RSL24D1, TINF2, APEX1, ERCC1, ACTB, TCP1, ATXN1, NME1-NME2, ANXA11, PSPC1, SH3D19, RNF40, ACTR10	
GO:0000502~proteasome complex	8.30E-15	PSMB10, KIAA0368, SHFM1, UBQLN1, PSMA7, PSMB4, PSMF1, PSMB7, PSMB6, PSMB1, PSMD1, PSMB2, PSMD2, PSMD4, PSMD6, PSMD7, TRIP12, PSMD8, PSMD9, UBXN1, PSMA2, ADRM1, PSMA1, PSMD13, PSMC5, PSMA6, PSME1, PSMC4, PSMA5, PSME2, PSMD11, PSMC3, PSMA4, PSMC2, PSMA3, POMP, PSME4	2.01E-13
GO:0031981~nuclear lumen	1.62E-14	XRCC5, MEF2C, S100A4, XRCC6, S100A9, CCT2, PDLIM1, WTAP, CTNNB1, ISG20, WDR74, EPC1, U2AF1, PQBP1, RPL11, SUPT5H, LUC7L3, GTPBP4, MCRS1, NCF2, ANAPC5, RAN, PTBP1, POLB, SKP1, TOX4, PPP1CA, RPS19, RPS14, RPS15, RPS13, FLII, LRCH4, MYO18A, FUS, SIVA1, CPSF3L, TXN2, NFKBIE, NHP2L1, ZCRB1, XAB2, RPS25, HNRNPA3, TAL1, FRG1, ISY1, PRPF40A, DHX9, SUB1, SRA1, SMAD3, RPS9, NXF1, PMF1, RPS6, DDX5, HNRNPA1, U2AF1L4, NOP10, FXR1, S100A12, RPS7, OASL, CCT5, NOP16, JAZF1, POP4, RBPJ, ZNF385A, TCF12, KLF4, ADAR,	3.78E-13

		BACH1, ENY2, TSG101, EZH2, NFKB1, NFKB2, SENP5, RPS19BP1, RTN3, HSF1, NARF, CREG1, QKI, TOP2B, DEDD2, STX5, EXOSC8, POLR1D, CCNH, SF1, NDUFA13, EXOSC1, RBBP7, MBD2, ECSIT, RBBP6, GTF2B, C19ORF33, SENP3, TAF10, EIF4A3, MED4, CHMP1A, RBM39, POLR2G, RBM34, POLR2F, POLR2E, POLR2L, CREM, SHFM1, YPEL3, POLR2I, RPL35, PML, RPL36, ZNF655, KIN, STAT6, PRPF19, SF3B1, SRRT, STAT4, SET, CIR1, SQSTM1, WDR13, USP36, SUPT4H1, THAP2, FAM32A, ETV6, PHLDA1, NSA2, RBM22, BCAS2, RRP12, RBM23, TRIM28, LMNA, YWHAB, ILF3, TRIM25, STAT1, STAT3, MEF2D, PAPOLA, DUSP3, RPS6KA1, RPL23, ILF2, SFPQ, TCEB2, ZNF259, CALM3, NOP58, NOP56, TCEB1, PHF5A, MPHOSPH6, CALM2, NCLN, CALM1, DUSP6, MORF4L1, EIF6, NCBP1, SNRPD3, MORF4L2, RBM4, SNRPD1, RBM5, RPS6KB2, SNRPD2, SRP19, CTNNBL1, KLHL7, KDM1A, IDH3G, SMARCD3, TARDBP, WIBG, SRRM2, DDX24, GNL2, IMP4, RPS27A, AKIRIN2, CIB1, PABPN1, STK24, MAGOH, RELA, MTA2, FMR1, HNRNPA2B1, ACTN1, PPP1CB, JUNB, KDM2A, SNRPB, CSTB, EDF1, SLU7, PAF1, SNRPC, SNRPF, SNRPE, UBA52, SNRPG, HMGB2, MCL1, ANAPC10, ANAPC11, DAZAP1, CXXC1, ZNF330, HNRNPL, HNRNPM, HNRNPK, DDX3X, TRIM69, HNRNPF, EIF3E, RPL9, NPM1, RPL3, EIF3L, TAF9, RPL5, UBE2D1, TFDP1, MAFB, TAF7, AKAP8L, SAP18, NR4A1, RNPS1, SNW1, KAT5, MED13L, INTS10, FOXP1, HDAC5, ATF5,	

		POLD4, DDX56, HDAC3, ATF4, CDKN1A, HNRNPH2, PSMC5, ATF3, HDAC1, EAF1, VCP, CIRBP, HDAC9, HNRNPH1, ASNA1, UBE2E1, HDAC6, NBN, FOSL2, E2F4, PRKAG2, MKI67IP, RPS2, ZNF207, FUBP1, FOS, PLRG1, DKC1, RPS3A, GTF2A2, CASP8, MSN, PSMD8, INO80B, ELP2, ATPAF2, SP100, NOL7, CCNL1, PRPF3, UBE2I, IFI16, NCL, HMGA1, PCGF1, ADRB2, IPO5, CFL1, UBC, MDM2, CPSF6, PSME4, UBB, KPNA2, MATR3, CPSF1, GLTSCR2, UBE2Z, UTP6, MGMT, NR3C1, IVNS1ABP, TCF7L2, EXOSC10, PRPF8, NUDT21, GTF3C6, GTF3C5, RSL24D1, APEX1, TINF2, CHD4, ERCC1, ACTB, CEBPB, FBL, GPS2, ATXN1, SON, ATXN3, ANXA11, PSPC1, SH3D19	
GO:0045271~respiratory chain complex I	1.04E-12	NDUFB3, NDUFB4, NDUFB5, NDUFB7, NDUFB8, NDUFB9, NDUFAB1, NDUFB1, NDUFB2, NDUFS6, NDUFS5, NDUFS4, NDUFS3, NDUFS2, NDUFA4, NDUFA5, NDUFA2, NDUFA3, NDUFB10, NDUFA9, NDUFA6, NDUFA7, NDUFC2, NDUFA13, NDUFC1, NDUFA10, NDUFA1, NDUFV1	2.34E-11
GO:0005747~mitochondrial respiratory chain complex I	1.04E-12	NDUFB3, NDUFB4, NDUFB5, NDUFB7, NDUFB8, NDUFB9, NDUFAB1, NDUFB1, NDUFB2, NDUFS6, NDUFS5, NDUFS4, NDUFS3, NDUFS2, NDUFA4, NDUFA5, NDUFA2, NDUFA3, NDUFB10, NDUFA9, NDUFA6, NDUFA7, NDUFC2, NDUFA13, NDUFC1, NDUFA10, NDUFA1, NDUFV1	2.34E-11
GO:0030964~NADH dehydrogenase complex	1.04E-12	NDUFB3, NDUFB4, NDUFB5, NDUFB7, NDUFB8, NDUFB9, NDUFAB1, NDUFB1, NDUFB2, NDUFS6, NDUFS5, NDUFS4, NDUFS3, NDUFS2, NDUFA4, NDUFA5, NDUFA2, NDUFA3, NDUFB10, NDUFA9, NDUFA6,	2.34E-11

		NDUFA7, NDUFC2, NDUFA13, NDUFC1, NDUFA10, NDUFA1, NDUFV1	
GO:0005730~nucleolus	4.16E-10	S100A4, EIF6, SNRPD3, S100A9, RBM4, MORF4L2, RBM5, CCT2, SRP19, WTAP, CTNNBL1, KLHL7, WDR74, IDH3G, WIBG, TARDBP, DDX24, PQBP1, RPL11, GNL2, IMP4, PABPN1, GTPBP4, MCRS1, NCF2, MTA2, FMR1, HNRNPA2B1, PTBP1, ACTN1, TOX4, JUNB, RPS19, RPS14, CSTB, FLII, RPS13, MYO18A, FUS, HMGB2, TXN2, NHP2L1, NFKBIE, ZNF330, DAZAP1, RPS25, HNRNPA3, HNRNPM, HNRNPK, FRG1, RPL9, HNRNPF, NPM1, ISY1, RPL3, EIF3L, RPL5, DHX9, SUB1, TAF7, RPS9, SNW1, KAT5, RPS6, DDX5, NOP10, HNRNPA1, FOXP1, FXR1, RPS7, S100A12, DDX56, CCT5, OASL, HNRNPH2, ATF3, PSMC5, VCP, NOP16, POP4, ASNA1, HNRNPH1, RBPJ, ZNF385A, KLF4, ADAR, BACH1, NBN, FOSL2, TSG101, MKI67IP, RPS2, SENP5, RPS19BP1, RTN3, ZNF207, FUBP1, FOS, DKC1, HSF1, RPS3A, CASP8, QKI, MSN, TOP2B, DEDD2, PSMD8, INO80B, STX5, ELP2, EXOSC8, SP100, POLR1D, NOL7, SF1, EXOSC1, IFI16, NCL, RBBP6, PCGF1, C19ORF33, SENP3, MED4, ADRB2, IPO5, MDM2, GLTSCR2, RBM34, POLR2F, UBE2Z, YPEL3, UTP6, PML, RPL35, RPL36, NR3C1, ZNF655, EXOSC10, STAT6, STAT4, CIR1, WDR13, USP36, THAP2, RSL24D1, ETV6, FAM32A, PHLDA1, NSA2, BCAS2, RBM22, RBM23, RRP12, TRIM25, ILF3, STAT1, STAT3, FBL, ATXN1, MEF2D, PAPOLA, ILF2, RPL23, ZNF259, PSPC1, NOP58, SH3D19, NOP56, MPHOSPH6, NCLN	9.06E-09

GO:0016469~proton-transporting two-sector ATPase complex	6.46E-10	ATP5D, ATP5E, ATP6V0E1, ATP5B, ATP6AP1, ATP5G2, ATP6V1G1, ATP6V1B2, ATP5G1, ATP5G3, ATP6V0B, ATP6V0C, ATP5L, ATP5O, ATP5I, ATP5H, ATP5J, TCIRG1, ATP5J2, ATP5F1, ATP6V1H, ATP6V1F, ATP6V1E1, ATP5C1, ATPIF1, ATP5A1	1.36E-08
GO:0045259~proton-transporting ATP synthase complex	9.65E-10	ATP5D, ATP5E, ATP5J2, ATP5B, ATP5F1, ATP5G2, ATP5G1, ATP5G3, ATP6V0C, ATP5C1, ATP5L, ATP5O, ATPIF1, ATP5A1, ATP5I, ATP5H, ATP5J	1.98E-08
GO:0005753~mitochondrial proton-transporting ATP synthase complex	1.40E-09	ATP5D, ATP5E, ATP5J2, ATP5B, ATP5F1, ATP5G2, ATP5G1, ATP5G3, ATP5C1, ATP5L, ATP5O, ATPIF1, ATP5A1, ATP5I, ATP5H, ATP5J	2.78E-08
GO:0030530~heterogeneous nuclear ribonucleoprotein complex	3.65E-08	RALY, PTBP1, HNRNPA2B1, RBMX, HNRNPA1, HNRNPA0, HNRNPU, HNRNPL, HNRNPH3, HNRNPK, HNRNPH2, HNRNPF, HNRNPD, HNRNPH1	7.05E-07
GO:0042470~melanosome	4.16E-08	HSP90AB1, RAB7A, YWHAZ, GANAB, PDIA3, ATP1B3, RAB5C, NAP1L1, PDIA6, ATP6V1B2, CANX, PRDX1, ANXA6, SND1, RAC1, RPN1, TMED10, HSPA5, HSPA8, RAB2A, P4HB, HSP90AA1, RAN, ERP29, YWHAB, SLC3A2, ATP1A1, YWHAE, ANXA2, LAMP1, HSP90B1, CCT4, PPIB, ANXA11, SDCBP, PDCD6IP	7.81E-07
GO:0048770~pigment granule	4.16E-08	HSP90AB1, RAB7A, YWHAZ, GANAB, PDIA3, ATP1B3, RAB5C, NAP1L1, PDIA6, ATP6V1B2, CANX, PRDX1, ANXA6, SND1, RAC1, RPN1, TMED10, HSPA5, HSPA8, RAB2A, P4HB, HSP90AA1, RAN, ERP29, YWHAB, SLC3A2, ATP1A1, YWHAE, ANXA2, LAMP1, HSP90B1, CCT4, PPIB, ANXA11, SDCBP, PDCD6IP	7.81E-07
GO:0031252~cell leading edge	4.65E-08	MTSS1, TLN1, S100A6, NRBP1, PDLIM7, ADORA2A, DIAPH1, NEDD9, ARF6, ABI1, MYO9B, TPM1, CAPZB, CTNNB1, RPS3,	8.50E-07

		ACTR3, EZR, ARPC3, CXCR4, ARPC2, GSN, RAC1, PSTPIP1, SYNJ2, PAFAH1B1, SH2B2, DBNL, PLD1, TESC, PLEK, BAIAP2, SRA1, S100A11, EVL, MYH9, SLC9A3R1, CDK5, GAS7, VASP, CORO1A, MTMR14, NME1-NME2, ITGA5, ITGB1BP1, RAB34, GNAS, LCP1, HDAC6	
GO:0005654~nucleoplasm	1.83E-07	MEF2C, XRCC5, XRCC6, PDLIM1, WTAP, ISG20, CTNNB1, EPC1, U2AF1, SUPT5H, LUC7L3, MCRS1, ANAPC5, RAN, PTBP1, POLB, SKP1, PPP1CA, RPS15, LRCH4, SIVA1, CPSF3L, ZCRB1, XAB2, TAL1, FRG1, PRPF40A, SUB1, SRA1, SMAD3, PMF1, NXF1, NOP10, U2AF1L4, HNRNPA1, JAZF1, TCF12, KLF4, ENY2, EZH2, NFKB1, NFKB2, RPS19BP1, CREG1, TOP2B, CCNH, POLR1D, NDUFA13, MBD2, ECSIT, RBBP7, GTF2B, EIF4A3, TAF10, SENP3, MED4, RBM39, POLR2G, POLR2F, POLR2E, POLR2L, CREM, POLR2I, SHFM1, PML, PRPF19, SRRT, SF3B1, CIR1, SET, SQSTM1, SUPT4H1, TRIM28, YWHAB, DUSP3, RPS6KA1, TCEB2, CALM3, TCEB1, CALM2, DUSP6, CALM1, MORF4L1, NCBP1, SNRPD3, RBM4, SNRPD1, RPS6KB2, SNRPD2, KDM1A, SMARCD3, SRRM2, WIBG, AKIRIN2, RPS27A, CIB1, PABPN1, STK24, RELA, MTA2, MAGOH, HNRNPA2B1, FMR1, PPP1CB, KDM2A, SNRPB, SLU7, EDF1, PAF1, SNRPC, SNRPF, SNRPE, UBA52, SNRPG, HMGB2, MCL1, ANAPC10, ANAPC11, CXXC1, HNRNPL, HNRNPK, DDX3X, TRIM69, EIF3E, HNRNPF, NPM1, EIF3L, TAF9, UBE2D1, TFDP1, MAFB, TAF7, NR4A1, SAP18, RNPS1, INTS10,	3.25E-06

		MED13L, KAT5, HDAC5, POLD4, ATF5, CDKN1A, ATF4, HDAC3, HNRNPH2, EAF1, HDAC1, CIRBP, HDAC9, HNRNPH1, HDAC6, UBE2E1, NBN, E2F4, PRKAG2, MKI67IP, RPS2, FOS, DKC1, PLRG1, GTF2A2, ATPAF2, ELP2, SP100, CCNL1, UBE2I, PRPF3, IFI16, HMGA1, NCL, UBC, CPSF6, MDM2, UBB, PSME4, KPNA2, CPSF1, MGMT, IVNS1ABP, TCF7L2, PRPF8, NUDT21, GTF3C6, GTF3C5, TINF2, APEX1, ERCC1, CHD4, ACTB, GPS2, ATXN1, ATXN3, SON, ANXA11, PSPC1	
GO:0005839~proteasome core complex	7.50E-07	PSMB10, PSMA7, PSMA2, PSMA1, PSMF1, PSMB4, PSMB7, PSMB6, PSMA6, PSMB1, PSMA5, PSMA4, PSMA3, PSMB2	1.30E-05
GO:0005773~vacuole	7.62E-07	SGSH, GM2A, NAAA, HEXA, ATP6AP1, HEXB, USE1, HPS1, IL4I1, ZNRF1, ASAH1, IDS, MAP1LC3B, VPS16, DPP7, CLN3, STX4, CTSZ, STX3, ATP6V1H, STXBP2, VTI1B, CD164, M6PR, ATP6V1F, ADRB2, CHID1, NPC2, PSEN1, PRDX6, SBF2, TIAL1, GAA, CTSC, ARL8A, ARL8B, CTSH, RAB7A, STX8, LITAF, IFI30, PPT1, CTSA, ATP6V1G1, ADA, CD74, ATP6V0B, ATP6V0C, SLC11A2, SLC11A1, CD68, LAPTM5, TRIP10, SRGN, GABARAPL2, RILP, GABARAPL1, RNASE2, PSAP, GUSB, CD63, MANBA, GABARAP, TMEM55B, DNPEP, MARCH2, LAMP1, LAMP2, SLC15A3, DRAM1	1.29E-05
GO:0030532~small nuclear ribonucleoprotein complex	1.83E-06	SNRPA1, SNRPD3, SNRPB2, SNRPD1, SNRPD2, PRPF8, SNRPB, SLU7, LSM4, SNRNP40, SNRPC, PHF5A, SNRPF, SNRPE, SNRPG	3.02E-05
GO:0001726~ruffle	3.02E-06	MTSS1, S100A6, TLN1, PDLIM7, DIAPH1, MYO9B, ARF6, TPM1,	4.86E-05

		RPS3, EZR, SYNJ2, SH2B2, DBNL, TESC, PLEK, BAIAP2, S100A11, MYH9, SLC9A3R1, GAS7, MTMR14, NME1-NME2, ITGA5, ITGB1BP1, RAB34, GNAS, LCP1	
GO:0031982~vesicle	3.54E-06	SEPT5, MTSS1, AP1G2, ATP1B3, VAPA, RGL4, ZNRF1, TGFB1, CHIC2, PICALM, CLK3, SND1, RPN1, IL15RA, IL1B, PLD1, NCF2, RAN, ERP29, ACTN1, CORO7, OPTN, MAP1LC3B2, SPAG9, CD36, PPBP, ARRB2, RHCG, VEGFA, SDCBP, RAB13, PDCD6IP, BIN1, ITGA2B, ALDOA, SRI, RAB7A, AGFG1, GRB2, CLU, COPZ1, PPT1, ATP6V1B2, RFFL, ABCA1, TIMP1, SLC11A2, SLC11A1, ECE1, RAC1, TMED10, SRGN, AP2M1, ARHGDIB, P4HB, RILP, RAB8B, BECN1, GARS, DENND1A, PMF1, LAMP1, CORO1A, LAMP2, CCT4, PPIB, YIPF3, PTPN1, TREML1, HSP90AB1, CLTA, CLTB, PDIA3, KIAA0368, LDLR, RAB5C, AP2S1, HEXB, NAP1L1, HPS1, ESD, PDIA6, ANPEP, CANX, PRDX1, RABAC1, AP1S2, CD93, MAP1LC3B, MAPKAP1, COPB1, NECAP2, SERPINA1, DPP7, CLINT1, IFNGR1, CLN3, STX4, HSP90AA1, STX3, ACRBP, CAMP, ANP32E, SLC3A2, STXBP2, PKN1, PIGT, WAS, LRPAP1, NCK2, PSEN1, CLIC4, PRDX6, MDM2, GNAS, COPE, RIN3, YWHAZ, AP1M1, GANAB, PF4, GPRC5A, GCH1, ANXA6, ANXA7, CD9, TMED2, KLC1, RAB11A, HSPA5, EHD1, THBS1, HSPA8, PHLDA1, RAB2A, YWHAB, ATP1A1, SPARC, SOD1, YWHAE, MARCH3, AGTRAP, ANXA2, CYBA, HSP90B1, LRP1, AP2A1, ANXA11	5.56E-05
GO:0000323~lytic vacuole	4.97E-	SGSH, GM2A, NAAA, HEXA,	7.64E-05

	06	HEXB, HPS1, USE1, IL4I1, ZNRF1, ASAH1, IDS, VPS16, DPP7, CLN3, CTSZ, STX3, VTI1B, STXBP2, CD164, M6PR, ADRB2, CHID1, NPC2, PSEN1, PRDX6, TIAL1, GAA, CTSC, ARL8A, ARL8B, CTSH, RAB7A, STX8, LITAF, IFI30, PPT1, CTSA, ATP6V1G1, CD74, ADA, SLC11A2, SLC11A1, CD68, LAPTM5, TRIP10, SRGN, RILP, RNASE2, PSAP, GUSB, CD63, MANBA, GABARAP, TMEM55B, MARCH2, LAMP1, LAMP2, SLC15A3, DRAM1	
GO:0005764~lysosome	4.97E-06	SGSH, GM2A, NAAA, HEXA, HEXB, HPS1, USE1, IL4I1, ZNRF1, ASAH1, IDS, VPS16, DPP7, CLN3, CTSZ, STX3, VTI1B, STXBP2, CD164, M6PR, ADRB2, CHID1, NPC2, PSEN1, PRDX6, TIAL1, GAA, CTSC, ARL8A, ARL8B, CTSH, RAB7A, STX8, LITAF, IFI30, PPT1, CTSA, ATP6V1G1, CD74, ADA, SLC11A2, SLC11A1, CD68, LAPTM5, TRIP10, SRGN, RILP, RNASE2, PSAP, GUSB, CD63, MANBA, GABARAP, TMEM55B, MARCH2, LAMP1, LAMP2, SLC15A3, DRAM1	7.64E-05
GO:0045263~proton-transporting ATP synthase complex, coupling factor F(o)	1.10E-05	ATP6V0C, ATP5J2, ATP5F1, ATP5L, ATP5G2, ATP5O, ATP5G1, ATP5I, ATP5H, ATP5G3, ATP5J	1.65E-04
GO:0005759~mitochondrial matrix	1.30E-05	MRPS36, ATP5D, ATP5E, GRPEL1, MRPS33, ATP5B, NDUFAB1, QARS, ALAS1, IDH3G, LRRC59, PDHA1, MRPL52, MRPL51, SSBP1, POLG, SLC25A5, SUCLG1, CYCS, NDUFA10, ACADVL, MRPS18C, C1QBP, MRPS18A, CLPP, ATP5C1, MRPL47, MDH2, TUFM, MRPS15, ETHE1, FARS2, MRPS11, NR3C1, HADHA, KARS, MRPL20, HADHB, GSTK1, MRPL55, MRPL18, SUPV3L1, HSPE1, ETFB, ETFA, HSPA9, MRPS28, NDUFA9,	1.92E-04

		MRPS24, ATP5F1, GARS, IDH3B, VDAC2, SOD1, SOD2, PPIF, MRPL23, MRPL28, GLS, ALDH2, ATP5A1	
GO:0031980~mitochondrial lumen	1.30E-05	MRPS36, ATP5D, ATP5E, GRPEL1, MRPS33, ATP5B, NDUFAB1, QARS, ALAS1, IDH3G, LRRC59, PDHA1, MRPL52, MRPL51, SSBP1, POLG, SLC25A5, SUCLG1, CYCS, NDUFA10, ACADVL, MRPS18C, C1QBP, MRPS18A, CLPP, ATP5C1, MRPL47, MDH2, TUFM, MRPS15, ETHE1, FARS2, MRPS11, NR3C1, HADHA, KARS, MRPL20, HADHB, GSTK1, MRPL55, MRPL18, SUPV3L1, HSPE1, ETFB, ETFA, HSPA9, MRPS28, NDUFA9, MRPS24, ATP5F1, GARS, IDH3B, VDAC2, SOD1, SOD2, PPIF, MRPL23, MRPL28, GLS, ALDH2, ATP5A1	1.92E-04
GO:0033177~proton-transporting two-sector ATPase complex, proton-transporting domain	1.31E-05	TCIRG1, ATP6V0E1, ATP5J2, ATP5F1, ATP5G2, ATP5G1, ATP5G3, ATP6V0B, ATP6V0C, ATP5L, ATP5O, ATP5I, ATP5H, ATP5J	1.88E-04
GO:0005783~endoplasmic reticulum	2.74E-05	HM13, VAPA, PTGS2, USE1, DNAJB12, RAB1A, DNAJB11, INSIG1, RPL10, PSENEN, AUP1, PLD1, UBE2J1, UBE2J2, ERGIC1, YIF1A, ERGIC3, TRAPPC9, MLEC, SURF4, TRAPPC5, EXT1, TRAPPC1, TRAPPC3, HSD17B10, OAS1, CALR, CD74, TMEM50A, LPCAT1, SERINC1, PEMT, TRAM1, EMD, ATP2A3, CNIH4, AHSA1, ALG13, RTN4, PDIA3, KIAA0368, ORMDL1, BSCL2, PDIA6, CANX, RTN3, TRAPPC6A, AGPAT6, SRPR, ANP32A, KDELR1, STX5, CLN3, CNPY3, CNPY2, PI4KB, LRPAP1, NCK2, PSEN1, SIL1, LRMP, ORMDL2, DEGS1, SERP1, GANAB, ABI1, FKBP1A, SET, ACSL1, PLIN2, ALOX5AP, PEX16, MPDU1, PCMT1,	3.85E-04

		ACSL3, ACSL5, RAB2A, CES1, SELK, MARCH6, GABARAP, AGTRAP, MARCH2, GBA2, PTP4A1, BNIP3L, DPM1, SVIP, TAPBPL, SSR4, SSR2, NCLN, SSR3, EIF5A, PRKCSH, DSE, FDFT1, ELOVL1, IDS, ELOVL5, LRRC59, RPN1, SEP15, IL15RA, ASPH, RPN2, SAR1B, SAR1A, FNDC3B, DNAJC1, CIB1, SPTLC2, ERP29, TECR, PNPLA6, POR, BCAP31, PNPLA8, SEC61B, SDCBP, SEC61G, CNBP, STX8, DERL2, DERL1, HAX1, UBE2V1, CTSA, NAPA, LMAN2, MIA3, SEC61A1, P4HB, HERPUD1, GABARAPL1, CKAP4, SEC11A, TXNDC12, TXNDC11, VCP, PPIB, SPCS3, SPCS1, ANTXR2, PTPN1, PDCD6, CYB5R3, OS9, HMOX2, TMEM173, IFNGR2, IFNGR1, CTSZ, SGK1, PIGT, KTN1, TMEM189, KRTCAP2, TMEM38B, DGAT1, DGAT2, SIGLEC5, NUCB2, CTSC, PRNP, CTSH, PPP1R15A, FKBP2, OSTC, FKBP8, PGS1, APH1A, NDUFB8, RSAD2, SEC62, GPRC5A, SLC35B1, INPP5K, SHISA5, DAD1, CD4, HSPA5, APEX1, EHD4, EBP, TMC6, PLP2, TMBIM6, TMED9, MGST3, HSP90B1, SULF2, BAX, TRPC4AP, MGST1, MGST2	
GO:0015629~actin cytoskeleton	3.40E-05	MTSS1, PDLIM7, PDLIM5, FERMT3, SNCA, CAPZA1, CASK, RHOQ, RHOU, VCL, ACTR3, ACTG1, GSN, ACTR1A, ILK, VPS16, ZYX, CAP1, INPPL1, DCTN6, MYH9, DCTN3, CTNNA1, SLC9A3R1, WAS, VASP, FLNA, DCTN2, ARPC1A, ARPC1B, TNNT1, CLIC4, CFL1, BIN1, MYO18A, LCP1, SEPT9, ALDOA, MYL6, HAX1, STK17B, FHL3, MYO9B, ARPC4, ARPC5, TPM1, CAPZB, TPM4, TPM3, MYL9, PFN1, EZR, ARPC3, ARPC2, PSTPIP1,	4.70E-04

		SH2B2, CDC42EP3, MYO1G, MYO1F, MYL12B, MYL12A, GAS7, GABARAP, CORO1C, LSP1, CORO1A, SVIL, ACTR10	
GO:0005768~endosome	6.09E-05	CYTIP, STARD3NL, LDLR, AP1G2, KIAA0368, RAB5C, TSG101, CHMP4B, CHMP5, VPS53, IL15, ZNRF1, B2M, SLA, STARD3, RNF103, RABGEF1, TPT1, VPS16, CLN3, PLD1, SCAMP2, HLA-A, PI4KB, CD164, M6PR, CHMP1A, ADRB2, CHMP1B, CCR5, VAMP8, TOM1, ARL8A, ARL8B, RIN3, VPS29, CHMP2A, RAB7A, SNX17, FPR1, ARF6, ATP6V0B, CD74, SLC11A2, SLC11A1, CD68, ECE1, SQSTM1, SNF8, NUMB, RAB11A, CD4, EHD1, SDF4, EHD4, RILP, WDFY1, RUFY1, CD63, TMEM55B, MARCH3, ANXA2, MARCH2, LAMP1, LAMP2, LRP1, RABEP1, FYN, PTP4A1, ATP6V1E1, RAB34, PTP4A2, HGS, GGA1, VPS28, GGA2	8.23E-04
GO:0005832~chaperonin-containing T-complex	8.31E-05	CCT7, CCT5, TCP1, CCT4, CCT2, CCT3, CCT6A	1.10E-03
GO:0031988~membrane-bounded vesicle	1.02E-04	SEPT5, MTSS1, AP1G2, ATP1B3, RGL4, TGFB1, CHIC2, PICALM, CLK3, SND1, RPN1, IL1B, NCF2, RAN, ERP29, ACTN1, CORO7, OPTN, SPAG9, CD36, PPBP, VEGFA, SDCBP, PDCD6IP, BIN1, ITGA2B, ALDOA, RAB7A, AGFG1, GRB2, COPZ1, CLU, PPT1, ATP6V1B2, RFFL, ABCA1, TIMP1, SLC11A1, ECE1, RAC1, TMED10, SRGN, ARHGDIB, AP2M1, P4HB, RILP, BECN1, GARS, DENND1A, PMF1, LAMP1, CORO1A, LAMP2, CCT4, PPIB, YIPF3, TREML1, HSP90AB1, CLTA, CLTB, KIAA0368, LDLR, PDIA3, RAB5C, AP2S1, HEXB, NAP1L1, HPS1, ESD, PDIA6, ANPEP, CANX, PRDX1, RABAC1, AP1S2, CD93, MAPKAP1, COPB1, NECAP2, SERPINA1,	1.32E-03

		DPP7, CLINT1, CLN3, STX4, HSP90AA1, STX3, ACRBP, CAMP, ANP32E, SLC3A2, STXBP2, PKN1, PIGT, WAS, NCK2, PRDX6, MDM2, COPE, RIN3, YWHAZ, GANAB, PF4, ANXA6, CD9, TMED2, RAB11A, HSPA5, THBS1, EHD1, HSPA8, PHLDA1, RAB2A, YWHAB, ATP1A1, SPARC, YWHAE, ANXA2, CYBA, HSP90B1, LRP1, AP2A1, ANXA11	
GO:0033178~proton-transporting two-sector ATPase complex, catalytic domain	1.02E-04	ATP5D, ATP5E, ATP5B, ATP6AP1, ATP6V1E1, ATP5C1, ATP6V1H, ATP5O, ATP6V1B2, ATP5A1, ATP6V1F	1.30E-03
GO:0005852~eukaryotic translation initiation factor 3 complex	1.03E-04	EIF3D, EIF3B, EIF3G, EIF3H, EIF3E, EIF3F, EIF3K, EIF3L, EIF3I, EIF3M	1.29E-03
GO:0031410~cytoplasmic vesicle	1.17E-04	SEPT5, MTSS1, AP1G2, ATP1B3, RGL4, ZNRF1, TGFB1, CHIC2, PICALM, CLK3, SND1, RPN1, IL15RA, IL1B, NCF2, RAN, ERP29, ACTN1, CORO7, OPTN, MAP1LC3B2, SPAG9, CD36, PPBP, ARRB2, RHCG, VEGFA, SDCBP, RAB13, PDCD6IP, BIN1, ITGA2B, RAB7A, AGFG1, COPZ1, CLU, PPT1, ATP6V1B2, RFFL, ABCA1, TIMP1, SLC11A2, SLC11A1, ECE1, RAC1, TMED10, SRGN, ARHGDIB, AP2M1, P4HB, RILP, RAB8B, BECN1, GARS, DENND1A, LAMP1, CORO1A, LAMP2, CCT4, PPIB, YIPF3, PTPN1, TREML1, HSP90AB1, CLTA, CLTB, PDIA3, KIAA0368, LDLR, RAB5C, AP2S1, HEXB, NAP1L1, HPS1, ESD, PDIA6, CANX, PRDX1, RABAC1, AP1S2, CD93, MAP1LC3B, MAPKAP1, COPB1, NECAP2, SERPINA1, DPP7, CLINT1, CLN3, STX4, HSP90AA1, STX3, ACRBP, CAMP, ANP32E, SLC3A2, STXBP2, PKN1, PIGT, PSEN1, CLIC4, PRDX6, MDM2, COPE, RIN3, YWHAZ, AP1M1, GANAB, PF4, GPRC5A, GCH1,	

		ANXA6, CD9, TMED2, KLC1, RAB11A, HSPA5, EHD1, THBS1, HSPA8, PHLDA1, RAB2A, YWHAB, ATP1A1, SPARC, SOD1, YWHAE, MARCH3, AGTRAP, ANXA2, CYBA, HSP90B1, LRP1, AP2A1, ANXA11	
GO:0005741~mitochondrial outer membrane	1.44E-04	CYB5R3, BID, TSPO, MCL1, CHKB, HK2, HK1, KMO, MFF, FIS1, TMEM173, TOMM7, ACSL1, TOMM6, SH3GLB1, TOMM5, CASP8, ACSL3, ACSL5, RAF1, PI4KB, BAD, VDAC2, BCL2L11, BAX, TOMM20, TOMM22, GK, MGST1	1.74E-03
GO:0005794~Golgi apparatus	1.47E-04	GNPTG, HRAS, AP1G2, RAB1B, VPS53, IL15, DSE, UXS1, TGFB1, RAB1A, KIFC3, B2M, CUL3, ST3GAL1, ST3GAL3, CHIC2, PICALM, CD44, IFT20, SND1, ST3GAL6, IL15RA, PRKACA, GNG2, PSENEN, SAR1B, RAB20, SAR1A, H1F0, PLD1, SCAMP2, BST2, CECR1, HLA-A, VTI1B, CORO7, OPTN, TOX4, YIF1A, ERGIC1, ERGIC3, BCAP31, MGAT1, PNPLA8, GLUL, CD36, TRAPPC9, VAMP8, ATP2C1, TRAPPC5, VAMP5, PDE4DIP, RAB13, RAB10, EXT1, TRAPPC1, MYO18A, TRAPPC3, RAB7A, ARFGAP2, MFNG, ARFGAP3, STX8, LITAF, AGFG1, GRB2, NFKBIE, COPZ1, RER1, NEDD9, NAPA, ARF6, PPT1, ARF5, LMAN2, ABCA1, ATP6V1B2, CD74, SLC11A2, ECE1, LPCAT1, ARPC2, TMED10, STX11, STX10, LFNG, SDF4, SRGN, B4GALT5, MGAT4B, B4GALT1, GABARAPL2, GABARAPL1, B4GALT3, LAPTM4A, BECN1, TAF7, ARF3, ARF4, YIPF3, NBN, CLTA, CLTB, KIAA0368, NDST2, SDC4, RHOU, RTN3, ACTR3, RABAC1, TRAPPC6A, GOLGA7, AP1S2, COPB1,	1.74E-03

		CHST11, CHST15, PAK1, CLINT1, PLCB2, STX5, CLN3, ARHGEF2, STX4, ZDHHC3, LYN, PI4KB, FLNA, GAK, NUCB1, PSEN1, NUCB2, GNAS, CTSC, MAPRE1, CUX1, PRNP, COPE, AP1M1, GANAB, APH1A, SNX17, CALCOCO2, RSAD2, ITM2B, GPRC5A, SEC14L1, GLIPR2, TMED2, SH3GLB1, TMED3, SLC35B2, RAB11A, TRIP10, QSOX1, RAB2A, SECTM1, GDI2, PSAP, NSFL1C, TRIM22, GABARAP, AGTRAP, GJB2, COG3, CYBA, SULF2, AP2A1, IFT57, RAB34, DYM, SVIP, SH3D19, GGA1, GGA2, ARAP1	
GO:0005625~soluble fraction	1.51E-04	LDHB, KYNU, CYB5R2, HEXB, HPS1, ANPEP, RAB1A, ACTG1, WARS, SPINT2, GPX4, GPX3, PDE4B, NSMAF, PPP2R1A, IK, YARS, PFKL, NCF1, CSNK1G2, PHKG2, FMR1, FBP1, PDE4D, CORO7, POR, ADM, TNFSF13B, CLIC4, PTMS, RNPEP, MAP3K11, MDH1, YWHAZ, TALDO1, HAX1, FARS2, CXCL2, CALCOCO2, MYO9B, ABI1, ADRBK1, COMT, NAGK, KARS, GCH1, ANXA7, TPI1, ACSL1, RASGRP4, PPP2CA, PAFAH1B1, CCS, SDF4, ACTB, UNC119, RAB8B, PLEK, SPHK1, GARS, CAPN2, MANBA, PTPN12, SOD2, PTPN11, ANXA2, CD55, TNFSF10, NME1-NME2, PYGL, BAX, YWHAQ, ASNA1, DUSP6	1.76E-03
GO:0031968~organelle outer membrane	1.58E-04	CYB5R3, BID, TSPO, MCL1, CHKB, HK2, HK1, KMO, MFF, FIS1, TMEM173, TOMM7, ACSL1, TOMM6, SH3GLB1, TOMM5, CASP8, ACSL3, EMD, ACSL5, RAF1, PI4KB, BAD, VDAC2, BCL2L11, PSEN1, BAX, NUCB2, TOMM20, TOMM22, GK, MGST1	1.80E-03
GO:0005793~ER-Golgi intermediate compartment	2.21E-04	P4HB, KIAA0368, PDIA6, ANPEP, LMAN2, TMED9,	2.49E-03

		YIF1A, ERGIC1, NUCB1, C19ORF10, TMED2, NUCB2, SURF4, TMED10, HSPA5, MYO18A, KDELR1	
GO:0016023~cytoplasmic membrane-bounded vesicle	3.23E-04	SEPT5, MTSS1, AP1G2, ATP1B3, RGL4, TGFB1, CHIC2, PICALM, CLK3, SND1, RPN1, IL1B, NCF2, RAN, ERP29, ACTN1, CORO7, OPTN, SPAG9, CD36, PPBP, VEGFA, SDCBP, PDCD6IP, BIN1, ITGA2B, RAB7A, AGFG1, COPZ1, CLU, PPT1, RFFL, ATP6V1B2, ABCA1, TIMP1, SLC11A1, ECE1, RAC1, TMED10, SRGN, ARHGDIB, AP2M1, P4HB, RILP, BECN1, GARS, DENND1A, LAMP1, LAMP2, CORO1A, CCT4, PPIB, YIPF3, TREML1, HSP90AB1, CLTA, CLTB, KIAA0368, LDLR, PDIA3, RAB5C, AP2S1, HEXB, NAP1L1, HPS1, ESD, PDIA6, PRDX1, CANX, RABAC1, AP1S2, CD93, MAPKAP1, COPB1, NECAP2, SERPINA1, DPP7, CLINT1, CLN3, STX4, HSP90AA1, STX3, ACRBP, CAMP, ANP32E, SLC3A2, STXBP2, PKN1, PIGT, PRDX6, MDM2, COPE, RIN3, YWHAZ, GANAB, PF4, ANXA6, CD9, TMED2, RAB11A, HSPA5, THBS1, EHD1, HSPA8, PHLDA1, RAB2A, YWHAB, ATP1A1, SPARC, YWHAE, ANXA2, CYBA, HSP90B1, LRP1, AP2A1, ANXA11	3.57E-03
GO:0019867~outer membrane	3.32E-04	CYB5R3, BID, TSPO, MCL1, CHKB, HK2, HK1, KMO, MFF, FIS1, TMEM173, TOMM7, ACSL1, TOMM6, SH3GLB1, TOMM5, CASP8, ACSL3, EMD, ACSL5, RAF1, PI4KB, BAD, VDAC2, BCL2L11, PSEN1, BAX, NUCB2, TOMM20, TOMM22, GK, MGST1	3.61E-03
GO:0022624~proteasome accessory complex	3.91E-04	PSMD13, PSME1, PSME2, PSMD1, PSMD2, PSMD7, PSMD8, PSMD9	4.19E-03
GO:0045261~proton-	4.63E-	ATP5D, ATP5E, ATP5B,	4.88E-03

transporting ATP synthase complex, catalytic core F(1)	04	ATP5C1, ATP5O, ATP5A1	
GO:0005770~late endosome	4.91E-04	RILP, RAB7A, CLN3, KIAA0368, TSG101, CD63, M6PR, CD74, SLC11A2, LAMP1, SLC11A1, LAMP2, RNF103, SQSTM1, SNF8, TPT1, ARL8A, ARL8B, VPS16, SDF4	5.10E-03
GO:0005925~focal adhesion	6.05E-04	LIMS1, TLN1, NEDD9, SDC4, RHOU, MLF2, VCL, ARHGAP4, DNAJC15, CD44, ARPC2, PTK2B, ILK, ZYX, PAK1, TES, PARVG, PTPRC, LPP, ACTN1, TRIM25, EVL, ARHGAP24, ARHGAP26, VASP, GAK, ITGA5, SDCBP, PARVB, ITGA2B	6.18E-03
GO:0030055~cell-substrate junction	6.60E-04	LIMS1, TLN1, NEDD9, SDC4, RHOU, MLF2, VCL, ARHGAP4, ACTR3, DNAJC15, CD44, ARPC2, PTK2B, ILK, ZYX, PAK1, TES, PARVG, PTPRC, LPP, ACTN1, EVL, TRIM25, ARHGAP24, VASP, ARHGAP26, GAK, ERBB2IP, ITGA5, SDCBP, PARVB, ITGA2B	6.64E-03
GO:0016281~eukaryotic translation initiation factor 4F complex	7.52E-04	EIF4B, EIF4G2, EIF4E, EIF4H, EIF4A2, EIF4A1, ANKHD1	7.45E-03
GO:0000267~cell fraction	7.96E-04	LDHB, HRAS, VAPA, AQP9, PTGS2, PDLIM5, XRCC6, SNCA, CD52, RAB1A, CTNNB1, ACTG1, NSMAF, PLD1, YARS, NCF1, BCL2L11, LILRB2, CD36, MDH1, TALDO1, GNAI2, FARS2, CHKB, AKAP13, OAS1, ADRBK1, ARF6, MYO9B, PPT1, NAGK, CALR, CD68, TPI1, RAC2, RAC1, PEMT, TMED10, UNC119, SPHK1, JTB, GARS, PTPN12, PTPN11, S100A12, CD55, SLC9A1, NRP2, ATP6V0E1, ANPEP, CD151, AGPAT6, DYNLL1, GPX4, GPX3, SYNJ2, KDELR1, SLC22A1, CLN3, STX4, STX3, LYN, PHKG2, ADM, PSEN1, CLIC4, TOMM22, GNAS, RNPEP, DEGS1, MAP3K11, BID, YWHAZ, CXCL2, PML, ABI1,	7.77E-03

		FKBP1A, GCH1, MTMR3, ACSL1, PPP2CA, CERK, ACSL3, ACSL5, GDI1, TBXAS1, PLEK, DLGAP4, LMNA, BIRC6, ATP1A1, CAPN2, MANBA, CAPN1, SOD2, SLC16A3, GBA2, LRP1, SLC16A7, PYGL, ARAF, DPM1, YWHAQ, TAPBPL, DUSP6, SSR3, GNPTG, SEPT5, CASK, IL15, AIP, FDFT1, LRRC59, PDE4B, PRKACA, DNAJC4, DNAJC1, CSNK1G2, FMR1, FBP1, CORO7, HLA-C, PDE4D, POR, ADRM1, PNPLA8, VAMP8, VAMP3, EMP1, HAX1, ADORA2A, ABCA1, KARS, ECE1, FOLR3, RPL7A, SDF4, GABARAPL2, P4HB, RAB8B, FIBP, SEC11A, CKAP4, PPIF, LAMP1, TNFSF10, LAMP2, VCP, ITGA5, CYFIP2, SPCS3, SPCS1, PTPN1, IKBKB, ASNA1, KYNU, CYB5R2, HEXB, HPS1, WARS, HMOX2, FOS, SPINT2, PLCB1, FOSL1, PPP2R1A, IK, PFKL, MPP1, CCDC88C, KTN1, MCTP1, ATP6V1F, ADRB2, DGAT1, DGAT2, TNFSF13B, MDM2, PTMS, PPM1A, CALCOCO2, CYTH2, COMT, ANXA7, SLC35B1, TNFRSF1B, TMED2, SH3GLB1, RASGRP4, RASGRP2, PAFAH1B1, CCS, ACTB, EBP, PLP2, OLR1, TMBIM6, ANXA2, MGST3, HSP90B1, NME1-NME2, BAX, RNF40, MGST1, SMPD2, MGST2	
GO:0005924~cell-substrate adherens junction	1.18E-03	LIMS1, TLN1, NEDD9, SDC4, RHOU, MLF2, VCL, ARHGAP4, DNAJC15, CD44, ARPC2, PTK2B, ILK, ZYX, PAK1, TES, PARVG, PTPRC, LPP, ACTN1, TRIM25, EVL, ARHGAP24, ARHGAP26, VASP, GAK, ITGA5, SDCBP, PARVB, ITGA2B	1.14E-02
GO:0005765~lysosomal membrane	1.40E-03	SLC11A2, RILP, STX8, LAMP2, LAPTM5, PSEN1, VTI1B, ARL8A, ARL8B, ATP6V1G1, CD63, SLC15A3	1.32E-02

GO:0005885~Arp2/3 protein complex	1.41E-03	ACTR3, ARPC1B, ARPC3, ARPC2, ARPC4, ARPC5	1.32E-02
GO:0048471~perinuclear region of cytoplasm	1.42E-03	NRBP1, VAPA, MRFAP1, TNFSF13, UBQLN1, ATXN10, GSN, ANP32A, SYNJ2, RARA, CLINT1, GTPBP4, PLD1, STX4, TWF2, LYN, OPTN, PI4KB, DCTN3, GAK, SPAG9, TAF10, PNPLA8, MTMR14, GNB2, BNIP2, GRASP, SEPT9, PTOV1, HMGB2, MOB2, NFKBIE, CLU, SIPA1, CALCOCO2, C14ORF166, MYO9B, ARF5, CALR, ANXA6, SLC11A2, CYLD, SET, ECE1, PTK2B, PSTPIP1, PAFAH1B1, HSPA5, TRIP10, GAPDH, APEX1, PLA2G16, LMNA, YWHAB, TPD52L2, KAT5, ANXA2, P2RX4, HSP90B1, NME1-NME2, ARF3, EIF4H, CYFIP2, DNAJB6, HDAC6	1.31E-02
GO:0005769~early endosome	1.91E-03	CYTIP, KIAA0368, SNX17, ARF6, B2M, SLC11A2, ECE1, NUMB, RABGEF1, CD4, VPS16, EHD1, CLN3, WDFY1, HLA-A, ANXA2, CHMP1A, RABEP1, VAMP8, PTP4A1, RAB34, PTP4A2, TOM1, HGS, RIN3	1.73E-02
GO:0000275~mitochondrial proton-transporting ATP synthase complex, catalytic core F(1)	2.51E-03	ATP5D, ATP5E, ATP5B, ATP5C1, ATP5A1	2.24E-02
GO:0012505~endomembrane system	3.08E-03	EIF6, GNPTG, S100A6, NRBP1, AP1G2, XPO6, VAPA, EIF5A, CBX3, WTAP, ANKLE2, FDFT1, B2M, ST3GAL1, EPC1, ST3GAL3, PICALM, AAK1, ST3GAL6, RPN1, RPN2, ASPH, SCAMP2, SPTLC2, RAN, HLA-A, CORO7, PNPLA6, BCAP31, MGAT1, CD36, SEC61B, VAMP8, ATP2C1, EXT1, SEC61G, TRAPPC3, ITGA2B, MVP, DERL2, MFNG, DERL1, HAX1, AGFG1, ADORA2A, GRB2, COPZ1, RER1, TAGLN2, SLC11A1, MIA3, RAC2, SERINC1, PEMT, TMED10, STX10, LFNG, EMD, TRAM1,	2.71E-02

		SEC61A1, AP2M1, GABARAPL2, RILP, HERPUD1, SEC11A, DENND1A, TREX1, SMAD3, NXF1, CORO1A, LAMP2, SPCS3, SEC13, SPCS1, RTN4, CYB5R3, MAD1L1, CLTA, CLTB, LDLR, ORMDL1, AP2S1, BSCL2, ANPEP, OS9, ACTR3, AP1S2, SRPR, GPX4, COPB1, NARF, NECAP2, STX5, CLN3, NUP88, KTN1, PIGT, PI4KB, KRTCAP2, WAS, TMEM38B, NCK2, CHMP1A, PSEN1, BNIP2, NUCB2, IPO5, LRMP, MDM2, GNAS, CUX1, KPNA2, MATR3, COPE, NXT1, OSTC, FKBP8, AP1M1, PML, NUP93, FKBP1A, ANXA7, CD9, NUP214, TMED2, ALOX5AP, PEX16, MPDU1, SHISA5, DAD1, PAFAH1B1, CD4, HSPA5, QSOX1, PHLDA1, PLP2, SLC2A9, LMNA, GABARAP, GJB2, HSP90B1, LRP1, AP2A1, BAX, BNIP3L, ANXA11, DPM1, SSR4, SSR2, SSR3	
GO:0005838~proteasome regulatory particle	3.29E-03	PSMD13, PSMD1, PSMD2, PSMD7, PSMD8, PSMD9	2.85E-02
GO:0005774~vacuolar membrane	3.70E-03	RILP, GABARAPL2, STX8, ATP6V1H, VTI1B, ATP6V1G1, CD63, GABARAP, ATP6V1F, SLC11A2, LAMP2, LAPTM5, PSEN1, SBF2, ARL8A, VPS16, ARL8B, SLC15A3	3.16E-02
GO:0009898~internal side of plasma membrane	3.80E-03	CYB5R3, CLTA, TLN1, HRAS, CLTB, GM2A, RAB5C, FERMT3, AP2S1, RAB1B, EIF5A, RHOU, VCL, CDC42, SMAP1, AP1S2, RAB28, COPB1, ILK, RHOA, RALA, GNG2, RHOC, VPS16, MSN, GNG5, RHOF, RHOG, RHOH, AUP1, RNF31, PARVG, PLD1, GBP5, RAP2C, RAB4B, CTNNA1, PNPLA6, DOK3, RAB13, RAB10, GBP2, GRASP, PARVB, COPE, GBP1, SERP1, CHMP2A, ARFGAP2, FKBP8, AP1M1, LIMS1, ARFGAP3, LITAF, COPZ1, RSAD2, EZR, RAC1, EHD1, AP2M1, RAB8B,	3.20E-02

		LSP1, RAB31, AP2A1, SVIL, HGS, PTPN1, GK	
GO:0000313~organellar ribosome	4.60E-03	MRPS36, MRPL52, MRPL51, MRPS28, MRPS15, MRPS33, MRPS11, MRPS24, MRPL20, MRPL23, MRPS18C, MRPL28, MRPS18A, MRPL55, MRPL18, MRPL47	3.82E-02
GO:0005761~mitochondrial ribosome	4.60E-03	MRPS36, MRPL52, MRPL51, MRPS28, MRPS15, MRPS33, MRPS11, MRPS24, MRPL20, MRPL23, MRPS18C, MRPL28, MRPS18A, MRPL55, MRPL18, MRPL47	3.82E-02
GO:0031201~SNARE complex	4.96E-03	STX5, STX4, STX3, VAMP8, VTI1B, STXBP2, NAPA, VAMP3, VTI1A	4.06E-02
GO:0042645~mitochondrial nucleoid	5.01E-03	ACADVL, TUFM, SSBP1, SLC25A5, POLG, ATP5B, LRRC59, SUPV3L1, VDAC2, HADHA, HSPA9, HADHB	4.06E-02
GO:0009295~nucleoid	5.01E-03	ACADVL, TUFM, SSBP1, SLC25A5, POLG, ATP5B, LRRC59, SUPV3L1, VDAC2, HADHA, HSPA9, HADHB	4.06E-02
GO:0016604~nuclear body	5.23E-03	MEF2C, PML, ISG20, CXXC1, SF3B1, CIR1, DKC1, DDX3X, PLRG1, TRIM69, FRG1, SRRM2, PRPF8, EIF3E, NPM1, NUDT21, U2AF1, LUC7L3, PRPF40A, ATPAF2, SP100, MAGOH, CCNL1, PRPF3, UBE2I, RNPS1, NXF1, U2AF1L4, NOP10, HDAC5, EIF4A3, SON, EAF1, PSPC1, SLU7, CPSF6, LRCH4, SNRPC, RBM39, PSME4	4.18E-02
GO:0044451~nucleoplasm part	5.81E-03	MORF4L1, MEF2C, RBM4, XRCC6, PDLIM1, CTNNB1, ISG20, KDM1A, EPC1, SRRM2, U2AF1, AKIRIN2, LUC7L3, MTA2, RELA, MAGOH, PPP1CB, PPP1CA, SLU7, EDF1, SNRPC, PAF1, LRCH4, CPSF3L, CXXC1, TAL1, DDX3X, TRIM69, FRG1, EIF3E, NPM1, TAF9, PRPF40A, TFDP1, MAFB, SUB1, SRA1, TAF7, SAP18, SMAD3, RNPS1, PMF1, NXF1, MED13L, KAT5, INTS10, U2AF1L4, NOP10, HDAC5,	4.58E-02

		ATF5, ATF4, HDAC3, EAF1, HDAC1, JAZF1, HDAC9, TCF12, KLF4, HDAC6, ENY2, E2F4, EZH2, FOS, DKC1, PLRG1, GTF2A2, CREG1, ELP2, ATPAF2, SP100, CCNH, CCNL1, UBE2I, PRPF3, MBD2, ECSIT, RBBP7, HMGA1, GTF2B, EIF4A3, TAF10, MED4, CPSF6, PSME4, RBM39, CPSF1, POLR2G, POLR2F, POLR2E, POLR2L, CREM, POLR2I, SHFM1, PML, IVNS1ABP, TCF7L2, SF3B1, CIR1, PRPF8, NUDT21, GTF3C6, GTF3C5, CHD4, ACTB, YWHAB, GPS2, ATXN1, SON, PSPC1	

Cellular Component (CC_FAT) associated with genes having decreased expression in **HNP_NeoHep** with respect to HNP_Monocyte

Term	PValue	Genes	Benjamini
GO:0030529~ribonucleoprotein complex	3.74E-17	MRPS36, NCBP1, NAF1, GAR1, RPL15, SNRPD1, RPL22L1, SRP19, PCBP1, U2AF1, MRPL32, LSM1, RPL36AL, RPL35A, SNRPA1, MAGOH, HNRNPA2B1, PRPF3, RPS4X, HNRNPU, PSMA1, EIF4A3, RPS16, LARP7, RPS14, RPS13, RPS10, SERP1, SNRPG, STRAP, SNRPB2, RPS15A, RPL37, RPS25, HNRNPA3, HNRNPL, CYLD, HNRNPK, RPL7, RPL31, RPL34, RPL9, RPL3, HNRNPD, DHX15, TAF9, RPL5, HNRNPC, RSL24D1, RPS20, RPS21, RPS23, NSA2, RPS24, BCAS2, SRA1, RPL26, RPL24, HBA2, HBA1, DDX5, RPS6, FXR1, RPS7, HNRNPH3, HNRNPH2, RPL22, RPL21, SYF2, PHF5A, HNRNPH1	1.57E-14
GO:0022626~cytosolic ribosome	3.48E-13	RPS15A, RPL37, RPS25, RPL7, RPL31, RPL34, RPL3, RPL5, RPS20, RPS21, RPS23, RPS24, RPL26, HBA2, RPL24, HBA1, RPS4X, RPS6, RPS7, RPS16, RPL22, RPL21, RPS14, RPS13, RPS10	7.30E-11
GO:0044445~cytosolic part	5.17E-13	RPS15A, RPL37, CTNNB1, RPS25, RPL7, RPL31, RPL34, PIK3C3, RPL3, RPL5, RPS20, RPS21, HBB, RPS23, RPS24, HBD, TCP1, RPL26, RPL24, HBA2, HBA1, RPS6, RPS4X, RPS7, PFDN2, RPS16, RPL22, RPS14, RPL21, PFDN4, AHSP, RPS13, RPS10	7.24E-11
GO:0005829~cytosol	4.98E-12	NCBP1, CHMP4B, CHMP5, PDLIM5, RPL15, SNRPD1, CASK, SLC7A5, PNP, CTNNB1, CUL5, PDE4B, MAP3K8, PIK3C3, RPL35A, NCF1, PDE4D, BCL2L11, PSMA2, PSMA1, RPS16,	5.23E-10

		RPS14, AHSP, RIPK2, RPS13, RPS10, GRAP2, SNRPG, ARFGAP3, EEF1B2, MVD, DIAPH2, NFKBIB, NFKBIA, ANAPC10, KMO, ADA, RPS25, RILPL2, RPL7, EIF3H, RPL9, EIF3F, HNRNPD, RPL3, RPL5, RPS20, RPS21, UBE2D1, RPS23, RPS24, UPB1, CIDEB, HBA2, HBA1, RPS6, ATG3, PTPN11, RPS7, S100A12, HNRNPH2, EIF4E, GK, KYNU, NFKB2, PTEN, RAB3IP, OFD1, NUBP1, RNF103, CASP9, COX19, SIK1, PLCB1, TOP2B, FOSL1, ZFP36, C9ORF89, RAB4A, RPS4X, SERPINB9, PFDN2, CHMP1B, SERPINB8, PFDN4, ALOX12, LCP2, BID, HAUS2, VIM, DCK, RPS15A, RPL37, GCH1, ZFP36L1, TYMP, PLEKHG2, RPL31, RPL34, RASGRP2, SOS2, PPP3CC, TPRKB, HBB, HBD, TCP1, AIMP1, RPL26, RPL24, RALGDS, ID2, RPL22, RPL21, SH3D19	
GO:0005840~ribosome	6.92E-11	MRPS36, RPL15, RPS15A, RPL37, RPL22L1, RPS25, CYLD, RPL7, RPL31, RPL9, RPL34, RPL3, RPL5, RPS20, RSL24D1, RPS21, MRPL32, RPS23, RPL36AL, RPS24, RPL35A, RPL26, HBA2, RPL24, HBA1, RPS6, RPS4X, RPS7, HNRNPH2, RPS16, RPL22, RPS14, RPL21, RPS13, RPS10, SERP1	5.81E-09
GO:0033279~ribosomal subunit	2.54E-10	MRPS36, RPS15A, RPL37, RPS25, RPL7, RPL31, RPL34, RPL3, RPL5, RPS20, MRPL32, RPS21, RPS23, RPS24, RPL26, RPL24, HBA2, HBA1, RPS4X, RPS6, RPS7, RPS16, RPL22, RPS14, RPL21, RPS13, RPS10	1.78E-08
GO:0022627~cytosolic small ribosomal subunit	8.06E-09	RPS15A, HBA2, HBA1, RPS6, RPS4X, RPS7, RPS25, RPS16, RPS14, RPS13, RPS10, RPS20, RPS21, RPS23, RPS24	4.84E-07

GO:0015935~small ribosomal subunit	4.38E-07	MRPS36, RPS15A, HBA2, HBA1, RPS6, RPS4X, RPS7, RPS25, RPS16, RPS14, RPS13, RPS10, RPS20, RPS21, RPS23, RPS24	2.30E-05
GO:0030530~heterogeneous nuclear ribonucleoprotein complex	4.05E-06	HNRNPL, HNRNPH3, HNRNPH2, HNRNPK, HNRNPA2B1, HNRNPD, HNRNPH1, HNRNPU	1.89E-04
GO:0031981~nuclear lumen	4.29E-06	MEF2C, NCBP1, S100A9, SNRPD1, WTAP, SRP19, CTNNB1, ISG20, EPC1, TARDBP, U2AF1, DDX21, GNL2, GTPBP4, MAGOH, HNRNPA2B1, JUNB, MED10, PCF11, RPS14, RPS13, SNRPG, HMGB1, HMGB2, MCL1, ANAPC10, HNRNPL, HNRNPA3, RPS25, TAL1, VRK1, HNRNPK, ZNF326, RPL9, RPL3, NPM3, RPL5, TAF9, UBE2D1, NFE2, SUB1, SRA1, RYBP, NR4A1, NXF1, RPS6, DDX5, FOXP1, S100A12, RPS7, FXR1, OASL, DNAJB9, HNRNPH2, HNRNPH1, KLF4, NBN, KDM6A, GAR1, EZH2, NFKB2, SENP5, FUBP1, ZNF350, GTF2A2, QKI, TWISTNB, TOP2B, MYC, INO80B, EXOSC8, CCNH, CCNL1, PRPF3, MBD2, GTF2B, C19ORF33, EIF4A3, ADRB2, LARP7, PSME4, RBM39, ING3, POLR2K, SHFM1, STAT4, TSPYL2, ERCC6, GTF3C6, THAP2, RSL24D1, NSA2, ETV3, PHLDA1, BCAS2, CEBPB, SH3D19, PHF5A, MPHOSPH6, DUSP6	1.80E-04
GO:0005681~spliceosome	4.87E-06	BCAS2, SNRPA1, STRAP, MAGOH, HNRNPA2B1, SNRPB2, SNRPD1, PRPF3, DDX5, HNRNPU, HNRNPA3, EIF4A3, HNRNPK, DHX15, SYF2, U2AF1, HNRNPC, PHF5A, HNRNPH1, SNRPG	1.86E-04
GO:0005730~nucleolus	8.40E-06	KDM6A, NBN, GAR1,	2.94E-04

		S100A9, WTAP, SRP19, SENP5, FUBP1, ZNF350, TARDBP, QKI, DDX21, TWISTNB, GNL2, TOP2B, INO80B, GTPBP4, EXOSC8, HNRNPA2B1, JUNB, C19ORF33, ADRB2, RPS14, RPS13, HMGB1, HMGB2, HNRNPA3, RPS25, VRK1, STAT4, HNRNPK, TSPYL2, ERCC6, RPL9, RPL3, NPM3, RPL5, THAP2, RSL24D1, NSA2, PHLDA1, BCAS2, NFE2, SUB1, DDX5, RPS6, FOXP1, S100A12, FXR1, RPS7, OASL, DNAJB9, HNRNPH2, SH3D19, HNRNPH1, MPHOSPH6, KLF4	
GO:0043233~organelle lumen	2.42E-05	MRPS36, MEF2C, NCBP1, PTGS2, F13A1, S100A9, SNRPD1, WTAP, SRP19, CTNNB1, ISG20, EPC1, TARDBP, U2AF1, DDX21, PDHA1, GNL2, MRPL32, GTPBP4, MAGOH, HNRNPA2B1, MED10, JUNB, PCF11, PPBP, RPS14, RPS13, SNRPG, HMGB1, HMGB2, MCL1, MVD, ANAPC10, RPS25, HNRNPA3, HNRNPL, TAL1, VRK1, HNRNPK, ZNF326, RPL9, RPL3, NPM3, HSPE1, RPL5, TAF9, UBE2D1, SRGN, FH, NFE2, SUB1, SRA1, RYBP, NR4A1, NXF1, RPS6, DDX5, FOXP1, FXR1, RPS7, S100A12, PPIF, OASL, DNAJB9, HNRNPH2, HNRNPH1, KLF4, ATP5E, NBN, KDM6A, GAR1, EZH2, NFKB2, SENP5, FUBP1, ZNF350, GTF2A2, QKI, TWISTNB, TOP2B, MYC, INO80B, EXOSC8, CCNH, CCNL1, PRPF3, MBD2, GTF2B, C19ORF33, EIF4A3, ADRB2, LARP7, PSME4, RBM39, PMPCB, ING3, POLR2K, SHFM1, PF4,	7.81E-04

		STAT4, TSPYL2, ERCC6, GTF3C6, THAP2, RSL24D1, THBS1, PHLDA1, ETV3, NSA2, BCAS2, CEBPB, SOD2, PHF5A, SH3D19, MPHOSPH6, DUSP6	
GO:0022625~cytosolic large ribosomal subunit	2.99E-05	RPL7, RPL31, RPL22, RPL34, RPL21, RPL3, RPL26, RPL37, RPL5, RPL24	8.97E-04
GO:0031974~membrane-enclosed lumen	3.54E-05	MRPS36, MEF2C, NCBP1, PTGS2, F13A1, S100A9, SNRPD1, WTAP, SRP19, CTNNB1, ISG20, EPC1, TARDBP, U2AF1, DDX21, PDHA1, GNL2, MRPL32, GTPBP4, MAGOH, HNRNPA2B1, MED10, JUNB, PCF11, PPBP, RPS14, RPS13, SNRPG, HMGB1, HMGB2, MCL1, MVD, ANAPC10, RPS25, HNRNPA3, HNRNPL, TAL1, VRK1, HNRNPK, ZNF326, RPL9, RPL3, NPM3, HSPE1, RPL5, TAF9, UBE2D1, SRGN, FH, NFE2, SUB1, SRA1, RYBP, NR4A1, NXF1, RPS6, DDX5, FOXP1, FXR1, RPS7, S100A12, PPIF, OASL, DNAJB9, HNRNPH2, HNRNPH1, KLF4, ATP5E, NBN, KDM6A, GAR1, EZH2, NFKB2, SENP5, FUBP1, ZNF350, GTF2A2, QKI, TWISTNB, TOP2B, MYC, COX17, INO80B, EXOSC8, CCNH, CCNL1, PRPF3, MBD2, GTF2B, C19ORF33, EIF4A3, ADRB2, LARP7, PSME4, RBM39, PMPCB, ING3, POLR2K, SHFM1, PF4, STAT4, TSPYL2, ERCC6, GTF3C6, THAP2, RSL24D1, THBS1, PHLDA1, ETV3, NSA2, BCAS2, CEBPB, SOD2, PHF5A, SH3D19, MPHOSPH6, DUSP6	9.90E-04
GO:0043232~intracellular non-membrane-bounded organelle	7.93E-05	MRPS36, MTSS1, TNNC2, PDLIM5, RPL15, S100A9, CASK, RPL22L1, SRP19, WTAP, PNP, CTNNB1,	2.08E-03

		TARDBP, TPT1, DDX21, MRPL32, GNL2, RPL36AL, H1F0, RPL35A, GTPBP4, HNRNPA2B1, PDE4D, BASP1, MAP1LC3B2, JUNB, TNNT3, REC8, BAZ1A, RPS16, SPAG5, RPS14, RPS13, RPS10, ARL8B, CSTA, BIN1, TNFAIP3, HMGB1, HMGB2, HMGB3, ADORA2A, B9D2, STK17B, NEDD9, C14ORF166, RPS25, HNRNPA3, TAL1, VRK1, CDC42EP2, HNRNPK, RPL7, RPL9, HNRNPD, RPL3, NPM3, RPL5, RPS20, RPS21, CDC42EP3, EMD, RPS23, RPS24, GABARAPL1, NFE2, IKZF1, SUB1, MYO1G, WAPAL, HBA2, HBA1, DDX5, RPS6, FOXP1, FXR1, RPS7, S100A12, OASL, CORO1A, DNAJB9, HNRNPH2, CDC42SE2, H3F3C, HNRNPH1, KLF4, KDM6A, NBN, GAR1, SENP5, RAB3IP, FUBP1, OFD1, ZNF350, MAP1LC3B, QKI, SYNJ2, TOP2B, TWISTNB, MYC, IFNGR1, INO80B, UBE2A, EXOSC8, HIST1H1D, HIST1H1C, DYNLT1, MID1IP1, RPS4X, MBD2, ARHGAP24, UBE2B, WAS, C19ORF33, ADRB2, SMTN, HIST2H2BE, TMSB4X, RBM39, FHOD1, SERP1, HIST1H2AC, PPP4R2, YWHAZ, HAUS2, VIM, RPS15A, RPL37, CYLD, STAT4, ERCC6, TSPYL2, MEFV, RPL31, RPL34, CHD1, RSL24D1, THAP2, PHLDA1, NSA2, BCAS2, TCP1, RPL26, ANXA1, RPL24, COTL1, TUBA8, ID2, RPL22, RPL21, SVIL, SH3D19, MPHOSPH6	
GO:0043228~non-membrane-bounded organelle	7.93E-05	MRPS36, MTSS1, TNNC2, PDLIM5, RPL15, S100A9, CASK, RPL22L1, SRP19,	2.08E-03

		WTAP, PNP, CTNNB1, TARDBP, TPT1, DDX21, MRPL32, GNL2, RPL36AL, H1F0, RPL35A, GTPBP4, HNRNPA2B1, PDE4D, BASP1, MAP1LC3B2, JUNB, TNNT3, REC8, BAZ1A, RPS16, SPAG5, RPS14, RPS13, RPS10, ARL8B, CSTA, BIN1, TNFAIP3, HMGB1, HMGB2, HMGB3, ADORA2A, B9D2, STK17B, NEDD9, C14ORF166, RPS25, HNRNPA3, TAL1, VRK1, CDC42EP2, HNRNPK, RPL7, RPL9, HNRNPD, RPL3, NPM3, RPL5, RPS20, RPS21, CDC42EP3, EMD, RPS23, RPS24, GABARAPL1, NFE2, IKZF1, SUB1, MYO1G, WAPAL, HBA2, HBA1, DDX5, RPS6, FOXP1, FXR1, RPS7, S100A12, OASL, CORO1A, DNAJB9, HNRNPH2, CDC42SE2, H3F3C, HNRNPH1, KLF4, KDM6A, NBN, GAR1, SENP5, RAB3IP, FUBP1, OFD1, ZNF350, MAP1LC3B, QKI, SYNJ2, TOP2B, TWISTNB, MYC, IFNGR1, INO80B, UBE2A, EXOSC8, HIST1H1D, HIST1H1C, DYNLT1, MID1IP1, RPS4X, MBD2, ARHGAP24, UBE2B, WAS, C19ORF33, ADRB2, SMTN, HIST2H2BE, TMSB4X, RBM39, FHOD1, SERP1, HIST1H2AC, PPP4R2, YWHAZ, HAUS2, VIM, RPS15A, RPL37, CYLD, STAT4, ERCC6, TSPYL2, MEFV, RPL31, RPL34, CHD1, RSL24D1, THAP2, PHLDA1, NSA2, BCAS2, TCP1, RPL26, ANXA1, RPL24, COTL1, TUBA8, ID2, RPL22, RPL21, SVIL, SH3D19, MPHOSPH6	
GO:0070013~intracellular organelle lumen	9.75E-05	MRPS36, MEF2C, NCBP1, PTGS2, S100A9, SNRPD1,	2.41E-03

		WTAP, SRP19, CTNNB1, ISG20, EPC1, TARDBP, U2AF1, DDX21, PDHA1, GNL2, MRPL32, GTPBP4, MAGOH, HNRNPA2B1, MED10, JUNB, PCF11, RPS14, RPS13, SNRPG, HMGB1, HMGB2, MVD, MCL1, ANAPC10, RPS25, HNRNPA3, HNRNPL, TAL1, VRK1, HNRNPK, ZNF326, RPL9, RPL3, NPM3, HSPE1, RPL5, TAF9, UBE2D1, FH, NFE2, SUB1, SRA1, RYBP, NR4A1, NXF1, RPS6, DDX5, FOXP1, FXR1, RPS7, S100A12, PPIF, OASL, DNAJB9, HNRNPH2, HNRNPH1, KLF4, ATP5E, NBN, KDM6A, GAR1, EZH2, NFKB2, SENP5, FUBP1, ZNF350, GTF2A2, QKI, TWISTNB, TOP2B, MYC, INO80B, EXOSC8, CCNH, CCNL1, PRPF3, MBD2, GTF2B, C19ORF33, EIF4A3, ADRB2, LARP7, PSME4, RBM39, PMPCB, ING3, POLR2K, SHFM1, STAT4, TSPYL2, ERCC6, GTF3C6, THAP2, RSL24D1, PHLDA1, ETV3, NSA2, BCAS2, CEBPB, SOD2, PHF5A, SH3D19, MPHOSPH6, DUSP6	
GO:0005615~extracellular space	5.17E-04	IL16, LDLR, IGFBP7, IL19, TNFSF14, IL15, CXCL10, CFP, ISG15, SMPDL3A, TPT1, IL1B, IL15RA, NRG1, IL1A, GNLY, OSM, INHBA, RETN, THBD, EREG, PPBP, DEFA4, DEFA3, SERPINB2, TNFAIP2, CSF3, CXCL1, CSF2, CCL2, CXCL3, CXCL2, PF4, CCL7, ADA, IL23A, CCL20, FCN1, THBS1, SRGN, IL6, CMTM2, AIMP1, IL8, IL1RN, TNFSF8, TNFSF10, MPO, HBEGF, CMTM5	1.20E-02
GO:0015934~large ribosomal subunit	6.44E-04	RPL7, RPL31, RPL22, RPL34, RPL21, RPL3, RPL26, RPL37, RPL5, RPL24, MRPL32	1.41E-02

Cellular Component (CC_FAT) associated with genes having increased expression in **H_RM** with respect to H_Monocyte

Term	PValue	Genes	Benjamini
GO:0044421~extracellular region part	1.07E-06	CSF3, CSF2, A2M, CXCL5, C3, MMP9, APOC1, MMP3, MMP2, IL10, MMP1, ACE, APOE, GPX3, SEPP1, TFPI2, ADAM9, ANGPTL4, SPP1, LAD1, PAPLN, IL24, MMP14, ECM1, MMP10, INHBA, C1QB, CTSK, PLA2G7, GDF15, MFAP5, OTOA, IGFBP4	0.00
GO:0005576~extracellular region	1.85E-06	A2M, FAM20A, MMP9, C12ORF73, SPINK1, MMP3, C1QC, MMP2, IL10, MMP1, APOE, HAMP, GPX3, HTRA4, SEPP1, TFPI2, ADAM9, LAD1, OLFML2B, IL24, MMP14, VEGFB, INHBA, C1QB, MMP10, CTSK, IL18BP, PTGDS, PLA2G7, CTSD, TREM2, OTOA, MFAP5, CSF3, CSF2, DEFB124, CXCL5, C3, APOC1, ACE, PLTP, ANGPTL4, SPP1, RNASE1, PAPLN, ECM1, MUCL1, TFRC, CD59, GDF15, IGFBP4, ADAMDEC1	0.00
GO:0005615~extracellular space	1.26E-05	CSF3, CSF2, A2M, CXCL5, C3, MMP9, APOC1, MMP3, MMP2, IL10, ACE, APOE, GPX3, SEPP1, ADAM9, ANGPTL4, SPP1, IL24, C1QB, INHBA, MMP10, CTSK, PLA2G7, GDF15, IGFBP4	0.00
GO:0009897~external side of plasma membrane	4.45E-04	KCNMA1, KCNJ5, ACE, APOE, CD59, CD274, CD22, OTOA, ABCG1, ADAM9	0.03
GO:0005578~proteinaceous extracellular matrix	1.18E-03	LAD1, MMP9, PAPLN, MMP3, MMP14, MMP2, ECM1, MMP1, MMP10, MFAP5, OTOA, TFPI2, ANGPTL4	0.05
GO:0031012~extracellular matrix	2.23E-03	LAD1, MMP9, PAPLN, MMP3, MMP14, MMP2, ECM1, MMP1, MMP10, MFAP5, OTOA, TFPI2, ANGPTL4	0.08
GO:0005773~vacuole	6.58E-03	HOOK1, CTSK, CD68, NPC1, LGMN, ACP5, CTSD, IL4I1, FUCA1, ATP6V0D2	0.20
GO:0045177~apical part of cell	9.93E-03	KCNMA1, PDPN, HAMP, LGMN, SRR, OTOA, ATP6V0D2, SPP1	0.25

Cellular Component (CC_FAT) associated with genes having increased expression in **H_NeoHep** with respect to H_Monocyte

Term	PValue	Genes	Benjamini
GO:0042470~melanosome	0.00	TFRC, SLC2A1, CAPG, CTSD, CTSB, MMP14, GPNMB	0.06
GO:0048770~pigment granule	0.00	TFRC, SLC2A1, CAPG, CTSD, CTSB, MMP14, GPNMB	0.06
GO:0045177~apical part of cell	0.00	KCNMA1, P2RY6, PDPN, HAMP, LGMN, CTSB, ATP6V0D2, DPP4, SPP1	0.05
GO:0031988~membrane-bounded vesicle	0.00	PAM, A2M, BGLAP, CADM1, OPTN, MMP14, RACGAP1, VEGFB, DAB2, TFRC, CAPG, SLC2A1, CTSD, CTSB, GPNMB, SPP1	0.04
GO:0005773~vacuole	0.00	MTMR2, NPC1, PLA2G15, LGMN, ACP5, CTSD, CTSB, FUCA1, ATP6V0D2, CHIT1	0.06
GO:0005615~extracellular space	0.00	MIA, A2M, BGLAP, C3, IL6ST, MMP9, APOC1, MMP2, CHIT1, C1QA, C1QB, APOE, KCP, SEPP1, IGFBP4, SPP1, ADAM9	0.06
GO:0009897~external side of plasma membrane	0.00	KCNMA1, KCNJ5, APOE, IL6ST, CD59, CD274, CTSB, ADAM9	0.06
GO:0005576~extracellular region	0.00	MIA, PAM, A2M, IL6ST, C3, MMP9, APOC1, C1QC, MMP2, CHIT1, PVRL4, HAMP, APOE, SEPP1, DPP4, ADAM9, SPP1, RNASE1, BGLAP, PLA2G15, MMP14, ECM1, VEGFB, C1QA, C1QB, TFRC, PTGDS, CD59, KCP, CTSD, CTSB, TPSAB1, TREM2, IGFBP4	0.08
GO:0031982~vesicle	0.00	PAM, A2M, BGLAP, CADM1, OPTN, MMP14, RACGAP1, VEGFB, DAB2, TFRC, CAPG, SLC2A1, CTSD, CTSB, GPNMB, SPP1	0.08
GO:0016023~cytoplasmic membrane-bounded vesicle	0.00	PAM, A2M, CADM1, OPTN, MMP14, RACGAP1, VEGFB, DAB2, TFRC, CAPG, SLC2A1, CTSD, CTSB, GPNMB	0.09
GO:0000323~lytic vacuole	0.01	NPC1, PLA2G15, LGMN, ACP5, CTSD, CTSB, FUCA1, CHIT1	0.12
GO:0005764~lysosome	0.01	NPC1, PLA2G15, LGMN, ACP5, CTSD, CTSB, FUCA1, CHIT1	0.12
GO:0044421~extracellular region part	0.01	MIA, A2M, BGLAP, C3, IL6ST, MMP9, APOC1, MMP14, MMP2, ECM1, CHIT1, C1QA, C1QB, APOE, KCP, SEPP1, IGFBP4, SPP1, ADAM9	0.15
GO:0009986~cell surface	0.01	KCNMA1, KCNJ5, APOE, IL6ST,	0.15

		CD59, CD274, BMPR2, CTSB, DPP4, ADAM9	

Cellular Component (CC_FAT) associated with genes having increased expression in **HNP_RM** with respect to HNP_Monocyte

Term	PValue	Genes	Benjamini
GO:0005764~lysosome	0.08	CTSD, CHIT1	0.93
GO:0000323~lytic vacuole	0.08	CTSD, CHIT1	0.93
GO:0005773~vacuole	0.09	CTSD, CHIT1	0.80

Cellular Component (CC_FAT) associated with genes having increased expression in **HNP_NeoHep** with respect to HNP_Monocyte

Term	PValue	Genes	Benjamini
GO:0044421~extracellular region part	1.41E-06	UTS2, A2M, MSR1, C3, MMP9, OXT, CSF1, IGFBP6, APOC1, MMP2, CHIT1, GPC4, CCL22, ACE, LGALS3BP, APOA2, LAMB2, GPC3, APOE, SAA1, APOC3, GPX3, COL6A2, COL6A1, SEPP1, PRSS36, SPON2, C19ORF24, SPP1, FN1, LPL, BGLAP, CHI3L1, ECM1, COL5A1, C1QA, AFP, C1QB, CTSK, NAV2, KCP, LAMC1	0.00
GO:0005764~lysosome	3.75E-06	NAGLU, PLA2G15, LIPA, LGMN, ACP5, ACP2, FUCA1, CHIT1, C1ORF85, CTSK, SLC29A3, SLC48A1, CTSD, CTSB, MAN2B1, GBA, CTSF	0.00
GO:0000323~lytic vacuole	3.75E-06	NAGLU, PLA2G15, LIPA, LGMN, ACP5, ACP2, FUCA1, CHIT1, C1ORF85, CTSK, SLC29A3, SLC48A1, CTSD, CTSB, MAN2B1, GBA, CTSF	0.00
GO:0005773~vacuole	8.89E-06	NAGLU, PLA2G15, LIPA, LGMN, ACP5, ACP2, FUCA1, CHIT1, C1ORF85, CTSK, SLC29A3, SLC48A1, CTSD, CTSB, MAN2B1, ATP6V0D2, GBA, CTSF	0.00
GO:0005615~extracellular space	1.03E-05	UTS2, A2M, MSR1, C3, MMP9, CSF1, OXT, IGFBP6, APOC1, MMP2, CHIT1, GPC4, CCL22, LGALS3BP, APOA2, ACE, GPC3, SAA1, APOE, GPX3, APOC3, SEPP1, FN1, SPP1, LPL, BGLAP, CHI3L1, C1QA, AFP, C1QB, CTSK, KCP	0.00
GO:0005576~extracellular region	2.93E-05	UTS2, A2M, MSR1, MMP9, IGFBP6, MMP2, C1QC, GPC4, APOA2, C17ORF99, GPC3, SAA1, HAMP, APOE, GPX3, SEPP1, PRSS36, SPON2, APOA1BP, OLFML2B, SPINT1, GAL, VEGFB, C1QA, C1QB, CTSK, IL18BP, PTGDS, NAV2, KCP, CTSD, LAMC1, CTSB, TREM2, EDA, C3, CSF1, OXT, APOC1, NMB,	0.00

		CHIT1, CCL22, ACE, LGALS3BP, LAMB2, PLA2G12A, APOC3, COL6A2, COL6A1, C19ORF24, PLTP, SPP1, FN1, LPL, RNASE1, BGLAP, PLA2G15, CHI3L1, SEPN1, ECM1, COL5A1, AFP, TFRC, CD59, C11ORF45	
GO:0032994~protein-lipid complex	5.20E-05	LPL, APOA2, MSR1, SAA1, APOE, APOC3, APOC1	0.00
GO:0034358~plasma lipoprotein particle	5.20E-05	LPL, APOA2, MSR1, SAA1, APOE, APOC3, APOC1	0.00
GO:0042627~chylomicron	6.15E-05	LPL, APOA2, APOE, APOC3, APOC1	0.00
GO:0034361~very-low-density lipoprotein particle	5.33E-04	LPL, APOA2, APOE, APOC3, APOC1	0.02
GO:0034385~triglyceride-rich lipoprotein particle	5.33E-04	LPL, APOA2, APOE, APOC3, APOC1	0.02
GO:0045177~apical part of cell	7.70E-04	HAMP, LGMN, SRR, AKAP7, CDH1, CTSB, SLC39A4, EDA, ATP6V0D2, SPP1, FN1, ACVR1	0.03
GO:0031012~extracellular matrix	1.20E-03	LPL, MMP9, CHI3L1, MMP2, ECM1, COL5A1, GPC4, LGALS3BP, GPC3, LAMB2, NAV2, COL6A2, COL6A1, LAMC1, PRSS36, SPON2, FN1	0.04
GO:0034364~high-density lipoprotein particle	1.29E-03	APOA2, SAA1, APOE, APOC3, APOC1	0.04
GO:0005578~proteinaceous extracellular matrix	1.53E-03	MMP9, CHI3L1, MMP2, ECM1, COL5A1, GPC4, LGALS3BP, GPC3, LAMB2, NAV2, COL6A2, COL6A1, LAMC1, PRSS36, SPON2, FN1	0.04
GO:0048770~pigment granule	7.78E-03	TFRC, TRPV2, CAPG, CTSD, CTSB, GPNMB, GCHFR	0.17
GO:0042470~melanosome	7.78E-03	TFRC, TRPV2, CAPG, CTSD, CTSB, GPNMB, GCHFR	0.17
GO:0005625~soluble fraction	8.52E-03	PLA2G15, AARS, NMB, FUCA1, GPX3, SRR, ENO2, IDH1, HSPB1, FABP4, CTSB, MAN2B1, GCHFR, PC	0.17
GO:0009897~external side of plasma membrane	1.84E-02	KCNJ5, GPC4, P2RX7, ACE, GPC3, APOE, CD59, CD276, CTSB	0.32
GO:0000267~cell fraction	1.93E-02	MME, NMB, ACP2, SDC3, ACE, POMGNT1, GPX3, SRR, ENO2, VKORC1, IDH1, SCARB1, RAPGEF3, MAN2B1,	0.32

		GCHFR, SLC12A6, PLA2G15, VAC14, AARS, SPINT1, TSPAN15, FUCA1, ITPR2, P2RX7, CD59, USO1, TXNRD3, HSPB1, FABP4, CTSB, EDA, PC	
GO:0005602~complement component C1 complex	3.86E-02	C1QA, C1QB	0.51
GO:0043025~cell soma	4.63E-02	P2RX7, APOE, SLC12A5, UCHL1, ENO2, SRR, CYGB, NRSN2	0.56
GO:0005925~focal adhesion	4.90E-02	ARHGAP31, LIMK1, FAIM3, CDH1, TENC1, PARVA	0.56
GO:0031988~membrane-bounded vesicle	5.18E-02	A2M, BGLAP, TRPV2, OXT, GAL, VEGFB, AP1S1, TFRC, CAPG, CTSD, NRSN2, CTSB, GPNMB, SLC39A4, GCHFR, FN1, SPP1, MT3	0.56
GO:0031965~nuclear membrane	5.39E-02	P2RX7, PTGDS, DHCR7, SCD, GCHFR	0.56
GO:0005783~endoplasmic reticulum	5.44E-02	TM7SF2, TDRD6, HSD3B7, APOC1, EDEM3, SEZ6L2, APOA2, LAMB2, PLOD1, PLA2G12A, DHCR7, VKORC1, PPAP2B, DHCR24, VAC14, SELM, ACO1, SCD, SEPN1, PKD2L1, ITPR2, KIF1C, PTGDS, TXNRD3, ASPHD1, EDA, RCN1	0.55
GO:0005924~cell-substrate adherens junction	5.61E-02	ARHGAP31, LIMK1, FAIM3, CDH1, TENC1, PARVA	0.54
GO:0043260~laminin-11 complex	5.73E-02	LAMB2, LAMC1	0.54
GO:0030055~cell-substrate junction	6.78E-02	ARHGAP31, LIMK1, FAIM3, CDH1, TENC1, PARVA	0.58
GO:0034363~intermediate-density lipoprotein particle	7.57E-02	APOE, APOC3	0.61
GO:0030424~axon	9.08E-02	SLC12A6, P2RX7, OXT, UCHL1, DOCK7, ITPR2, SDC3	0.67

Molecular Function (MF_FAT) associated with genes having decreased expression in **H_RM** with respect to H_Monocyte

Term	PValue	Genes	Benjamini
GO:0001871~pattern binding	0.00	AZU1, SPOCK3, SELL, PGLYRP1, PF4, CLEC7A, PF4V1, PTX3, THBS1, NLRP3	0.08
GO:0030247~polysaccharide binding	0.00	AZU1, SPOCK3, SELL, PGLYRP1, PF4, CLEC7A, PF4V1, PTX3, THBS1, NLRP3	0.08
GO:0030246~carbohydrate binding	0.00	CLEC2L, SPOCK3, SELL, LGALS12, PGLYRP1, PF4, PF4V1, NLRP3, AZU1, ASGR2, FCN1, CLEC7A, THBS1, PTX3, CLEC1B	0.05
GO:0016566~specific transcriptional repressor activity	0.00	IKZF1, ZBTB16, HDAC9, TCF7L2, FOXP1	0.15
GO:0003700~transcription factor activity	0.00	MEF2C, VENTX, ZEB2, ZBTB16, TCF7L2, FOS, TAL1, JUND, MYC, FOSL1, MAFF, NFE2, IKZF1, NR4A2, NR4A1, CREB5, FOSB, MECOM, FOXP1, JUNB, MTF1, CSRNP1, IRF7, KLF2, SCAND1	0.20
GO:0005509~calcium ion binding	0.00	S100P, TNNC2, S100A8, SPOCK3, S100A9, MMP17, PADI4, S100Z, S100A12, GCH1, MYL9, SAG, ASGR2, F5, RASGRP4, PVALB, RASGRP2, FCN1, MPO, PLCB1, RAPGEF2, EHD1, THBS1, ITGA2B	0.16
GO:0005539~glycosaminoglycan binding	0.00	AZU1, SPOCK3, SELL, PGLYRP1, PF4, PF4V1, THBS1, NLRP3	0.15
GO:0043565~sequence-specific DNA binding	0.00	MEF2C, MAFF, NFE2, IKZF1, NR4A2, NR4A1, CREB5, VENTX, ZEB2, FOSB, JUNB, FOXP1, TAL1, FOS, JUND, FOSL1, MYC, KDM6B	0.15
GO:0030695~GTPase regulator activity	0.01	RASGEF1B, RGL4, RPH3A, RGS14, TAGAP, CDC42EP2, RGS1, RGS2, RASGRP4, RASGRP2, RAPGEF2, ARAP2, SYTL1	0.30
GO:0030528~transcription regulator activity	0.01	MEF2C, VENTX, PDLIM1, ZEB2, ZBTB16, TCF7L2, ZNF345, FOS, TAL1, GFI1B, JUND, NRG1, MYC, FOSL1, MAFF, NFE2, IKZF1, NR4A2, NR4A1, CREB5, FOSB,	0.31

		MECOM, JUNB, FOXP1, HES1, ATXN1, MTF1, IRF7, CSRNP1, HDAC9, KLF2, SCAND1	
GO:0060589~nucleoside-triphosphatase regulator activity	0.01	RASGEF1B, RGL4, RPH3A, RGS14, TAGAP, CDC42EP2, RGS1, RGS2, RASGRP4, RASGRP2, RAPGEF2, ARAP2, SYTL1	0.29
GO:0008047~enzyme activator activity	0.01	CDC42EP2, RGS1, AIFM3, RGS2, MMP17, GHRL, NRG1, RAPGEF2, ARAP2, NLRP1, RGS14	0.38
GO:0003702~RNA polymerase II transcription factor activity	0.02	MEF2C, FOS, GFI1B, IRF7, JUND, ZNF345, TCF7L2, JUNB, FOXP1	0.40
GO:0003704~specific RNA polymerase II transcription factor activity	0.02	FOS, GFI1B, IRF7, ZNF345	0.43
GO:0008009~chemokine activity	0.02	PPBP, PF4, PF4V1, CXCL10	0.47
GO:0001872~zymosan binding	0.03	CLEC7A, PTX3	0.49
GO:0042379~chemokine receptor binding	0.03	PPBP, PF4, PF4V1, CXCL10	0.48
GO:0046983~protein dimerization activity	0.03	NFE2, IKZF1, NR4A2, NR4A1, CREB5, ZBTB16, FOSB, FOXP1, JUNB, GCH1, FOS, CORO1A, JUND, FOSL1	0.47
GO:0004866~endopeptidase inhibitor activity	0.04	SERPINB9, SPOCK3, CARD16, CARD17, ITIH4, SLPI	0.61
GO:0005125~cytokine activity	0.05	CMTM2, PPBP, IL1RN, PF4, PF4V1, CXCL10, CMTM5	0.61
GO:0005529~sugar binding	0.05	ASGR2, CLEC2L, SELL, FCN1, LGALS12, CLEC7A, CLEC1B	0.61
GO:0008201~heparin binding	0.05	AZU1, SELL, PF4, PF4V1, THBS1	0.61
GO:0005085~guanyl-nucleotide exchange factor activity	0.05	TAGAP, RASGRP4, RASGRP2, RASGEF1B, RGL4, RAPGEF2	0.61
GO:0030414~peptidase inhibitor activity	0.05	SERPINB9, SPOCK3, CARD16, CARD17, ITIH4, SLPI	0.61
GO:0046872~metal ion binding	0.06	ADCY4, S100A8, TNNC2, S100A9, RNF217, ZEB2, PDLIM1, ZNF345, APOBEC3B, ASGR2, APOBEC3A, GFI1B, PVALB, MAP3K8, LTF, PLCB1, RAPGEF2, ZFP36, ZBTB20, ZCCHC10, AIFM3, MMP17, PADI4, RPH3A, MECOM, TCN1, SAG, MAST4, MTF1, F5, TNFAIP3, KDM6B, ALOX12,	0.62

		ITGA2B, SPOCK3, CA5B, ZBTB16, KCNJ2, ZNF780B, GCH1, MYL9, MEFV, RASGRP4, RASGRP2, FCN1, LIMD2, EHD1, THBS1, SCO2, CRIP1, S100P, IKZF1, ANKMY1, NR4A2, MTL5, NR4A1, CREB5, TET2, S100Z, SNAI1, FOXP1, S100A12, MPO, RNF19B, KLF2, ARAP2	
GO:0016814~hydrolase activity, acting on carbon-nitrogen (but not peptide) bonds, in cyclic amidines	0.06	APOBEC3B, APOBEC3A, GCH1	0.65
GO:0004857~enzyme inhibitor activity	0.07	SERPINB9, SPOCK3, CARD16, CDKN2D, CARD17, ITIH4, SLPI, SAG	0.66
GO:0043169~cation binding	0.07	ADCY4, S100A8, TNNC2, S100A9, RNF217, ZEB2, PDLIM1, ZNF345, APOBEC3B, ASGR2, APOBEC3A, GFI1B, PVALB, MAP3K8, LTF, PLCB1, RAPGEF2, ZFP36, ZBTB20, ZCCHC10, AIFM3, MMP17, PADI4, RPH3A, MECOM, TCN1, SAG, MAST4, MTF1, F5, TNFAIP3, KDM6B, ALOX12, ITGA2B, SPOCK3, CA5B, ZBTB16, KCNJ2, ZNF780B, GCH1, MYL9, MEFV, RASGRP4, RASGRP2, FCN1, LIMD2, EHD1, THBS1, SCO2, CRIP1, S100P, IKZF1, ANKMY1, NR4A2, MTL5, NR4A1, CREB5, TET2, S100Z, SNAI1, FOXP1, S100A12, MPO, RNF19B, KLF2, ARAP2	0.64
GO:0019899~enzyme binding	0.09	TAL1, CORO1A, CDC42EP2, DUSP2, CDKN2D, NFKBIA, PLCB1, HDAC9, RPH3A, SYTL1, TCF7L2, NLRP1	0.73
GO:0043167~ion binding	0.09	ADCY4, S100A8, TNNC2, S100A9, RNF217, ZEB2, PDLIM1, ZNF345, APOBEC3B, ASGR2, APOBEC3A, GFI1B, PVALB, MAP3K8, LTF, PLCB1, RAPGEF2, ZFP36, ZBTB20, ZCCHC10, AIFM3, MMP17, PADI4, RPH3A, MECOM, TCN1, SAG, MAST4, MTF1, F5,	0.71

		TNFAIP3, KDM6B, ALOX12, ITGA2B, SPOCK3, CA5B, ZBTB16, KCNJ2, ZNF780B, GCH1, MYL9, MEFV, RASGRP4, RASGRP2, FCN1, LIMD2, EHD1, THBS1, SCO2, CRIP1, S100P, IKZF1, ANKMY1, NR4A2, MTL5, NR4A1, CREB5, TET2, S100Z, SNAI1, FOXP1, S100A12, MPO, RNF19B, KLF2, ARAP2	
GO:0019887~protein kinase regulator activity	0.09	CDKN2D, GHRL, NRG1, CXCL10	0.72

Molecular Function (MF_FAT) associated with genes having decreased expression in **H_NeoHep** with respect to H_Monocyte

Term	PValue	Genes	Benjamini
GO:0003723~RNA binding	9.02E-16	RPL18, NCBP1, SRP14, RPL36A, LSM6, RBM3, RPL15, UNC50, ISG20, NONO, APOBEC3B, EIF1AY, U2AF1, LSM5, RBMS2, DDX10, LUC7L3, SNRPA1, RPL35A, SF3B14, MAGOH, HNRNPA2B1, RSL1D1, PSMA1, AQR, RPS16, TIAL1, RPS14, EIF2S1, RPS13, SLU7, SNRPG, FUS, AGFG1, FARS2, SNRPB2, OAS3, OAS2, NAA38, RPS25, HNRNPL, HNRNPM, HNRNPK, RPL7, RPL6, RPL9, HNRNPF, DDX3Y, HNRNPD, RPL3, PABPC3, RPL5, HNRNPC, PABPC1, RPL7A, RPS20, DHX58, HNRNPAB, SSB, NXF1, HNRNPA0, RPF1, EIF4B, TRNT1, HNRNPH3, OASL, HNRNPH2, EIF4E, DDX59, RNPC3, CWC15, MKI67IP, EIF2A, PCBP1, MAGOHB, QKI, ZFP36, MRPL1, EXOSC8, EXOSC9, MRPL3, RBM45, EXOSC5, EXOSC3, MBD2, RPS4X, TARBP1, WRAP53, FARSB, CPSF6, RBM39, CPSF3, SRP9, RBM34, TRA2B, TRA2A, RPS15A, RPL37, RPL38, ZFP36L1, KIAA0020, ZFP36L2, RPL31, RPL34, MRPL16, MSI2, PINX1, ZBP1, TTC14, AIMP1, RPL26, RPL24, ATXN1, HSP90B1, SON, MRPL21, ILF2, RPL22, RPL21, RBM19, NOP58, RBM14, RBM17	9.17E-13
GO:0003735~structural constituent of ribosome	9.34E-13	MRPS36, RPL18, RPL17, MRPL42, RPL36A, RPL15, RPS15A, RPL37, RPL38, RPS27, RPL7, RPL31, RPL6, RPL9, RPL34, MRPL16, RPL3, MRPL19, MRPL18, RPL5,	4.82E-10

		RPL7A, RPS20, RPS21, MRPL32, MRPL33, RPS23, RPS24, RPL36AL, MRPL1, RPL35A, MRPL3, RPL26, RPL27, RPL24, RPS6, RPS4X, RSL1D1, MRPL21, HNRNPH2, RPS16, RPL22, RPL13A, RPL21, RPS14, RPS13, UBB	
GO:0043028~caspase regulator activity	3.02E-04	TNFRSF10B, TNFAIP8, SNCA, NLRP12, TNFSF14, PRDX3, HSPA5, BCL2L13, CASP1, NLRP1	9.88E-02
GO:0003729~mRNA binding	6.15E-04	ZFP36, SSB, NXF1, MBD2, ZFP36L1, RPL7, RPS14, HNRNPD, SLU7, RPS13, PABPC3, HNRNPC, PABPC1, LUC7L3, HNRNPAB	1.47E-01
GO:0003924~GTPase activity	1.46E-03	RAB9A, GNAI2, RHOQ, RRAD, GNG11, ARF5, RHOU, RAB1A, GNG8, RAC2, RAB28, EHD1, MX2, RAB27A, RAB2A, GTPBP1, GTPBP4, DNM1L, GBP5, RAP2C, NCF1, RAB4A, RASL11A, GNB2, TUBA4A, RAP1A, ARL8B, GBP4, GBP3, GBP2, GBP1	2.61E-01
GO:0016566~specific transcriptional repressor activity	6.61E-03	EID1, IKZF1, SMAD3, NFKB1, ZBTB16, HDAC9, TCF7L2, AKIRIN2, FOXP1	6.81E-01
GO:0004869~cysteine-type endopeptidase inhibitor activity	7.85E-03	CARD16, TNFAIP8, SNCA, CARD17, TNFSF14, CSTA, PRDX3, HSPA5, PTTG1	6.88E-01
GO:0003743~translation initiation factor activity	8.42E-03	EIF4B, EIF4E, EIF3H, EIF2S1, EIF3E, EIF1AY, EIF3F, EIF2A, EIF1B, MTIF3, GTF2B, EIF3M	6.64E-01
GO:0043027~caspase inhibitor activity	8.55E-03	TNFAIP8, SNCA, TNFSF14, PRDX3, HSPA5	6.27E-01
GO:0008009~chemokine activity	9.71E-03	PPBP, IL8, CCL20, CXCL3, CXCL2, CKLF, PF4, PF4V1, CCL7, CXCL10	6.35E-01
GO:0008134~transcription factor binding	1.06E-02	ZNF85, ENY2, NBN, HMGN3, TSG101, PDLIM1, NFKB2, CITED4, RAB1A, CITED2, KDM1A, GTF2A2, BRD7, RARA, NFIL3, USP16, NRG1, TBPL1, SERTAD2, ZFX, FOSB, CDK7, JUNB, AHR, MXD4, BTG1, NCOA6, SIAH2, SUPT3H, EID1,	6.32E-01

		YWHAZ, NMI, NFKBIA, NFYC, TCF7L2, TRIB1, DRAP1, BCL11A, BCL3, PEX14, MYCBP, TAF9, APEX1, NFE2, MAFB, SUB1, CREB1, CEBPG, SMAD3, OASL, HDAC3, MED30, HDAC1, GSK3B, JAZF1, HDAC9, RBM14	
GO:0000175~3'-5'-exoribonuclease activity	1.20E-02	EXOSC8, EXOSC9, EXOSC5, EXOSC3, ISG20	6.47E-01
GO:0005125~cytokine activity	1.29E-02	IL16, CXCL3, CXCL2, TNFSF14, PF4, IL15, PF4V1, CCL7, CXCL10, IL23A, CCL20, CKLF, IL1B, IL1A, SECTM1, IL6, CMTM2, IL8, AIMP1, IL1RN, TNFSF8, OSM, TNFSF10, PPBP, CMTM6, CMTM5	6.43E-01
GO:0050700~CARD domain binding	1.40E-02	NOD2, CARD9, C9ORF89, RIPK2	6.48E-01
GO:0042379~chemokine receptor binding	1.46E-02	PPBP, IL8, CCL20, CXCL3, CXCL2, CKLF, PF4, PF4V1, CCL7, CXCL10	6.37E-01
GO:0004532~exoribonuclease activity	1.63E-02	EXOSC8, EXOSC9, EXOSC5, EXOSC3, ISG20	6.54E-01
GO:0016896~exoribonuclease activity, producing 5'-phosphomonoesters	1.63E-02	EXOSC8, EXOSC9, EXOSC5, EXOSC3, ISG20	6.54E-01
GO:0016796~exonuclease activity, active with either ribo- or deoxyribonucleic acids and producing 5'-phosphomonoesters	1.84E-02	RAD1, EXOSC8, EXOSC9, EXOSC5, EXOSC3, ISG20	6.76E-01
GO:0016564~transcription repressor activity	2.04E-02	ZNF85, EID1, HMGB2, TSG101, EZH2, CBX3, ZNF675, NFKB1, ZBTB16, TCF7L2, CITED2, EPC1, KDM1A, DRAP1, BCL11A, PEX14, BHLHE40, NFIL3, APEX1, AKIRIN2, IKZF1, SMAD3, MBD2, RBBP7, JUNB, FOXP1, MXD4, HES1, ATXN1, HDAC3, ID2, NAB1, JAZF1, HIVEP1, SIAH2, HDAC9, KLF4	6.93E-01
GO:0008408~3'-5' exonuclease activity	2.13E-02	RAD1, EXOSC8, EXOSC9, EXOSC5, EXOSC3, APEX1, ISG20	6.90E-01
GO:0000049~tRNA binding	2.25E-02	TRNT1, RPL35A, AIMP1,	6.92E-01

		FARS2, EIF2A, SSB	
GO:0019787~small conjugating protein ligase activity	2.44E-02	UBE2A, TSG101, UBE2J1, RNF217, ANAPC10, UBE2J2, UBE2Q2, ATG3, UBE2Q1, RBBP6, RBX1, SUMO2, ARIH1, UBE2E3, RNF7, UBE2M, FBXL5, SIAH2, UBE2D1, FBXL3, UBE2E2, UBE2E1	7.03E-01
GO:0015078~hydrogen ion transmembrane transporter activity	2.66E-02	ATP5E, COX7B, ATP5F1, COX7C, COX7A2L, UQCRFS1, COX6C, UQCRH, ATP5S, ATP5L, ATP5A1, ATP5H, UQCRB, ATP5J	7.18E-01
GO:0042834~peptidoglycan binding	2.98E-02	NOD2, PGLYRP1, TLR2, NLRP3	7.44E-01
GO:0016814~hydrolase activity, acting on carbon-nitrogen (but not peptide) bonds, in cyclic amidines	3.39E-02	DCTD, APOBEC3B, APOBEC3A, CDA, AMPD2, ZBP1, GCH1	7.74E-01
GO:0008656~caspase activator activity	3.42E-02	TNFRSF10B, NLRP12, BCL2L13, CASP1, NLRP1	7.63E-01
GO:0019788~NEDD8 ligase activity	3.45E-02	RNF7, UBE2M, RBX1	7.52E-01
GO:0015077~monovalent inorganic cation transmembrane transporter activity	3.76E-02	ATP5E, COX7B, ATP5F1, COX7C, ATP1A1, COX7A2L, UQCRFS1, COX6C, UQCRH, ATP5S, ATP5L, ATP5A1, ATP5H, UQCRB, ATP5J	7.69E-01
GO:0004866~endopeptidase inhibitor activity	4.14E-02	SPOCK3, SNCA, TNFSF14, SERPING1, PTTG1, PRDX3, SERPINB9, CARD16, SPINT2, CPAMD8, TNFAIP8, CARD17, ITIH4, SERPINB2, SERPINB1, SLPI, SERPINA1, HSPA5, CSTA	7.90E-01
GO:0043566~structure-specific DNA binding	4.14E-02	EGR1, KLF6, HMGB2, SUB1, CEBPG, CREB1, HNRNPA2B1, SMAD3, ZNF12, WBP11, MLH3, ZBTB16, JUNB, MCM7, PCBP1, POLG2, MYC, KLF4, ZBP1	7.90E-01
GO:0004857~enzyme inhibitor activity	4.20E-02	SPOCK3, SNCA, CDC42SE1, TNFSF14, PRDX3, PTTG1, TRIB1, SPINT2, CDKN2D, ITIH4, SERPINA1, HSPA5, PINX1, TESC, SOCS3, ANXA1, SERPING1, ANXA5, ANXA3, SAG, SERPINB9,	7.83E-01

		CARD16, CPAMD8, GMFG, TNFAIP8, CARD17, SERPINB2, SLPI, SERPINB1, ATPIF1, CSTA	
GO:0005525~GTP binding	4.98E-02	RAB9A, GNAI2, GTPBP10, RHOQ, RRAGA, RRAD, ARF5, RHOU, RRAGB, RAB1A, EFCAB4B, GCH1, ARL11, RAC2, RAB28, RAB24, SAR1B, EHD1, ARL5B, MX2, RAB27A, RAB2A, GIMAP2, GTPBP1, GIMAP4, GTPBP4, DNM1L, RAP2C, GBP5, NCF1, RAB4A, RASL11A, RAB37, TUBA4A, RAP1A, ARL8B, GBP4, GBP3, GBP2, GBP1	8.28E-01
GO:0004536~deoxyribonuclease activity	5.06E-02	RAD1, MUS81, TATDN2, APEX1, TATDN3, DNASE1L1, ISG20	8.23E-01
GO:0043014~alpha-tubulin binding	5.20E-02	B4GALT1, SNCA, ARL8B, C9ORF24	8.22E-01
GO:0003702~RNA polymerase II transcription factor activity	5.38E-02	HMGN1, MEF2C, SUPT3H, TAF1C, NFYC, ZNF345, TCF7L2, GTF2E2, GFI1B, GTF2A2, BTF3, RUNX2, TBPL1, CEBPB, CREB1, SMAD3, GTF2B, MED10, FOXP1, JUNB, MED31, TAF13, MED30, ILF2, TIAL1, IRF8, RBM14, KLF4	8.23E-01
GO:0003712~transcription cofactor activity	5.40E-02	ENY2, ZNF85, SUPT3H, EID1, NMI, TSG101, NFYC, PDLIM1, NFKB2, CITED4, CITED2, DRAP1, GTF2A2, BCL11A, PEX14, RARA, MYCBP, TAF9, NRG1, USP16, NFIL3, APEX1, TBPL1, SERTAD2, NFE2, SUB1, CREB1, ZFX, CDK7, JUNB, MXD4, HDAC3, MED30, BTG1, NCOA6, JAZF1, SIAH2, RBM14, HDAC9	8.15E-01
GO:0016413~O-acetyltransferase activity	5.45E-02	GNPAT, CRAT, LPCAT2	8.08E-01
GO:0030246~carbohydrate binding	5.74E-02	SPOCK3, LTBP4, TLR2, PGLYRP1, PF4, PF4V1, CLEC10A, CCL7, AZU1, ASGR2, NOD2, CD44, CD93, CLEC4E, FCN1, CLEC2D,	8.16E-01

		CLEC4A, CLEC4D, PTX3, THBS1, CLEC2L, OLR1, SELL, LGALS2, LGALS12, FBP1, SIGLEC14, NLRP3, MANBA, LGALS9, TNFAIP6, CLEC12A, SIGLEC5, RPL22, VCAN, CLEC7A, CD302, CLEC1B	
GO:0005198~structural molecule activity	5.80E-02	MRPS36, RPL18, RPL17, TLN1, MRPL42, RPL36A, VAPA, RPL15, VCL, ACTG1, COPB1, MSN, MRPL32, MRPL33, RPL36AL, MRPL1, RPL35A, MRPL3, RPS4X, RSL1D1, JUP, NCK2, RPS16, RPS14, TUBA4A, RPS13, CSTA, UBB, LMNB1, VIM, RPS15A, RPL37, RPL38, TPM1, MYL9, RPS27, RPL7, ARPC3, RPL31, RPL6, RPL9, RPL34, MRPL16, RPL3, MRPL19, MRPL18, RPL5, RPL7A, RPS20, RPS21, THBS1, RPS23, RPS24, ANXA1, RPL26, RPL27, RPL24, RPS6, MRPL21, HNRNPH2, RPL13A, RPL22, RPL21, MAPK8IP3	8.11E-01
GO:0016407~acetyltransferase activity	6.19E-02	SAT1, SUPT3H, EPC1, ING3, NAA20, NAT1, GNPAT, HAT1, TAF9, CRAT, LPCAT2, ACAT1	8.24E-01
GO:0030414~peptidase inhibitor activity	6.39E-02	SPOCK3, SNCA, TNFSF14, SERPING1, PTTG1, PRDX3, SERPINB9, CARD16, SPINT2, CPAMD8, TNFAIP8, CARD17, ITIH4, SERPINB2, SERPINB1, SLPI, SERPINA1, HSPA5, CSTA	8.26E-01
GO:0005529~sugar binding	6.42E-02	CLEC2L, OLR1, SELL, LGALS2, LGALS12, FBP1, SIGLEC14, CLEC10A, MANBA, LGALS9, ASGR2, CLEC12A, CD93, CLEC4E, SIGLEC5, FCN1, CLEC2D, CLEC4A, VCAN, CLEC7A, CLEC4D, CD302, CLEC1B	8.20E-01
GO:0000166~nucleotide binding	6.71E-02	LDHB, DYNC1LI1, CLK1, AURKB, RAB1A, HIBADH, BTK, TPK1, ACTG1, ATP2B1,	8.26E-01

		NONO, MAP3K4, CLK3, RAB28, LTB4R, MAP3K8, U2AF1, RAB24, MRPL39, DDX10, MAP2K6, RAB27A, GTPBP1, GTPBP4, GBP5, NCF1, SF3B14, UBE2J1, PIM3, PIM2, UBE2J2, MAPK6, TUBA4A, ARL8B, HLA-DRA, FUS, GNAI2, FARS2, STK17B, OAS3, ARF5, OAS2, VRK1, RAC2, PABPC3, PABPC1, DHX58, RPS24, HNRNPAB, MAP2K3, NXF1, HNRNPA0, OASL, GK, SPG7, GTPBP10, ZNF12, CRCP, MLH3, NOD2, NUBP1, MCCC1, ARL5B, RAP2C, ACTA2, RAB4A, RBM45, GRHPR, DARS2, MBD2, MAST4, CBWD1, ADK, FARSB, CDK11B, RBM39, SLC27A3, CBWD5, RBM34, TFG, GCH1, GPHN, ACSL1, HK3, DHX15, ABCD3, ACTL6A, ETFA, CSNK1A1, RAB2A, ATP1A1, NADK, SPATA5L1, ILF2, RAB37, FYN, GSK3B, NLRP12, RBM19, ATP5A1, RBM14, PGK1, RBM17, RPL36A, RAB9A, RBM3, DAK, RRAD, KDM1A, ARL11, RBMS2, STK39, SAR1B, MX2, STK25, CSNK1G2, HNRNPA2B1, RBKS, STK3, CDKL1, TIAL1, RIPK2, GBP4, GBP3, PCCA, GBP2, GBP1, MVD, FGR, SNRPB2, RRAGA, RRAGB, HNRNPL, HNRNPM, HNRNPF, DDX3Y, HNRNPD, TAF9, HNRNPC, UBE2D1, RUNX2, SMCHD1, MYO1G, SSB, ABCB7, EIF4B, TRNT1, UBE2E3, HNRNPH3, PLK3, HNRNPH2, P2RX1, PRKAR1B, DDX59, RAP1A, CARS2, RNPC3, UBE2E2, UBE2E1, RHOQ, MKI67IP, RHOU, TOR3A, MCM7, ACTR1B, POLG2, DUS1L, IRAK2, UBE2A, LIMK2,	

		CDK7, NLRP3, PRKCD, NLRP1, COQ6, TOR2A, UBE2M, CPSF6, TRA2B, TRA2A, EFCAB4B, TRIB1, MTHFS, MAP3K3, CAMK2D, MSI2, HSPA5, UCK2, EHD1, DUS3L, GIMAP2, GIMAP4, DNM1L, HCK, UBE2Q2, UBE2Q1, HSP90B1, RASL11A, JAK1, JAK3	
GO:0032561~guanyl ribonucleotide binding	6.84E-02	RAB9A, GNAI2, GTPBP10, RHOQ, RRAGA, RRAD, ARF5, RHOU, RRAGB, RAB1A, EFCAB4B, GCH1, ARL11, RAC2, RAB28, RAB24, SAR1B, EHD1, ARL5B, MX2, RAB27A, RAB2A, GIMAP2, GTPBP1, GIMAP4, GTPBP4, DNM1L, RAP2C, GBP5, NCF1, RAB4A, RASL11A, RAB37, TUBA4A, RAP1A, ARL8B, GBP4, GBP3, GBP2, GBP1	8.25E-01
GO:0019001~guanyl nucleotide binding	6.84E-02	RAB9A, GNAI2, GTPBP10, RHOQ, RRAGA, RRAD, ARF5, RHOU, RRAGB, RAB1A, EFCAB4B, GCH1, ARL11, RAC2, RAB28, RAB24, SAR1B, EHD1, ARL5B, MX2, RAB27A, RAB2A, GIMAP2, GTPBP1, GIMAP4, GTPBP4, DNM1L, RAP2C, GBP5, NCF1, RAB4A, RASL11A, RAB37, TUBA4A, RAP1A, ARL8B, GBP4, GBP3, GBP2, GBP1	8.25E-01
GO:0005048~signal sequence binding	7.07E-02	SRP14, BRE, NFKBIA, KDELR1, PEX7	8.28E-01
GO:0070003~threonine-type peptidase activity	7.07E-02	PSMA2, PSMB4, PSMA1, PSMA5, PSMA7	8.28E-01
GO:0004298~threonine-type endopeptidase activity	7.07E-02	PSMA2, PSMB4, PSMA1, PSMA5, PSMA7	8.28E-01
GO:0010860~proteasome regulator activity	7.74E-02	PSMF1, PSME1, PSME2	8.49E-01
GO:0043498~cell surface binding	7.78E-02	CD48, AIMP1, F3, ATP5A1, ANXA5, THBS1	8.44E-01
GO:0019843~rRNA binding	7.78E-02	RPL9, MRPL16, RPL37, RPL5, RPS4X, RPF1	8.44E-01
GO:0033549~MAP kinase phosphatase activity	8.00E-02	DUSP5, DUSP2, DUSP1, DUSP6	8.46E-01

GO:0017017~MAP kinase tyrosine/serine/threonine phosphatase activity	8.00E-02	DUSP5, DUSP2, DUSP1, DUSP6	8.46E-01
GO:0019899~enzyme binding	8.18E-02	DENND5A, HINT1, PDLIM5, CBX3, PRDX3, RAB3IP, NOD2, CDKN2D, RANBP3, SERPINA1, PLCB1, UBE2A, NCF2, PLK1S1, RPH3A, PRKCD, NLRP1, PLAUR, LILRB1, JUP, CCND3, BTG1, F3, ATPIF1, AKAP8, CSTA, GRASP, EID1, ADAMTSL4, DIAPH2, VIM, NFKBIA, TCF7L2, TRIB1, TAL1, CDC42EP2, HK3, PLEK, SMAD3, COTL1, ATG3, PTPN11, CORO1A, HDAC3, DUSP2, HDAC1, GSK3B, MAPK8IP3, RAP1A, SYTL3, HDAC9, SYTL1	8.47E-01
GO:0005484~SNAP receptor activity	8.21E-02	SNAP29, NPEPL1, STX7, STX11, STX10	8.42E-01
GO:0016881~acid-amino acid ligase activity	8.30E-02	UBE2A, TSG101, UBE2J1, RNF217, TTLL5, ANAPC10, UBE2J2, UBE2Q2, ATG3, UBE2Q1, RBBP6, RBX1, SUMO2, ARIH1, UBE2E3, RNF7, UBE2M, FBXL5, SIAH2, UBE2D1, FBXL3, UBE2E2, UBE2E1	8.39E-01
GO:0003713~transcription coactivator activity	9.07E-02	ENY2, SUPT3H, NFE2, SUB1, TSG101, ZFX, NFYC, PDLIM1, CDK7, NFKB2, CITED4, JUNB, CITED2, MED30, GTF2A2, NCOA6, RARA, MYCBP, TAF9, USP16, RBM14, APEX1, TBPL1, SERTAD2	8.60E-01
GO:0008135~translation factor activity, nucleic acid binding	9.16E-02	EEF1B2, EIF1B, EIF2A, GTF2B, MTIF3, EIF4B, EIF4E, EIF3H, EIF3E, EIF2S1, EIF1AY, EIF3F, EIF3M	8.57E-01
GO:0032813~tumor necrosis factor receptor superfamily binding	9.78E-02	BID, TNFSF10, MYD88, BRE, TNFSF14, TNFSF8	8.71E-01

Molecular Function (MF_FAT) associated with genes having decreased expression in **HNP_RM** with respect to HNP_Monocyte

Term	PValue	Genes	Benjamini
GO:0003735~structural constituent of ribosome	3.31E-38	RPL18, MRPS36, RPL17, MRPS33, RPL19, RPL14, RPL13, RPL15, RPLP2, RPS27L, RPL22L1, RPLP0, RPLP1, RPL10, FAU, RPL11, RPL12, MRPL33, RPS27A, RPL36AL, RPL35A, MRPL52, MRPL51, MRPS18C, RPS19, RPS16, MRPS18A, RPS14, RPS15, RPS12, RPS13, MRPL47, RPS11, MRPL48, UBA52, MRPS15, MRPS11, RPS27, RPS28, RPL7, RPS29, RPL6, RPL9, RPL8, RPL3, MRPL55, RPL5, RPL10A, RPL7A, RPL4, RPS20, RPS21, RPS23, RPS24, RPSA, MRPS24, RPS9, RPL23A, RPS6, RPS5, RPS8, RPS7, HNRNPH2, RPL18A, RPL37A, RPS2, RPS3, RPS3A, MRPS6, RPS4X, RPL41, UBC, UBB, RPL35, RPL27A, RPS15A, RPL36, RPL37, RPL38, MRPL20, CYLD, RPL30, RPL32, MRPL14, RPL31, RPL34, MRPL18, RSL24D1, RPL26, RPL27, RPL24, RPL28, RPL29, MRPL23, MRPL21, RPL23, MRPL28, RPL13A, RPL22	4.86E-35
GO:0003723~RNA binding	2.25E-30	RPL18, NAF1, RPL19, RPL14, RPL13, U2AF2, RPL15, LSM7, RPLP2, ISG20, NONO, DDX17, RPLP0, RPLP1, EIF1AY, U2AF1, FAU, LSM4, RPL11, RPL12, LUC7L3, YARS, SF3B14, RRP7A, PTBP1, RPS19, RPS16, RPS14, RPS13, RPS11, MCTS1, FUS, FIP1L1, NHP2L1, FARS2, PABPC4, ZCRB1, OAS1, CALR, NAA38, RPS25, HNRNPA3, MOV10, DDX60L, PABPC3, SUPV3L1, RPS20, PABPC1, DHX58, HNRNPAB, DHX9, RPS9, NXF1, DDX5, U2AF1L4, HNRNPA1, RPS5, HNRNPA0, FXR1, RPS7, OASL, RPL18A, DCP2, POP4, ADAR, GTF3A,	1.65E-27

		CWC15, SPI1, EIF2A, YBX1, QKI, SYNJ2, ZFP36, EXOSC8, SF1, EXOSC1, MBD2, EIF4A3, RPL41, EIF4A2, EIF4A1, RBM39, THOC2, POLR2G, RBM34, RPL35, RPL27A, RPL37, RPL38, KIN, MRPL20, ZFP36L1, RPL30, RPL31, RPL34, RNASET2, CSDE1, SNRNP70, MARS, RBM22, RBM23, RPL26, RPL24, ILF3, ANKHD1, ETF1, RPL28, TRIM21, RPL29, MRPL23, PAPOLA, MRPL21, ILF2, RPL22, SFPQ, NOP58, NOP56, RBM17, RALY, SRP14, NCBP1, ZC3HAV1, RBM3, RBM4, RBM5, SNRPD1, EIF5A, RBM6, SRP19, WIBG, SRRM2, TARDBP, DDX24, PABPN1, SNRPA1, RPL35A, MAGOH, FMR1, HNRNPA2B1, HNRNPU, SLTM, PSMA1, PSMA6, TIAL1, SNRPB, CELF2, SNRPA, SLU7, CELF1, SNRPC, SNRPF, SNRPE, SNRPG, CNBP, HDLBP, AGFG1, SNRPB2, DAZAP1, HNRNPL, HNRNPM, EIF3B, HNRNPK, DDX49, DDX3X, EIF3G, RPL7, RPL6, RPL9, HNRNPF, RPL8, NPM1, HNRNPD, RPL3, RPL5, RPL7A, RPL10A, HNRNPC, RPL4, DDX41, EWSR1, RNPS1, RPL23A, RBMX, SAFB2, EIF4B, DDX56, HNRNPH3, HNRNPH2, EIF4E, EIF4H, CPNE1, CIRBP, HNRNPH1, EIF4E2, PUF60, MKI67IP, RPS2, PDCD4, RPS3, FUBP1, MAZ, DKC1, RPS3A, PCBP1, PCBP2, LRRFIP1, SARS, MRPS6, MBNL1, RPS4X, NCL, SARNP, CPSF6, MATR3, CPSF1, TRA2B, TRA2A, RPS15A, EXOSC10, PRPF8, NUDT21, MSI2, FBL, ATXN1, HSP90B1, SON, ZRANB2, PSPC1	
GO:0016651~oxidoreductase activity, acting on NADH or NADPH	6.89E-15	NDUFB3, CYB5R3, NDUFB4, NDUFB5, CYB5R2, GMPR2, NDUFB7, NDUFB8, NDUFB9, NDUFAB1, KMO, NDUFB1, NDUFAF2, NDUFB2, NDUFS6,	3.37E-12

		NDUFS5, NDUFS4, GSTO1, NDUFS3, NDUFS2, NQO2, NDUFA4, NDUFA5, NDUFA2, NDUFA3, NDUFB10, NCF2, NDUFA9, NDUFA6, NDUFA7, NDUFC2, NDUFA13, NDUFC1, NDUFA10, ECSIT, GMPR, NDUFA1, NDUFA12, POR, CYBA, CYBB, TXNDC17, NDUFV1, TXNRD2	
GO:0003954~NADH dehydrogenase activity	1.43E-14	NDUFB3, NDUFB4, NDUFB5, NDUFB7, NDUFB8, NDUFB9, NDUFAB1, NDUFB1, NDUFAF2, NDUFB2, NDUFS6, NDUFS5, NDUFS4, NDUFS3, NDUFS2, NDUFA4, NDUFA5, NDUFA2, NDUFA3, NDUFB10, NDUFA9, NDUFA6, NDUFA7, NDUFC2, NDUFA13, NDUFC1, NDUFA10, NDUFA1, NDUFA12, NDUFV1	5.26E-12
GO:0008137~NADH dehydrogenase (ubiquinone) activity	1.43E-14	NDUFB3, NDUFB4, NDUFB5, NDUFB7, NDUFB8, NDUFB9, NDUFAB1, NDUFB1, NDUFAF2, NDUFB2, NDUFS6, NDUFS5, NDUFS4, NDUFS3, NDUFS2, NDUFA4, NDUFA5, NDUFA2, NDUFA3, NDUFB10, NDUFA9, NDUFA6, NDUFA7, NDUFC2, NDUFA13, NDUFC1, NDUFA10, NDUFA1, NDUFA12, NDUFV1	5.26E-12
GO:0050136~NADH dehydrogenase (quinone) activity	1.43E-14	NDUFB3, NDUFB4, NDUFB5, NDUFB7, NDUFB8, NDUFB9, NDUFAB1, NDUFB1, NDUFAF2, NDUFB2, NDUFS6, NDUFS5, NDUFS4, NDUFS3, NDUFS2, NDUFA4, NDUFA5, NDUFA2, NDUFA3, NDUFB10, NDUFA9, NDUFA6, NDUFA7, NDUFC2, NDUFA13, NDUFC1, NDUFA10, NDUFA1, NDUFA12, NDUFV1	5.26E-12
GO:0016655~oxidoreductase activity, acting on NADH or NADPH, quinone or similar compound as acceptor	2.35E-13	NDUFB3, NDUFB4, NDUFB5, NDUFB7, NDUFB8, NDUFB9, NDUFAB1, NDUFB1, NDUFAF2, NDUFB2, NDUFS6, NDUFS5, NDUFS4, GSTO1, NDUFS3, NDUFS2, NDUFA4, NDUFA5, NDUFA2, NDUFA3,	6.90E-11

		NDUFB10, NDUFA9, NDUFA6, NDUFA7, NDUFC2, NDUFA13, NDUFC1, NDUFA10, NDUFA1, NDUFA12, NDUFV1	
GO:0015078~hydrogen ion transmembrane transporter activity	1.39E-12	ATP5D, ATP5E, ATP6V0E1, UQCRC1, ATP5B, ATP6AP1, COX7B, COX7C, ATP5G2, ATP5G1, ATP6V1B2, COX7A2L, ATP6V1G1, UQCRFS1, COX5A, UQCRQ, COX5B, ATP5G3, ATP6V0B, ATP6V0C, UQCR11, COX6B1, ATP5L, ATP5O, ATP5I, ATP5H, ATP5J, TCIRG1, COX7A2, COX8A, ATP5F1, COX4I1, ATP6V1H, ATP6V1F, COX6C, UQCRH, ATP6V1E1, ATP5C1, COX6A1, ATP5A1, SURF1, UQCRB, SLC9A1	3.40E-10
GO:0003924~GTPase activity	4.47E-11	SEPT5, GNA15, HRAS, RAB5C, EIF5, RHOQ, RHOU, RAB1A, CDC42, RAB28, SRPR, RHOA, TUBB6, RALA, RHOC, GNL2, TUBA1A, MX1, GNG5, RHOF, MX2, TUBA1B, SAR1A, AGAP3, RHOG, TUBA1C, GTPBP2, GTPBP1, GTPBP4, GBP5, NUDT1, RAP2C, NCF1, RAN, EFTUD2, RAB4B, EIF2S3, EEF2, GNB2, GNB1, TUBA4A, GNAS, ARL8A, ARL8B, RAB13, GBP4, ARL4C, GBP2, GBP1, SEPT9, TUFM, RAB7A, GNAI2, ARF6, ARF5, RAC2, RAC1, RAB11A, EHD1, EHD4, RAB2A, EEF1A1, RAB8B, RAB31, ARF3, RAB34, ARF4, RAP1A, RAP1B, RIT1, DNM2	9.38E-09
GO:0015077~monovalent inorganic cation transmembrane transporter activity	1.06E-10	ATP5D, ATP5E, ATP6V0E1, UQCRC1, ATP5B, ATP6AP1, COX7B, COX7C, ATP5G2, ATP5G1, ATP6V1B2, COX7A2L, ATP6V1G1, UQCRFS1, COX5A, UQCRQ, COX5B, ATP5G3, ATP6V0B, ATP6V0C, UQCR11, COX6B1, ATP5L, ATP5O, ATP5I, ATP5H, ATP5J, TCIRG1, COX7A2, COX8A, ATP5F1, COX4I1, ATP6V1H, ATP1A1, ATP6V1F, COX6C, UQCRH, ATP6V1E1, ATP5C1, COX6A1,	1.94E-08

		ATP5A1, SURF1, UQCRB, SLC9A1	
GO:0019899~enzyme binding	2.05E-10	PDLIM5, U2AF2, SNRPD3, CBX3, PRKCSH, UBQLN1, CRADD, CTNNBL1, CTNNB1, CUL3, CDKN2D, RBCK1, PRKACA, SUPT5H, NCF2, POLG, RELA, STRN4, CST3, POLB, PPP1CB, PLAUR, LILRB1, SPAG9, NPC2, F3, CSTB, ATPIF1, CSTA, BIN1, CAMTA2, DIAPH1, NFKBIA, MYO9B, ITGB2, SNX3, ABCA1, FTH1, TAL1, PFN1, RAC1, SKIL, GABARAPL2, RILP, TAF7, AKAP8L, SMAD3, ATG3, PTPN11, CORO1A, HDAC3, HDAC1, RAP1A, HDAC9, GNB2L1, HDAC6, DENND5A, HINT1, PRKAG2, SHOC2, RPS3, MAPKAP1, CDK5RAP3, SERPINA1, PAK1, PLCB1, DOCK10, TOP2B, RHOH, FMNL1, IRAK1, STX4, ARHGEF2, UBE2A, SP100, LYN, PFKL, ZP3, PHKG2, UBE2I, PRKCE, PRKCD, UBE2B, HMGA1, FLNA, NLRP1, TAF10, ADRB2, CCND3, UBE2K, SBF2, BTG1, IPO5, MDM2, KPNA2, GRASP, RIN3, MAP3K11, GLRX3, CSF3, NXT1, CARHSP1, VIM, PML, ATP6V1G1, TCF7L2, STUB1, TRIB1, PTK2B, SQSTM1, HK3, NUDT21, PSTPIP1, PAFAH1B1, CCS, CD4, TPRKB, ACTB, UBXN1, PTPRC, PLEK, YWHAB, BAD, COTL1, SOD1, YWHAE, STAT3, ANXA2, DUSP2, LRP1, GSK3A, GSK3B, GGA2, RNF40, DNM2	3.34E-08
GO:0022890~inorganic cation transmembrane transporter activity	3.33E-10	ATP5D, ATP5E, ATP6V0E1, UQCRC1, ATP6AP1, ATP5B, SNCA, UQCRFS1, COX5A, COX5B, UQCRQ, ATP2B1, UQCR11, ATP5L, ATP5O, ATP5I, ATP5H, ATP5J, COX4I1, ATP6V1H, ATP6V1F, COX6C, RHCG, UQCRH, ATP2C1,	4.88E-08

		ATP5C1, SLC25A37, SURF1, UQCRB, COX7B, COX7C, ATP5G2, ATP6V1G1, COX7A2L, ATP6V1B2, ATP5G1, ATP5G3, ATP6V0B, ATP6V0C, SLC11A2, SLC11A1, COX6B1, CCS, SLC31A2, SLC31A1, TCIRG1, COX7A2, COX8A, ATP5F1, ATP1A1, ATP2A3, ATP6V1E1, COX6A1, ATP5A1, SLC9A1	
GO:0046961~proton-transporting ATPase activity, rotational mechanism	6.61E-09	ATP5D, ATP5E, ATP6V0E1, ATP5B, ATP6AP1, ATP5F1, ATP6V1H, ATP6V1B2, ATP5G1, ATP6V0B, ATP6V1F, ATP6V0C, ATP6V1E1, ATP5C1, ATP5O, ATP5A1, ATP5J	8.81E-07
GO:0019829~cation-transporting ATPase activity	2.52E-08	ATP5D, ATP5E, ATP6V0E1, ATP5B, ATP6AP1, ATP5F1, ATP6V1H, ATP6V1B2, ATP5G1, ATP6V0B, ATP6V1F, ATP6V0C, ATP2B1, ATP2C1, ATP2A3, ATP6V1E1, ATP5C1, ATP5O, ATP5A1, ASNA1, ATP5J	3.08E-06
GO:0008135~translation factor activity, nucleic acid binding	1.33E-07	EIF6, TUFM, EEF1B2, EIF5, ABTB1, EIF5A, EIF2A, EIF3D, EIF3B, EIF3G, EIF3H, EIF3E, EIF3F, EIF1AY, EIF3K, EIF3L, EIF1, EIF3I, EIF3M, EIF2B5, EEF1A1, EIF2S3, EEF2, EIF1B, ETF1, GTF2B, EIF4B, EIF4G2, EIF2AK1, EIF4E, EIF4H, EIF4A2, EIF4A1, TCEB2, EEF1G, EEF1D, EIF4E2	1.50E-05
GO:0046933~hydrogen ion transporting ATP synthase activity, rotational mechanism	1.50E-07	ATP5D, ATP5E, ATP6AP1, ATP5B, ATP5F1, ATP6V1B2, ATP5G1, ATP6V1F, ATP6V0C, ATP5C1, ATP5O, ATP5A1, ATP5J	1.57E-05
GO:0051082~unfolded protein binding	1.52E-07	HSP90AB1, SPG7, GRPEL1, DNAJB12, CCT2, CCT3, CALR, CANX, CDC37, AIP, DNAJB11, NPM1, DNAJA1, HSPE1, HSPA5, DNAJC4, HSPA8, DNAJC1, HSPA9, CLN3, TCP1, HSP90AA1, CCT6A, LRPAP1, CCT7, UXT, PFDN2, HSP90B1, CCT5, PFDN1, CCT4, PPIB, TBCA, PPIA, PFDN5, PFDN4, CCT8, AHSP, TOMM20, SIL1, DNAJB2	1.49E-05

GO:0003743~translation initiation factor activity	2.49E-07	EIF6, EIF5, EIF2A, EIF3D, EIF3B, EIF3G, EIF3H, EIF3E, EIF1AY, EIF3F, EIF3K, EIF3L, EIF1, EIF3I, EIF3M, EIF2B5, EIF2S3, EIF1B, GTF2B, EIF4B, EIF4G2, EIF4E, EIF2AK1, EIF4A2, EIF4H, EIF4A1, EIF4E2	2.28E-05
GO:0005198~structural molecule activity	5.97E-07	SEPT5, RPL18, MRPS36, RPL17, RPL19, MRPS33, RPL14, VAPA, RPL13, RPL15, RPLP2, RPS27L, RPL22L1, VCL, ACTG1, RPLP0, RPLP1, FAU, RPL10, RPL11, RPL12, ASPH, MRPL33, RPS27A, RPL36AL, RPL35A, MRPL52, MRPL51, DCTN3, CTNNA1, ISCU, ARPC1B, RPS19, MRPS18C, RPS16, MRPS18A, RPS14, RPS15, RPS12, RPS13, TUBA4A, MRPL47, MRPL48, CSTA, RPS11, UBA52, MYL6, MRPS15, MRPS11, ARPC5, MYL9, LAMB3, RPS27, RPS28, EIF3B, RPS29, ARPC3, RPL7, RPL6, ARPC2, RPL9, RPL8, RPL3, MRPL55, RPL5, RPS20, RPL10A, RPL7A, RPL4, RPS21, RPS23, RPS24, RPSA, MRPS24, RPS9, RPL23A, PMF1, EMILIN2, RPS6, RPS5, RPS8, RPS7, HNRNPH2, RPL18A, CDC42SE2, RPL37A, TLN1, CLTA, CLTB, RPS2, RPS3, RPS3A, COPB1, TUBB6, MSN, TUBA1A, TUBA1B, DEDD2, TUBA1C, MRPS6, RPS4X, NCK2, EPB41L3, RPL41, UBC, UBB, MATR3, COPE, VIM, RPL27A, RPL35, RPL36, RPS15A, RPL37, RPL38, TPM1, TPM4, MRPL20, CYLD, RPL30, RPL32, RPL31, MRPL14, RPL34, MRPL18, CD4, RSL24D1, THBS1, ACTB, RPL26, LMNA, ANXA1, RPL27, RPL24, RPL28, RPL29, MRPL23, MRPL21, MRPL28, ERBB2IP, RPL23, RPL22, RPL13A	5.15E-05
GO:0003729~mRNA binding	5.67E-06	PABPC4, RBM5, RPL35, CALR, RPS3, ZFP36L1, RPL7, HNRNPD, PABPC3, HNRNPC,	4.62E-04

		PABPC1, LUC7L3, HNRNPAB, ZFP36, FMR1, RNPS1, NXF1, MBD2, RPS5, EIF4A3, RPS14, EIF4A1, SLU7, RPS13, CELF1, CPSF1	
GO:0004298~threonine-type endopeptidase activity	5.70E-06	PSMB10, PSMA7, PSMA2, PSMA1, PSMB4, PSMB7, PSMB6, PSMB1, PSMA6, PSMA5, PSMA4, PSMA3, PSMB2	4.40E-04
GO:0070003~threonine-type peptidase activity	5.70E-06	PSMB10, PSMA7, PSMA2, PSMA1, PSMB4, PSMB7, PSMB6, PSMB1, PSMA6, PSMA5, PSMA4, PSMA3, PSMB2	4.40E-04
GO:0016564~transcription repressor activity	6.02E-06	JDP2, TSG101, EZH2, CBX3, NFKB1, HSBP1, YBX1, CITED2, EPC1, KDM1A, AES, PLRG1, CREG1, LRRFIP1, NFIL3, AKIRIN2, SP100, MTA2, RELB, ARID5A, MECP2, SF1, UBE2I, IFI16, RBBP7, MBD2, MXD1, MSC, JUNB, FOXN3, DDIT3, HES1, PFDN5, TGIF1, MDM2, LRCH4, CUX1, SBNO2, BCLAF1, HMGB2, PML, CALR, TCF7L2, CIR1, DRAP1, TSC22D4, BCL11A, BCL6, BHLHE40, SKIL, APEX1, ENO1, IKZF1, JARID2, TRIM28, TAF7, YWHAB, SMAD3, SAP18, SNW1, ILF3, KAT5, TRIM22, FOXP1, GPS2, HDAC5, ATXN1, ATF5, HDAC3, ATF3, ID2, PSMC3, PHB2, JAZF1, HIVEP1, HDAC9, KLF4, DNAJB6, HDAC6	4.42E-04
GO:0019904~protein domain specific binding	1.06E-05	HSP90AB1, MTSS1, TLN1, E2F4, RHOQ, CBX3, ARHGAP17, PTEN, WBP1, CRADD, DYNLL1, SRRM2, ILK, U2AF1, SYNJ2, IL1B, QKI, MICAL1, LRRFIP2, TUBA1A, HSP90AA1, SP100, NCF1, CCDC88C, INPPL1, C9ORF89, BAIAP2, STRN4, MECP2, UBE2I, MBD2, SLC9A3R1, SIRPA, NLRP1, VASP, ELMO1, LAT2, CHMP1A, CHMP1B, PSEN1, CNTROB, RIPK2,	7.42E-04

		ADAM19, GRASP, ADAM15, CHMP2A, YWHAZ, USP8, LITAF, GRB2, DAZAP2, STUB1, SRC, HNRNPM, SQSTM1, SHISA5, PYCARD, EXOC4, TAF9, ETV6, INPP5A, ERCC1, TFDP1, CARD8, HCLS1, DLGAP4, DENND1A, YWHAB, EVL, YWHAE, PTPN12, PTPN11, CYBA, SH3BGRL, PLSCR1, SON, VCP, BAX, YWHAQ, HGS, CALM3, CALM2, CALM1	
GO:0043022~ribosome binding	1.11E-05	EIF6, RPSA, SEC61B, WIBG, NAA10, EIF3K, EIF5A, SPCS1, EIF2A, HSPA5, ETF1, SEC61A1	7.42E-04
GO:0000166~nucleotide binding	2.10E-05	XRCC5, LDHB, HRAS, XRCC6, PGD, CLK1, KIFC3, ACTG1, DHX38, CLK3, ILK, MAP3K8, TLK2, YARS, GBP5, NCF1, PTBP1, UBE2J1, EIF2S3, UBE2J2, MYH9, GLUL, EIF2AK1, MAPK6, ARL8A, ARL8B, MYO18A, HSD17B10, GNAI2, PABPC4, UBA7, ZCRB1, ARF6, ARF5, NAGK, ATP6V0B, HADHA, HADHB, RAC2, RAC1, DDX60L, PABPC3, PABPC1, DHX58, RPS24, DHX9, MAP2K2, MAP2K3, TAOK3, SPHK1, GARS, NXF1, VDAC2, UBA1, ARF3, ARF4, GK, ZNF12, QARS, NUBP1, SRPR, RAP2C, LYN, PHKG2, NDUFA13, DECR1, DGUOK, CCT6A, GRHPR, PI4KB, NDUFA10, EIF4A3, EIF4A2, EIF4A1, GNAS, ARL4C, TUFM, MKNK2, HK2, HK1, ITM2B, DGKA, ACSL1, PTK2B, HK3, DHX15, RAB11A, CERK, ACSL3, ETFA, MARS, ACSL5, NADK, SDHA, MEF2D, RPS6KA1, CSNK1D, GSK3A, CSNK1E, SFPQ, GSK3B, ARAF, DPYD, DNM2, RALY, GNA15, ATP6AP1, RBM3, EIF5, RBM4, RBM5, RPS6KB2, RBM6, MTHFD1L, NLRC5, KDM1A, IDH3G, AAK1, DDX24, RALA, SAR1B, SAR1A, PABPN1,	1.34E-03

		PDXK, TWF2, STK24, HNRNPA2B1, RBKS, EEF2, HNRNPU, PNPLA8, SMARCA4, RAB7A, FGR, STK10, SNRPB2, MAPKAPK3, MAPKAPK2, ABCA1, HNRNPL, HNRNPM, DDX49, DDX3X, HNRNPF, HNRNPD, TAF9, HSPE1, HNRNPC, DDX41, RAB8B, MYO1G, MYO1F, IDH3B, RPL23A, RBMX, SAFB2, P2RX4, DDX56, HNRNPH3, HNRNPH2, PLK3, VCP, NDUFV1, PRKAR1A, HNRNPH1, IKBKB, ACOX1, RHOQ, FES, RHOU, CDC42, WARS, RHOA, RHOC, NDUFS2, RHOF, RHOG, RHOH, SGK1, SARS, PKN1, NCL, CDK5, GAK, ACADVL, CPSF6, TXNRD2, PGS1, UBE2R2, CHD2, HSPA4, HSPA5, EHD1, CHD4, HSPA8, HSPA9, EHD4, HCK, RAF1, DRG1, UBE2Q1, CYBB, HSP90B1, U2AF2, RAB1B, CCT2, CCT3, RAB1A, KIF13A, ATP2B1, NONO, DDX17, RAB28, NT5C2, U2AF1, RAB24, RAB20, GTPBP2, GTPBP1, GTPBP4, SF3B14, RAN, SUCLG1, PIM1, PIM3, PIM2, ATP2C1, CAMK1, TUBA4A, RAB13, RAB10, MDH1, FUS, COASY, PFKFB3, FARS2, CHKB, STK17B, OAS1, ADRBK1, MYO9B, CDC34, HNRNPA3, MOV10, SUPV3L1, HNRNPAB, TREX1, DDX5, HNRNPA1, U2AF1L4, HNRNPA0, CCT7, OASL, CCT5, CCT4, ATP2A3, CCT8, GRK6, RIT1, ATP5D, SPG7, ABCF3, RAB5C, ATP5B, CKB, ACTR3, TUBB6, CHST15, DOCK10, TOP2B, AGAP3, NT5C, RAB4B, MBD2, CDK11A, ADK, RBM39, SLC27A3, MAP3K11, RBM34, GCH1, STK40, SNRNP70, RAB2A, CSNK1A1, RBM22, RBM23, ATP1A1, MRPL23,	

		RAB32, PAPOLA, RAB31, ILF2, PYGL, FYN, RAB34, ATP5A1, PGK1, RBM17, SEPT5, GRPEL1, NRBP1, CASK, TARDBP, PRKACA, GNL2, MX1, MX2, CSNK1G2, EFTUD2, POR, MAP4K3, SLTM, MAP4K4, RIOK3, TIAL1, CLPP, SNRPA, RIPK2, CELF2, CELF1, SEPT6, GBP4, GBP2, SEPT9, GBP1, GPN2, MVD, HDGF, MAP4K1, KMO, SRC, KARS, DAZAP1, UBE2D3, EIF3B, UBE2D2, EIF3G, SKIL, UBE2D1, RUNX1, EWSR1, RUNX3, CSF1R, EEF1A1, AK2, RNPS1, EIF4B, TXNDC12, PSMC5, PSMC4, PSMC3, PSMC2, EIF4H, CPNE1, RAP1A, CIRBP, RAP1B, FPGS, ASNA1, GUK1, UBE2E2, PUF60, MYLK, UBE2E1, HSP90AB1, PRKAG2, MKI67IP, ACTR1A, PAK1, TUBA1A, TUBA1B, DUS1L, AKT3, TUBA1C, IRAK2, IRAK1, UBE2A, HSP90AA1, PFKL, NADSYN1, UBE2F, MINK1, UBE2I, UBE2H, PRKCE, NLRP3, UBE2B, PRKCD, NLRP1, UBE2N, TYK2, PRKD2, TARS, UBE2K, UBE2M, MATR3, UBE2Z, TRA2B, TRA2A, TRIB1, MSI2, GAPDH, ACTB, TCP1, NME1-NME2, PSPC1, TRPC4AP	
GO:0042802~identical protein binding	4.48E-05	S100A4, LDHB, S100A6, GRPEL1, NRBP1, SNCA, MTHFD1L, TGFB1, ACTG1, NONO, ATG7, FTL, DBNL, PDXK, PCBD1, RELA, FBP1, MYH9, GLUL, EIF2AK1, PSMA4, VEGFA, ATPIF1, SNRPC, EXT1, MYO18A, ITGA2B, SMARCA4, ALDOA, HSD17B10, OSTF1, MVD, STRAP, PFKFB3, NFKBIA, MYO9B, C14ORF166, PSMA7, CD74, SLC11A1, ECE1, RILPL2, BLOC1S2, RPL7, GSTK1, ITGB7, NPM1, PVRL2, CDA, HNRNPC, RUNX1, SDF4,	2.73E-03

		CSF1R, B4GALT1, SSSCA1, CARD8, LGALS1, S100A11, NR4A1, TREX1, SMAD3, TPD52L2, TAX1BP1, FOXP1, USF2, CCT7, P2RX4, DDX56, CORO1A, ATF3, HDAC1, ALDH2, POP4, IKBKB, KYNU, HEXB, NFKB1, STAC3, RABAC1, CASP8, GTF2A2, IRAK2, IRAK1, EXOSC8, HSP90AA1, SP100, PFKL, ADIPOR1, WAS, FLNA, TARS, ADRB2, SBF2, MDM2, TXNRD2, CTSC, COMMD1, GNAS, PRNP, LCP1, MAP3K11, TRA2A, PML, CALCOCO2, STUB1, APLP2, GCH1, EXOSC10, SH3GLB1, PYCARD, PCMT1, PAFAH1B1, CD4, THBS1, PTS, TRIP10, PTPRE, CEBPB, PLEK, SOD1, IRF9, ATXN1, NME1-NME2, PRLR, PYGL, JMJD6, FYN, BAX, BNIP3L, TRPC4AP, DPYD, SSNA1, RNF40, GCA, MGST1	
GO:0043021~ribonucleoprotein binding	5.39E-05	EIF6, RPSA, RBM3, EIF5A, EIF2A, ETF1, PPP1CA, SEC61B, SRPR, WIBG, NAA10, NPM1, EIF3K, SPCS1, HSPA5, SEC61A1	3.16E-03
GO:0046983~protein dimerization activity	5.56E-05	S100A6, GRPEL1, JDP2, NRBP1, VAPA, MTHFD1L, TGFB1, BATF, ATG7, SUPT5H, CAPNS1, PDXK, CD3D, SUCLG1, NCF4, MYH9, DDIT3, JUNB, EIF2AK1, VEGFA, SDCBP, ATPIF1, SNRPC, NFE2L2, BIN1, EXT1, MCL1, MVD, ADORA2A, MYO9B, PDSS1, ITGAM, PEF1, SLC11A1, ECE1, RPL7, NPM1, JUND, PVRL2, CDA, RUNX1, CSF1R, B4GALT1, CARD8, IKZF1, LGALS1, S100A11, TREX1, SMAD3, NR4A1, TPD52L2, FOXP1, USF2, P2RX4, ATF5, ATF4, CORO1A, ATF3, IKBKB, PDCD6, BACH1, KYNU, FOSL2, HEXA, HEXB, HPS1, NFKB1, MAX, FOS,	3.13E-03

		GTF2A2, NFIL3, TOP2B, FOSL1, IRAK2, PPP2R1A, IRAK1, HSP90AA1, SP100, POLR1D, ADIPOR1, FOSB, FLNA, TARS, ADRB2, SBF2, TXNRD2, MAP3K11, CREM, PML, CALCOCO2, STUB1, GCH1, XBP1, SH3GLB1, PPP2CA, PYCARD, PAFAH1B1, CD4, SUPT4H1, PTS, PTPRE, CEBPB, PLEK, CREB5, SOD1, STAT3, CYBA, CYBB, PRLR, PYGL, BAX, BNIP3L, TRPC4AP, DPYD, RNF40, MGST1, GCA	
GO:0019787~small conjugating protein ligase activity	1.11E-04	UBE2Z, TSG101, UBE2V1, ANAPC10, CDC34, ANAPC11, STUB1, RBX1, UBE2R2, PRPF19, ARIH1, UBE2D3, UBE2D2, RNF167, NEDD4L, UBE2D1, FBXO9, TRIP12, FBXO7, RNF144B, UBE2A, ANAPC5, UBR4, UBE2J1, BIRC6, UBE2L6, UBE2F, UBE2I, UBE2H, UBE2J2, TMEM189, BIRC3, ATG3, UBE2B, RBBP6, UBE2Q1, UBE2N, NOSIP, RNF7, UBE2K, UBE2M, MDM2, UBE2E2, RNF40, UBE2E1, FBXO11	6.04E-03
GO:0008092~cytoskeletal protein binding	1.47E-04	MTSS1, PDLIM5, CAPZA1, SNCA, VCL, MED28, TARDBP, BCL7B, DBNL, TWF2, BAIAP2, ACTN1, POLB, TMSB10, MYH9, CTNNA1, BCL2L11, ARPC1A, SPAG9, ARPC1B, UXT, TNNT1, CCR5, RHCG, SDCBP, FLII, BIN3, ARL8A, ARL8B, ALDOA, ADORA2A, DIAPH1, SSH2, MYO9B, ARPC4, ARPC5, FXYD5, CAPZB, PFN1, BLOC1S2, ARPC3, ARPC2, EMD, CDC42EP3, B4GALT1, GABARAPL2, PHACTR1, GABARAPL1, MYO1G, MYO1F, EVL, GAS7, MPRIP, CORO1C, CORO1A, WDR1, MYLK, HDAC6, TLN1, SDC4, SDC2, ACTR3, CXCR4, GSN, RHOA, VPS16, CAP1, MSN, FMNL1, PARVG, STX4, ARHGEF2,	7.67E-03

		CLMN, INPPL1, FLNA, VASP, NCK2, EPB41L3, ATG4D, CFL1, TMSB4X, MAPRE1, PRNP, PARVB, FHOD1, LCP1, FHL3, ABI1, TPM1, TPM4, TPM3, EZR, MEFV, PSTPIP1, PAFAH1B1, CNN2, COTL1, CAPN2, GABARAP, CAPN1, ANXA2, LSP1, NME1-NME2, GMFG, FYN, SVIL, CALM3, CALM2, CALM1, DNM2	
GO:0042625~ATPase activity, coupled to transmembrane movement of ions	4.34E-04	ATP5D, ATP5E, ATP6V0E1, ATP1B3, ATP5B, ATP6AP1, ATP5F1, ATP6V1H, ATP1A1, ATP6V1G1, ATP6V1B2, ATP5G1, ATP6V0B, ATP6V1F, ATP2B1, ATP6V0C, ATP2C1, ATP2A3, ATP6V1E1, ATP5C1, ATP5O, ATP5A1, ASNA1, ATP5J	2.17E-02
GO:0008320~protein transmembrane transporter activity	5.65E-04	SEC61B, TOMM7, MCL1, TIMM17A, SNCA, TIMM17B, TOMM20, TOMM22, SEC61A1, SEC61G	2.73E-02
GO:0043028~caspase regulator activity	6.16E-04	SIVA1, CARD8, SNCA, PRDX5, TNFSF14, RPS27L, BCL2L13, NLRP1, TNFRSF10B, TNFAIP8, PYCARD, HSPA5, CASP1	2.88E-02
GO:0005525~GTP binding	6.57E-04	SEPT5, HRAS, GNA15, RAB5C, EIF5, RAB1B, RHOQ, RHOU, RAB1A, CDC42, RAB28, SRPR, RHOA, TUBB6, RAB24, RALA, RHOC, DOCK10, MX1, SAR1B, GNL2, TUBA1A, MX2, RAB20, RHOF, TUBA1B, SAR1A, TUBA1C, RHOG, AGAP3, RHOH, GTPBP2, GTPBP1, GTPBP4, GBP5, RAP2C, RAN, NCF1, EFTUD2, SUCLG1, RAB4B, EIF2S3, EEF2, TUBA4A, GNAS, ARL8A, ARL8B, RAB13, GBP4, SEPT6, RAB10, ARL4C, GBP2, GBP1, SEPT9, TUFM, GPN2, RAB7A, GNAI2, ARF6, ARF5, GCH1, RAC2, RAC1, RAB11A, EHD1, EHD4, RAB2A, EEF1A1, RAB8B, DRG1, RAB32, RAB31, NME1-NME2, ARF3, RAB34, ARF4, RAP1A, RAP1B, RIT1,	2.97E-02

		DNM2	
GO:0016881~acid-amino acid ligase activity	7.75E-04	TSG101, TTLL4, UBE2A, ANAPC5, UBR4, UBE2J1, HERC4, UBE2F, UBE2I, UBE2H, UBE2J2, TMEM189, RBBP6, UBE2B, UBE2N, UBE2K, UBE2M, MDM2, FBXO11, UBE2Z, UBE2V1, ARPC4, ANAPC10, CDC34, ANAPC11, STUB1, RBX1, UBE2R2, PRPF19, ARIH1, UBE2D3, UBE2D2, NEDD4L, RNF167, UBE2D1, FBXO9, TRIP12, FBXO7, RNF144B, BIRC6, UBE2L6, BIRC3, UBE2Q1, ATG3, NOSIP, RNF7, FPGS, UBE2E2, RNF40, UBE2E1	3.39E-02
GO:0008134~transcription factor binding	1.02E-03	HMGN3, RBM4, PDLIM1, RAB1A, CITED2, AIP, CTNNB1, KDM1A, SMARCD3, SND1, PQBP1, RARA, RAN, PCBD1, RELA, RELB, PIM1, MECP2, JUNB, DDIT3, AHR, MTF1, TGIF1, EDF1, SMARCA4, CAMTA2, NFKBIB, NFKBIA, NR1H2, DRAP1, NPM1, TAF9, SKIL, RUNX1, TFDP1, MAFB, SUB1, TAF7, SRA1, SAP18, SMAD3, PMF1, DDX5, KAT5, USF2, HDAC5, ATF5, OASL, HDAC3, ATF3, PSMC5, HDAC1, PSMC3, JAZF1, HDAC9, SLC9A1, ENY2, NBN, E2F4, TSG101, NFKB2, HSBP1, RFXANK, RPS3, MAX, AES, PLRG1, GPX3, GTF2A2, CREG1, NRG1, NFIL3, SERTAD2, PSMD9, SP100, SF1, FOSB, MXD1, HMGA1, MSC, FLNA, TAF10, MED4, BTG1, PFDN5, YWHAZ, NMI, CREM, PML, TCF7L2, TRIB1, CIR1, BCL11A, BCL3, APEX1, TRIP12, CHD4, ENO1, TRIM28, YWHAB, DRG1, TRIM22, STAT3, GPS2, GSK3B	4.33E-02
GO:0015405~P-P-bond-hydrolysis-driven transmembrane transporter activity	1.10E-03	ATP5D, ATP5E, ATP6V0E1, ATP1B3, ATP5B, TIMM17A, ATP6AP1, SNCA, TIMM17B, ATP6V1B2, ATP6V1G1,	4.51E-02

		ATP5G1, ATP6V0B, ATP2B1, ATP6V0C, TOMM7, ATP5O, SEC61A1, ATP5J, ATP5F1, ATP6V1H, ATP1A1, ATP6V1F, SEC61B, ATP2C1, ATP2A3, ATP6V1E1, ATP5C1, TOMM20, TRPC4AP, ATP5A1, ASNA1, SEC61G	
GO:0015399~primary active transmembrane transporter activity	1.10E-03	ATP5D, ATP5E, ATP6V0E1, ATP1B3, ATP5B, TIMM17A, ATP6AP1, SNCA, TIMM17B, ATP6V1B2, ATP6V1G1, ATP5G1, ATP6V0B, ATP2B1, ATP6V0C, TOMM7, ATP5O, SEC61A1, ATP5J, ATP5F1, ATP6V1H, ATP1A1, ATP6V1F, SEC61B, ATP2C1, ATP2A3, ATP6V1E1, ATP5C1, TOMM20, TRPC4AP, ATP5A1, ASNA1, SEC61G	4.51E-02
GO:0051087~chaperone binding	1.11E-03	GRPEL1, TBCA, TBCD, PFDN4, HSPE1, SOD1, CALR, AHSA2, AHSA1, DNAJB6, DNAJC1	4.42E-02
GO:0003727~single-stranded RNA binding	1.21E-03	ZFP36, ATXN1, EIF4A3, CNBP, PTBP1, PABPC4, MSI2, PABPC3, PABPC1, SNRPC, HNRNPH1, HNRNPA1	4.69E-02
GO:0001846~opsonin binding	1.30E-03	C1QBP, CD93, CLEC7A, CALR, ITGAM, CD14	4.91E-02
GO:0022884~macromolecule transmembrane transporter activity	1.38E-03	SEC61B, TOMM7, TIMM17A, SNCA, TIMM17B, TOMM20, SEC61A1, SEC61G	5.05E-02
GO:0015450~P-P-bond-hydrolysis-driven protein transmembrane transporter activity	1.38E-03	SEC61B, TOMM7, TIMM17A, SNCA, TIMM17B, TOMM20, SEC61A1, SEC61G	5.05E-02
GO:0019001~guanyl nucleotide binding	1.47E-03	SEPT5, HRAS, GNA15, RAB5C, EIF5, RAB1B, RHOQ, RHOU, RAB1A, CDC42, RAB28, SRPR, RHOA, TUBB6, RAB24, RALA, RHOC, DOCK10, MX1, SAR1B, GNL2, TUBA1A, MX2, RAB20, RHOF, TUBA1B, SAR1A, TUBA1C, RHOG, AGAP3, RHOH, GTPBP2, GTPBP1, GTPBP4, GBP5, RAP2C, RAN, NCF1, EFTUD2, SUCLG1, RAB4B, EIF2S3, EEF2, TUBA4A, GNAS, ARL8A, ARL8B, RAB13, GBP4, SEPT6,	5.25E-02

		RAB10, ARL4C, GBP2, GBP1, SEPT9, TUFM, GPN2, RAB7A, GNAI2, ARF6, ARF5, GCH1, RAC2, RAC1, RAB11A, EHD1, EHD4, RAB2A, EEF1A1, RAB8B, DRG1, RAB32, RAB31, NME1-NME2, ARF3, RAB34, ARF4, RAP1A, RAP1B, RIT1, DNM2	
GO:0032561~guanyl ribonucleotide binding	1.47E-03	SEPT5, HRAS, GNA15, RAB5C, EIF5, RAB1B, RHOQ, RHOU, RAB1A, CDC42, RAB28, SRPR, RHOA, TUBB6, RAB24, RALA, RHOC, DOCK10, MX1, SAR1B, GNL2, TUBA1A, MX2, RAB20, RHOF, TUBA1B, SAR1A, TUBA1C, RHOG, AGAP3, RHOH, GTPBP2, GTPBP1, GTPBP4, GBP5, RAP2C, RAN, NCF1, EFTUD2, SUCLG1, RAB4B, EIF2S3, EEF2, TUBA4A, GNAS, ARL8A, ARL8B, RAB13, GBP4, SEPT6, RAB10, ARL4C, GBP2, GBP1, SEPT9, TUFM, GPN2, RAB7A, GNAI2, ARF6, ARF5, GCH1, RAC2, RAC1, RAB11A, EHD1, EHD4, RAB2A, EEF1A1, RAB8B, DRG1, RAB32, RAB31, NME1-NME2, ARF3, RAB34, ARF4, RAP1A, RAP1B, RIT1, DNM2	5.25E-02
GO:0016676~oxidoreductase activity, acting on heme group of donors, oxygen as acceptor	1.72E-03	COX7A2, COX7B, COX8A, COX6B1, COX7C, COX4I1, COX6A1, COX7A2L, COX5A, SURF1, COX5B, COX6C	5.98E-02
GO:0015002~heme-copper terminal oxidase activity	1.72E-03	COX7A2, COX7B, COX8A, COX6B1, COX7C, COX4I1, COX6A1, COX7A2L, COX5A, SURF1, COX5B, COX6C	5.98E-02
GO:0004129~cytochrome-c oxidase activity	1.72E-03	COX7A2, COX7B, COX8A, COX6B1, COX7C, COX4I1, COX6A1, COX7A2L, COX5A, SURF1, COX5B, COX6C	5.98E-02
GO:0016675~oxidoreductase activity, acting on heme group of donors	1.72E-03	COX7A2, COX7B, COX8A, COX6B1, COX7C, COX4I1, COX6A1, COX7A2L, COX5A, SURF1, COX5B, COX6C	5.98E-02
GO:0004842~ubiquitin-protein ligase activity	2.10E-03	UBE2Z, ANAPC10, CDC34, ANAPC11, STUB1, UBE2R2,	7.09E-02

		PRPF19, UBE2D3, UBE2D2, RNF167, NEDD4L, UBE2D1, FBXO9, TRIP12, FBXO7, RNF144B, UBE2A, ANAPC5, UBR4, UBE2J1, BIRC6, UBE2L6, UBE2H, UBE2J2, BIRC3, UBE2B, RBBP6, UBE2Q1, UBE2N, NOSIP, UBE2K, UBE2M, MDM2, UBE2E2, RNF40, UBE2E1, FBXO11	
GO:0003779~actin binding	2.28E-03	MTSS1, TLN1, PDLIM5, CAPZA1, VCL, ACTR3, MED28, GSN, CXCR4, VPS16, CAP1, MSN, BCL7B, PARVG, FMNL1, DBNL, TWF2, CLMN, INPPL1, ACTN1, TMSB10, MYH9, VASP, FLNA, ARPC1A, EPB41L3, ARPC1B, UXT, CCR5, CFL1, FLII, TMSB4X, FHOD1, LCP1, PARVB, ALDOA, DIAPH1, SSH2, FHL3, ARPC4, MYO9B, ARPC5, FXYD5, TPM1, CAPZB, TPM4, TPM3, PFN1, EZR, ARPC3, MEFV, ARPC2, PSTPIP1, CNN2, EMD, PHACTR1, MYO1G, MYO1F, EVL, COTL1, GAS7, MPRIP, CORO1C, LSP1, CORO1A, GMFG, SVIL, WDR1, MYLK, HDAC6	7.50E-02
GO:0019843~rRNA binding	2.40E-03	RPL9, RPL8, NPM1, RPS9, RPL37, MRPS6, RPL5, RPL11, RPL23A, RPS11, RPS4X, MRPL20	7.69E-02
GO:0042803~protein homodimerization activity	2.76E-03	GRPEL1, S100A6, KYNU, NRBP1, HEXB, NFKB1, TGFB1, MTHFD1L, ATG7, GTF2A2, IRAK2, IRAK1, HSP90AA1, PDXK, SP100, MYH9, FLNA, TARS, ADRB2, EIF2AK1, SBF2, VEGFA, TXNRD2, ATPIF1, SNRPC, EXT1, MAP3K11, MVD, PML, CALCOCO2, MYO9B, STUB1, GCH1, SLC11A1, ECE1, RPL7, SH3GLB1, PVRL2, NPM1, PYCARD, CDA, PAFAH1B1, CD4, PTS, RUNX1, CSF1R, B4GALT1, CARD8, CEBPB, PTPRE, PLEK, LGALS1, S100A11, SMAD3, NR4A1,	8.62E-02

		TREX1, TPD52L2, SOD1, FOXP1, USF2, P2RX4, CORO1A, PRLR, PYGL, BAX, BNIP3L, TRPC4AP, DPYD, RNF40, MGST1, GCA	
GO:0008121~ubiquinol-cytochrome-c reductase activity	3.04E-03	UQCRC1, UQCR11, UQCRH, UQCRFS1, UQCRQ, UQCRB	9.26E-02
GO:0016681~oxidoreductase activity, acting on diphenols and related substances as donors, cytochrome as acceptor	3.04E-03	UQCRC1, UQCR11, UQCRH, UQCRFS1, UQCRQ, UQCRB	9.26E-02
GO:0051920~peroxiredoxin activity	3.04E-03	PRDX6, SEP15, PRDX5, PRDX2, PRDX1, PARK7	9.26E-02
GO:0016879~ligase activity, forming carbon-nitrogen bonds	3.32E-03	TSG101, TTLL4, MTHFD1L, UBE2A, ANAPC5, NADSYN1, UBR4, UBE2J1, HERC4, UBE2F, UBE2I, UBE2H, UBE2J2, TMEM189, RBBP6, UBE2B, UBE2N, GLUL, UBE2K, UBE2M, MDM2, FBXO11, UBE2Z, UBE2V1, ARPC4, ANAPC10, CDC34, ANAPC11, STUB1, RBX1, UBE2R2, PRPF19, ARIH1, UBE2D3, UBE2D2, NEDD4L, RNF167, UBE2D1, FBXO9, TRIP12, FBXO7, RNF144B, BIRC6, UBE2L6, BIRC3, UBE2Q1, ATG3, NOSIP, RNF7, FPGS, UBE2E2, RNF40, UBE2E1	9.86E-02
GO:0019900~kinase binding	4.04E-03	GLRX3, PDLIM5, HINT1, VIM, PRKAG2, ITGB2, UBQLN1, PRKCSH, TCF7L2, FTH1, TRIB1, CTNNB1, RPS3, PTK2B, SQSTM1, CDKN2D, RBCK1, CD4, CDK5RAP3, PRKACA, TOP2B, TPRKB, ACTB, PTPRC, IRAK1, SP100, PLEK, PFKL, RELA, SMAD3, BAD, PPP1CB, FLNA, STAT3, SPAG9, CORO1A, DUSP2, CCND3, GSK3A, BTG1, GSK3B, MAP3K11	1.17E-01
GO:0032403~protein complex binding	4.42E-03	UQCRC2, ATP5D, YWHAZ, UQCRC1, TXN2, NFKBIA, FCGRT, FKBP1A, UQCRFS1, CALR, SLC11A2, TNFRSF1A, LAMB3, DOCK2, PTK2B,	1.24E-01

		TGFBI, ILK, FCER1G, ATP5O, PRKACA, THBS1, FCER1A, ICAM1, LYN, LGALS3, ICAM2, RELA, ICAM3, STRN4, YWHAB, TREX1, ACTN1, C12ORF44, PTPN11, CDKN1A, ADRB2, DOK3, FCAR, ERBB2IP, CLIC4, UQCRH, ITGA5, PTPN1, RNF40, HDAC6	
GO:0008565~protein transporter activity	4.66E-03	ARFGAP3, AP1G2, XPO6, MCL1, TIMM17A, AP2S1, TIMM17B, SNCA, RUFY1, SNX2, SEC62, VTI1A, COG3, AP1S2, SEC61B, TOMM7, AP2A1, TOMM5, IPO5, TOMM20, TOMM22, KPNA2, SEC61A1, SEC61G	1.28E-01
GO:0016566~specific transcriptional repressor activity	5.31E-03	IKZF1, TAF7, SMAD3, NFKB1, UBE2I, TCF7L2, FOXP1, HDAC5, PHB2, TGIF1, HDAC9, AKIRIN2, HDAC6	1.42E-01
GO:0003714~transcription corepressor activity	5.71E-03	TSG101, HSBP1, CITED2, CIR1, AES, PLRG1, DRAP1, BCL11A, CREG1, SKIL, NFIL3, APEX1, ENO1, SP100, RELB, TRIM28, YWHAB, SF1, MECP2, SAP18, MXD1, MSC, TRIM22, JUNB, DDIT3, GPS2, HDAC5, ATF5, HDAC3, ATF3, PSMC3, PFDN5, JAZF1, TGIF1, HDAC9	1.49E-01
GO:0009055~electron carrier activity	5.95E-03	CYB5R3, ACOX1, CYB5R2, PYROXD1, CYC1, IL4I1, COX5A, UQCRFS1, NDUFAF2, HMOX2, KDM1A, NDUFS6, UQCR11, NARF, SMOX, ASPH, NDUFS3, NDUFS2, NQO2, NCF2, NCF1, CYCS, POR, NDUFA12, ACADVL, TXNRD2, DEGS1, GLRX3, TXN2, KMO, COX7A2L, DOCK2, AKR1A1, ETFB, GLRX, ETFA, TBXAS1, COX7A2, IDH3B, SDHA, TXNDC12, SDHB, CYBA, CYBB, TXNDC17, SDHC, ALDH2, DPYD, SH3BGRL3	1.52E-01
GO:0060590~ATPase regulator activity	5.98E-03	GRPEL1, ATPIF1, AHSA2, AHSA1, DNAJB6, DNAJC1	1.51E-01
GO:0016679~oxidoreductase activity, acting on diphenols and related substances as	5.98E-03	UQCRC1, UQCR11, UQCRH, UQCRFS1, UQCRQ, UQCRB	1.51E-01

donors			
GO:0048487~beta-tubulin binding	6.03E-03	B4GALT1, UXT, GABARAPL2, GABARAPL1, ARL8A, ARL8B, EMD, GABARAP	1.49E-01
GO:0008656~caspase activator activity	6.03E-03	SIVA1, CARD8, TNFRSF10B, PYCARD, RPS27L, BCL2L13, CASP1, NLRP1	1.49E-01
GO:0004869~cysteine-type endopeptidase inhibitor activity	6.80E-03	CAST, SNCA, CST3, PRDX5, TNFSF14, BIRC6, CARD16, CST7, TNFAIP8, CARD17, CSTB, CSTA, HSPA5	1.64E-01
GO:0019901~protein kinase binding	7.12E-03	GLRX3, PDLIM5, HINT1, PRKAG2, VIM, ITGB2, PRKCSH, TCF7L2, RPS3, TRIB1, PTK2B, SQSTM1, CDKN2D, RBCK1, CD4, CDK5RAP3, PRKACA, TOP2B, TPRKB, ACTB, PTPRC, IRAK1, PLEK, RELA, SMAD3, BAD, PPP1CB, FLNA, STAT3, SPAG9, DUSP2, CCND3, GSK3A, GSK3B, MAP3K11	1.68E-01
GO:0003712~transcription cofactor activity	9.63E-03	ENY2, TSG101, RBM4, PDLIM1, NFKB2, HSBP1, RFXANK, CTNNB1, CITED2, AIP, MAX, AES, PLRG1, SMARCD3, SND1, GTF2A2, CREG1, PQBP1, RARA, NFIL3, NRG1, SERTAD2, PSMD9, SP100, PCBD1, RAN, RELB, MECP2, SF1, MXD1, MSC, HMGA1, JUNB, DDIT3, TAF10, MED4, MTF1, BTG1, PFDN5, TGIF1, EDF1, NMI, CREM, NFKBIB, PML, CIR1, DRAP1, NPM1, BCL11A, TAF9, SKIL, APEX1, TFDP1, ENO1, SUB1, TRIM28, SRA1, TAF7, YWHAB, SAP18, PMF1, DDX5, KAT5, TRIM22, GPS2, HDAC5, ATF5, HDAC3, ATF3, PSMC5, PSMC3, JAZF1, HDAC9	2.17E-01
GO:0004197~cysteine-type endopeptidase activity	9.78E-03	USP7, CFLAR, CTSZ, USP8, CAPNS1, PDIA3, CAPN2, CRADD, CTSW, CAPN1, ATG4D, CASP4, CARD16, CASP8, CARD17, PYCARD, CTSC, CASP1, USP15, CTSH	2.17E-01
GO:0005080~protein kinase C binding	1.23E-02	GLRX3, PLEK, SQSTM1, HINT1, PDLIM5, RBCK1,	2.62E-01

		PRKCSH, TOP2B, FLNA	
GO:0019788~NEDD8 ligase activity	1.25E-02	RNF7, UBE2M, UBE2F, RBX1	2.61E-01
GO:0047485~protein N-terminus binding	1.32E-02	MORF4L1, ACOX1, STX5, NBN, HAX1, PDLIM5, RELA, MECP2, EIF5A, TGFB1, SRRM2, EIF3E, ALOX5AP, YWHAQ, EXOC4, CALM3, SDCBP, RPS21, CALM2, CALM1, SMARCA4, GLRX	2.70E-01
GO:0042826~histone deacetylase binding	1.36E-02	TAL1, CAMTA2, HDAC3, HDAC1, NUDT21, YWHAB, HDAC9, TOP2B, KPNA2, YWHAE, HDAC6	2.72E-01
GO:0004721~phosphoprotein phosphatase activity	1.46E-02	PPP6C, STYXL1, SSH2, DUSP10, PPM1A, PTEN, MTMR3, PPP2CA, CTDSP1, PPP3CC, PPP4C, PTPN6, PTPRC, PTPRE, PTPN18, PTPN2, CYCS, DUSP22, PPP1CB, PTPN12, PTPN11, DUSP5, PPM1F, PPM1G, DUSP3, PPP1CA, MTMR14, DUSP2, SBF1, DUSP1, PTP4A1, PTP4A2, PTPN1, PPP1R15B, PHPT1, DUSP6, SSU72	2.86E-01
GO:0004601~peroxidase activity	1.71E-02	MGST3, PTGS2, TXNDC17, PRDX6, GSTK1, GPX4, GPX3, SEP15, PRDX5, PRDX2, PRDX1	3.23E-01
GO:0048306~calcium-dependent protein binding	1.71E-02	RBM22, S100A4, ANXA7, CLN3, S100A6, S100P, TSG101, ANXA11, S100A11, PDCD6IP, PDCD6	3.23E-01
GO:0016684~oxidoreductase activity, acting on peroxide as acceptor	1.71E-02	MGST3, PTGS2, TXNDC17, PRDX6, GSTK1, GPX4, GPX3, SEP15, PRDX5, PRDX2, PRDX1	3.23E-01
GO:0002020~protease binding	1.75E-02	LRP1, POLG, F3, CST3, CSTB, SERPINA1, CSTA, CRADD	3.25E-01
GO:0008022~protein C-terminus binding	2.00E-02	XRCC5, USP7, HRAS, SNX17, VIM, XRCC6, PPM1A, SRC, CTNNB1, NIPBL, DYNLL1, BLOC1S2, PPP2CA, PEX16, PABPC1, TOP2B, TINF2, ERCC1, NCF2, BAIAP2, YWHAB, OPTN, SLC9A3R1, PRKCD, NCL, PCGF1, FOXN3, ATXN1, CORO1A, ATF4, MAPRE1, BIN1	3.58E-01
GO:0016209~antioxidant activity	2.03E-02	PTGS2, PRDX5, PRDX2, SOD1, PRDX1, SOD2, MGST3,	3.58E-01

		TXNDC17, PRDX6, GPX4, GSTK1, GPX3, SEP15, TXNRD2	
GO:0003746~translation elongation factor activity	2.15E-02	TUFM, EEF1A1, EEF1B2, ABTB1, TCEB2, EEF1G, EIF5A, EEF2, EEF1D	3.70E-01
GO:0008187~poly-pyrimidine tract binding	2.24E-02	ATXN1, PTBP1, PABPC4, MSI2, HNRNPH1	3.78E-01
GO:0019200~carbohydrate kinase activity	2.35E-02	PFKL, PFKFB3, FGGY, HK3, HK2, HK1, RBKS, NAGK	3.89E-01
GO:0019992~diacylglycerol binding	2.37E-02	ARHGEF2, RAF1, AKAP13, MYO9B, PRKCE, VAV1, STAC3, PRKCD, DGKA, HMHA1, PRKD2, DGAT1, RASGRP4, ARAF, DEF8, RASGRP2, CHN2, RAPGEF2	3.87E-01
GO:0008289~lipid binding	2.45E-02	TSPO, PTGS2, PITPNA, SNCA, NDUFAB1, BUD31, PTEN, STAC3, STARD3, HMHA1, PICALM, RARA, ATP5O, RAPGEF2, PLCB2, CLINT1, PLD1, ARHGEF2, STX3, LYN, INPPL1, NCF1, NCF4, PRKCE, PRKCD, MCTP1, PRKD2, NPC2, CD36, DGAT1, SBF2, F3, PRAM1, DEF8, SNX10, HSD17B10, HDLBP, RBP7, SNX17, FFAR2, SNX2, AKAP13, MYO9B, ATP5G2, SNX4, ATP5G1, ABCA1, SNX3, NR3C1, ATP5G3, HADHA, HADHB, DGKA, ANXA6, ANXA7, RASGRP4, SH3GLB1, ALOX5AP, RASGRP2, PEMT, SNX24, TRIP10, AP2M1, WDFY1, SNX29, PLEK, SELL, PSAP, NSFL1C, ANXA1, RUFY1, RAF1, ANXA5, DBI, VAV1, ANXA2, APOL3, VCP, PYGL, BAX, ANXA11, ARAF, CPNE1, CHN2, ARAP1	3.93E-01
GO:0016408~C-acyltransferase activity	2.51E-02	SPTLC2, SLC27A3, ACAT2, ACSL3, HADHA, ACAA1, HADHB	3.96E-01
GO:0001671~ATPase activator activity	2.77E-02	AHSA2, AHSA1, DNAJB6, DNAJC1	4.23E-01
GO:0042379~chemokine receptor binding	2.83E-02	CXCL1, YARS, CCL2, IL8, CXCL3, CXCL2, PF4, PF4V1, CCL7, CXCL10, CCL20, PPBP, CXCL16, CKLF	4.26E-01
GO:0004177~aminopeptidase	3.01E-02	LAP3, NPEPL1, PEPD, TPP2,	4.41E-01

activity		RNPEPL1, ANPEP, DPP7, RNPEP, XPNPEP1, DNPEP	
GO:0016791~phosphatase activity	3.26E-02	IMPA2, PTEN, NT5C2, SYNJ2, CTDSP1, PPP4C, NT5C, INPPL1, CYCS, FBP1, PPP1CB, PPM1F, MTMR11, PPM1G, PPP1CA, MTMR14, SBF1, SBF2, PPP1R15B, SSU72, VPS29, STYXL1, PPP6C, PFKFB3, SSH2, DUSP10, PPM1A, MTMR3, INPP5K, PPP2CA, PPP3CC, INPP5A, PTPRC, PTPN6, PTPRE, PTPN18, PTPN2, DUSP22, ATP1A1, PTPN12, PTPN11, DUSP5, DUSP3, DUSP2, DUSP1, PTP4A1, PTP4A2, PTPN1, PHPT1, DUSP6	4.64E-01
GO:0003697~single-stranded DNA binding	3.29E-02	SSBP4, HMGB2, SSBP3, CNBP, SSBP2, SSBP1, SUB1, RAD23A, HNRNPA2B1, TREX1, HNRNPA1, YBX1, FUBP1, PCBP1, ERCC1	4.63E-01
GO:0008234~cysteine-type peptidase activity	3.31E-02	USP7, USP8, PDIA3, USP3, BAP1, SENP5, CRADD, CYLD, CASP4, CASP8, PYCARD, USP36, CASP1, USP15, CFLAR, CTSZ, CAPNS1, UFD1L, CAPN2, CTSW, CAPN1, SENP3, ATG4D, CARD16, OTUB1, ATG4B, CARD17, CTSC, UCHL3, TNFAIP3, CTSH	4.61E-01
GO:0070717~poly-purine tract binding	3.56E-02	ATXN1, EIF4A3, PABPC4, PABPC3, PABPC1	4.81E-01
GO:0042626~ATPase activity, coupled to transmembrane movement of substances	3.67E-02	ATP5D, ATP5E, ATP6V0E1, ATP1B3, ATP6AP1, ATP5B, ATP6V1G1, ATP6V1B2, ATP5G1, ATP6V0B, ATP6V0C, ATP2B1, ATP5O, ATP5J, ATP5F1, ATP6V1H, ATP1A1, ATP6V1F, ATP2C1, ATP2A3, ATP6V1E1, ATP5C1, TRPC4AP, ATP5A1, ASNA1	4.88E-01
GO:0032813~tumor necrosis factor receptor superfamily binding	3.68E-02	BID, SIVA1, TNFSF10, MYD88, TNFSF13B, MADD, BRE, TNFSF14, TNFSF13, TNFSF8	4.85E-01
GO:0016887~ATPase activity	3.87E-02	XRCC5, ATP5D, ATP5E, ATP6V0E1, ABCF3, ATP1B3, ATP6AP1, ATP5B, XRCC6, ATP2B1, DDX17, DHX38, DDX24, ATP5L, ATP5O,	4.98E-01

		PSMD6, ATP5I, ATP5H, ATP5J, CCNH, ATP6V1H, MYH9, ATP6V1F, EIF4A3, ATP2C1, EIF4A2, EIF4A1, ATP5C1, MYO18A, SMARCA4, MYL6, MYO9B, ATP5G1, ATP6V1G1, ABCA1, ATP6V1B2, ATP6V0B, ATP6V0C, DDX3X, DDX49, DDX60L, CHD2, DHX15, DDX41, CHD4, DHX58, HSPA8, DHX9, ATP5J2, ATP5F1, ATP1A1, DDX5, DDX56, CRBN, PSMC5, PSMC4, VCP, ATP2A3, PSMC2, ATP6V1E1, CCT8, TRPC4AP, ATP5A1, ASNA1	
GO:0008009~chemokine activity	3.88E-02	CXCL1, CCL2, IL8, CXCL3, CXCL2, PF4, PF4V1, CCL7, CXCL10, CCL20, PPBP, CXCL16, CKLF	4.95E-01
GO:0043492~ATPase activity, coupled to movement of substances	4.04E-02	ATP5D, ATP5E, ATP6V0E1, ATP1B3, ATP6AP1, ATP5B, ATP6V1G1, ATP6V1B2, ATP5G1, ATP6V0B, ATP6V0C, ATP2B1, ATP5O, ATP5J, ATP5F1, ATP6V1H, ATP1A1, ATP6V1F, ATP2C1, ATP2A3, ATP6V1E1, ATP5C1, TRPC4AP, ATP5A1, ASNA1	5.06E-01
GO:0017076~purine nucleotide binding	4.16E-02	XRCC5, HRAS, XRCC6, RAB1B, CCT2, CCT3, CLK1, RAB1A, KIFC3, KIF13A, ACTG1, ATP2B1, DDX17, DHX38, CLK3, RAB28, ILK, MAP3K8, RAB24, TLK2, RAB20, GTPBP2, GTPBP1, YARS, GTPBP4, GBP5, RAN, NCF1, SUCLG1, PIM1, UBE2J1, EIF2S3, PIM3, UBE2J2, PIM2, MYH9, GLUL, EIF2AK1, MAPK6, ATP2C1, CAMK1, TUBA4A, ARL8A, RAB13, ARL8B, RAB10, MYO18A, COASY, GNAI2, PFKFB3, CHKB, FARS2, UBA7, STK17B, OAS1, MYO9B, ADRBK1, ARF6, CDC34, ARF5, NAGK, ATP6V0B, MOV10, RAC2, RAC1, DDX60L, SUPV3L1, DHX58, DHX9, MAP2K2, MAP2K3, TAOK3, SPHK1, GARS, TREX1, DDX5, CCT7,	5.12E-01

		OASL, CCT5, CCT4, ATP2A3, UBA1, ARF3, ARF4, CCT8, GRK6, RIT1, GK, ATP5D, SPG7, ABCF3, RAB5C, ATP5B, ZNF12, QARS, CKB, ACTR3, NUBP1, SRPR, TUBB6, CHST15, DOCK10, TOP2B, AGAP3, RAP2C, LYN, PHKG2, RAB4B, NDUFA13, DGUOK, CCT6A, PI4KB, NDUFA10, EIF4A3, CDK11A, ADK, EIF4A2, EIF4A1, GNAS, ARL4C, MAP3K11, TUFM, MKNK2, HK2, HK1, ITM2B, GCH1, DGKA, ACSL1, STK40, PTK2B, HK3, DHX15, RAB11A, CERK, ACSL3, ETFA, MARS, ACSL5, RAB2A, CSNK1A1, NADK, ATP1A1, SDHA, RAB32, RAB31, PAPOLA, CSNK1D, RPS6KA1, ILF2, PYGL, GSK3A, FYN, CSNK1E, GSK3B, ARAF, RAB34, ATP5A1, DPYD, PGK1, DNM2, SEPT5, GNA15, GRPEL1, NRBP1, ATP6AP1, EIF5, RPS6KB2, CASK, MTHFD1L, NLRC5, KDM1A, IDH3G, AAK1, DDX24, RALA, PRKACA, GNL2, MX1, SAR1B, MX2, SAR1A, PDXK, TWF2, CSNK1G2, STK24, EFTUD2, EEF2, RBKS, HNRNPU, MAP4K3, PNPLA8, MAP4K4, RIOK3, CLPP, RIPK2, SEPT6, GBP4, GBP2, SEPT9, GBP1, SMARCA4, GPN2, RAB7A, FGR, MVD, STK10, MAPKAPK3, MAP4K1, KMO, ABCA1, MAPKAPK2, KARS, SRC, UBE2D3, UBE2D2, DDX49, DDX3X, TAF9, HSPE1, UBE2D1, RUNX1, DDX41, RUNX3, CSF1R, EEF1A1, RAB8B, MYO1G, AK2, MYO1F, TXNDC12, P2RX4, DDX56, PLK3, PSMC5, VCP, PSMC4, PSMC3, PSMC2, PRKAR1A, RAP1A, RAP1B, IKBKB, ASNA1, FPGS, GUK1, MYLK, UBE2E2, UBE2E1, HSP90AB1,	

		ACOX1, PRKAG2, RHOQ, FES, RHOU, CDC42, WARS, ACTR1A, RHOA, RHOC, PAK1, TUBA1A, DUS1L, TUBA1B, RHOF, AKT3, TUBA1C, RHOG, RHOH, IRAK2, IRAK1, SGK1, UBE2A, HSP90AA1, PFKL, SARS, NADSYN1, MINK1, UBE2F, PKN1, UBE2I, UBE2H, PRKCE, NLRP3, UBE2B, PRKCD, CDK5, NLRP1, GAK, UBE2N, TYK2, ACADVL, PRKD2, TARS, UBE2K, UBE2M, TXNRD2, PGS1, UBE2Z, TRIB1, UBE2R2, CHD2, HSPA4, HSPA5, EHD1, CHD4, HSPA8, EHD4, HSPA9, ACTB, TCP1, HCK, RAF1, DRG1, UBE2Q1, CYBB, HSP90B1, NME1-NME2, TRPC4AP	
GO:0016820~hydrolase activity, acting on acid anhydrides, catalyzing transmembrane movement of substances	4.44E-02	ATP5D, ATP5E, ATP6V0E1, ATP1B3, ATP6AP1, ATP5B, ATP6V1G1, ATP6V1B2, ATP5G1, ATP6V0B, ATP6V0C, ATP2B1, ATP5O, ATP5J, ATP5F1, ATP6V1H, ATP1A1, ATP6V1F, ATP2C1, ATP2A3, ATP6V1E1, ATP5C1, TRPC4AP, ATP5A1, ASNA1	5.31E-01
GO:0015631~tubulin binding	4.45E-02	B4GALT1, ALDOA, GABARAPL2, ARHGEF2, GABARAPL1, SNCA, POLB, GABARAP, BCL2L11, UXT, ATG4D, NME1-NME2, BLOC1S2, FYN, TARDBP, ARL8A, PAFAH1B1, ARL8B, MAPRE1, PRNP, EMD, DNM2, HDAC6	5.28E-01
GO:0042623~ATPase activity, coupled	4.55E-02	XRCC5, ATP5D, ATP5E, ATP6V0E1, ATP1B3, ATP6AP1, ATP5B, XRCC6, ATP2B1, DDX17, DHX38, DDX24, ATP5O, ATP5J, CCNH, ATP6V1H, MYH9, ATP6V1F, EIF4A3, ATP2C1, EIF4A2, EIF4A1, ATP5C1, MYO18A, SMARCA4, MYL6, MYO9B, ATP6V1G1, ATP6V1B2, ATP5G1, ATP6V0B, ATP6V0C, DDX3X, DDX49, DDX60L,	5.32E-01

		CHD2, DHX15, DDX41, CHD4, DHX58, HSPA8, DHX9, ATP5F1, ATP1A1, DDX5, DDX56, CRBN, ATP2A3, ATP6V1E1, CCT8, TRPC4AP, ATP5A1, ASNA1	
GO:0046982~protein heterodimerization activity	4.61E-02	JDP2, VAPA, MCL1, ADORA2A, HEXA, HEXB, PML, PDSS1, TGFB1, ITGAM, PEF1, MAX, FOS, PPP2CA, NPM1, GTF2A2, SUPT4H1, RUNX1, TOP2B, SUPT5H, IRAK2, IRAK1, PPP2R1A, CEBPB, CAPNS1, CD3D, IKZF1, SUCLG1, ADIPOR1, NR4A1, FOXP1, USF2, CYBA, P2RX4, CYBB, BAX, BNIP3L, TRPC4AP, SDCBP, IKBKB, EXT1, BIN1	5.33E-01
GO:0032555~purine ribonucleotide binding	4.66E-02	XRCC5, HRAS, XRCC6, RAB1B, CCT2, CCT3, CLK1, RAB1A, KIFC3, KIF13A, ACTG1, ATP2B1, DDX17, DHX38, CLK3, RAB28, ILK, MAP3K8, RAB24, TLK2, RAB20, GTPBP2, GTPBP1, YARS, GTPBP4, GBP5, RAN, NCF1, SUCLG1, PIM1, UBE2J1, EIF2S3, PIM3, PIM2, UBE2J2, MYH9, GLUL, EIF2AK1, MAPK6, ATP2C1, CAMK1, TUBA4A, ARL8A, RAB13, ARL8B, RAB10, MYO18A, COASY, GNAI2, PFKFB3, CHKB, FARS2, UBA7, STK17B, OAS1, MYO9B, ADRBK1, ARF6, CDC34, ARF5, NAGK, ATP6V0B, MOV10, RAC2, RAC1, DDX60L, SUPV3L1, DHX58, DHX9, MAP2K2, MAP2K3, TAOK3, SPHK1, GARS, DDX5, CCT7, OASL, CCT5, CCT4, ATP2A3, UBA1, ARF3, ARF4, CCT8, GRK6, RIT1, GK, ATP5D, SPG7, ABCF3, RAB5C, ATP5B, ZNF12, QARS, CKB, ACTR3, NUBP1, SRPR, TUBB6, DOCK10, TOP2B, AGAP3, RAP2C, LYN, PHKG2, RAB4B, NDUFA13, DGUOK, CCT6A, PI4KB, NDUFA10, EIF4A3, CDK11A, ADK, EIF4A2, EIF4A1, GNAS,	

		ARL4C, MAP3K11, TUFM, MKNK2, HK2, HK1, ITM2B, GCH1, DGKA, ACSL1, STK40, PTK2B, HK3, DHX15, RAB11A, CERK, ACSL3, ACSL5, MARS, RAB2A, CSNK1A1, NADK, ATP1A1, RAB32, RAB31, PAPOLA, CSNK1D, RPS6KA1, ILF2, PYGL, GSK3A, FYN, CSNK1E, GSK3B, ARAF, RAB34, ATP5A1, PGK1, DNM2, SEPT5, GNA15, NRBP1, ATP6AP1, EIF5, RPS6KB2, CASK, MTHFD1L, NLRC5, IDH3G, AAK1, DDX24, RALA, PRKACA, GNL2, MX1, SAR1B, MX2, SAR1A, PDXK, TWF2, CSNK1G2, STK24, EFTUD2, EEF2, RBKS, HNRNPU, MAP4K3, PNPLA8, MAP4K4, RIOK3, CLPP, RIPK2, SEPT6, GBP4, GBP2, SEPT9, GBP1, SMARCA4, GPN2, RAB7A, FGR, MVD, STK10, MAPKAPK3, MAP4K1, ABCA1, MAPKAPK2, KARS, SRC, UBE2D3, UBE2D2, DDX49, DDX3X, TAF9, HSPE1, UBE2D1, RUNX1, DDX41, RUNX3, CSF1R, EEF1A1, RAB8B, MYO1G, AK2, MYO1F, P2RX4, DDX56, PLK3, PSMC5, VCP, PSMC4, PSMC3, PSMC2, PRKAR1A, RAP1A, RAP1B, IKBKB, ASNA1, FPGS, GUK1, MYLK, UBE2E2, UBE2E1, HSP90AB1, PRKAG2, RHOQ, FES, RHOU, CDC42, WARS, ACTR1A, RHOA, RHOC, PAK1, TUBA1A, TUBA1B, RHOF, AKT3, TUBA1C, RHOG, RHOH, IRAK2, IRAK1, SGK1, UBE2A, HSP90AA1, PFKL, SARS, NADSYN1, MINK1, UBE2F, PKN1, UBE2I, UBE2H, PRKCE, NLRP3, UBE2B, PRKCD, CDK5, NLRP1, GAK, UBE2N, TYK2, PRKD2, TARS, UBE2K, UBE2M, PGS1, UBE2Z, TRIB1, UBE2R2, CHD2, HSPA4, HSPA5, EHD1,	

		CHD4, HSPA8, EHD4, HSPA9, ACTB, TCP1, HCK, RAF1, DRG1, UBE2Q1, HSP90B1, NME1-NME2, TRPC4AP	
GO:0032553~ribonucleotide binding	4.66E-02	XRCC5, HRAS, XRCC6, RAB1B, CCT2, CCT3, CLK1, RAB1A, KIFC3, KIF13A, ACTG1, ATP2B1, DDX17, DHX38, CLK3, RAB28, ILK, MAP3K8, RAB24, TLK2, RAB20, GTPBP2, GTPBP1, YARS, GTPBP4, GBP5, RAN, NCF1, SUCLG1, PIM1, UBE2J1, EIF2S3, PIM3, PIM2, UBE2J2, MYH9, GLUL, EIF2AK1, MAPK6, ATP2C1, CAMK1, TUBA4A, ARL8A, RAB13, ARL8B, RAB10, MYO18A, COASY, GNAI2, PFKFB3, CHKB, FARS2, UBA7, STK17B, OAS1, MYO9B, ADRBK1, ARF6, CDC34, ARF5, NAGK, ATP6V0B, MOV10, RAC2, RAC1, DDX60L, SUPV3L1, DHX58, DHX9, MAP2K2, MAP2K3, TAOK3, SPHK1, GARS, DDX5, CCT7, OASL, CCT5, CCT4, ATP2A3, UBA1, ARF3, ARF4, CCT8, GRK6, RIT1, GK, ATP5D, SPG7, ABCF3, RAB5C, ATP5B, ZNF12, QARS, CKB, ACTR3, NUBP1, SRPR, TUBB6, DOCK10, TOP2B, AGAP3, RAP2C, LYN, PHKG2, RAB4B, NDUFA13, DGUOK, CCT6A, PI4KB, NDUFA10, EIF4A3, CDK11A, ADK, EIF4A2, EIF4A1, GNAS, ARL4C, MAP3K11, TUFM, MKNK2, HK2, HK1, ITM2B, GCH1, DGKA, ACSL1, STK40, PTK2B, HK3, DHX15, RAB11A, CERK, ACSL3, ACSL5, MARS, RAB2A, CSNK1A1, NADK, ATP1A1, RAB32, RAB31, PAPOLA, CSNK1D, RPS6KA1, ILF2, PYGL, GSK3A, FYN, CSNK1E, GSK3B, ARAF, RAB34, ATP5A1, PGK1, DNM2, SEPT5, GNA15, NRBP1, ATP6AP1, EIF5, RPS6KB2,	5.33E-01

		CASK, MTHFD1L, NLRC5, IDH3G, AAK1, DDX24, RALA, PRKACA, GNL2, MX1, SAR1B, MX2, SAR1A, PDXK, TWF2, CSNK1G2, STK24, EFTUD2, EEF2, RBKS, HNRNPU, MAP4K3, PNPLA8, MAP4K4, RIOK3, CLPP, RIPK2, SEPT6, GBP4, GBP2, SEPT9, GBP1, SMARCA4, GPN2, RAB7A, FGR, MVD, STK10, MAPKAPK3, MAP4K1, ABCA1, MAPKAPK2, KARS, SRC, UBE2D3, UBE2D2, DDX49, DDX3X, TAF9, HSPE1, UBE2D1, RUNX1, DDX41, RUNX3, CSF1R, EEF1A1, RAB8B, MYO1G, AK2, MYO1F, P2RX4, DDX56, PLK3, PSMC5, VCP, PSMC4, PSMC3, PSMC2, PRKAR1A, RAP1A, RAP1B, IKBKB, ASNA1, FPGS, GUK1, MYLK, UBE2E2, UBE2E1, HSP90AB1, PRKAG2, RHOQ, FES, RHOU, CDC42, WARS, ACTR1A, RHOA, RHOC, PAK1, TUBA1A, TUBA1B, RHOF, AKT3, TUBA1C, RHOG, RHOH, IRAK2, IRAK1, SGK1, UBE2A, HSP90AA1, PFKL, SARS, NADSYN1, MINK1, UBE2F, PKN1, UBE2I, UBE2H, PRKCE, NLRP3, UBE2B, PRKCD, CDK5, NLRP1, GAK, UBE2N, TYK2, PRKD2, TARS, UBE2K, UBE2M, PGS1, UBE2Z, TRIB1, UBE2R2, CHD2, HSPA4, HSPA5, EHD1, CHD4, HSPA8, EHD4, HSPA9, ACTB, TCP1, HCK, RAF1, DRG1, UBE2Q1, HSP90B1, NME1-NME2, TRPC4AP	
GO:0031267~small GTPase binding	4.90E-02	NXT1, FMNL1, RILP, STX4, ARHGEF2, DENND5A, NCF2, DIAPH1, MYO9B, ABCA1, FLNA, ANXA2, PFN1, MAPKAP1, IPO5, RAP1A, PAK1, DOCK10, GGA2, GRASP, RHOH, MAP3K11, RIN3	5.47E-01
GO:0005375~copper ion transmembrane transporter	4.91E-02	SLC11A2, CCS, SLC31A2, SLC31A1	5.45E-01

activity			
GO:0010860~proteasome regulator activity	4.91E-02	PSMF1, ADRM1, PSME1, PSME2	5.45E-01
GO:0042979~ornithine decarboxylase regulator activity	4.91E-02	OAZ2, OAZ1, PRLR, AZIN1	5.45E-01
GO:0008266~poly(U) RNA binding	4.91E-02	ATXN1, PABPC4, MSI2, HNRNPH1	5.45E-01
GO:0016504~peptidase activator activity	4.94E-02	SIVA1, CARD8, TNFRSF10B, PYCARD, RPS27L, BCL2L13, CASP1, NLRP1	5.43E-01
GO:0031072~heat shock protein binding	5.01E-02	MVD, SNCA, NFKBIA, DNAJB12, STUB1, AHR, CDC37, GAK, DNAJC15, DNAJB11, DNAJC8, DNAJA1, DNAJC7, DNAJB2, DNAJC4, DNAJC1, DNAJB6, HDAC6	5.44E-01
GO:0017124~SH3 domain binding	5.14E-02	MTSS1, USP8, NCF1, INPPL1, HCLS1, BAIAP2, DENND1A, ARHGAP17, EVL, SIRPA, VASP, PTPN12, ELMO1, SH3BGRL, PLSCR1, CYBA, ILK, QKI, SYNJ2, MICAL1, ADAM19, ADAM15	5.50E-01
GO:0017048~Rho GTPase binding	5.35E-02	FMNL1, PFN1, ARHGEF2, NCF2, DIAPH1, MYO9B, PAK1, DOCK10, FLNA, MAP3K11, RHOH	5.61E-01
GO:0008047~enzyme activator activity	5.53E-02	GM2A, PRKAG2, RPS27L, ARHGAP17, ARHGAP15, ARHGAP4, HMHA1, SMAP2, SMAP1, CASP1, NRG1, RAPGEF2, DNAJC1, AGAP3, ARHGAP9, DBNL, ARHGEF1, MADD, ARHGAP24, BCL2L13, PRKCD, NLRP1, ARHGAP26, ARHGAP30, TNFRSF10B, BNIP2, SIPA1L1, ACAP1, RIN2, RIN3, SIVA1, CXCL1, ARFGAP2, ARFGAP3, AGFG1, SIPA1, RABGAP1L, AZIN1, CTSA, MYO9B, ADAP1, DOCK2, ALOX5AP, PYCARD, TBC1D7, TBC1D1, ARHGDIB, GDI1, CARD8, GDI2, ABR, PSAP, RGS1, PRLR, RABEP1, RGS2, GMFG, CHN2, AHSA2, AHSA1, ARAP1, DNAJB6, MGST2	5.70E-01
GO:0016765~transferase	5.99E-02	FDPS, PDSS1, RABGGTB,	5.96E-01

activity, transferring alkyl or aryl (other than methyl) groups		RABGGTA, FDFT1, MGST3, NANS, GSTK1, DHPS, GSTO1, MGST1, GSTP1, MGST2	
GO:0031202~RNA splicing factor activity, transesterification mechanism	6.09E-02	BCAS2, SF3B1, PRPF8, TRA2B, SLU7, SNRNP40, PRPF3, TXNL4A	5.99E-01
GO:0051020~GTPase binding	6.12E-02	NXT1, FMNL1, RILP, STX4, ARHGEF2, DENND5A, NCF2, DIAPH1, MYO9B, ABCA1, FLNA, ANXA2, PFN1, MAPKAP1, IPO5, RAP1A, PAK1, BIN1, DOCK10, GGA2, GRASP, RHOH, MAP3K11, RIN3	5.97E-01
GO:0019955~cytokine binding	6.12E-02	PLP2, HAX1, CCR1, TNFRSF14, CD74, CCRL2, IFNAR2, TNFRSF1A, TNFRSF1B, CCR7, CD36, PRLR, CCR5, CXCR4, IL10RB, IL4R, IL10RA, CSF3R, IL15RA, IL2RG, THBS1, IFNGR2, IFNGR1, CSF1R	5.97E-01
GO:0001849~complement component C1q binding	6.24E-02	C1QBP, CD93, CALR	6.01E-01
GO:0030911~TPR domain binding	6.24E-02	HSP90AB1, HSP90AA1, STUB1	6.01E-01
GO:0042296~ISG15 ligase activity	6.24E-02	UBE2L6, UBE2E2, UBE2E1	6.01E-01
GO:0030693~caspase activity	6.24E-02	CFLAR, CASP4, CRADD	6.01E-01
GO:0016667~oxidoreductase activity, acting on sulfur group of donors	6.26E-02	GLRX3, TXNL1, TXNDC12, MSRA, TXNDC17, TXN2, GSTK1, TXNRD2, SH3BGRL3, QSOX1, GLRX	5.98E-01
GO:0008060~ARF GTPase activator activity	6.27E-02	ARFGAP2, SMAP2, ARFGAP3, SMAP1, AGFG1, ACAP1, ARAP1, AGAP3, ADAP1	5.96E-01
GO:0003724~RNA helicase activity	6.27E-02	EIF4A3, DHX9, DDX17, DDX56, DDX3X, DHX38, DDX24, DHX15, DDX5	5.96E-01
GO:0045182~translation regulator activity	6.27E-02	MRPL28, PAIP2, RPS14, MRPL18, RPS9, RPS27L, CELF1, ANKHD1, PABPC1	5.96E-01
GO:0042162~telomeric DNA binding	6.49E-02	XRCC5, HNRNPA2B1, XRCC6, HNRNPD, TINF2, NCL	6.05E-01
GO:0033558~protein deacetylase activity	6.49E-02	HDAC5, HDAC3, HDAC1, MTA2, HDAC9, HDAC6	6.05E-01
GO:0004407~histone deacetylase activity	6.49E-02	HDAC5, HDAC3, HDAC1, MTA2, HDAC9, HDAC6	6.05E-01
GO:0004722~protein serine/threonine phosphatase	7.06E-02	PPM1F, PPP6C, MTMR3, PPM1G, PPP1CA, CYCS,	6.34E-01

activity		PPM1A, PPP1R15B, PPP4C, PPP1CB, PTEN, DUSP6	
GO:0060589~nucleoside-triphosphatase regulator activity	7.06E-02	GRPEL1, DENND5A, RASGEF1B, RGL4, ARHGAP17, ARHGAP15, ARHGAP4, HMHA1, SMAP2, SMAP1, DOCK10, RAPGEF2, DNAJC1, AGAP3, EIF2B5, RHOH, ARHGAP9, STX4, ARHGEF2, ARHGEF1, MADD, MINK1, ARHGAP24, WAS, ARHGAP26, ARHGAP30, MAP4K3, MAP4K4, BNIP2, SIPA1L1, ACAP1, IPO5, RIN2, ATPIF1, RIN3, ARFGAP2, ARFGAP3, AGFG1, CYTH1, SIPA1, CYTH4, MAP4K1, AKAP13, RABGAP1L, CDC42SE1, MYO9B, CYTH2, ADAP1, PLEKHG2, DOCK2, TAGAP, RASGRP4, RASGRP2, TBC1D7, TBC1D1, IQSEC1, ARHGDIB, GDI1, RILP, SERGEF, GDI2, ABR, DOCK5, VAV1, RALGDS, GPS2, ANXA2, RGS1, RGS2, RABEP1, CHN2, AHSA2, AHSA1, DNAJB6, ARAP1	6.30E-01
GO:0043566~structure-specific DNA binding	7.11E-02	XRCC5, HMGB2, JDP2, CNBP, RAD23A, XRCC6, ZNF12, KIN, YBX1, CTNNB1, FUBP1, FOS, PCBP1, BCL6, ERCC1, KLF6, SSBP4, SSBP3, SSBP2, SSBP1, SUB1, HNRNPA2B1, MECP2, SMAD3, TREX1, IFI16, HNRNPA1, JUNB, USF2, KLF4	6.30E-01
GO:0005048~signal sequence binding	7.12E-02	SRP14, BRE, NFKBIA, KDELR1, KPNA2, SSR2, SSR3	6.27E-01
GO:0048029~monosaccharide binding	7.25E-02	ALDOA, TALDO1, PFKL, PYGL, LGALS1, LGALS2, HK2, FBP1, M6PR, MANBA, LGALS9	6.31E-01
GO:0043027~caspase inhibitor activity	7.25E-02	TNFAIP8, SNCA, TNFSF14, PRDX5, HSPA5	6.27E-01
GO:0043014~alpha-tubulin binding	7.25E-02	B4GALT1, SNCA, ARL8A, ARL8B, HDAC6	6.27E-01
GO:0050839~cell adhesion molecule binding	7.46E-02	P2RX4, EZR, PSEN1, NUMB, PVRL2, MSN, FXYD5, CTNNA1, CTNNB1	6.35E-01
GO:0060090~molecular adaptor activity	7.61E-02	MTSS1, GRB2, BAIAP2, SRC, HSH2D, SLA, ARHGAP4, NCK2, SH3BGRL, LAT, CRKL, SDCBP,	6.39E-01

		CHN2, SH2B2, BIN3, PDCD6	
GO:0010843~promoter binding	8.36E-02	XRCC5, IKZF1, XRCC6, TFE3, TAF7, SMAD3, NFKB1, CTNNB1, HDAC5, TAL1, FOS, KDM1A, TAF9, TCF12	6.72E-01
GO:0031625~ubiquitin protein ligase binding	8.59E-02	CUL3, UBE2A, LYN, UBE2K, PML, NFKBIA, SMAD3, SKIL, UBE2B, RNF40	6.79E-01
GO:0005484~SNAP receptor activity	8.74E-02	STX5, STX4, NPEPL1, STX3, STX11, STX10, VTI1A	6.83E-01
GO:0005164~tumor necrosis factor receptor binding	8.74E-02	SIVA1, TNFSF10, TNFSF13B, BRE, TNFSF14, TNFSF13, TNFSF8	6.83E-01
GO:0005544~calcium-dependent phospholipid binding	8.85E-02	ANXA6, ANXA7, ANXA11, ANXA1, CPNE1, ANXA5, MCTP1, ANXA2	6.84E-01
GO:0017016~Ras GTPase binding	9.03E-02	NXT1, FMNL1, RILP, STX4, ARHGEF2, DENND5A, NCF2, DIAPH1, MYO9B, FLNA, ANXA2, PFN1, MAPKAP1, IPO5, RAP1A, PAK1, DOCK10, RHOH, MAP3K11, RIN3	6.89E-01
GO:0051015~actin filament binding	9.82E-02	DBNL, ACTN1, MYO9B, ARPC4, MYH9, GAS7, FLNA, UXT, CORO1A, EZR, SVIL, VPS16, LCP1	7.18E-01
GO:0001948~glycoprotein binding	9.89E-02	PTPRC, CSNK1G2, FYN, LGALS1, ITGB2, CD4, THBS1, ITGAM, FLNA, OS9	7.17E-01

Molecular Function (MF_FAT) associated with genes having decreased expression in **HNP_NeoHep** with respect to HNP_Monocyte

Term	PValue	Genes	Benjamini
GO:0003723~RNA binding	1.45E-11	NCBP1, NAF1, CPEB2, ZC3HAV1, GAR1, RPL15, SNRPD1, SRP19, ISG20, NONO, FUBP1, PCBP1, TARDBP, EIF1AY, U2AF1, SYNJ2, QKI, DDX21, LSM1, ZFP36, RPL35A, SNRPA1, EXOSC8, SF3B14, MAGOH, HNRNPA2B1, RPS4X, MBD2, HNRNPU, PSMA1, EIF4A3, RPS16, LARP7, RPS14, RPS13, RBM39, THOC2, SNRPG, IFIH1, TRA2B, TRA2A, SNRPB2, RPS15A, RPL37, OAS1, ZFP36L1, RPS25, HNRNPA3, HNRNPL, HNRNPK, RPL7, RPL31, RPL34, RPL9, RPL3, HNRNPD, RPL5, HNRNPC, RPS20, ZBP1, CSTF3, AIMP1, RPL26, RPL24, NXF1, DDX5, FXR1, RPS7, OASL, HNRNPH3, HNRNPH2, EIF4E, RPL22, RPL21, ZRANB2, HNRNPH1	1.12E-08
GO:0003735~structural constituent of ribosome	1.35E-10	MRPS36, RPL15, RPS15A, RPL37, RPL22L1, CYLD, RPL7, RPL31, RPL34, RPL9, RPL3, RPL5, RSL24D1, RPS20, RPS21, MRPL32, RPS23, RPL36AL, RPS24, RPL35A, RPL26, RPL24, RPS6, RPS4X, RPS7, HNRNPH2, RPS16, RPL22, RPS14, RPL21, RPS13	5.22E-08
GO:0005125~cytokine activity	8.09E-08	CXCL1, CSF3, CSF2, CCL2, IL16, CXCL3, IL19, CXCL2, TNFSF14, PF4, IL15, PF4V1, CCL7, CXCL10, IL23A, CCL20, IL1B, IL1A, IL6, CMTM2, IL8, AIMP1, IL1RN, TNFSF8, OSM, INHBA, TNFSF10, PPBP, CMTM5	2.09E-05
GO:0008009~chemokine activity	3.61E-05	CXCL1, CCL2, PPBP, IL8, CCL20, CXCL3, CXCL2, PF4, PF4V1, CCL7, CXCL10	6.98E-03

GO:0042379~chemokine receptor binding	6.43E-05	CXCL1, CCL2, PPBP, IL8, CCL20, CXCL3, CXCL2, PF4, PF4V1, CCL7, CXCL10	9.92E-03
GO:0033549~MAP kinase phosphatase activity	2.28E-03	DUSP5, DUSP2, DUSP1, DUSP8, DUSP6	2.56E-01
GO:0017017~MAP kinase tyrosine/serine/threonine phosphatase activity	2.28E-03	DUSP5, DUSP2, DUSP1, DUSP8, DUSP6	2.56E-01
GO:0003729~mRNA binding	3.11E-03	ZFP36, ZFP36L1, EIF4A3, RPL7, RPS14, HNRNPD, RPS13, HNRNPC, NXF1, MBD2	2.92E-01
GO:0030246~carbohydrate binding	7.15E-03	CCL2, TLR2, PF4, PF4V1, CLEC10A, CCL7, ASGR2, CD44, CD93, CLEC4E, CD69, CLEC2B, FCN1, CLEC4A, CLEC4D, THBS1, PTX3, OLR1, SELL, LGALS2, NLRP3, TNFAIP6, CLEC12A, RPL22, SIGLEC5, HBEGF, CLEC7A, CLEC1B	5.01E-01
GO:0046982~protein heterodimerization activity	7.62E-03	IRAK2, CEBPB, CD3D, IKZF1, MCL1, ADORA2A, CD3E, ALG2, NR4A2, NR4A1, PDSS1, FOXP1, INHBA, IRAK3, GTF2A2, BIN1, RUNX1, EXT1, TOP2B	4.82E-01
GO:0043566~structure-specific DNA binding	1.64E-02	EGR1, KLF6, HMGB2, SSBP2, SUB1, HNRNPA2B1, ZNF12, JUNB, CTNNB1, FUBP1, PCBP1, MYC, KLF4, ZBP1	7.22E-01
GO:0003924~GTPase activity	1.80E-02	RAB2A, GTPBP4, GBP5, RAP2C, RAB9A, NCF1, RAB4A, GNG11, GEM, RAB1A, TUBA8, RAB28, ARL8B, GBP4, EHD1, GNL2, GBP2, GBP1	7.21E-01
GO:0001871~pattern binding	2.56E-02	CCL2, SELL, TLR2, PF4, PF4V1, NLRP3, CCL7, TNFAIP6, CD44, RPL22, HBEGF, CLEC7A, THBS1, PTX3	8.13E-01
GO:0030247~polysaccharide binding	2.56E-02	CCL2, SELL, TLR2, PF4, PF4V1, NLRP3, CCL7, TNFAIP6, CD44, RPL22, HBEGF, CLEC7A, THBS1, PTX3	8.13E-01
GO:0019899~enzyme binding	2.66E-02	CSF3, NXT1, DENND5A, PRKAG1, DIAPH2, PDLIM5, VIM, NFKBIA, RAB3IP,	8.00E-01

		CRADD, CTNNB1, TRIB1, TAL1, CDC42EP2, CUL5, CDKN2D, PLCB1, TOP2B, TPRKB, RHOH, UBE2A, CD3E, COTL1, UBE2B, ATG3, NLRP1, PLAUR, PTPN11, CORO1A, ADRB2, DUSP2, BTG1, CSTA, BIN1, GRASP	
GO:0046983~protein dimerization activity	2.67E-02	KYNU, MCL1, MVD, ADORA2A, ALG2, PDSS1, GCH1, IRAK3, RPL7, XBP1, GTF2A2, TOP2B, NFIL3, RUNX1, PTS, FOSL1, B4GALT1, IRAK2, CEBPB, NFE2, CD3D, AIMP1, IKZF1, CD3E, NR4A2, NR4A1, FOSB, JUNB, FOXP1, INHBA, CORO1A, ADRB2, PRLR, ATF7, BIN1, EXT1	7.77E-01
GO:0048487~beta-tubulin binding	3.48E-02	B4GALT1, GABARAPL1, ARL8B, EMD	8.39E-01
GO:0005529~sugar binding	3.60E-02	OLR1, SELL, LGALS2, CLEC10A, ASGR2, CD93, CLEC4E, CLEC12A, CD69, SIGLEC5, CLEC2B, FCN1, CLEC4A, CLEC7A, CLEC4D, CLEC1B	8.31E-01
GO:0005198~structural molecule activity	3.96E-02	MRPS36, RPL15, VIM, RPS15A, RPL37, RPL22L1, CYLD, LAMB3, RPL7, RPL31, RPL9, RPL34, RPL3, RPL5, RPS20, RSL24D1, RPS21, THBS1, MRPL32, RPS23, RPL36AL, RPS24, RPL35A, CD3E, ANXA1, RPL26, RPL24, RPS6, RPS4X, RPS7, TUBA8, SMTN, HNRNPH2, RPS16, CDC42SE2, RPL22, RPS14, RPL21, RPS13, CSTA	8.41E-01
GO:0019843~rRNA binding	4.24E-02	GAR1, RPL9, RPL37, RPL5, RPS4X	8.46E-01
GO:0004857~enzyme inhibitor activity	4.38E-02	PHACTR1, TESC, SOCS3, PKIG, ANXA1, SPINK1, TNFSF14, TRIB1, SAG, SERPINB9, CARD16, SERPINB8, CDKN2D, PI3, TNFAIP8, CARD17, SERPINB2, SLPI, CSTA, RHOH	8.39E-01
GO:0008138~protein	5.05E-02	DUSP5, DUSP2, DUSP1,	8.66E-01

tyrosine/serine/threonine phosphatase activity		DUSP8, PTEN, DUSP6	
GO:0008134~transcription factor binding	5.12E-02	HMGB1, NBN, YWHAZ, NMI, NFKBIB, NFKBIA, NFKB2, CITED4, RAB1A, CTNNB1, TRIB1, GTF2A2, BCL3, RARA, TAF9, NFIL3, NRG1, RUNX1, NFE2, SUB1, SRA1, RYBP, FOSB, DDX5, MXD1, MSC, JUNB, OASL, MTF1, BTG1, TGIF1, JMJD1C, LCOR	8.56E-01
GO:0019904~protein domain specific binding	5.33E-02	MTSS1, YWHAZ, NFE2, C9ORF89, CCDC88C, NCF1, CD3E, MBD2, PTEN, NLRP1, CRADD, LIN7A, PTPN11, CHMP1B, U2AF1, QKI, RIPK2, IL1B, SYNJ2, TAF9, ADAM19, GRASP, PMEPA1	8.55E-01
GO:0005539~glycosaminoglycan binding	5.85E-02	TNFAIP6, CCL2, CD44, RPL22, SELL, TLR2, HBEGF, PF4, PF4V1, NLRP3, THBS1, CCL7	8.69E-01
GO:0008234~cysteine-type peptidase activity	6.09E-02	CASP5, CYLD, CASP9, CARD16, CARD17, UCHL3, TNFAIP3, SENP5, CASP1, CRADD, CTSW, SENP6	8.69E-01
GO:0003743~translation initiation factor activity	6.10E-02	EIF4E, EIF3H, EIF1AY, EIF3F, EIF1B, GTF2B, EIF3M	8.58E-01
GO:0019955~cytokine binding	6.40E-02	CCRL2, PLP2, CCR7, CCR5, PRLR, IL1RAP, IL15RA, THBS1, IFNGR2, IFNGR1	8.61E-01
GO:0030528~transcription regulator activity	8.07E-02	MEF2C, CHURC1, EZH2, ZEB2, NFKB2, CITED4, CTNNB1, FUBP1, EPC1, ZNF350, TARDBP, GTF2A2, RARA, SIK1, NRG1, NFIL3, MYC, FOSL1, EGR1, ZNF33A, SSBP2, FOSB, MBD2, MXD1, MSC, GTF2B, JUNB, MED10, MTF1, BTG1, HES4, TGIF1, TGIF2, LCOR, HMGB2, CCRN4L, NMI, NFKBIB, ZNF131, ZFP36L1, TAL1, TSC22D3, STAT4, TSC22D2, ERCC6, RPL7, XBP1, POU2F2, GTF3C6, HNRNPD, BCL3, TAF9, RUNX1, ETV3, KLF6, MAFF, NFE2, CEBPB, IKZF1, KLF13, SUB1, JARID2, KLF10, SRA1, RYBP, NR4A2,	9.11E-01

		NR4A1, NR4A3, DDX5, FOXP1, ID2, ETS2, IRF7, CSRNP1, ATF7, ZRANB2, IRF1, HIVEP1, PHF5A, KLF4, DNAJB6, KLF3	
GO:0008135~translation factor activity, nucleic acid binding	8.21E-02	EIF4E, EEF1B2, EIF3H, EIF1AY, EIF3F, MTRF1L, EIF1B, GTF2B, EIF3M	9.07E-01
GO:0051087~chaperone binding	8.70E-02	PFDN4, HSPE1, AHSA2, DNAJB6	9.12E-01
GO:0008301~DNA bending activity	8.85E-02	HMGB1, HMGB2, HMGB3	9.09E-01
GO:0019210~kinase inhibitor activity	8.89E-02	SOCS3, CDKN2D, PKIG, TRIB1, RHOH	9.02E-01
GO:0004869~cysteine-type endopeptidase inhibitor activity	8.89E-02	CARD16, TNFAIP8, CARD17, TNFSF14, CSTA	9.02E-01
GO:0022890~inorganic cation transmembrane transporter activity	8.91E-02	SLC9A9, ATP2B1, SLC9A8, ATP5E, RHCG, ATP5S, SLC25A37, ATP5L, ATP1A1, COX7A2L, UQCRFS1, UQCRB	8.96E-01
GO:0019964~interferon-gamma binding	9.00E-02	IFNGR2, IFNGR1	8.91E-01
GO:0001872~zymosan binding	9.00E-02	CLEC7A, PTX3	8.91E-01
GO:0004906~interferon-gamma receptor activity	9.00E-02	IFNGR2, IFNGR1	8.91E-01
GO:0031202~RNA splicing factor activity, transesterification mechanism	9.61E-02	BCAS2, TRA2B, PRPF3, LSM1	9.00E-01

Molecular Function (MF_FAT) associated with genes having increased expression in **H_RM** with respect to H_Monocyte

Term	PValue	Genes	Benjamini
GO:0004222~metalloendopeptidase activity	7.50E-06	MMP10, KEL, MMP9, PAPLN, MMP14, MMP3, MMP2, ADAMDEC1, MMP1, ADAM9	0.00
GO:0008237~metallopeptidase activity	1.27E-04	MMP10, ACE, KEL, MMP9, PAPLN, MMP14, MMP3, MMP2, ADAMDEC1, MMP1, ADAM9	0.03
GO:0046870~cadmium ion binding	2.35E-04	MT1M, MT1E, MT1H, MT1G	0.03
GO:0004175~endopeptidase activity	3.47E-04	KEL, LGMN, MMP9, PAPLN, MMP3, MMP14, MMP2, MMP1, PIGK, MMP10, CTSK, CTSD, HTRA4, ADAMDEC1, ADAM9	0.04
GO:0004866~endopeptidase inhibitor activity	5.67E-04	A2M, C3, LXN, SERPINB7, CSTB, SPINK1, PAPLN, BCL2L1, TFPI2	0.05
GO:0030414~peptidase inhibitor activity	8.08E-04	A2M, C3, LXN, SERPINB7, CSTB, SPINK1, PAPLN, BCL2L1, TFPI2	0.06
GO:0004857~enzyme inhibitor activity	2.59E-03	A2M, C3, LXN, SERPINB7, APOC1, CSTB, SPINK1, PAPLN, BCL2L1, TFPI2, ANGPTL4	0.15
GO:0070011~peptidase activity, acting on L-amino acid peptides	4.82E-03	KEL, MMP9, LGMN, PAPLN, MMP3, MMP14, MMP2, MMP1, PIGK, MMP10, ACE, CTSK, CTSD, HTRA4, ADAMDEC1, ADAM9	0.23
GO:0008289~lipid binding	5.42E-03	RBP1, APOC1, RACGAP1, ABCG1, PIGK, STAC, PTGDS, RASGRP3, APOE, FABP3, PLA2G7, SCARB1, PLA2G4C, PLTP	0.23
GO:0019842~vitamin binding	6.66E-03	RBP1, P4HA2, P4HA1, SDS, EGLN3, SRR, PDXDC1	0.26
GO:0008233~peptidase activity	7.18E-03	KEL, MMP9, LGMN, PAPLN, MMP3, MMP14, MMP2, MMP1, PIGK, MMP10, ACE, CTSK, CTSD, HTRA4, ADAMDEC1, ADAM9	0.25

Molecular Function (MF_FAT) associated with genes having increased expression in **H_NeoHep** with respect to H_Monocyte

Term	PValue	Genes	Benjamini
GO:0042803~protein homodimerization activity	0.00	MTMR2, ALS2, C1QB, CADM1, SDS, APOE, IL6ST, ENO2, QPRT, DPP4, CRYBB2	0.62
GO:0008047~enzyme activator activity	0.01	ALS2, RASAL1, GAPVD1, RASGRP3, APOE, APOC1, MMP14, ARHGEF12, RACGAP1, RASA4	0.80

Molecular Function (MF_FAT) associated with genes having increased expression in **HNP_NeoHep** with respect to HNP_Monocyte

Term	PValue	Genes	Benjamini
GO:0008430~selenium binding	1.96E-06	GPX1, SELM, DIO2, GPX3, TXNRD3, TXNRD1, SEPP1, SEPN1	0.00
GO:0016209~antioxidant activity	2.60E-04	GPX1, APOE, GPX3, TXNRD3, TXNRD1, CYGB, MT3	0.07
GO:0016628~oxidoreductase activity, acting on the CH-CH group of donors, NAD or NADP as acceptor	4.08E-04	TM7SF2, PTGR1, DHCR7, AKR1C1, DHCR24	0.08
GO:0003941~L-serine ammonia-lyase activity	1.09E-03	SDS, SRR, SDSL	0.15
GO:0005543~phospholipid binding	2.20E-03	APOA2, PLA2G15, EPB41, APOE, APOC3, APOC1, SCARB1, SNX24, PHLDA3, PLEKHA2, ITPR2	0.23
GO:0008289~lipid binding	2.67E-03	LPL, PLA2G15, EPB41, RBP1, APOC1, TENC1, ITPR2, APOA2, PTGDS, APOE, APOC3, FABP3, FABP4, SCARB1, SNX24, PHLDA3, AKR1C1, PLTP, PLEKHA2	0.23
GO:0016841~ammonia-lyase activity	3.53E-03	SDS, SRR, SDSL	0.26
GO:0060228~phosphatidylcholine-sterol O-acyltransferase activator activity	3.53E-03	APOA2, APOE, APOC1	0.26
GO:0031406~carboxylic acid binding	6.57E-03	PLOD1, SRR, FABP3, APOC1, FABP4, ALDH1L2, AKR1C1, GCHFR, PC	0.39
